# Toward Higher Fatigue Resistance of Photochromic Polymer Layers Containing Novel Diarylethenes

**DOI:** 10.1021/acsomega.6c05374

**Published:** 2026-07-16

**Authors:** Mattes Plieth, André Eitzeroth, Henrik Hercht, Jingrun Zhang, Sven Nagorny, Christian Rembe, Jörg Adams, Andreas Schmidt

**Affiliations:** 1 Institute of Organic Chemistry, 26534 Clausthal University of Technology, Leibnizstr. 6, 38678 Clausthal-Zellerfeld, Germany; 2 Institute of Physical Chemistry, Clausthal University of Technology, Arnold-Sommerfeld-Str. 4, 38678 Clausthal-Zellerfeld, Germany; 3 Institute for Electrical Information Technology, 26534 Clausthal University of Technology, Leibnizstraße 10, 38678 Clausthal-Zellerfeld, Germany; 4 Department of NanoBiophotonics, 28296 Max Planck Institute for Multidisciplinary Sciences, Am Faβberg 11, 37077 Göttingen, Germany

## Abstract

For the use of diarylethenes (DAE) in optical microscopy with the aim of overcoming the Abbe limit, interdependent parameters must be coordinated. These include, in particular, the fatigue resistance ***X***_**A**_, the position of the isosbestic points (λ_isos_ > 400 nm), and the molar extinction coefficients of the closed form (ε_closed_ > 20,000 L/mol·cm). Thirteen photoswitchable DAEs were prepared and spectroscopically characterized (**λ**_**isos**_, ***ε***_**closed**_, quantum yields **φ**), among those a first example of a pyridazine substituted derivative. The spectroscopic properties of the closed form of known and hitherto unknown DAEs can be predicted using the spectroscopic Hammett equation, since the excitation energy differences *E*_T,R_ – *E*_T,H_/2.303·R·T correlate linearly with the Hammett parameters σ_p_. Matrix effects of eight different polymers in the spin-coated absorbance modulation layer (AML) on the properties of model DAEs were investigated. A correlation was found between the glass transition temperature *T*_g_ and the degradation rate of the photochrome. We found that replacing PMMA with ZEONEX480R results in a 3-fold increase in photostability. DFT calculations of the frontier orbital profiles of the DAEs as well as the changes in the geometric requirements of the 6π-electrocyclic ring closure provide insights into structure–property relationships.

## Graphical abstract

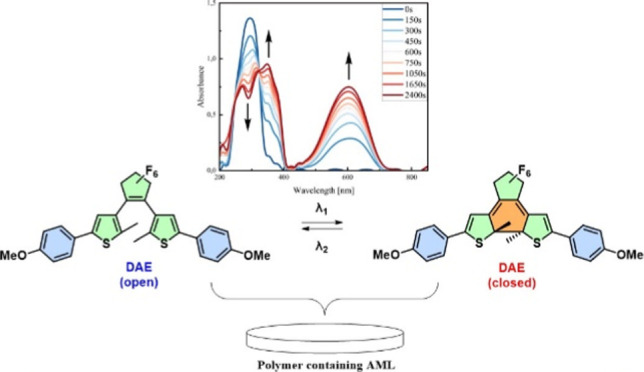

## 1 Introduction

Photochromic compounds are a class of molecules that undergo reversible structural changes upon irradiation, resulting in the formation of isomers with different optical and spectroscopic properties.^1–6^ While photochromes generally exhibit this fundamental behavior, the underlying switching mechanisms can vary considerably. For example, azobenzene **1** is switchable because it undergoes cis–trans isomerization,^7,8^ while punicine **2** undergoes a light-induced redox reaction to form radical cations and anions.^9–12^ In contrast, spiropyran **3** and spirooxazine **4**, under the action of UV light, ring open to a photomerocyanine form which can then ring close with visible light or thermally.^13,14^ Results of the crystal structure analysis indicate that the quinoidal resonance contributor of the spirooxazine predominates over the phenolic contributor.^15^ This indicates that electrocyclization is more likely than nucleophilic attack as the ring-closing mechanism. Another example of ring closure photochromism is exhibited by furylfulgide **5** ^16^ and diarylethenes **6**,^1,6,17^ which undergo 6π-electrocyclizations (Scheme 1). The latter class of compounds has been the subject of extensive research for over three decades.

**Scheme 1:**
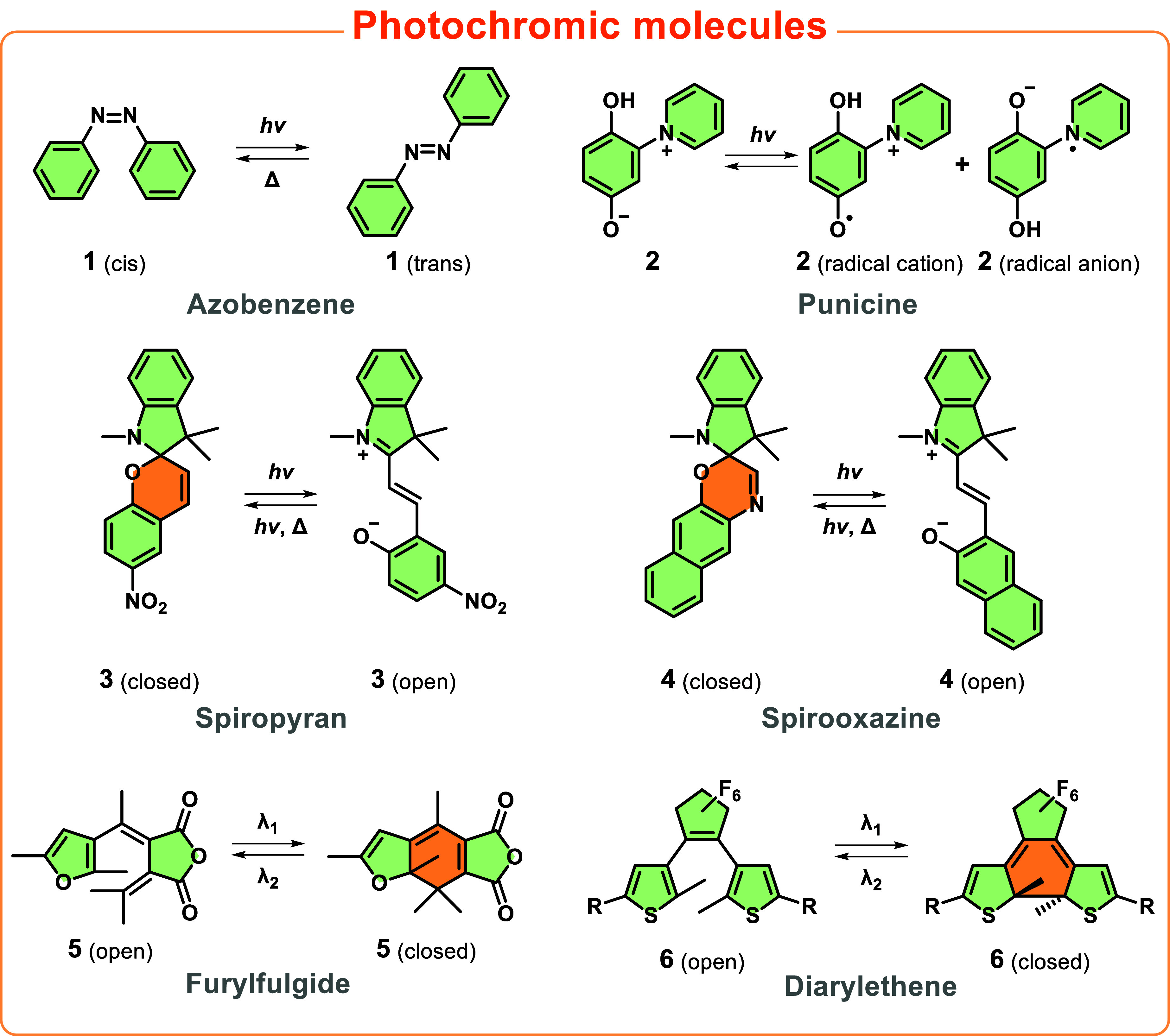
Different Examples of Photochromes: Azobenzene **1**, Punicine **2**, Spiropyran **3**, Spirooxazine **4**, Furylfulgide **5**, and Diarylethene **6**

Compared to other switchable molecules, diarylethenes (DAEs) are characterized in particular by their high thermal stability,^18^ exceptional fatigue resistance,^19,20^ and fast switching kinetics upon irradiation.^21^ These properties make them extremely attractive for applications such as optical data storage,^22,23^ photoswitchable devices,^24^ photopharmacology,^25,26^ and light-responsive materials^27^ or actuators.^1,28–31^ Their respective electrochemical parameters in their ring open and closed state make them interesting candidates for conducting materials in electronic devices.^32–35^

For applications in optical near field nanoscopy, systems consisting of photochromes and polymer matrices must be designed or identified that meet specific requirements. Until now, due to the diffraction-limited resolution of optical microscopes (Abbe limit^36,37^ ), light microscopic examinations of modern materials such as metals with grain sizes below 1 μm have not been possible. The further development of the principles underlying high-resolution far-field fluorescence microscopy is therefore the goal of intensive efforts. To this end, the surfaces or objects to be examined must be covered with a photochromic layer. This is switched to opaque by an activation laser with a wavelength of λ_1_ using a ring mode (doughnut mode) for a measuring laser with a wavelength of λ_2_. This creates a diaphragm for the measuring laser beam, whereby the photochrome is then switched back with a laser wavelength of λ_3_. With a suitable layer, this principle allows successive scanning of surfaces (Figure 1). A key advantage of this method is its ability to enable the investigation of living tissues through minimally invasive measurements, without the need for fluorescence markers.^38–40^

**Figure 1:**
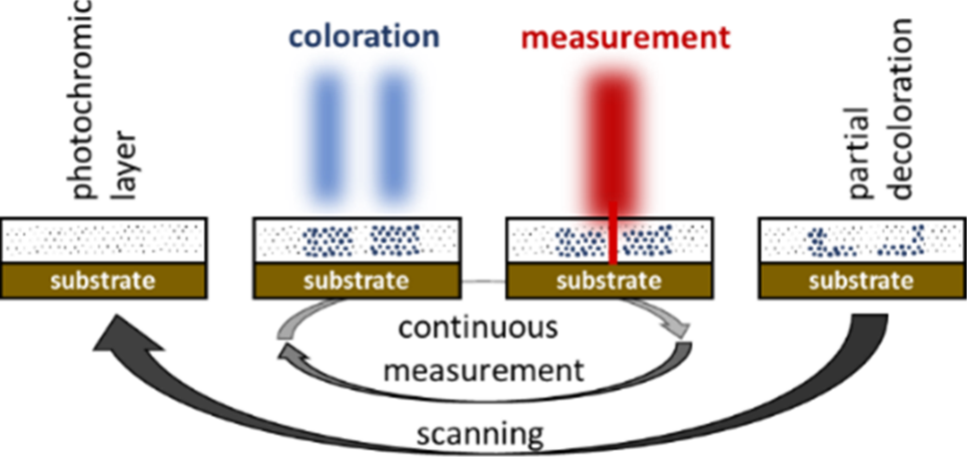
Imaging techniques based on the RESOLFT principle for the nanometer range.

Recent results from our research group have shown that DAEs have indeed promising potential for super-resolution reflection microscopy.^41–44^ In these studies, an absorbance modulation layer (AML) which contained poly­(methyl methacrylate) (PMMA) and the photochromic compound was coated on a substrate and examined using a technique known as absorbance modulation imaging (AMI). This approach achieved a 2.4-fold resolution enhancement beyond the diffraction limit.^41^ For such applications, photochromic molecules must be identified and developed that exhibit a strong absorption contrast between the isomeric forms and, advantageously, can be switched by only using visible or near-UV light to enable the use of more cost-effective laser systems and allow the investigation of living tissues.^41,45–50^ With regard to the latter aspect in particular, numerous results from the literature relating to photochromes in solution are not applicable.

For example, previous studies by our group have shown that β-methyl substituents of perfluorocyclopentene-based 1,2-bis­(thienyl)­ethene decrease the fatigue resistance of PMMA-based AMLs (Scheme 2).^42^ This observation contrasts with numerous reports on DAEs investigated in solution.^51,52^ Another class that has been extensively studied includes benzo­[*b*]­thiophene-substituted DAEs.^48,51,53–56^ Although these bulky substituents often enhance photostability in solution, they can increase the distance between the carbon atoms involved in the 6π–electrocyclization reaction.^57^ When this distance exceeds 4.2 Å, cyclization in the solid state is suppressed.^57,58^

**Scheme 2:**
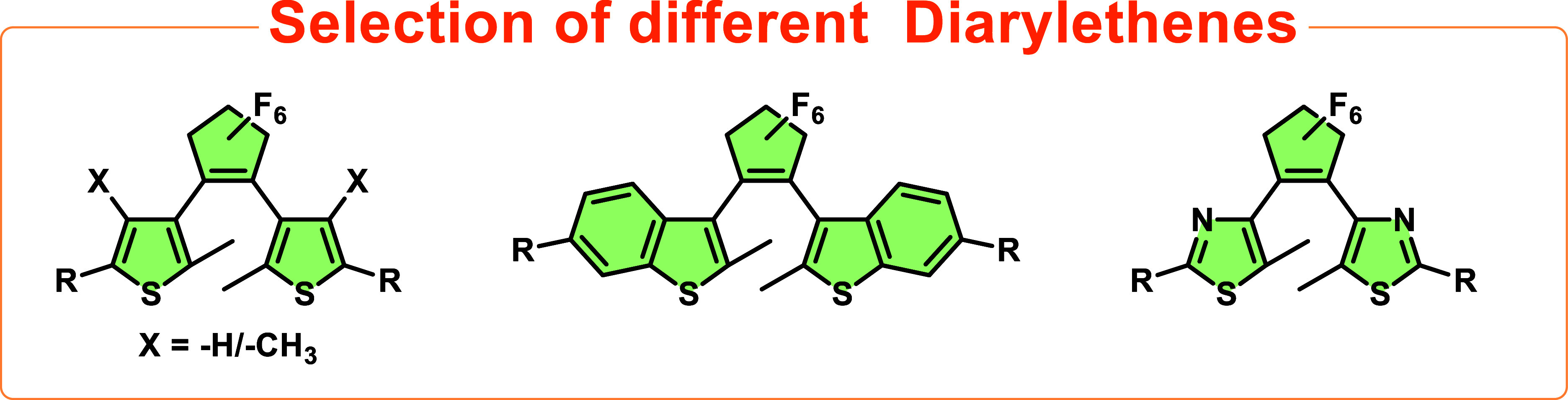
Selection of Different DAEs^a^

a Left: thiophene-substituted DAE with or without β-methyl groups. Middle: Benzo­[*b*]­thiophene-substituted DAE. Right: thiazole-substituted DAE.

DAEs with thiazole substituents have also been investigated as candidates for improved stability.^59–66^ While these compounds typically exhibit a slightly bathochromically shifted UV absorption, their visible absorption is often hypsochromically shifted, resulting in poor spectral separation and reduced switching efficiency when irradiated at the isosbestic point. Consequently, 3,3′-bis­(thienyl)­ethenes remain as promising candidates for solid-state switching. Despite sustained interest over the past two decades, systematic studies on their photostability in AMLs remain limited.^41–43,67–71^

In this study, we investigate several polymer/photochrome systems for AML use by varying the structure of both components. New diarylethenes were developed and prepared to improve the properties and meet the above-mentioned requirements.

## 2 Results and Discussion

### 2.1 Synthesis of DAEs

The synthesis of DAEs used in this study was carried out following modified literature procedures (Scheme 3).^72^ Substituents that are not prone to decomposition by strong bases can be introduced via a 2-step synthesis starting from 3,5-dibromo-2-methylthiophene **7** or 3-bromo-2-methylthiophene-5-boronic acid **8** (Scheme 3, **Strategy A**). The aryl substituents are first transferred onto the thiophene via Suzuki–Miyaura coupling using catalytic amounts of tetrakis­(triphenylphosphine)-palladium to give aryl-substituted thiophenes **9** [reaction step (**i)**]. Subsequently the bicyclic building blocks are then lithiated at the β-position of the thiophene ring of **9** using *n*-butyllithium (*n*-BuLi) followed by quenching the reaction mixture with octafluorocyclopentene [reaction step (**ii)**] to give the corresponding DAEs.

**Scheme 3:**
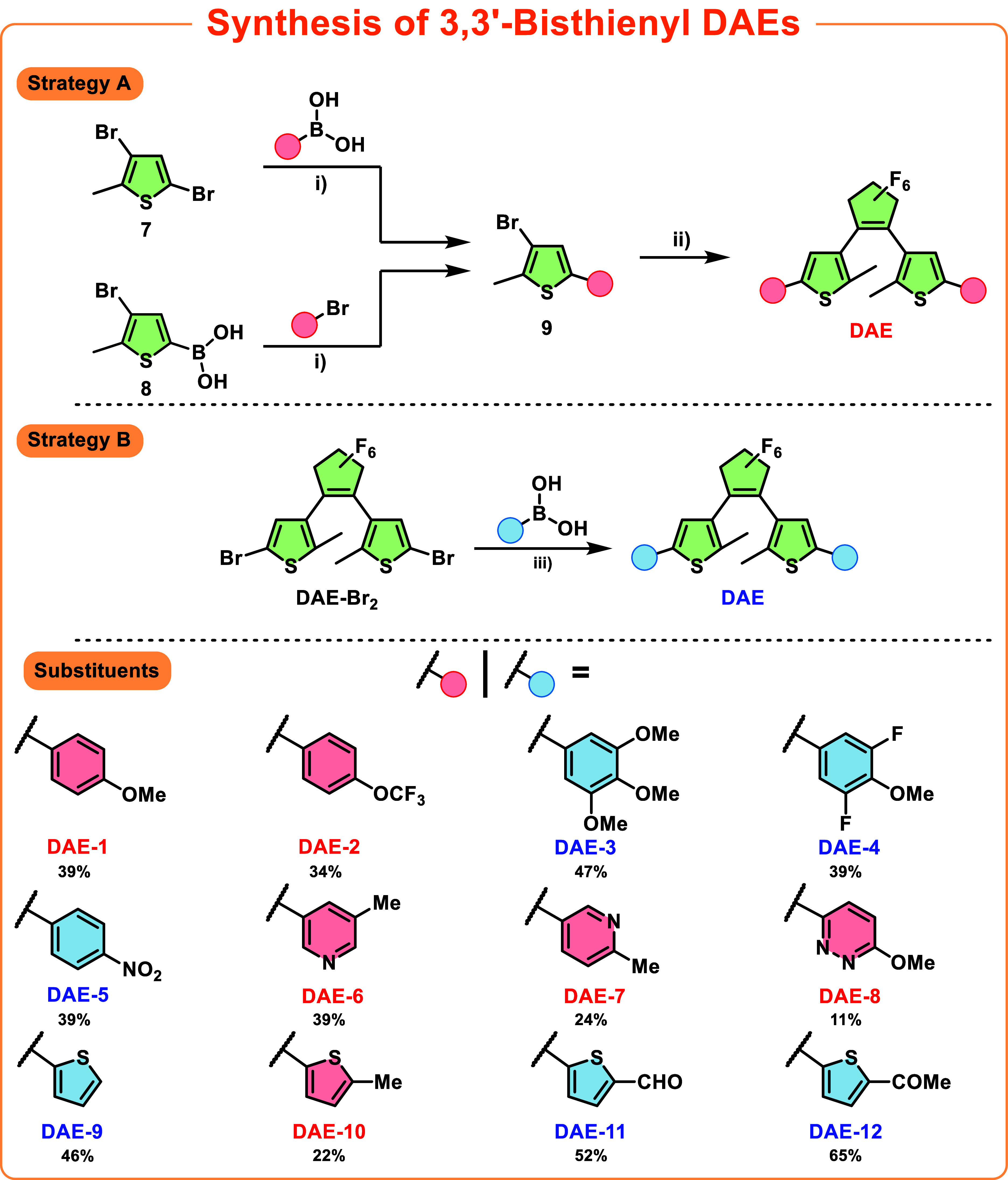
Design of 3,3′-Bisthienylethene DAEs^a^

a Above: synthesis strategies of DAEs either through initial Suzuki coupling of thiophene building blocks **7** or **8** and subsequent lithiation of the aryl-substituted thiophene compound **9** (strategy A) or through Suzuki–Miyaura coupling of **DAE-Br**_**2**_ (strategy B). Below: scope of the DAEs synthesized in this work. DAEs marked in red were synthesized following strategy A, DAEs written in blue were synthesized according to strategy B. (i) Pd­(PPh_3_)_4_, K_2_CO_3_, THF, H_2_O, reflux, 12 h (ii) *n*-BuLi, octafluorocyclopentene, −78 °C to rt, 12 h (iii) G3 SPhos Pd, K_3_PO_4_, dioxane, H_2_O, reflux, 4 h.

Substituents which readily undergo reactions with *n*-BuLi are most effectively introduced via Suzuki–Miyaura cross-coupling using terminally brominated **DAE-Br**_**2**_ as starting material (Scheme 3, **Strategy B**). In contrast to previous reports where Pd­(PPh_3_)_4_ was used,^73–76^ we employed (2-dicyclohexylphosphino-2′,6′-dimethoxybiphenyl) [2-(2′-amino-1,1′-biphenyl)]­palladium­(II)-methanesulfonate (SPhos Pd G3) as the catalyst, resulting in higher yields (39% to 65%) and significantly reduced reaction times [reaction step **iii)**].

Previous studies from our group identified **DAE-1** as a spectroscopically promising and readily accessible compound.^42^ On this basis, we synthesized the structurally related derivatives **DAE-2**, **DAE-3**, and **DAE-4**, which have not been previously reported. **DAE-5** to **DAE-8** constitute a series of nitrogen-containing DAEs, of which the latter three are new compounds. While DAEs bearing pyridine or nitro substituents are well documented,^42,77^ derivatives incorporating a pyridazine moiety such as **DAE-8** have, to the best of our knowledge, not been reported. Finally, we prepared **DAE-9** to **DAE-12**, a small set of thienyl-substituted molecular switches; among these, **DAE-12** is new, whereas **DAE-11** has not previously been subjected to UV spectroscopic analyses.^78^

To further induce a bathochromic shift, we designed a visible-light-switchable DAE based on a π-extension strategy (**DAE-13**, Figure 2A).^70,79,80^ **DAE-13** was synthesized via Wittig olefination of **DAE-11** using commercially available Ph_3_PCHCO_2_Et in refluxing THF, affording the product in excellent yield (88%). Although π-extension of DAEs through Wittig-type olefination has been reported previously,^81^ our protocol provided higher yields and proceeded without the need for a phase-transfer catalyst or additional base.

**Figure 2:**
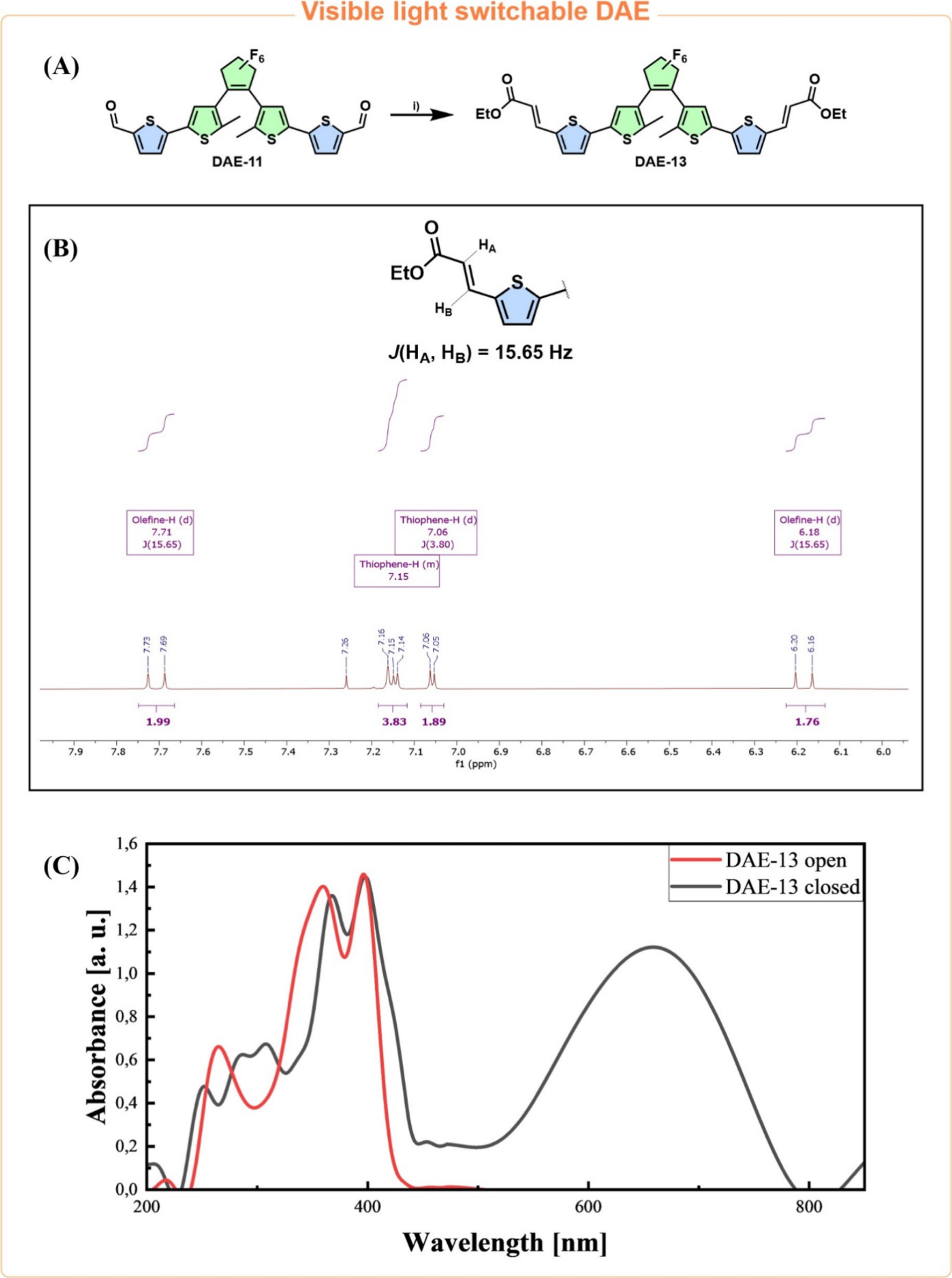
(A) Synthesis of **DAE-13** via Wittig olefination. (B) ^1^H NMR spectrum of **DAE-13** proving *E*-isomer. (C) UV/vis spectra showing the open (red) and the closed form (black) of **DAE-13** (**λ**_**isos**_ = 403 nm). Maxima of open and closed form are shown in Table S10 in Supporting Information. (i) Ph_3_PCHCO_2_Et, THF, reflux, 4 h.

Formation of the *E*-isomer was confirmed by ^1^H NMR spectroscopy through evaluation of the coupling constant (***J***) of the two olefinic protons (Figure 2B). A value of ***J*** = 15.65 Hz is characteristic for *E* configuration. As anticipated, UV/vis spectroscopy revealed an isosbestic point in the visible region (**λ**_**isos**_ = 403 nm; Figure 2C). Switching of **DAE-13** was initiated with an irradiation wavelength of 405 nm. The UV absorption maximum (**λ**_**UV,max**_ = 396 nm) was bathochromically shifted by 40 nm compared to related π-extended DAEs reported by Mattay et al.^81^ The pronounced bathochromic shift arises from the extended π-conjugated framework and the donor–acceptor architecture established by the electron-donating thiophene unit and the electron-withdrawing ester moiety.^82^ These findings clearly demonstrate that the Wittig-based synthetic strategy effectively induces the targeted bathochromic shift, thereby validating its suitability as a powerful approach for tuning the spectral properties of DAEs.

### 2.2 Examination of the Spectroscopic Properties of DAEs

In order to evaluate the suitability of the selected DAE for nanoscopy, we determined their key spectroscopic parameters. These included the position of the isosbestic point, the molar extinction coefficient at the visible absorption maximum of the closed form, and the quantum yields for both cyclization and cycloreversion. A bathochromically shifted isosbestic point enables switching at longer wavelengths, while a high molar extinction coefficient enhances absorbance contrast and thereby improves imaging resolution.^45^ The quantum yields were obtained by repeated switching between the open and closed forms under controlled irradiation conditions. All of these spectroscopic parameters were determined in solution (THF; concentration = 31 mg/L) in a closed vial without further degassing.

Beyond these intrinsic molecular properties, fatigue resistance within an AML represents a critical parameter for nanoscopy applications. Bianco and co-workers evaluated photostability by alternating irradiation with UV and visible light and quantifying the fraction of *degraded molecules* after 80 switching cycles (***X***_**D**_).^59^

To focus on the functionality of AMLs, we adapted this approach and defined fatigue resistance as the fraction of *active molecules* remaining after 80 cycles (***X***_**A**_). In these experiments, values approaching zero indicate extensive photobleaching, whereas values close to 1 reflect high stability. AMLs were prepared by spin coating a solution containing 25 wt % DAE and 75 wt % PMMA onto a sapphire glass disk.^42,43^ Measurements were conducted using the following experimental setup (Figure 3).^42^

**Figure 3:**
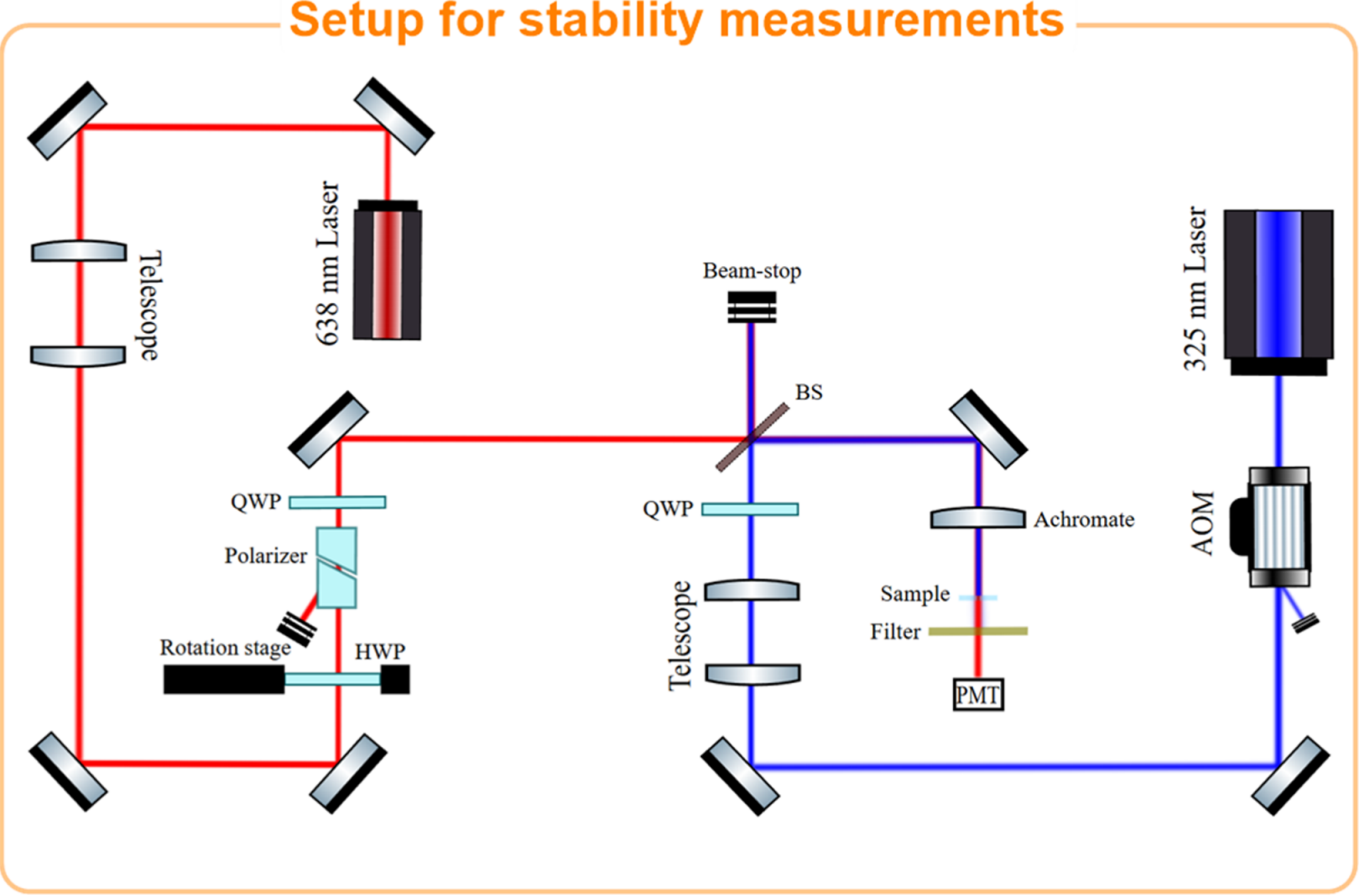
Experimental setup for the measurement of AML photostability in the switching mode. BS = beam splitter; QWP = quarter wave plate; AOM = acousto-optic modulator, HWP = half wave plate; PMT = photo multiplier tube.

Stability investigations were performed in a so-called *switching mode*, with irradiation at 325 nm to induce cyclization and at 638 nm to trigger ring opening. This protocol was selected to ensure direct comparability with previously reported fatigue resistance data^42,43^ and to closely mimic the operating conditions of the nanoscopic systems for which the AMLs are intended.^41^ Finally the absorbance of the AMLs at the visible maxima was monitored and the values after the first cycle and after 80 cycles were used to calculate ***X***_**A**_.

Shown in Table 1 are the DAEs with their wavelength of the isosbestic point **(λ**_**isos**_), the molar extinction coefficient of the closed form (***ε***_**closed**_), the quantum yield of the cyclization (φ_**o→c**_) and the ring opening reaction (φ_**c→o**_) as well as ***X***_**A**_ as a parameter for the fatigue resistance.

**Table 1:** Spectroscopic Parameters of Various DAEs^a^

molecule	**λ**_isos_ [nm]	*ε*_closed_ [M^–1^·cm^–1^]	***Χ***_**A**_	φ_o→c_	φ_c→o_
**DAE-1**	318	17,700	0.754	0.62	0.0032^a^
**DAE-2**	319	11,440	0.738	0.63	0.0086^a^
**DAE-3**	328	19,920	0.661	0.84	0.0019^a^
**DAE-4**	318	17,890	0.634	0.70	0.0043^a^
**DAE-5**	335	21,350	0.443	0.41	0.0045^b^
**DAE-6**	319	11,970	0.396	0.44	0.0040^a^
**DAE-7**	314	13,220	0.330	0.35	0.0028^a^
**DAE-8**	330	12,480	0.855	0.64	0.0069^b^
**DAE-9**	313	8910	0.396	0.40	0.0029^a^
**DAE-10**	335	21,300	0.557	0.27	0.0050^b^
**DAE-11**	345	5500	0.524	0.46	0.0044^b^
**DAE-12**	382	17,710	0.436	0.52	0.0007^b^
**DAE-13**	403	12,820	0.375	0.27	0.0002^b^

a **λ**_**isos**_, ***ε***_**closed**_ and quantum yields (**φ**) have been measured in solution (THF). ***X***_**A**_ has been determined irradiating an AML containing a homogenous 3:1 (w/w) PMMA/DAE mixture. Irradiation wavelength for quantum yield determination was at the isosbestic point for the ring closing quantum yield and at 595^a^ nm or 635^b^ nm, respectively.

Our previous studies established a correlation between Hammett and Hammett–Brown parameters and the excitation energy difference between a given DAE and a reference compound.^43,44^ The basis of this relationship is the spectroscopic Hammett equation (eq 1), where *R* is the gas constant, *T* is the temperature, ρ_A_ is the absorption constant, and *E*_T,R_ and *E*_T,H_ are excitation energies of the substituted molecule and its corresponding unsubstituted reference compound (see Supporting Information).^43,44^

1 $$\frac{E_{T,R}-E_{T,H}}{2.303\cdot R\cdot T}=\sigma \cdot \rho_{A}$$

This relationship enables a general prediction of absorption maxima for not-yet-synthesized DAEs. However, a significant limitation of this approach is the scarcity of available Hammett and Hammett–Brown parameters, particularly for many heteroaryl substituents with more complex structures. Herein, we report a modified approach for predicting the absorption maxima of novel DAEs using Hammett parameters calculated according to the methodology published by Ertl.^83^ The calculated **σ**_**m**_ and **σ**_**p**_ values were plotted against $\frac{E_{T,R}-E_{T,H}}{2.303\cdot R\cdot T}$ and then the data points were subjected to linear regression (Figure 4; see Supporting Information for methodological details). For this analysis, **DAE-13** was excluded as an outlier. We attribute its deviating behavior to the extended π-conjugated system, which enables delocalization over a substantially larger framework and thus renders it not directly comparable to DAEs bearing more simply substituted (hetero)­aryl moieties. Earlier reports regarding conjugation length proved that longer π-conjugated systems lead to bathochromically shifted absorption spectra,^84^ while a variation in conjugation length alters the photostability.^85^

**Figure 4:**
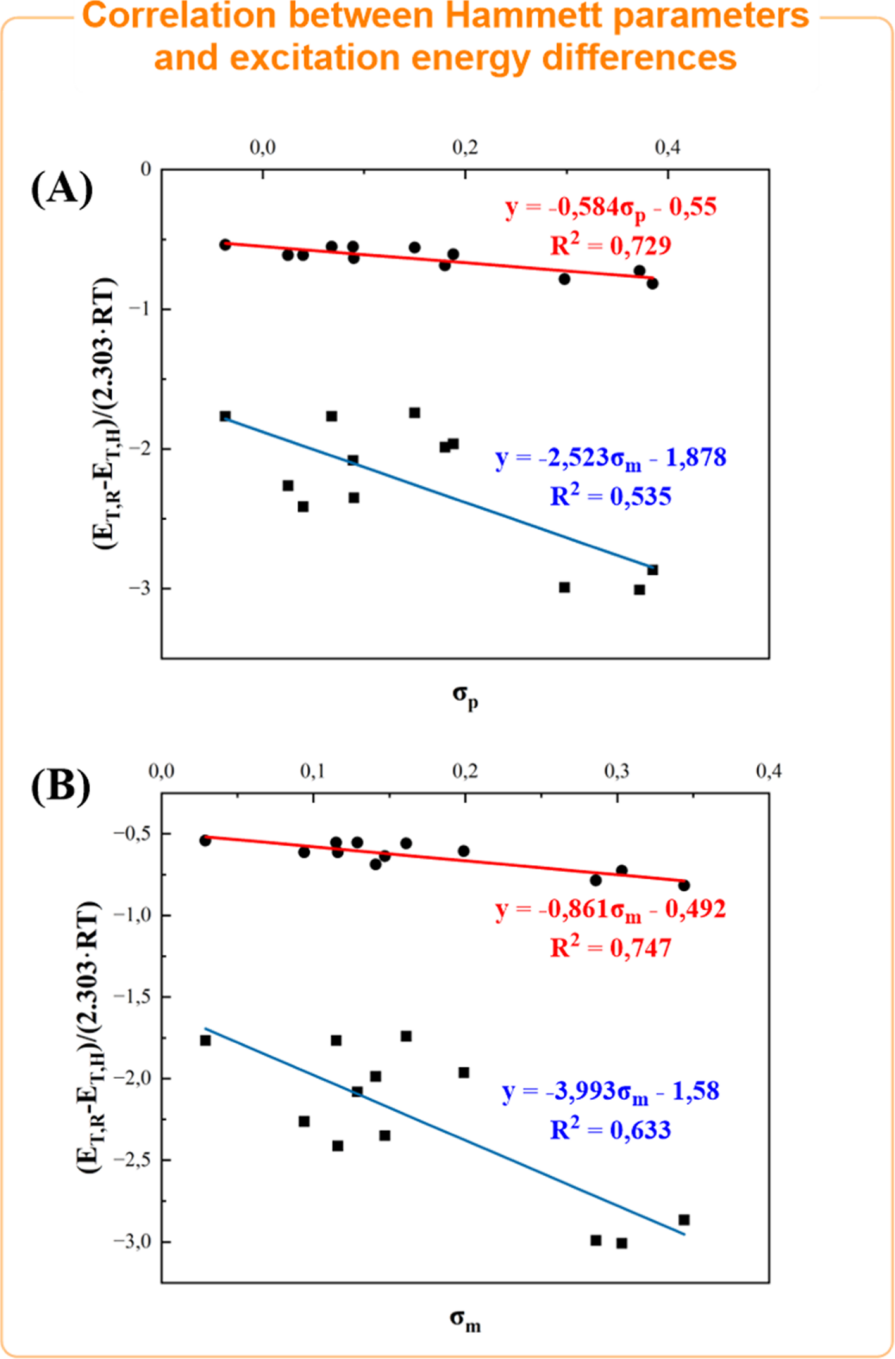
Plot of excitation energy difference against calculated Hammett parameters **σ**_**p**_ (A) and **σ**_**m**_ (B) (values found in Supporting Information). Data points based on the open form UV maximum are indicated by ■ and those based on the closed form visible maximum are indicated by ●.

When the UV absorption maxima of the open form are taken into account, only weak trends, if any, are discernible between the parameters σ_**m**_ and σ_**p**_ and the term $\frac{E_{T,R}-E_{T,H}}{2.303\cdot R\cdot T}$ , which is in well agreement to our previous findings.^43,44^ However, correlations are obtained for the visible absorption maximum of the closed form. The best fit (*R* ^2^ = 0.747) is observed when correlating the closed-form maximum with σ_**m**_. These findings allow to predict the location of the visible light maxima of the closed DAEs, but obviously not of those of the open forms. Nevertheless, on the basis of these values certain DAEs can be excluded from further investigations for various applications, e.g., because their maxima are too close together.

As mentioned in the introduction, suitable photochromes must consist of a bathochromically shifted UV absorbance, a high contrast of the closed form as well as a high fatigue resistance after prolonged irradiation. Those key features can be evaluated with the location of the isosbestic point, the molar extinction coefficient of the closed form visible light maximum as well as the fraction of active molecules (vide supra, Table 1). As shown in previous studies, irradiation of AMLs can be carried out at the isosbestic point rather than the UV maximum of the open form DAEs.^42,43^ All three parameters must be carefully adjusted, as they influence each other. For clarity, we have created a 3D scatter plot to visualize the suitability of our photochromes for application in AMLs when PMMA is used as the matrix (Figure 5). Recently, we were able to show that **DAE-1** and **DAE-10** are particularly well suited for nanoscopy due to their high fatigue resistance, relatively bathochromically shifted isosbestic points, and above-average molar extinction coefficients of the closed form.^41,42^ Now, we identify several additional photochromic dyes that exceed these benchmarks. **DAE-5**, **DAE-11**, **DAE-12** and **DAE-13** all exhibit higher **λ**_**isos**_ values than the reference compound **DAE-10**, while **DAE-5** is the only molecule with a higher ***ε***_**closed**_ than **DAE-10**.

**Figure 5:**
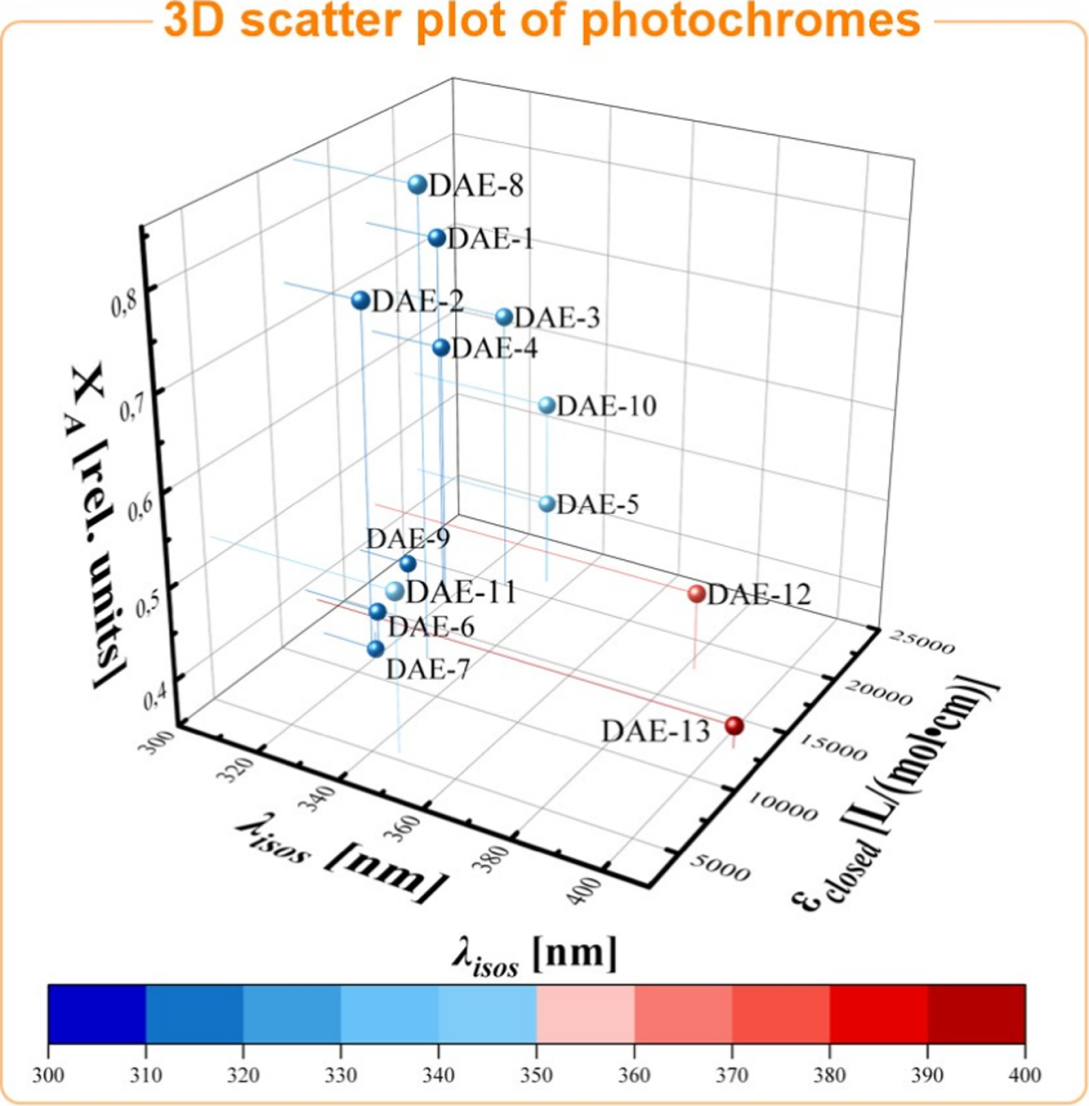
3D scatter plot of the spectroscopic parameters of various DAEs. Desirable values of the selected parameters are for **ε**_**closed**_ > 20.000 (L/mol·cm), **λ**_**isos**_ > 400 nm and ***X***_**A**_ ≈ 1.

With respect to stability, two distinct clusters of DAEs with comparable spectroscopic characteristics were identified. The first cluster comprises five photochromes (**DAE-1** to **DAE-4** and **DAE-8**), which exhibit markedly higher stability than the remaining compounds and can therefore be regarded as comparatively robust photoswitches. A prominent structural feature shared by these molecules is the presence of a *para*-alkoxy substituent on the (hetero)­aryl ring. **DAE-1**, **DAE-3**, and **DAE-4** contain a *para*-methoxy group, whereas **DAE-2** bears a *para*-trifluoromethoxy substituent. Notably, these groups differ substantially in their electronic properties: methoxy substituents are electron donating (Hammett constant **σ**_**p**_ = −0.27), while the trifluoromethoxy group is electron withdrawing (**σ**_**p**_ = 0.35).

Additional variation arises from the meta substitution pattern in **DAE-3** and **DAE-4**. The former carries two further methoxy groups, whereas **DAE-4** contains fluorine atoms in the 3- and 5-positions, which exert weak electron-withdrawing effects (**σ**_**m**_ = 0.34). A distinct electronic situation is observed for **DAE-8**, whose pyridazine ring renders the heteroaryl substituent significantly more electron deficient than anisole. Taken together, these observations indicate that simple electronic substituent effects alone cannot directly account for the enhanced stability of this group of photochromes.

The second cluster consists of photochromes with lower ***X***_**A**_ values, namely **DAE-6**, **DAE-7**, **DAE-9**, and **DAE-11**. The picoline derivatives **DAE-6** and **DAE-7** display isosbestic points (**λ**_**isos**_ = 319 and 314 nm, respectively) comparable to that of the unsubstituted 3-pyridyl analogue (**λ**_**isos**_ ≈ 315 nm).^86^ Due to the electron-deficient nature of these heteroaryl substituents, their reduced stability relative to **DAE-8** is unexpected. Notably, both cyclization and ring-opening quantum yields **φ** are also lower than those of the pyridazine-containing analogue. As anticipated, the formyl-substituted thienyl derivative **DAE-11** exhibits significantly lower stability than the corresponding methyl analogue **DAE-10**, consistent with earlier observations for structurally related DAEs.^43^

**DAE-9** represents the most striking case within this cluster, differing from **DAE-10** only by the absence of a methyl group. Despite this minor structural modification, **DAE-9** shows a substantially lower molar extinction coefficient of the closed form, reduced ***X***_**A**_, and a hypsochromically shifted **λ**_**isos**_. One possible explanation for the low stability is radical formation upon UV irradiation of the closed isomer, as proposed by Irie and co-workers.^87^ Literature reports indicate that α-positions of thiophenes are more susceptible to radical reactions than β-positions, which may account for the observed instability.^88–90^

**DAE-5** exhibits a stability comparable to this second cluster but displays a markedly higher ***ε***_**closed**_. This observation is unexpected, as strongly electron-withdrawing substituents such as the nitro group in **DAE-5** (σ = 0.78) are generally reported to decrease absorption intensity of the colored isomer in DAEs.^91^ Finally, comparison of **DAE-12** and **DAE-13** reveals a trend toward higher isosbestic wavelengths accompanied by reduced stability, suggesting that the molecular design of DAEs requires balancing bathochromic shifting against photochemical robustness.

To gain deeper insight into the origin of these differences, density functional theory (DFT) calculations were performed, explicitly considering the electronic environment of the polymer matrix.

### 2.3 Computational Analysis of Structural and Electronic Parameters

DFT calculations were carried out for **DAE-1**, **-8**, and **-13** in their open and closed forms using a polarizable continuum model with a dielectric constant corresponding to PMMA (see Supporting Information for computational details and parameters used). The frequency analysis shows that the open forms of the calculated DAEs are thermodynamically favorable against the closed form (Table S1). The calculated free energy changes Δ*G* for the electrocyclic ring-closing reaction reveal that **DAE-1** and **DAE-13** exhibit remarkably similar thermodynamic profiles, whereas the pyridazine derivative **DAE-8** shows a distinctly larger Δ*G*.

In both **DAE-1** and **DAE-13**, the highest occupied molecular orbital (HOMO) is symmetrically delocalized over both thiophene wings and the adjacent aromatic substituents and extends to the olefin in case of **DAE-13**. In contrast, the HOMO of **DAE-8** is almost exclusively localized on only one thiophene-pyridazine wing, while the second thiophene unit contributes negligibly to the orbital density (Figure 6).

**Figure 6:**
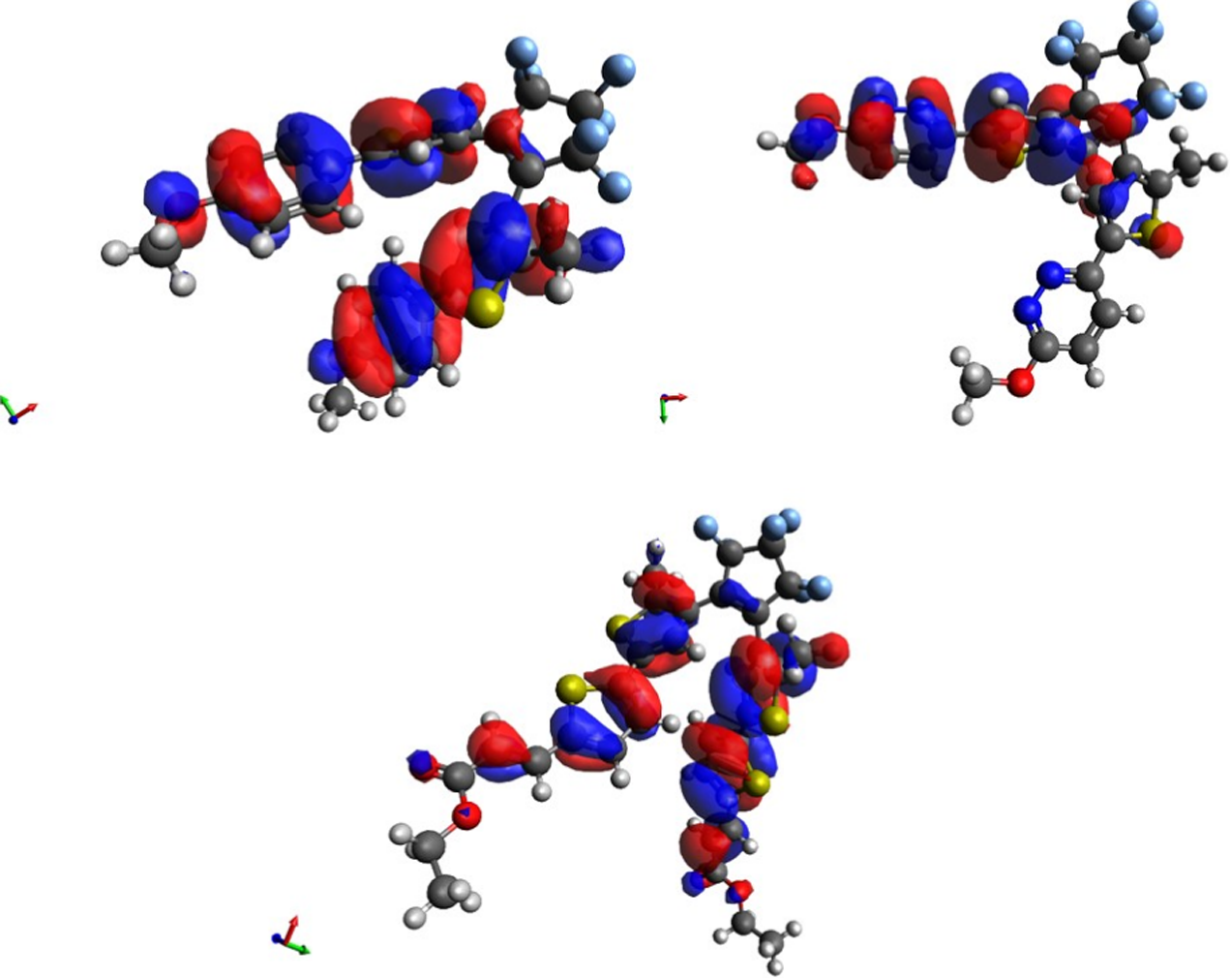
HOMO-profiles of open form **DAE-1** (left) and **DAE-8** (right) showing the large twisted thienyl-moieties and open form **DAE-13** (bottom).

This pronounced electric nonsymmetry indicates a partial decoupling of the two reactive fragments and suggests a reduced orbital coherence during the ring-closing process (LUMO-profiles shown in Supporting Information). This behavior is also reflected in the HOMO/LUMO gaps of the investigated DAEs (Table 2).

**Table 2:** HOMO/LUMO Gaps of the Open (**o**) and Closed (**c**) Forms, Calculated in PMMA Using B3LYP/Def2TZVP

DAE	HOMO [eV]	LUMO [eV]	Gap [eV]
**1o**	–5.603	–1.975	3.628
**1c**	–4.946	–2.683	2.263
**8o**	–6.032	–1.943	4.089
**8c**	–5.248	–3.017	2.231
**13o**	–5.786	–2.412	3.374
**13c**	–5.158	–3.307	1.851

In addition to electronic effects, pronounced geometric differences were identified. The pyridazine derivative **DAE-8** exhibits significantly larger torsional angles (129.0° and −47.0°) between the thiophene rings and the central perfluorocyclopentene ring compared to **DAE-1** (−122.4° and −147.7°) and **DAE-13** (−120.9° and −147.6°). The increased twisting further reduces π-overlap between the two thiophene units and reinforces the electronic decoupling observed in the frontier orbitals. Despite this, **DAE-8** displays the shortest distance between the two reactive α-carbon (C2–C2′) atoms in the open form (Table S3).

Changes in molecular surface area and volume upon ring closure were also analyzed using the Multiwfn software developed by Lu and Chen.^92^ While **DAE-1** and **DAE-13** show nearly identical overall changes (Table S5), **DAE-8** undergoes the largest relative variation. This suggests a higher degree of internal structural reorganization during switching, which is derived from the aforementioned torsion angles.

A particularly striking difference is observed when considering anisotropic dimensional changes (Figures S1–S6 & Table 3).

**Table 3:** Anisotropic Dimensional Changes in *x*-Axis

DAE	open form [Bohr]	closed form [Bohr]	Δ*x* [Bohr]	Δ*x* [Å]
**1**	37.5	47.25	9.75	5.159
**8**	37.5	47.25	9.75	5.159
**13**	43.5	56.00	12.50	6.614

The general space needed along the *x*-axis during the switching requires a comparable amount of space. For **DAE-1** and **DAE-8**, the absolute change is 21.6% in each case; for **DAE-13** it is 22.3%. Whereas **DAE-1** and **DAE-8** exhibit nearly identical changes (5.159 Å) along the molecular long axis upon ring closure, **DAE-13** requires a change of 6.615 Å with an approximately 22% relatively larger extension along this axis. This pronounced unidirectional expansion is expected to generate substantial mechanical stress within the polymer matrix during repeated switching cycles. We propose that this anisotropic volume demand represents a key factor contributing to the poor fatigue resistance of **DAE-13** in polymer films, despite its favorable spectroscopic properties in solution.

Furthermore, the LUMO of **DAE-13** (Figure 7) reveals a notable through–space interaction between the two β-carbon atoms of the thiophene moieties, in effect, a π-electronic short-circuit of the photochemical excitation orbital, whereas the double bond of the perfluorocyclopentene has no atomic orbital coefficients in the LUMO.

**Figure 7:**
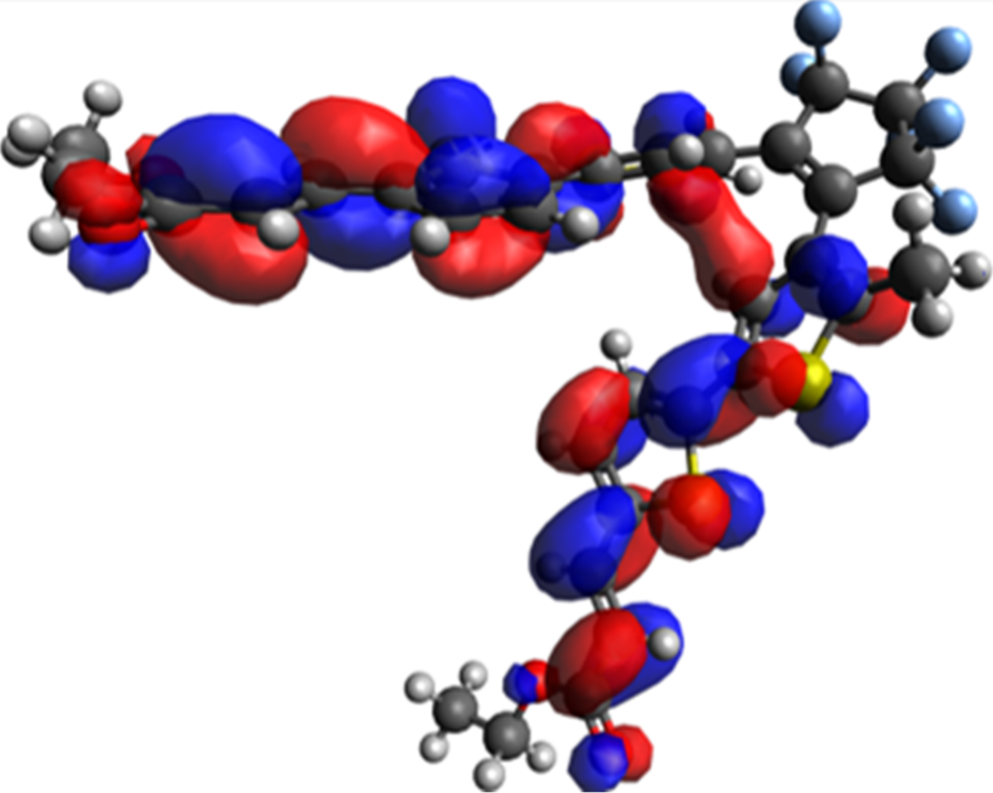
LUMO-profiles of **DAE-13** showing through space conjugation, π-separating the double bond from the other atomic orbitals.

This observation was unique for **DAE-13**. This behavior can be explained by the HOMO–LUMO-gaps as **DAE-13** has the lowest gap for the open (and closed) state in comparison with the other calculated DAEs (vide supra, Table 2).

### 2.4 Investigation of Matrix Effects on Fatigue Resistance of the Reference DAE

In addition to structural modifications of the photochrome, the polymer matrix provides an alternative means to enhance fatigue resistance. Owing to its favorable spectroscopic properties in solution, **DAE-1** was selected as a model compound for AML investigations (Figure 8A).

**Figure 8:**
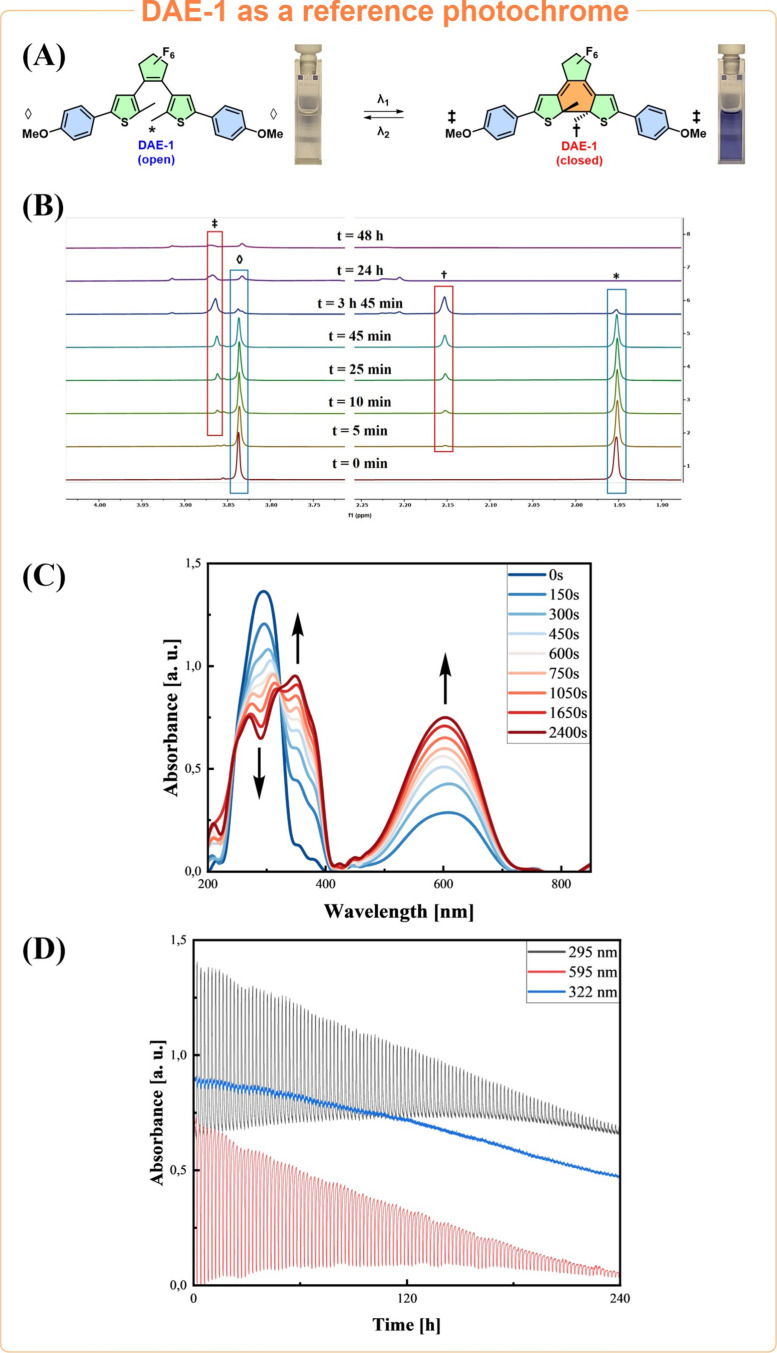
(A) Structure of open and closed form of reference **DAE-1**. Irradiation wavelength λ = 254 nm. (B) Irradiation of **DAE-1** over time and monitoring the methyl groups of both forms in the ^1^H NMR (CDCl_3_). Irradiation wavelength λ = 254 nm. (C) UV/vis spectra of irradiating **DAE-1** over time (THF). Irradiation wavelength λ = 322 nm. (D) Monitored absorbance of **DAE-1** of the isosbestic point as well as the UV and visible absorption maximum (THF). Irradiation wavelength λ = 322 nm.

Upon prolonged irradiation of a solution of **DAE-1** in CDCl_3_, ^1^H NMR spectroscopy reveals the gradual disappearance of the methyl resonances first corresponding to the open form and, upon extended exposure, also to the closed form which finally leads to the formation of yet unidentified decomposition products (Figure 8B). This observation is confirmed by photodegradation monitoring by tracking the absorbance at the isosbestic point as well as at the UV and visible absorption maxima in solution, either under continuous irradiation (*continuous mode*) or during alternating irradiation cycles (*switching mode*) (Figure 8C,D). Based on findings by Bianco et al.,^59^ which demonstrated comparable degradation behavior under continuous and switching conditions, stability measurements in the present study were performed in *continuous mode*, in contrast to the aforementioned stability measurements of the DAEs (compare Table 1). However, for applications in nanoscopy, undesirable decomposition phenomena in polymer matrices are more relevant and must be avoided. We therefore studied the corresponding behavior in matrices and found significant differences compared to the behavior in solution. A critical requirement for AML fabrication is the formation of a homogeneous film across the sapphire glass substrate. Spin coating is the most commonly employed technique for AML preparation. Typical solvents for polymer–DAE solutions include acetonitrile,^67^ THF,^67^ chloroform,^93^ and toluene.^94^ We^42,43^ and others^45^ previously demonstrated that anisole is particularly well suited for this purpose due to its good solubilizing properties and relatively high boiling point, which enables slower solvent evaporation during spin coating. In the present study, mainly anisole was used as well as 1,2-dichlorobenzene as a solvent for the highly nonpolar cyclic olefin polymer ZEONEX480R (vide infra).

A typical challenge when preparing AMLs this way is the occurrence of micro droplets that trap the photoswitch with the solvent (Figure 9A,B). To circumvent this phenomenon, we modified our procedure for AML fabrication by spin-coating a solution of the polymer first and after a small waiting time apply the polymer-DAE solution in the same way. The result is a homogeneous and droplet free AML (Figure 9C,D). In recent studies, we employed mainly PMMA/photochrome mixtures that were spin-coated onto a sapphire glass disk to produce the AMLs.^41–43^

**Figure 9:**
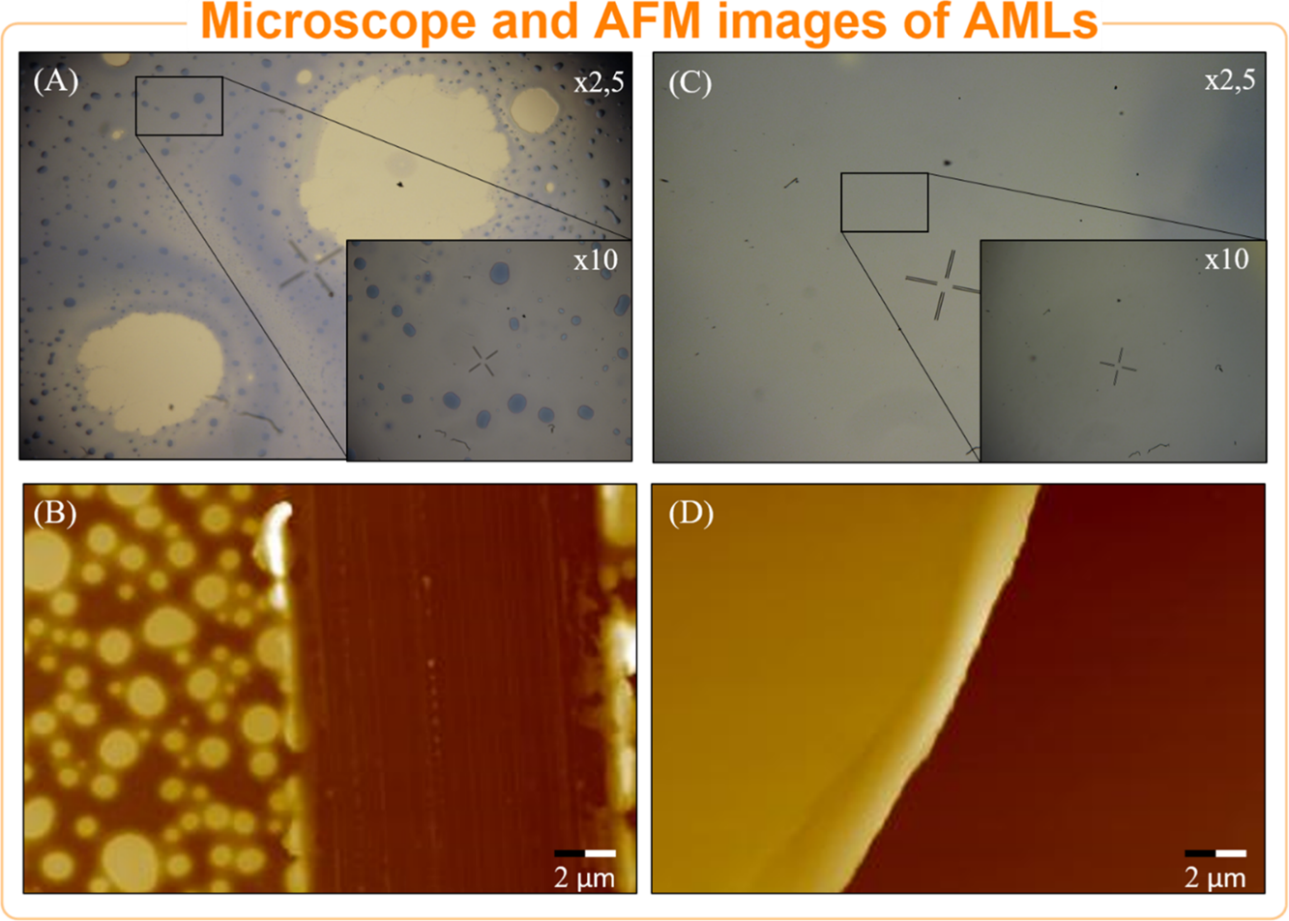
Example of vesicle formation. Left: microscope image (A) and AFM image (B) of AML containing **DAE-9** and ZEONEX480R using the typical procedure for the fabrication of AMLs. Right: microscope image (C) and AFM image (D) of AML containing **DAE-1** and ZEONEX480R using modified method of AML fabrication.

To investigate the correlation between glass transition temperature (***T***_**g**_) and degradation rate, we prepared a series of poly­(methacrylate-*co*-*n*-butyl acrylate) (PMMA/BA) copolymers with systematically varied ***T***_**g**_ values (Figure 10A).

**Figure 10:**
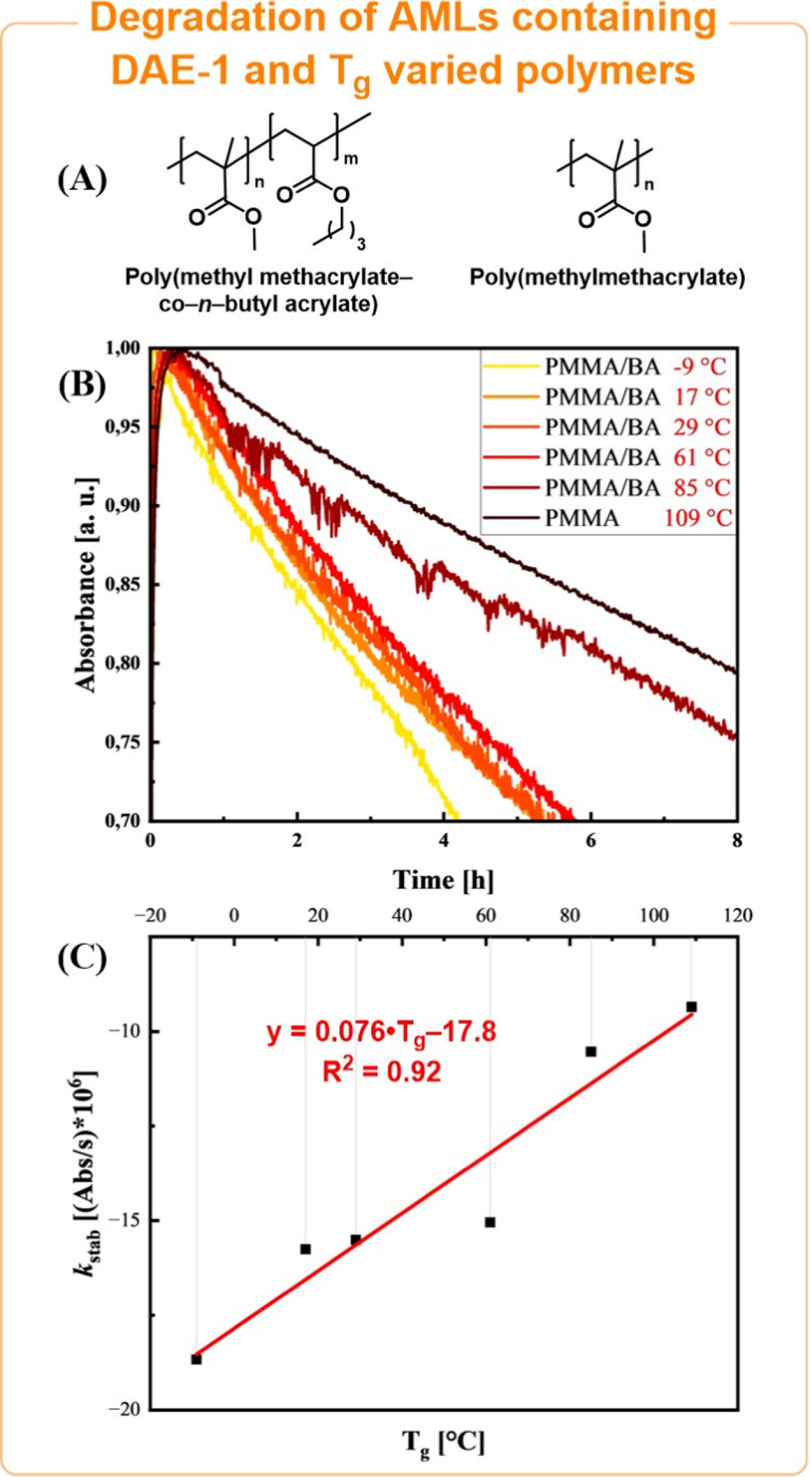
***T***_**g**_ variation experiment. (A) Structure of the used polymers (left: PMMA/BA; right: PMMA). (B) Degradation of AMLs containing **DAE-1** and the respective PMMA or PMMA/BA polymer measured in *continuous mode*. Numbers after the polymer indicate the ***T***_**g**_ values determined experimentally via DSC measurements. (C) Plot of degradation rate against the glas transition temperature ***T***_**g**_.

All irradiation experiments on AMLs based on PMMA or PMMA/BA initially exhibited an increase in visible-light absorbance, followed by a nearly linear decay over time (Figure 10B). In contrast to the determination of ***X***_**A**_ values, the measurements were performed in continuous mode, with constant irradiation at 318 nm and monitoring of the relative absorbance at the visible maximum of **DAE-1**. To quantitatively assess the observed behavior, we calculated the slope ***k***_**stab**_ beginning from the highest absorbance value, which was normalized to 1, until an absorbance of 0.7 was reached. Both parameters for stability can be assumed to be proportional to another based on previous reports in the literature, as switching mode and continuous mode led to the same degradation rate for comparable DAEs.^59^ The ***k***_**stab**_ values were then plotted against the corresponding ***T***_**g**_ values, yielding a satisfying linear correlation (*R* ^2^ = 0.92; Figure 10C). These results indicate a direct relationship between glass transition temperature and fatigue resistance for polymers closely related to PMMA. Accordingly, AMLs for applications related to the field of nanoscopy should use polymers with high ***T***_**g**_ values for higher fatigue resistance.

### 2.5 Investigation on the Influence of the Polymer Structure

In light of these findings, we evaluated a range of polymers as matrices using the model photochrome **DAE-1** (Table 4). The determination of the decay rate parameter ***k***_**stab**_ was carried out in the same way as for the ***T***_**g**_ variation experiment (vide supra).

**Table 4:**
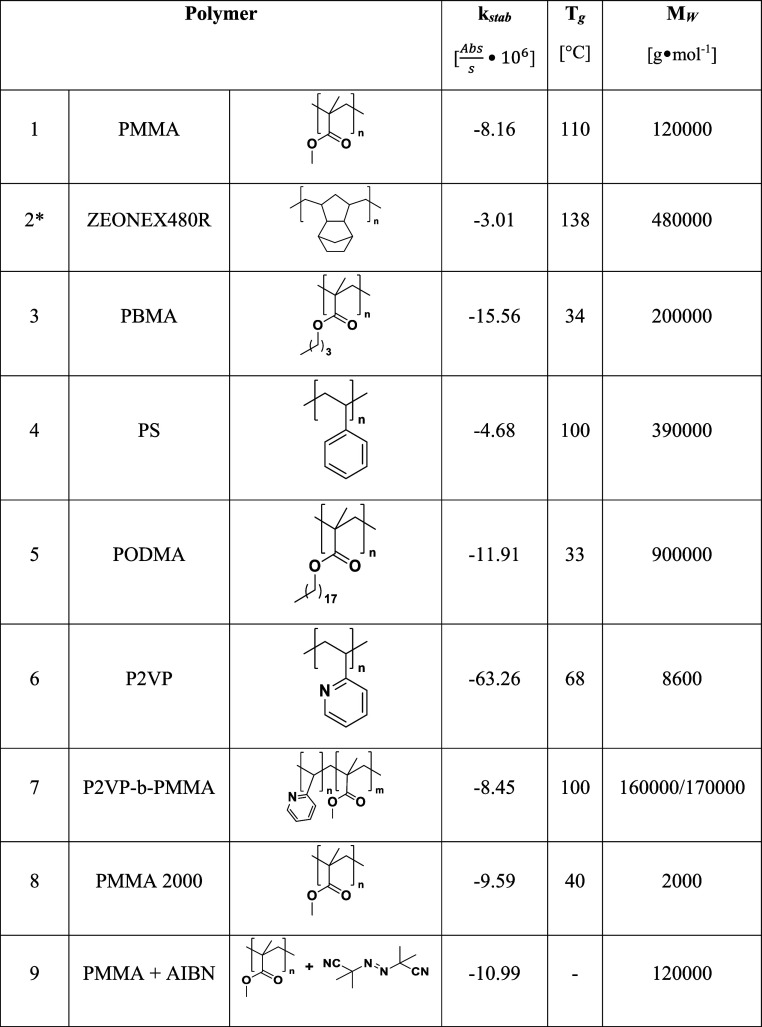
Stability Coefficients, Glass Transition Temperatures and Weight Average Molecular Weights of Polymers Used in Stability Measurements for **DAE-1** ^a^

a AMLs were spin coated using anisole (* sample spin coated using 1,2-dichlorobenzene as solvent).

All measurements started with an increase of the visible absorbance of the AML, which can be accounted to the formation of the closed form isomer, followed by steady decline over time due to fatigue (Figure 11). Changing the polymer matrix from PMMA to poly­(butyl methacrylate) (PBMA), poly­(octadecyl methacrylate) (PODMA) or PMMA with a lower average molecular weight (PMMA 2000) results in a faster degradation of the AML. This degradation is further accelerated when poly­(2-vinylpyridine) (P2VP) is employed. Only a slight decrease in fatigue resistance was observed when using a block copolymer composed of P2VP and PMMA (P2VP-*b*-PMMA), showing that PMMA is the superior polymer for nanoscopy applications in comparison to P2VP. To achieve improved performance, we subsequently evaluated a cyclic olefin polymer (ZEONEX480R); the high thermal stability and excellent transparency as well as its resistance toward thermooxidation makes it a suitable candidate for investigation toward optical applications.^95,96^ As anticipated we could achieve a 3-fold increase in stability of the AML with model photochrome **DAE-1** and ZEONEX480R (Table 4, **Entry 2**). Experiments carried out with polystyrene (PS) lead to a second polymer scaffold for increased photostability. Comparison of structural features of ZEONEX480R and PS indicate a correlation between lower polarity and increased stability.

**Figure 11:**
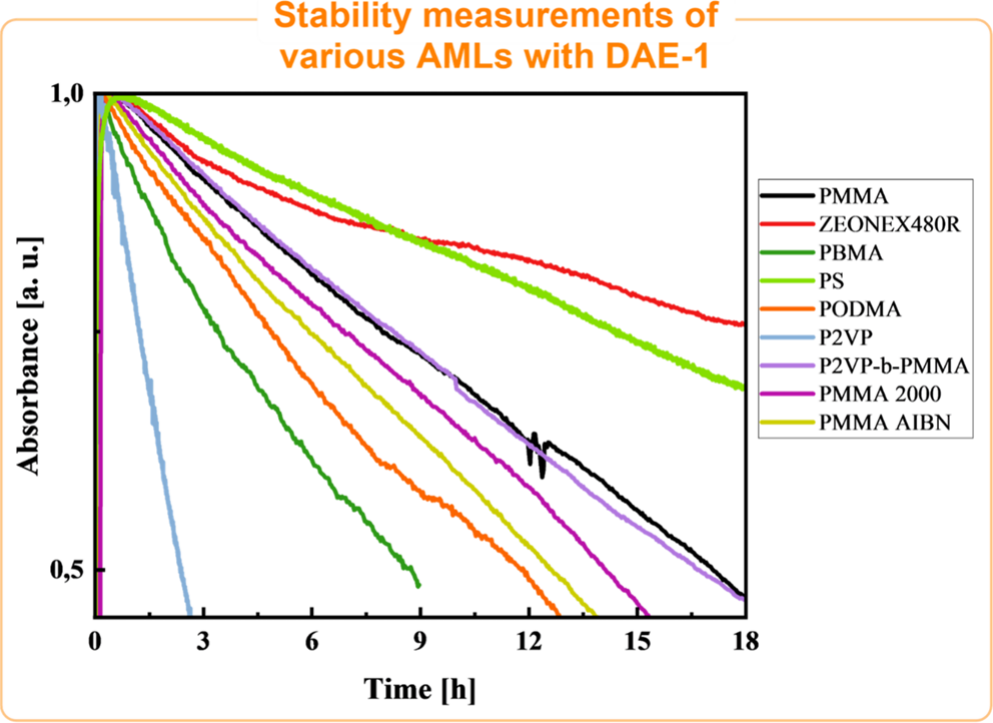
Stability measurements of **DAE-1** in different matrices. The AMLs have been spin-coated using a 3:1 (w/w) ratio of polymer to DAE in anisole or dichlorobenzene. The open form of the samples has been irradiated continuously with light of 318 nm.

If a radical pathway contributes to the degradation of the photochromes, we investigated whether the addition of the radical initiator azobis­(isobutyronitrile) (AIBN) led to a pronounced acceleration of the degradation process. The decreased fatigue resistance of this AML could hint the formation of radicals, which lead to a faster degradation.

Next, we evaluated a possible correlation between ***T***_**g**_ and the degradation rate similar to the experiments we had done with PMMA and PMMA/BA (vide supra). No correlation was observed when taking all AMLs into account. When only plotting the data of the polymers similar in molecular weight (Table 4, **entries 1** through **5** and **entry 7**) or structurally the same with a varied molecular weight (**entry 8**), a correlation between ***T***_**g**_ and **k**_**stab**_ is observed (*R* ^2^ = 0.964) (Figure 12). Taking the data provided by ***T***_**g**_ variation plot (Figure 10) into account, polymers with a high ***T***_**g**_ (>100 °C), an average molecular mass of 100,000 to 300,000 as well as a high thermal stability and resistance to thermooxidation like the cyclic olefin polymer ZEONEX480R presented in this study, are suitable systems for applications in nanoscopy.

**Figure 12:**
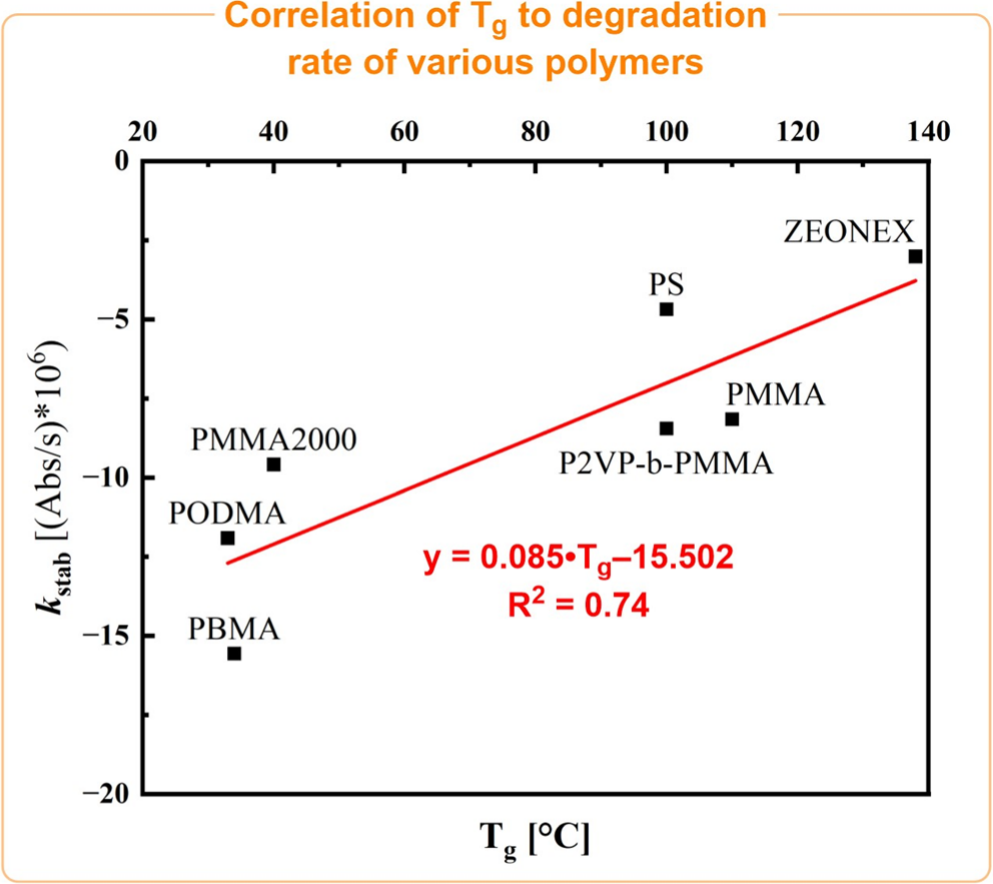
Correlation of the glass transition temperature of selected polymers in comparison to the degradation rate with **DAE-1**. All values can be found in Table 2.

### 2.6 Photochrome Variation for Stability Measurements

To assess the general applicability of our findings, we prepared AMLs incorporating different DAEs with three polymer matrices: PMMA as a reference, the structurally related PBMA with a lower ***T***_**g**_ and accelerated degradation for **DAE-1**, and the cyclic olefin polymer ZEONEX480R, which exhibits a higher ***T***_**g**_ and enhanced fatigue resistance. Based on this framework, we selected photochromes with comparatively low (**DAE-9**) and high (**DAE-8**) intrinsic fatigue resistance. All irradiation experiments were performed in *continuous mode*.

For AMLs incorporating **DAE-8** (Figure 13A), degradation rates were largely similar across the investigated polymers. The most striking observation was the increased degradation rate observed for AMLs containing ZEONEX480R. We attribute this deviation to the unique pyridazine moiety of **DAE-8**, which may enable distinct N-to-S interactions with the adjacent sulfur atom of the thiophene ring and participate in hydrogen bonding as an effective proton acceptor.^97,98^ These interactions could lead to a different molecular environment in more polar polymers, such as PMMA and PBMA, compared to the nonpolar ZEONEX480R matrix. This hypothesis is further supported by the higher dipole moment of the pyridazine ring (4.22 D)^97^ relative to the thiophene substituent (0.51 D)^99^ in **DAE-9** and the anisole substituent (1.26 D)^100^ in **DAE-1**.

**Figure 13:**
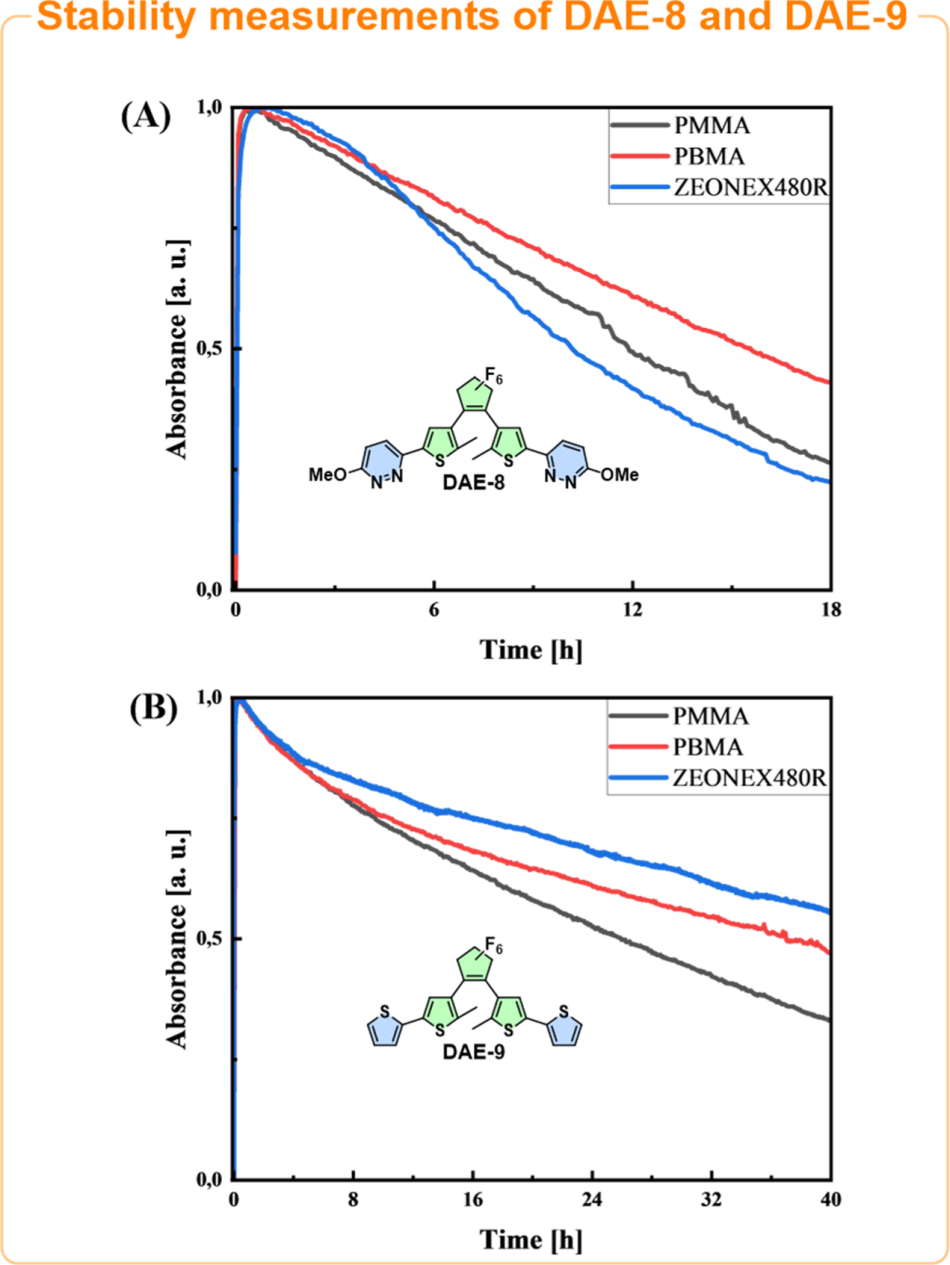
Stability measurements of **DAE-8** (A) and **DAE-9** (B) in PMMA, ZEONEX480R and PBMA. The AMLs have been spin-coated using a 3:1 (w/w) ratio of polymer to DAE in anisole or dichlorobenzene. The samples have been irradiated continuously with light of 318 nm. **DAE-9** was subjected to our modified spin-coating protocol (vide supra).

The degradation behavior of AMLs containing **DAE-9** (Figure 13B) closely resembled that of the corresponding **DAE-1** samples. Notably, PBMA led to a slight increase in fatigue resistance in this case, in contrast to its accelerating effect on the degradation of AMLs containing **DAE-1**.

An additional noteworthy result is the reversal of the relative stability of AMLs containing **DAE-8** and **DAE-9** under continuous irradiation (compare Table 1). This finding contrasts with previous reports by Bianco and co-workers, who observed no difference in degradation behavior between continuous irradiation and repeated switching.^59^

## 3 Conclusion

In summary, we synthesized a series of known and previously unreported photochromic diarylethenes and evaluated their spectroscopic properties. Several derivatives displayed enhanced characteristics, rendering them promising candidates for nanoscopy applications. We further identified a correlation between calculated Hammett parameters of the aryl substituents and the excitation energy differences relative to a reference dye. This allows to predict the maxima of the two isomers of nonyet synthesized DAEs to effectively evaluate their suitability. DFT calculations carried out for selected molecules revealed varying electronic natures without correlation to spectroscopic behavior. In addition, we investigated the influence of polymer matrices on a model system and achieved a 3-fold increase in photostability by replacing PMMA with the cyclic olefin polymer ZEONEX480R. Our screening also demonstrated that polymers with higher glass transition temperatures provide increased fatigue resistance, a trend confirmed with excellent correlation for PMMA/BA copolymers and with reasonable agreement for structurally related polymers of comparable molecular weight.

## 4 Materials and Methods

### 4.1 Synthesis and Characterization of Photochromes

All reactions were carried out under an atmosphere of nitrogen in oven-dried glassware. Unless otherwise mentioned, all chemicals were purchased and used without further purification. Anhydrous solvents were dried according to standard procedures before usage. *n*-Butyllithium was not titrated prior to its use. The ATR-IR spectra were obtained on a Bruker Alpha in the range of 400 to 4000 cm^–1^. ^1^H NMR spectra were recorded at 400 MHz. ^13^C NMR spectra were recorded at 100 MHz, with the solvent peak used as the internal ref 101. Multiplicities are described by using the following abbreviations: s = singlet, d = doublet, t = triplet, q = quartet, and m = multiplet. Signal orientations in DEPT experiments were described as follows: o = no signal; + = up (CH, CH_3_); – = down (CH_2_). The electrospray ionization mass spectra (ESIMS) were measured with a Bruker Impact-II mass spectrometer. Samples were sprayed from MeCN. Chromatography: The reactions were traced by thin layer chromatography with silica gel 60 (F254, MERCK KGAA). For the detection of substances, quenching was used at either 254 or 366 nm with a mercury lamp. The preparative column chromatography was conducted through silica gel 60 (230–400 mesh).

### 4.2 Preparation of Photochromic Films

For the photostability measurements we used sapphire substrates (10.0 mm θ, 1 mm thickness, SaphTec). For building up a matrix we used various polymers (specification and seller details are shown in the Supporting Information). For producing the coatings, a spin-coater (LAURELL WS-650 MZ-23MPP) was used. The produced coatings were investigated by using optical microscopy (ZEISS Axioplan) and atomic force microscopy (Park Systems NX-12).

### 4.3 Determination of Spectroscopic Parameters

The UV–vis spectra were recorded with an CCD UV–vis–NIR spectrophotometer (type Exemplar LS from BWTek, USA), and the reviewed wavelengths were in the range of 250 and 850 nm. The used quartz cuvettes had a path length of 1 cm. The irradiation in the UV-range was carried out using a Xe-short-arc lamp (300 W, Quantum Design, Germany) in combination with a custom Czerny-Turner-monochromator for wavelength selection. In the visible range a set of LEDs with suitable wavelengths for the DAEs were used (3 W, Avonec, Germany). A 3D-printed cell holder allows to irradiate the solutions while simultaneously acquiring spectra. To determine the quantum yields, the CCD UV–vis–NIR spectrophotometer mentioned above was used. As solvents, THF (99.9%, anhydrous, inhibitor-free) and cyclohexane (99.9%, spectrometry grade) were used.

### 4.4 Stability Measurements of Photochromes

To determine the photochrome’s stabilities in films via switching mode, we used a setup as shown in the Supporting Information (Figure S7). We applied a red laser with a wavelength of 638 nm (Hübner Photonics, Cobolt 06-mld 638 nm, max. power 180 mW) and a UV laser with a wavelength of 325 nm (Kimmon Koha, IK 3083 R-D). To control the intensity of the red laser, we employed a rotation stage (Thorlabs, K10CR1/M) with a half wave plate and a following polarizer. An acousto-optic modulator (Opto Electronic, AA.MQ110-A3-UV) was used to regulate the light power of the UV laser. A photosensor (Hamamatsu Photonics, H10722-20 with 700 mV gain) was employed as detector, with an edge filter positioned in front to block UV light.

The stabilities under continuous irradiation in films were measured using an UV–vis–NIR spectrophotometer (type UV-670 JASCO GMBH, Germany) equipped with a 3D-printed film holder containing a servo-motor that enables the rotation of the film between the irradiation position and the measurement position to enable an equivalence of the light paths. The switching procedure was carried out using the light of a fluorescence spectrometer (FP-8500, JASCO) that was guided via a liquid light guide (250-series, Lumatec) into the UV–vis-spectrometer. For the irradiation a half-width of 20 nm was selected for the light source, which yielded a power of 2–3 mW/cm^2^, depending on the selected wavelengths.

## Supplementary Material



## Notes

General experimental methods; ^1^H NMR, ^13^C NMR, and ^19^F NMR data; DFT calculations; UV spectra; additional information for polymers used (PDF)

## References

[1] Irie M., Fukaminato T., Matsuda K., Kobatake S. (2014). Photochromism of Diarylethene
Molecules and Crystals: Memories, Switches, and Actuators. Chem. Rev..

[2] Exelby R., Grinter R. (1965). Phototropy (or Photochromism). Chem. Rev..

[3] Lutsyk P., Sworakowski J., Janus K., Nešpůrek S., Kochalska A. (2010). Photochromic
Systems as Models for Opto-Electrical
Switches. Mol. Cryst. Liq. Cryst..

[4] Dürr, H. ; Bouas-Laurent, H. Photochromism: Molecules and Systems, Rev.; Elsevier: Amsterdam Boston, 2003.

[5] Kobatake S., Irie M. (2004). Single-Crystalline
Photochromism of Diarylethenes. Bull. Chem.
Soc. Jpn..

[6] Nakamura S., Yokojima S., Uchida K., Tsujioka T., Goldberg A., Murakami A., Shinoda K., Mikami M., Kobayashi T., Kobatake S., Matsuda K., Irie M. (2008). Theoretical Investigation
on Photochromic Diarylethene: A Short Review. J. Photochem. Photobiol. Chem..

[7] Merino E., Ribagorda M. (2012). Control over
Molecular Motion Using the *Cis* – *Trans* Photoisomerization of the Azo Group. Beilstein
J. Org. Chem..

[8] Kunz A., Oberhof N., Scherz F., Martins L., Dreuw A., Wegner H. A. (2022). Azobenzene-Substituted
Triptycenes: Understanding the
Exciton Coupling of Molecular Switches in Close Proximity. Chem.—Eur. J..

[9] Fischer M. H., Zgheib A., El Hraoui I., Schnickmann A., Schirmer T., Jeschke G., Schmidt A. (2025). Inverse Punicines:
Isomers of Punicine and Their Application in LiAlO2, Melilite and
CaSiO3 Separation. Separations.

[10] Schmidt A., Mordhorst T., Nieger M. (2005). Investigation of a
Betainic Alkaloid
from Punica Granatum. Nat. Prod. Res..

[11] Schmidt A., Mordhorst T. (2004). Conjugated,
Cross-Conjugated, and Pseudo-Cross-Conjugated
Derivatives of a Pyridinium Alkaloid from Punica Granatum. Arkivoc.

[12] Otto C. F., Herzberger C., Liu M., Namyslo J. C., Nieger M., Freese T., Lederle F., Hübner E. G., Schmidt A. (2020). Borane Adducts of Punicine and of Its Dehydroxy Derivatives
(Pyridinium-1-Yl)-2- and 3-Phenolates. Tetrahedron.

[13] Xia H., Xie K., Zou G. (2017). Advances in
Spiropyrans/Spirooxazines and Applications
Based on Fluorescence Resonance Energy Transfer (FRET) with Fluorescent
Materials. Molecules.

[14] Berkovic G., Krongauz V., Weiss V. (2000). Spiropyrans and Spirooxazines
for
Memories and Switches. Chem. Rev..

[15] Patel D. G., Benedict J. B., Kopelman R. A., Frank N. L. (2005). Photochromism of
a Spirooxazine in the Single Crystalline Phase. Chem. Commun..

[16] Heller H. G., Oliver S. (1981). Photochromic Heterocyclic
Fulgides. Part 1. Rearrangement
Reactions of (E)-α-3-Furylethylidene­(Isopropylidene)­Succinic
Anhydride. J. Chem. Soc. Perkin.

[17] Irie, M. Photo-Reactive Materials for Ultrahigh Density Optical Memory: MITI Research and Development Program on Basic Technologies for Future Industries; Elsevier, 1994.

[18] Takami S., Kobatake S., Kawai T., Irie M. (2003). Extraordinarily High
Thermal Stability of the Closed-Ring Isomer of 1,2-Bis­(5-Methyl-2-Phenylthiazol-4-Yl)­Perfluorocyclopentene. Chem. Lett..

[19] Jean-Ruel H., Cooney R. R., Gao M., Lu C., Kochman M. A., Morrison C. A., Miller R. J. D. (2011). Femtosecond Dynamics of the Ring
Closing Process of Diarylethene: A Case Study of Electrocyclic Reactions
in Photochromic Single Crystals. J. Phys. Chem.
A.

[20] Hanazawa M., Sumiya R., Horikawa Y., Irie M. (1992). Thermally irreversible
photochromic systems. Reversible photocyclization of 1,2-bis (2-methylbenzo­[b]­thiophen-3-yl)­perfluorocyclocoalkene
derivatives. J. Chem. Soc., Chem. Commun..

[21] Kolarski D., Steinbach P., Bannwarth C., Klaue K., Hecht S. (2024). Diaryltriazolium
Photoswitch: Reaching a Millisecond Cycloreversion with High Stability
and NIR Absorption. Angew. Chem., Int. Ed..

[22] Irie, M. , Ed. Photo-reactive materials for ultrahigh density optical memory : MITI research and development program on basic technologies for future industries; Elsevier Science Ltd: Amsterdam, 1994.

[23] Irie M. (2000). Diarylethenes
for Memories and Switches. Chem. Rev..

[24] Yao J. N., Hashimoto K., Fujishima A. (1992). Photochromism Induced in an Electrolytically
Pretreated Mo03 Thin Film by Visible Light. Nature.

[25] Zhang H., Qi Y., Zhao X., Li M., Wang R., Cheng H., Li Z., Guo H., Li Z. (2022). Dithienylethene-Bridged Fluoroquinolone
Derivatives for Imaging-Guided Reversible Control of Antibacterial
Activity. J. Org. Chem..

[26] Guo H., Dai J., Deng L., Zhang Z., Tian H., Zhang J. (2025). Photopharmacology
beyond Azobenzene Photoswitches. Responsive
Mater..

[27] Zhang P., Wang G., Yu H. (2024). Ultraviolet-Visible-near-Infrared
Light-Responsive Soft Materials: Fabrication, Photomechanical Deformation
and Applications. Responsive Mater..

[28] Lahikainen M., Kuntze K., Zeng H., Helantera S., Hecht S., Priimagi A. (2020). Tunable Photomechanics in Diarylethene-Driven
Liquid Crystal Network Actuators. ACS Appl.
Mater. Interfaces.

[29] Morimoto M., Irie M. (2010). A Diarylethene Cocrystal That Converts Light into Mechanical Work. J. Am. Chem. Soc..

[30] Terao F., Morimoto M., Irie M. (2012). Light-Driven Molecular-Crystal Actuators:
Rapid and Reversible Bending of Rodlike Mixed Crystals of Diarylethene
Derivatives. Angew. Chem., Int. Ed..

[31] Dong X., Tong F., Hanson K. M., Al-Kaysi R. O., Kitagawa D., Kobatake S., Bardeen C. J. (2019). Hybrid
Organic–Inorganic Photon-Powered
Actuators Based on Aligned Diarylethene Nanocrystals. Chem. Mater..

[32] Logtenberg H., Browne W. R. (2013). Electrochemistry of Dithienylethenes
and Their Application
in Electropolymer Modified Photo- and Redox Switchable Surfaces. Org. Biomol. Chem..

[33] van
der Molen S. J., Liao J., Kudernac T., Agustsson J. S., Bernard L., Calame M., van Wees B. J., Feringa B. L., Schönenberger C. (2009). Light-Controlled Conductance Switching of Ordered Metal–Molecule–Metal
Devices. Nano Lett..

[34] Whalley A. C., Steigerwald M. L., Guo X., Nuckolls C. (2007). Reversible Switching
in Molecular Electronic Devices. J. Am. Chem.
Soc..

[35] Kronemeijer A. J., Akkerman H. B., Kudernac T., van Wees B. J., Feringa B. L., Blom P. W. M., de Boer B. (2008). Reversible Conductance Switching
in Molecular Devices. Adv. Mater..

[36] Murzyn K., van der Geest M. L. S., Guery L., Nie Z., van Essen P., Witte S., Kraus P. M. (2024). Breaking Abbe’s Diffraction
Limit with Harmonic Deactivation Microscopy. Sci. Adv..

[37] Abbe E. (1873). Beiträge
zur Theorie des Mikroskops und der mikroskopischen Wahrnehmung. Arch. Mikrosk. Anat. Entwicklungsmech..

[38] Hell S. W. (2015). Nanoscopy
with Focused Light (Nobel Lecture). Angew. Chem.,
Int. Ed..

[39] Hell S. W. (2004). Strategy
for Far-Field Optical Imaging and Writing without Diffraction Limit. Phys. Lett. A.

[40] Kim D., Aktalay A., Jensen N., Uno K., Bossi M. L., Belov V. N., Hell S. W. (2022). Supramolecular Complex
of Photochromic
Diarylethene and Cucurbit[7]­Uril: Fluorescent Photoswitching System
for Biolabeling and Imaging. J. Am. Chem. Soc..

[41] Jain P., Geisler C., Leitz D., Udachin V., Nagorny S., Weingartz T., Adams J., Schmidt A., Rembe C., Egner A. (2023). Super-Resolution
Reflection Microscopy via Absorbance Modulation. ACS Nanosci. Au.

[42] Nagorny S., Schewe M., Weingartz T., Eitzeroth A., Adams J., Rembe C., Schmidt A. (2024). Stabilities
of Bis­(Thienyl)­Ethenes
in Polymethyl Methacrylate (PMMA) Coatings as Absorbance Modulation
Layers for Nanoscale Imaging. Mater. Adv..

[43] Weingartz T., Nagorny S., Adams J., Eitzeroth A., Schewe M., Rembe C., Schmidt A. (2023). Bis­(Thienyl)­Ethenes
with α-Methoxymethyl Groups. Syntheses, Spectroscopic Hammett
Plots, and Stabilities in PMMA Films. RSC Adv..

[44] Nagorny S., Weingartz T., Namyslo J. C., Adams J., Schmidt A. (2023). Correlation
between Absorption and Substitution of Photochromic 1,2-Bis­(Thienyl)­Ethenes
(BTEs) Using Modified Spectroscopic Hammett Equations. Eur. J. Org. Chem..

[45] Majumder A., Wan X., Masid F., Pollock B. J., Andrew T. L., Soppera O., Menon R. (2016). Reverse-Absorbance-Modulation-Optical Lithography for Optical Nanopatterning
at Low Light Levels. AIP Adv..

[46] Nevskyi O., Sysoiev D., Dreier J., Stein S. C., Oppermann A., Lemken F., Janke T., Enderlein J., Testa I., Huhn T., Wöll D. (2018). Fluorescent
Diarylethene Photoswitches—A Universal Tool for Super-Resolution
Microscopy in Nanostructured Materials. Small.

[47] Nevskyi O., Sysoiev D., Oppermann A., Huhn T., Wöll D. (2016). Nanoscopic
Visualization of Soft Matter Using Fluorescent Diarylethene Photoswitches. Angew. Chem., Int. Ed..

[48] Roubinet B., Weber M., Shojaei H., Bates M., Bossi M. L., Belov V. N., Irie M., Hell S. W. (2017). Fluorescent Photoswitchable
Diarylethenes for Biolabeling and Single-Molecule Localization Microscopies
with Optical Superresolution. J. Am. Chem. Soc..

[49] Arai Y., Ito S., Fujita H., Yoneda Y., Kaji T., Takei S., Kashihara R., Morimoto M., Irie M., Miyasaka H. (2017). One-Colour
Control of Activation, Excitation and Deactivation of a Fluorescent
Diarylethene Derivative in Super-Resolution Microscopy. Chem. Commun..

[50] Wäldchen S., Lehmann J., Klein T., van de Linde S., Sauer M. (2015). Light-Induced Cell Damage in Live-Cell Super-Resolution Microscopy. Sci. Rep..

[51] Herder M., Schmidt B. M., Grubert L., Pätzel M., Schwarz J., Hecht S. (2015). Improving the Fatigue
Resistance
of Diarylethene Switches. J. Am. Chem. Soc..

[52] Irie M., Lifka T., Uchida K., Kobatake S., Shindo Y. (1999). Fatigue Resistant
Properties of Photochromic Dithienylethenes: By-Product Formation. Chem. Commun..

[53] Barrez E., Laurent G., Pavageau C., Sliwa M., Métivier R. (2018). Comparative
Photophysical Investigation of Doubly-Emissive Photochromic-Fluorescent
Diarylethenes. Phys. Chem. Chem. Phys..

[54] Roubinet B., Bossi M. L., Alt P., Leutenegger M., Shojaei H., Schnorrenberg S., Nizamov S., Irie M., Belov V. N., Hell S. W. (2016). Carboxylated
Photoswitchable Diarylethenes
for Biolabeling and Super-Resolution RESOLFT Microscopy. Angew. Chem., Int. Ed..

[55] Ichikawa T., Morimoto M., Sotome H., Ito S., Miyasaka H., Irie M. (2016). Photochromism of Diarylethene Derivatives Having Benzophosphole and
Benzothiophene Groups. Dyes Pigm..

[56] Uno K., Nagorny S., Aktalay A., Kim D., Bossi M. L., Belov V. N., Hell S. W. (2025). Fluorescent Diarylethenes
With Polar
Groups: Synthesis, Spectra, and Optical Microscopy Applications. Chem.—Eur. J..

[57] Kobatake S., Uchida K., Tsuchida E., Irie M. (2002). Single-Crystalline
Photochromism of Diarylethenes: Reactivity–Structure Relationship. Chem. Commun..

[58] Ramamurthy V., Venkatesan K. (1987). Photochemical
Reactions of Organic Crystals. Chem. Rev..

[59] Pariani G., Quintavalla M., Colella L., Oggioni L., Castagna R., Ortica F., Bertarelli C., Bianco A. (2017). New Insight into the
Fatigue Resistance of Photochromic 1,2-Diarylethenes. J. Phys. Chem. C.

[60] Lvov A. G., Khusniyarov M. M., Shirinian V. Z. (2018). Azole-Based
Diarylethenes as the
next Step towards Advanced Photochromic Materials. J. Photochem. Photobiol. C Photochem. Rev..

[61] Hu T., Li Z., Wang T., Zeng H. (2016). Synthesis and Properties of Photochromic
Diarylethenes Bearing Thiazole Moiety. J. Heterocycl.
Chem..

[62] Fukumoto S., Nakashima T., Kawai T. (2011). Photon-Quantitative Reaction of a
Dithiazolylarylene in Solution. Angew. Chem.,
Int. Ed..

[63] Herder M., Utecht M., Manicke N., Grubert L., Pätzel M., Saalfrank P., Hecht S. (2013). Switching with Orthogonal Stimuli:
Electrochemical Ring-Closure and Photochemical Ring-Opening of Bis­(Thiazolyl)­Maleimides. Chem. Sci..

[64] Nickel F., Bernien M., Herder M., Wrzalek S., Chittas P., Kraffert K., Arruda L. M., Kipgen L., Krüger D., Hecht S., Kuch W. (2017). Light-Induced Photoisomerization
of a Diarylethene Molecular Switch on Solid Surfaces. J. Phys.: Condens. Matter.

[65] Qiu H., Herder M., Hecht S., Samorì P. (2021). Ternary-Responsive
Field-Effect Transistors and Multilevel Memories Based on Asymmetrically
Functionalized Janus Few-Layer WSe2. Adv. Funct.
Mater..

[66] Qiu H., Liu Z., Yao Y., Herder M., Hecht S., Samorì P. (2020). Simultaneous
Optical Tuning of Hole and Electron Transport in Ambipolar WSe2 Interfaced
with a Bicomponent Photochromic Layer: From High-Mobility Transistors
to Flexible Multilevel Memories. Adv. Mater..

[67] Lazareva S. K., Glebov E. M., Nevostruev D. A., Lonshakov D. V., Lvov A. G., Shirinian V. Z., Zinovyev V. A., Smolentsev A. B. (2019). Fluorescence
Modulation of Eosin Y in a PMMA Film by Diarylethene Switching. Mendeleev Commun..

[68] Guo H., Zhang F., Wu G., Sun F., Pu S., Mai X., Qi G. (2003). Multi-Wavelength Optical Storage of Diarylethene PMMA
Film. Opt. Mater..

[69] Luo S., Chen K., Cao L., Liu G., He Q., Jin G., Zeng D., Chen Y. (2005). Photochromic
Diarylethene for Rewritable
Holographic Data Storage. Opt. Express.

[70] Li R., Ou T., Wen L., Yan Y., Li W., Qin X., Wang S. (2024). All-Visible-Light-Activated
Diarylethene Photoswitches. Molecules.

[71] Wang Y., Tang Y., Zheng Y., Feng Y., Wang P., Zhang L., Li Z., Guo H., Li Q. (2026). A Visible
and NIR Light-Driven Photoswitch With High Fluorescence On/Off Contrast
and Near-Quantitative Cyclization Yield for Photonic Applications. Adv. Funct. Mater..

[72] Gilat S. L., Kawai S. H., Lehn J.-M. (1995). Light-Triggered Molecular Devices:
Photochemical Switching Of Optical and Electrochemical Properties
in Molecular Wire Type Diarylethene Species. Chem.—Eur. J..

[73] Gwebu S., Marshall D., Sonar P., Philippa B., Vamvounis G. (2024). Structure–Property
Relationships of Solution-Processable Diarylethene-Based Main-Chain
Photochromic Polymers. Macromol. Chem. Phys..

[74] Liu J.-X., Xin B., Li C., Xie N.-H., Gong W.-L., Huang Z.-L., Zhu M.-Q. (2017). PEGylated Perylenemonoimide-Dithienylethene
for Super-Resolution
Imaging of Liposomes. ACS Appl. Mater. Interfaces.

[75] Liu G., Zhang Y.-M., Xu X., Zhang L., Liu Y. (2017). Optically
Switchable Luminescent Hydrogel by Synergistically Intercalating Photochromic
Molecular Rotor into Inorganic Clay. Adv. Opt.
Mater..

[76] Fu H.-G., Wu X., Wang H.-J., Zhang F., Chen Y., Xu J. (2025). Color-Tunable
Multi-Stimuli-Responsive Luminescent System Based on Diarylethene
and Photoacid. Chin. Chem. Lett..

[77] Mantero M. C., Oggioni L., Pariani G., Ortica F., Tosi S., Canepa M., Bertarelli C., Tommasini M., Bianco A. (2020). High Response Photochromic Films
Based on D–A
Diarylethenes and Their Application in Holography. RSC Adv..

[78] Oggioni L., Toccafondi C., Pariani G., Colella L., Canepa M., Bertarelli C., Bianco A. (2017). Photochromic Polyurethanes Showing
a Strong Change of Transparency and Refractive Index. Polymers.

[79] Liu G., Tian C., Li G., Zhang H., Fan X., Liu L., Cao Z., Jiang S., Zheng X., Niu C., Xu X. (2023). Conformational
Restriction-Induced Dual Visible Light-Switched Diarylethene
Fluorochrome by Composite Films. ACS Mater.
Lett..

[80] Das P., Grinalds N. J., Ghiviriga I., Abboud K. A., Dobrzycki Ł., Xue J., Castellano R. K. (2024). Dicyanorhodanine-Pyrrole
Conjugates
for Visible Light-Driven Quantitative Photoswitching in Solution and
the Solid State. J. Am. Chem. Soc..

[81] Tosic O., Altenhöner K., Mattay J. (2010). Photochromic Dithienylethenes with
Extended π-Systems. Photochem. Photobiol.
Sci..

[82] Patel D. G., Feng F., Ohnishi Y., Abboud K. A., Hirata S., Schanze K. S., Reynolds J. R. (2012). It Takes
More Than an Imine: The
Role of the Central Atom on the Electron-Accepting Ability of Benzotriazole
and Benzothiadiazole Oligomers. J. Am. Chem.
Soc..

[83] Ertl P. A. (2022). Web Tool
for Calculating Substituent Descriptors Compatible with Hammett Sigma
Constants. Chem.: Methods.

[84] Meier H., Stalmach U., Kolshorn H. (1997). Effective Conjugation
Length and
UV/Vis Spectra of Oligomers. Acta Polym..

[85] Kutuvantavida Y., Williams G. V. M., Bhuiyan M. D. H., Raymond S. G., Kay A. J. (2015). Effects
of Chromophore Conjugation Length and Concentration on the Photostability
of Indoline-Based Nonlinear Optical Chromophore/Polymer Films. J. Phys. Chem. C.

[86] Wei S.-C., Pan M., Fan Y.-Z., Liu H., Zhang J., Su C.-Y. (2015). Creating
Coordination-Based Cavities in a Multiresponsive Supramolecular Gel. Chem.—Eur. J..

[87] Higashiguchi K., Matsuda K., Kobatake S., Yamada T., Kawai T., Irie M. (2000). Fatigue Mechanism of Photochromic 1,2-Bis­(2,5-Dimethyl-3-Thienyl)­Perfluorocyclopentene. Bull. Chem. Soc. Jpn..

[88] Porter, A. E. A. Radical Reactions of Thiophene. Chemistry of Heterocyclic Compounds; Chemistry of Heterocyclic Compounds: A Series Of Monographs; John Wiley & Sons, 1985; pp 651–670. 10.1002/9780470187234.ch9.

[89] Rapta P., Zeika O., Rohde D., Hartmann H., Dunsch L. (2006). Thiophene–Thiophene
versus Phenyl–Phenyl Coupling in 2-(Diphenylamino)-Thiophenes:
An ESR-UV/Vis/NIR Spectroelectrochemical Study. ChemPhysChem.

[90] Detková K. R., Jakubcová K., Malatinský T., Filo J., Cigáň M., Budzák S. ˇ., Medved’ M., Jakubec P. (2026). Regioselective C-Arylation of Functionalized Nitroalkanes
with Furan, Thiophene, and Substituted Thiophenes. J. Org. Chem..

[91] Oggioni L., Pariani G., Zamkotsian F., Bertarelli C., Bianco A. (2019). Holography with Photochromic Diarylethenes. Materials.

[92] Lu T., Chen F. M. (2012). A Multifunctional
Wavefunction Analyzer. J. Comput. Chem..

[93] Fu Q.-T., Yan X., Li T., Zhang X.-Y., He Y., Zhang W.-D., Liu Y., Li Y., Gu Z.-G. (2019). Diarylethene-Based Conjugated Polymer
Networks for Ultrafast Photochromic Films. New
J. Chem..

[94] Toccafondi C., Occhi L., Cavalleri O., Penco A., Castagna R., Bianco A., Bertarelli C., Comoretto D., Canepa M. (2014). Photochromic and Photomechanical
Responses of an Amorphous
Diarylethene-Based Polymer: A Spectroscopic Ellipsometry Investigation
of Ultrathin Films. J. Mater. Chem. C.

[95] Gutiérrez-Villarreal M. H., Zavala-Betancourt S. A. (2014). Thermo-Oxidative Stability of Cyclic Olefin Copolymers
in the Presence of Fe, Co and Mn Stearates as Pro-Degradant Additives. Polym.-Plast. Technol. Eng..

[96] Zhang F., Mao X., Lei H., Guo F., Shen R., Xing Z., Wu G. (2024). Investigation on Discoloration
Mechanism of Cyclic Olefin Copolymer
under Ionizing Irradiation Sterilization. Polym.
Degrad. Stab..

[97] Li M., Yuan Y., Chen Y. (2018). Acid-Induced
Multicolor Fluorescence
of Pyridazine Derivative. ACS Appl. Mater. Interfaces.

[98] Meanwell N. A. (2023). The Pyridazine
Heterocycle in Molecular Recognition and Drug Discovery. Med. Chem. Res..

[99] Lien E. J., Kumler W. D. (1970). Dipole Moment and Structure of Thiophene
Derivatives
and Benzene Analogs. J. Pharm. Sci..

[100] Lindic M. M., Zajonz M., Hebestreit M.-L., Schneider M., Meerts W. L., Schmitt M. (2018). Excited State Dipole
Moments of Anisole in Gas Phase and Solution. J. Photochem. Photobiol. Chem..

[101] Gottlieb H. E., Kotlyar V., Nudelman A. (1997). NMR Chemical
Shifts
of Common Laboratory Solvents as Trace Impurities. J. Org. Chem..

