# Supplementary material for: Toward Higher Fatigue Resistance of Photochromic Polymer Layers Containing Novel Diarylethenes
Source: ACS Omega. 2026 Jul 16;11(29):44443–61. doi: 10.1021/acsomega.6c05374 (PMC13425520; doi:10.1021/acsomega.6c05374)
Supplement: Supplementary file 1 [file ao6c05374_si_001.pdf]

Supporting Information

Towards Higher Fatigue Resistance of Photochromic Polymer Layers Containing Novel Diarylethenes

*Mattes Plieth[#], André Eitzeroth[‡], Henrik Hercht[#], Jingrun Zhang[†], Sven Nagorny[◊], Christian Rembe[†], Jörg Adams[‡], Andreas Schmidt[#]**

[#] Technische Universität Clausthal, Institute of Organic Chemistry
Leibnizstr. 6, 38678 Clausthal-Zellerfeld, Germany

† Technische Universität Clausthal, Institute for Electrical Information Technology, Leibnizstr. 10, 38678 Clausthal-Zellerfeld, Germany

‡ Technische Universität Clausthal, Institute of Physical Chemistry, Arnold-Sommerfeld-Str. 4, 38678 Clausthal-Zellerfeld, Germany

◊ Max Planck Institute for Multidisciplinary Sciences, Department of NanoBiophotonics, Am Faßberg 11, 37077 Göttingen, Germany

Table of Contents

Materials and characterizations: Experimental procedures and spectra ………………………… 3

DFT calculations ………………………………………………………………………………… 45

Table S1. ΔG-calculations using B3LYP/Def2TZVP GD3BJ ………………………………….. 72

Table S2. ΔG-calculations using B3LYP/36G d,p ……………………………….………….. 72

Table S3. Change of the distance of C2-C2' positions in PMMA (B3LYP/Def2TZVP) ………. 73

Table S4. HOMO/LUMO Gap, in PMMA B3LYP/Def2TZVP ………………………………… 73

Table S5. Change in volume and surface area of the selected DAEs …………………………… 73

Table S6. Diameter change of the system in Angström ………………………………………… 74

Table S7. Hammett parameters and calculated excitation energy differences used
for the correlation plot (Figure 4). Hammett parameters have been calculated using
the webtool published by Ertl …………………………………………………….………… 75

Table S8. Location of uv and visible light maxima as well as the isosbestic point of
the DAEs used in this work……………………………………………………………….. 75

Table S9. Specification and seller details for the polymers used for film preparation.
$\mathbf{T_g}$ values marked with * have been obtained by DSC measurement …………………………… 75

Table S10. Results of synthesized polymers with targeted and resulted glass transition
temperature ……………………………………………………………………………….. 76

Setup for stability measurements of photochromes ……………………………………......... 77

Materials and characterizations: Experimental Procedures and Spectra

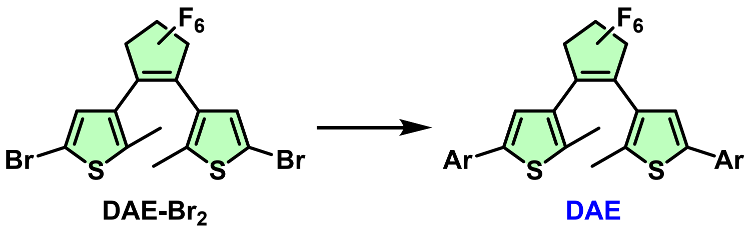

Scheme S1. Synthesis of DAEs via Suzuki-Miyaura coupling.

General Procedure A for the Synthesis of DAEs

The reactions are carried out under nitrogen atmosphere. 1 eq. of DAE-Br_2, 2.5 eq. of the arylboronic species, 2.5 eq. of K_3PO_4 and 0.05 eq. of G3 SPhos Pd ((2-dicyclohexylphosphino-2',6'-dimethoxybiphenyl)[2-(2'-amino-1,1'-biphenyl)]palladium(II) methanesulfonate) were dried under vacuum in an oven dried Schlenk flask. The mixture was dissolved in 32 mL of dry dioxane and degassed water (sparged with nitrogen for 20 min) (3:1 *v*:*v*) and stirred under refluxing conditions for 12 h. Afterwards the reaction mixture was allowed to cool to room temperature and was quenched with a sat. NaCl solution. The resulting biphasic mixture was separated and the water phase was extracted three times with small amounts (20-30 mL) diethyl ether. The combined organic phases were washed two times with a sat. NaCl solution, one time with water and dried over $MgSO_4$. The solids were removed by filtration and the solvents were removed *in vacuo*. The crude product was adsorbed onto a small amount of silica and further purified *via* column chromatography using petroleum ether or petroleum ether/ ethyl acetate mixture as stated below to yield the corresponding DAE.

General Procedure B for the Synthesis of DAEs

The reactions were carried out under nitrogen atmosphere. 1 eq. of aryl-substituted thiophene was dissolved in 25-60 mL of dry THF and cooled to -78 °C. Following a cooling time of 15 min 1.25 eq of a 2.8 M solution of *n*-Butyllithium (*n*-BuLi) in hexane were added to the starting material while stirring vigorously. After 30 min of reaction time, 0.5 eq. of octafluorocyclopentene were added with a cooled syringe. After another hour of cooling, the mixture was allowed to warm to room temperature and stirred for 12 h. To quench the reaction the solvent amount of a sat. NaCl solution was added to the mixture and the phases were separated if possible. The water phase was extracted three times with small amounts (20-30 mL) of diethyl ether. The combined organic phases were washed two times with a sat. NaCl solution, one time with water and dried over $MgSO_4$. The solids were removed by filtration and the solvents were removed *in vacuo*. The crude product was adsorbed onto a small amount of silica and further purified *via* column chromatography using petroleum ether or petroleum ether/ ethyl acetate mixture as stated below to yield the corresponding DAE.

General Procedure C for the Synthesis of Aryl-substituted Thiophenes

The reactions were carried out under nitrogen atmosphere. 1 eq. of 3,5-dibromo-2-methylthiophene, 1 to 1.1 eq. of arylboronic acid, 3 eq. of Na_2CO_3 and 0.05 to 0.1 eq. of $Pd(PPh_3)_4$ were added to a oven dried Schlenk flask under nitrogen. The mixture was evacuated and filled with nitrogen for three times. After that 60 mL of a 5:1 mixture of THF and degassed water (sparged with nitrogen for 20 min) were used to solve the solids. The solution was refluxed under vigorous stirring for 12 h. To quench the reaction the solvent amount of a sat. NaCl solution was added to the mixture and the phases were separated. The water phase was extracted three times with small amounts (20-30 mL) of diethyl ether. The combined organic phases were washed two times with a sat. NaCl solution, one time with water and dried over $MgSO_4$. The

solids were removed by filtration and the solvents were removed *in vacuo*. The crude product was adsorbed onto a small amount of silica and further purified *via* column chromatography using petroleum ether or petroleum ether/ ethyl acetate mixture as stated below to yield the corresponding DAE precursors.

General Procedure D for the Synthesis of Aryl-substituted Thiophenes
The reactions were carried out under nitrogen atmosphere. 1 eq. of (4-bromo-5-methylthiophen-2-yl)boronic acid, 1 to 1.1 eq. of arylbromide, 3 eq. of Na_2CO_3 or K_2CO_3 and 0.05 to 0.1 eq. of $Pd(PPh_3)_4$ were added to a oven dried Schlenk flask under nitrogen. The mixture was evacuated and filled with nitrogen for three times. After that 60 mL of a 5:1 mixture of THF and degassed water (sparged with nitrogen for 20 min) were used to solve the solids. The solution was refluxed under vigorous stirring for 12 h. To quench the reaction the solvent amount of a sat. NaCl solution was added to the mixture and the phases were separated. The water phase was extracted three times with small amounts (20-30 mL) diethyl ether. The combined organic phases were washed two times with a sat. NaCl solution, one time with water and dried over $MgSO_4$. The solids were removed by filtration and the solvents were removed *in vacuo*. The crude product was adsorbed onto a small amount of silica and further purified *via* column chromatography using petroleum ether or petroleum ether/ ethyl acetate mixture as stated below to yield the corresponding DAE precursors.

Synthesis of 3,5-dibromo-2-methylthiophene

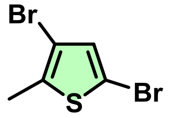

The synthesis follows a modified literature procedure.[1] 5.00 g of 2-methylthiophene are dissolved in 50 mL of acetic acid. Under vigorous stirring 19.94 g (2.2 eq.) of *N*-bromosuccinimide were added in 5 portions of a period of 45 minutes. The mixture was stirred at room temperature in the dark for 16 h. To quench the reaction 100 mL of a 1M NaOH solution was added. The resulting mixture was extracted 5 times with diethyl ether. The combined organic phases were washed with 1M NaOH solution for 3 times and with saturated $Na_2S_2O_3$ solution 2 times (or until the water phase remained colorless). The resulting solution was dried over $MgSO_4$. The solids were removed by filtration and the crude was concentrated *in vacuo*. The mixture was directly applied onto a silica plug and eluted from the stationary phase with petroleum ether. Removing of the solvents resulted in 12.32 g of a colorless to light yellow liquid (yield 95%). ^{1}H NMR (400 MHz, $CDCl_3$): δ = 6.85 (s, 1H), 2.83 (s, 3H) ppm. HRMS (APCI): calc.: 253.8400 [M], found: 253.890 [M].
Spectroscopic data are in agreement with those reported in the literature.[1]

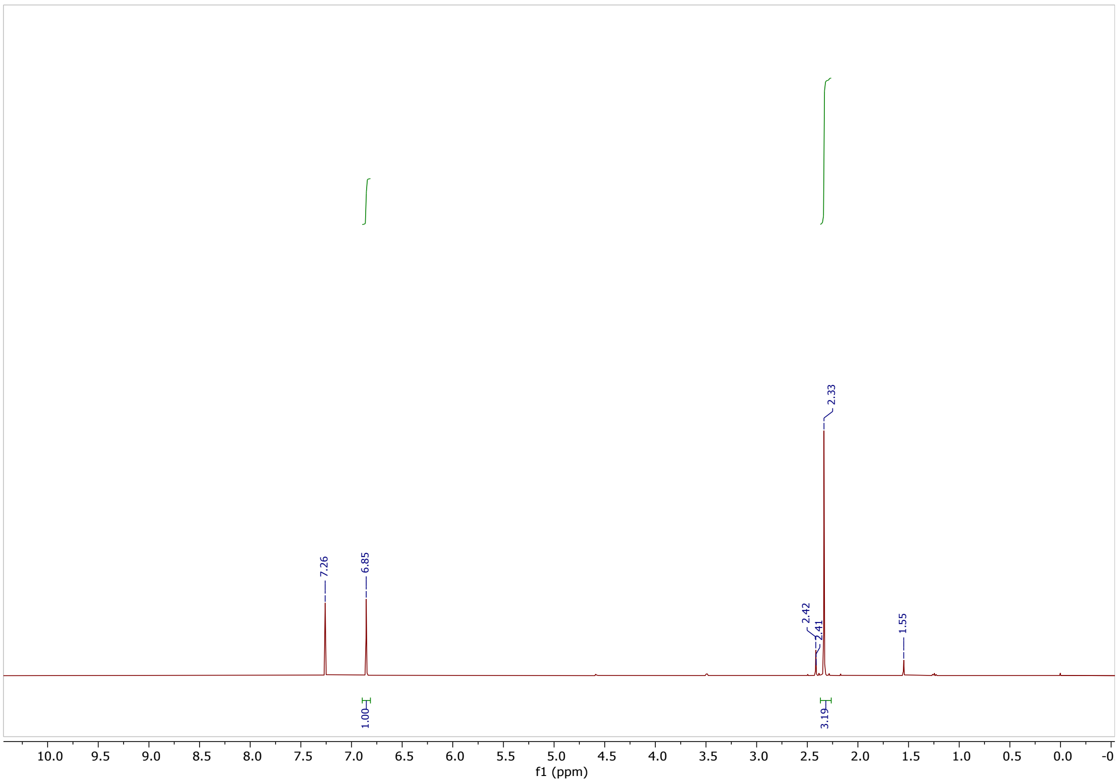

Synthesis of (4-bromo-5-methylthiophen-2-yl)boronic acid

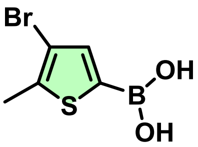

The synthesis follows a modified literature procedure.[1] In an oven dried Schlenk flask, 12.20 g of 3,5-dibromo-2-methylthiophene (1.0 eq.) were dissolved in 100 mL dry diethyl ether under nitrogen. The resulting solution was cooled to -78 °C. Then, 18.72 mL (1.1 eq.) of a 2.8 M solution of *n*-butyllithium in hexane were added carefully. The mixture was stirred for 45 minutes at -78 °C. After that, 17.83 mL (1.5 eq.) of tributylborate were added and the resulting suspension was stirred additionally for 30 min. The reaction was warmed up to room temperature and stirred for 12 h. To quench the reaction, small amounts (<10 mL) of conc. HCl were added, until the precipitates were dissolved completely. The product was extracted from the crude with up to 200 mL of a 1M solution NaOH. The water phases were combined and the product was precipitated with addition of necessary amounts of conc. HCl. The resulting light brown solid was removed by filtration and dried *in vacuo* to give 10.50 g of (4-bromo-5-methylthiophen-2-yl)boronic acid (quantitative yield). ^{1}H NMR (400 MHz, DMSO d_6): δ = 8.29(bs, 2 H), 7.51 (s, 1H), 2.36 (s, 3H) ppm. HRMS (APCI): calc.: 219.9365 [M], found: 219.9353 [M].
Spectroscopic data are in agreement with those reported in the literature.[1]

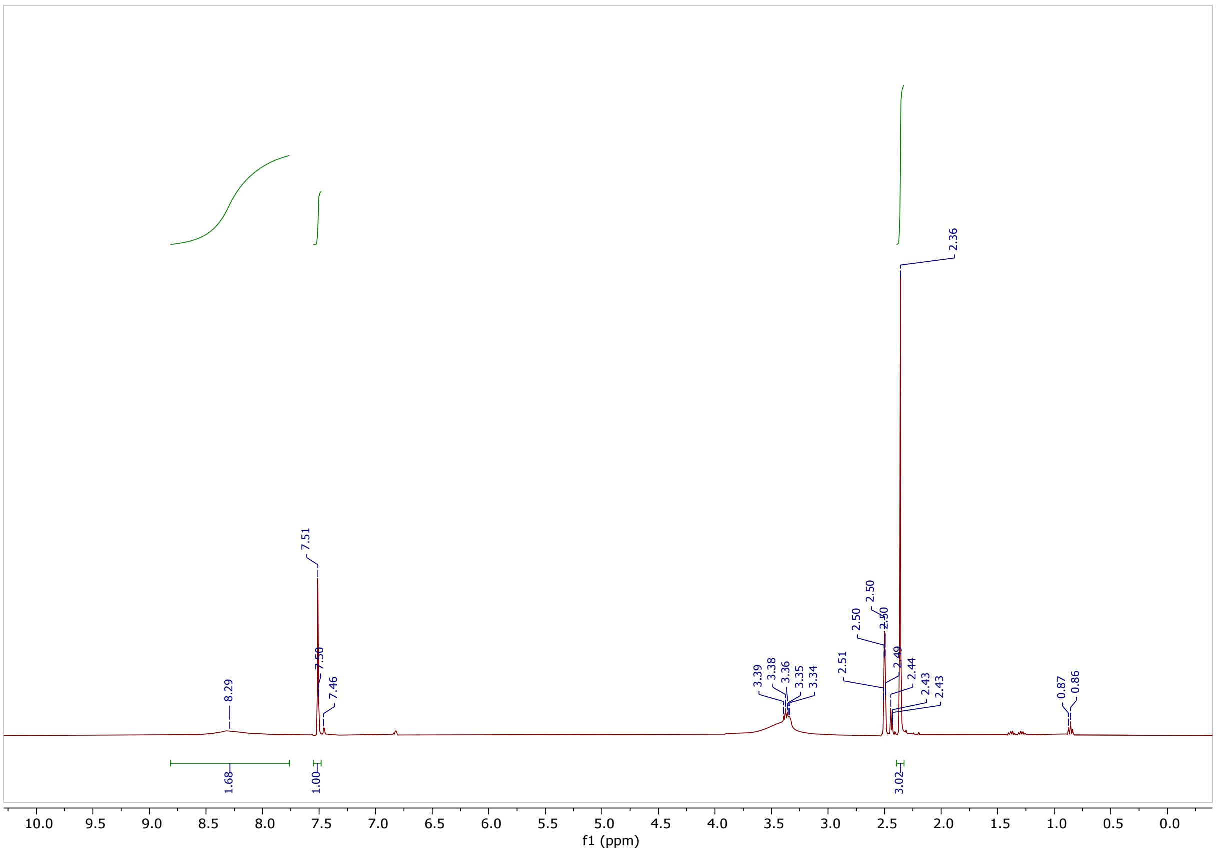

Synthesis of DAE-1

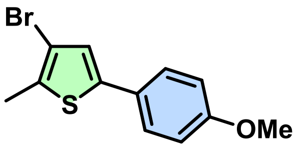

Following General Procedure C, 1.50 g bromothiophene (1.05 eq.), 0.78 g (4-methoxyphenyl)boronic acid (2.50 eq.), 1.55 g Na_2CO_3 (3.00 eq.) and 0.28 g $Pd(PPh_3)_4$ (0.05 eq.) were used. The resulting crude product was purified *via* column chromatography using a petroleum ether/ ethyl acetate mixture (20:1 *v*:*v*) to give 1.06 g of DAE-1 precursor as a beige solid (yield 77%). ^{1}H NMR (400 MHz, $CDCl_3$): δ = 7.44 (d, J= 8.8 Hz, 2H), 6.98 (s, 1H), 6.90 (d, J= 8.8 Hz, 2H), 3.83 (s, 3H), 2.40 (s, 3H) ppm. HRMS (APCI): calc.: 281.9174 [M], found: 281.9705 [M].
Spectroscopic data are in agreement with those reported in the literature.[2]

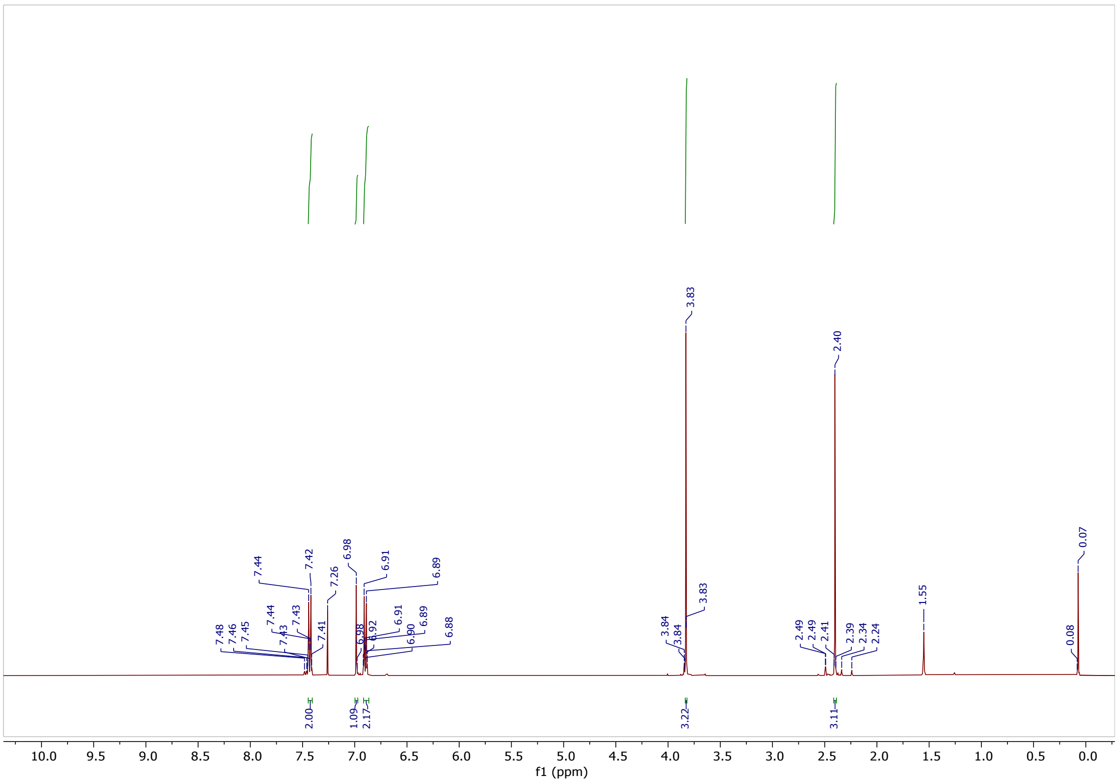

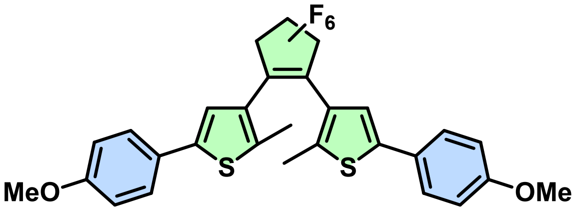

Following General Procedure B, 936 mg 3-bromo-5-(4-methoxyphenyl)-2-methylthiophene (1.00 eq.), 1.47 mL *n*-BuLi (2.8 M in hexane, 1.25 eq.) and 222 µl octafluorocyclopentene (0.50 eq.) reacted in 35 mL of dry THF. The crude product was purified *via* column chromatography using a gradient of petroleum ether/ ethyl acetate (20:1 to 15:1 *v*:*v*) mixture to give 748 mg of DAE-1 as an off white powder (yield 39%). ^{1}H NMR (400 MHz, $CDCl_3$): δ = 7.48-7.45 (m, 4H), 7.16 (s, 2H), 6.93-6.90 (m, 4H), 3.84 (s, 6H), 1.95 (s, 6H) ppm. ^{13}C NMR (100 MHz, $CDCl_3$): δ=159.6 (o), 142.2 (o), 140.5 (o), 127.1 (o), 126.4 (+), 125.9 (o), 121.5 (+), 114.5 (+), 55.6 (+), 14.6 (+) ppm. IR (ATR): $\tilde{\nu}$ = 2957, 2836, 1609, 1511, 1439, 1337, 1247, 1177, 1089, 1029, 986, 887, 821, 740, 505 cm^{-1}. HRMS (APCI): calc.: 581.1038 [M+H^{+}], found: 581.1036 [M+H^{+}].
Spectroscopic data are in agreement with those reported in the literature.[3]

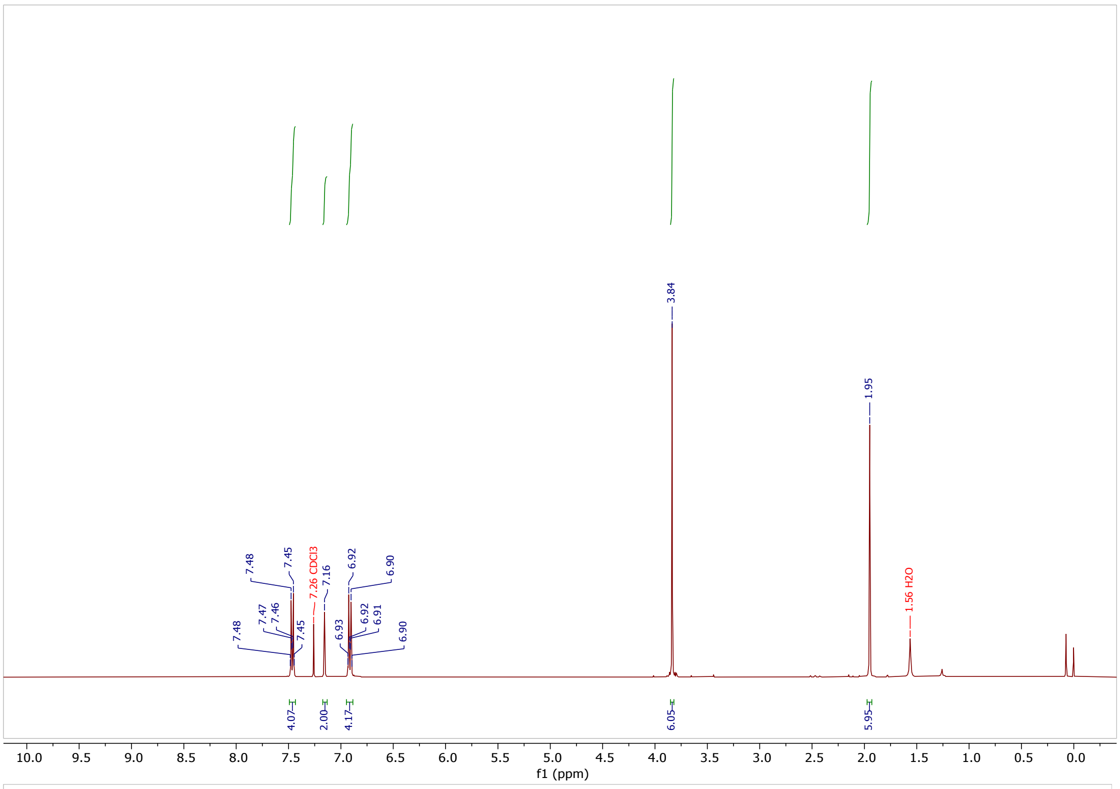
7.48
7.45
7.26 CDCl3
7.16
6.92
6.90
7.48
7.47
7.46
7.45
6.93
6.92
6.91
6.90
3.84
1.95
1.56 H2O
4.07
2.00
4.17
6.05
5.95
f1 (ppm)

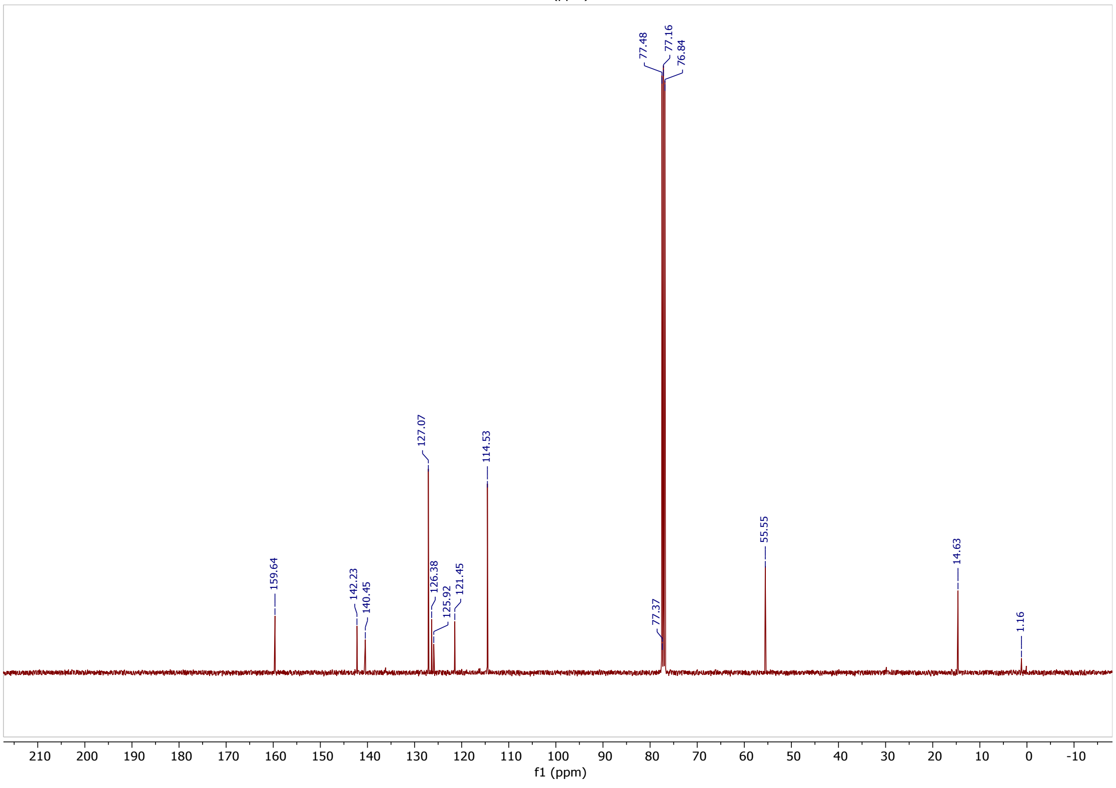
77.48
77.16
76.84
127.07
114.53
55.55
159.64
142.23
140.45
126.38
125.92
121.45
77.37
14.63
1.16
f1 (ppm)

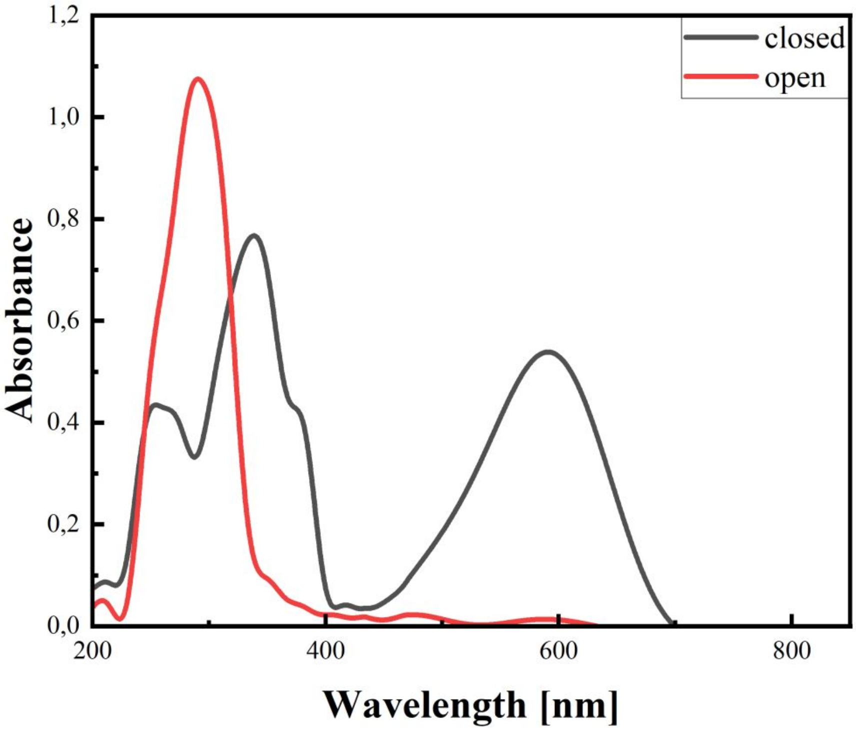

Synthesis of DAE-2

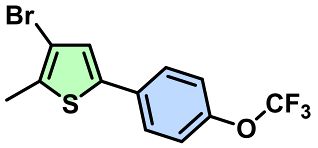

Following General Procedure C, 800 mg bromothiophene (1.00 eq.), 694 mg (4-(trifluoromethoxy)phenyl)boronic acid (1.10 eq.), 812 mg Na_2CO_3 (2.50 eq.) and 248 mg $Pd(PPh_3)_4$ (0.07 eq.) were used. The resulting crude product was purified *via* column chromatography using a petroleum ether as eluent to give 890 mg of DAE-2 precursor as a white solid (yield 87%). ^{1}H NMR (400 MHz, $CDCl_3$): δ=7.52 (d, J= 8.8 Hz, 2H), 7.21 (d, J= 8.2 Hz, 2H), 7.09 (s, 1H), 2.42 (s, 3H) ppm. HRMS (APCI): calc.: 335.9426 [M], found: 335.9426 [M].

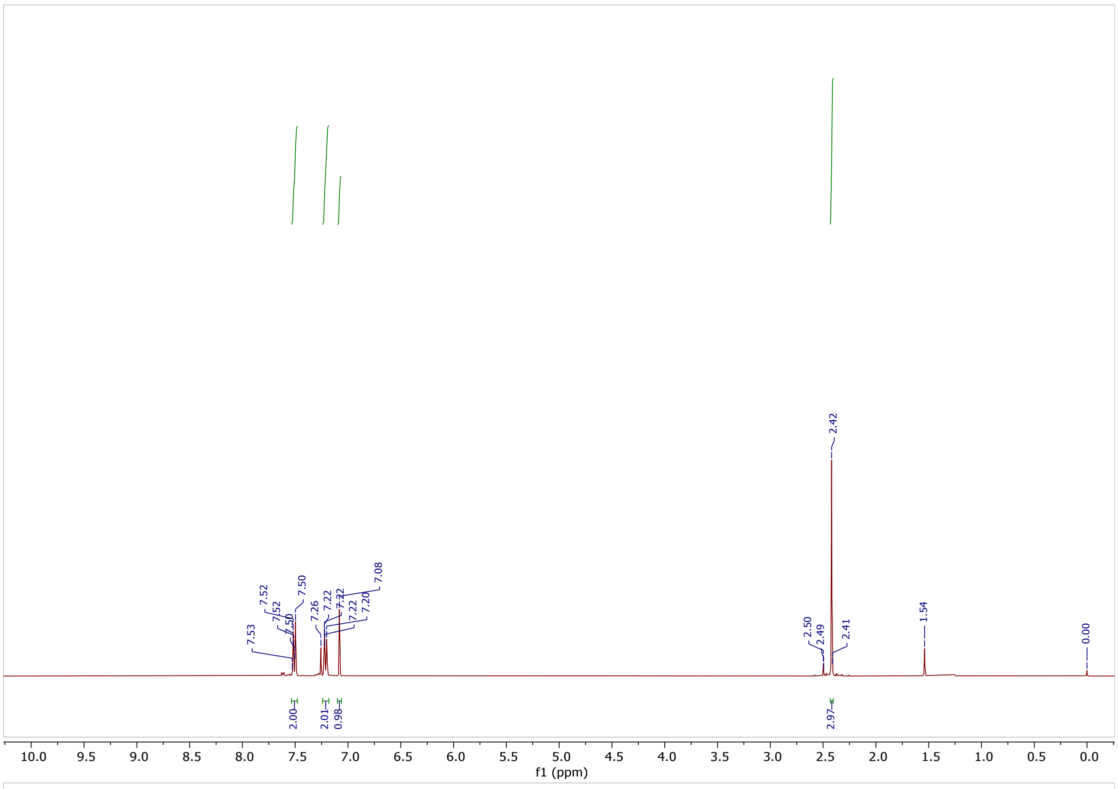
7.53
7.52
7.52
7.50
7.50
7.26
7.22
7.22
7.22
7.20
7.08
2.50
2.49
2.42
2.41
1.54
0.00
2.00
2.01
0.98
2.97
10.0 9.5 9.0 8.5 8.0 7.5 7.0 6.5 6.0 5.5 5.0 4.5 4.0 3.5 3.0 2.5 2.0 1.5 1.0 0.5 0.0
f1 (ppm)

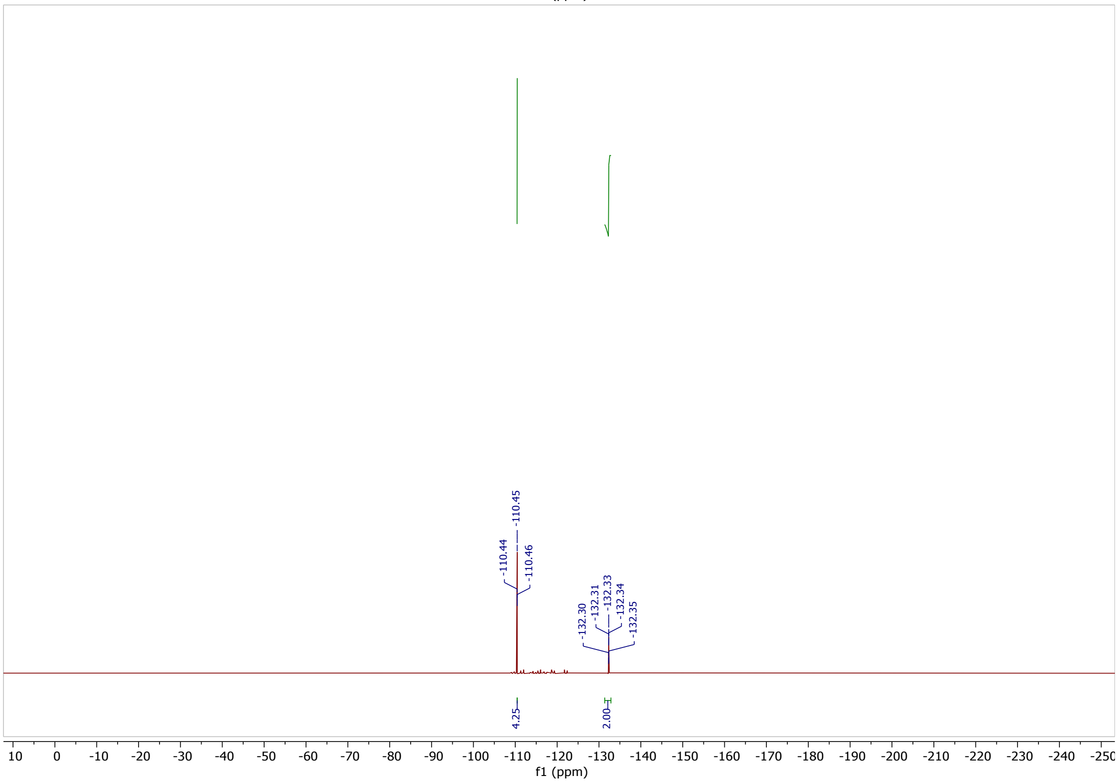
-110.44
-110.45
-110.46
-132.30
-132.31
-132.33
-132.34
-132.35
4.25
2.00
10 0 -10 -20 -30 -40 -50 -60 -70 -80 -90 -100 -110 -120 -130 -140 -150 -160 -170 -180 -190 -200 -210 -220 -230 -240 -250
f1 (ppm)

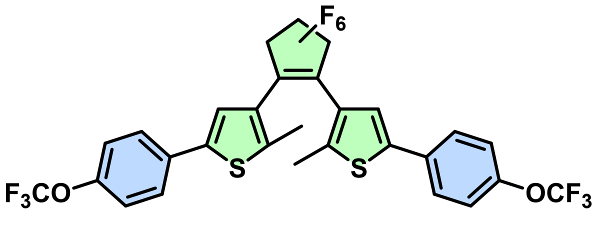
F6
S
S
F3CO
OCF3

Following General Procedure B, 1 g 3-bromo-2-methyl-5-(4-(trifluoromethoxy)phenyl)thiophene (1.00 eq.), 1.3 mL *n*-BuLi (2.8 M in hexane, 1.25 eq.) and 0.199 mL octafluorocyclopentene (0.50 eq.) reacted in 40 mL of dry THF. The crude product was purified *via* column chromatography using a gradient of petroleum ether to petroleum ether/ ethyl acetate (50:1 *v*:*v*) to give 346 mg of DAE-2 as a beige powder (yield 34%). ^{1}H NMR (400 MHz, $CDCl_3$): δ = 7.57-7.53 (m, 4H), 7.26-7.22 (m, 6H), 1.98 (s, 6H) ppm. ^{13}C NMR (100 MHz, $CDCl_3$): δ = 148.9 (o), 142.0 (o), 140.9 (o), 132.2 (o), 127.1 (+), 126.1 (o), 123.1 (+), 121.5 (+), 119.7 (o), 118.0 (o), 116.3 (o), 14.7 (+) ppm. IR (ATR): $\tilde{\nu}$ = 2924, 1556, 1513, 1439, 1251, 1212, 1165, 1107, 1055, 980, 898, 805, 740, 614, 564, 490 cm^{-1}. HRMS (APCI): calc.: 688.0400 [M+H^{+}], found: 688.0394 [M+H^{+}].

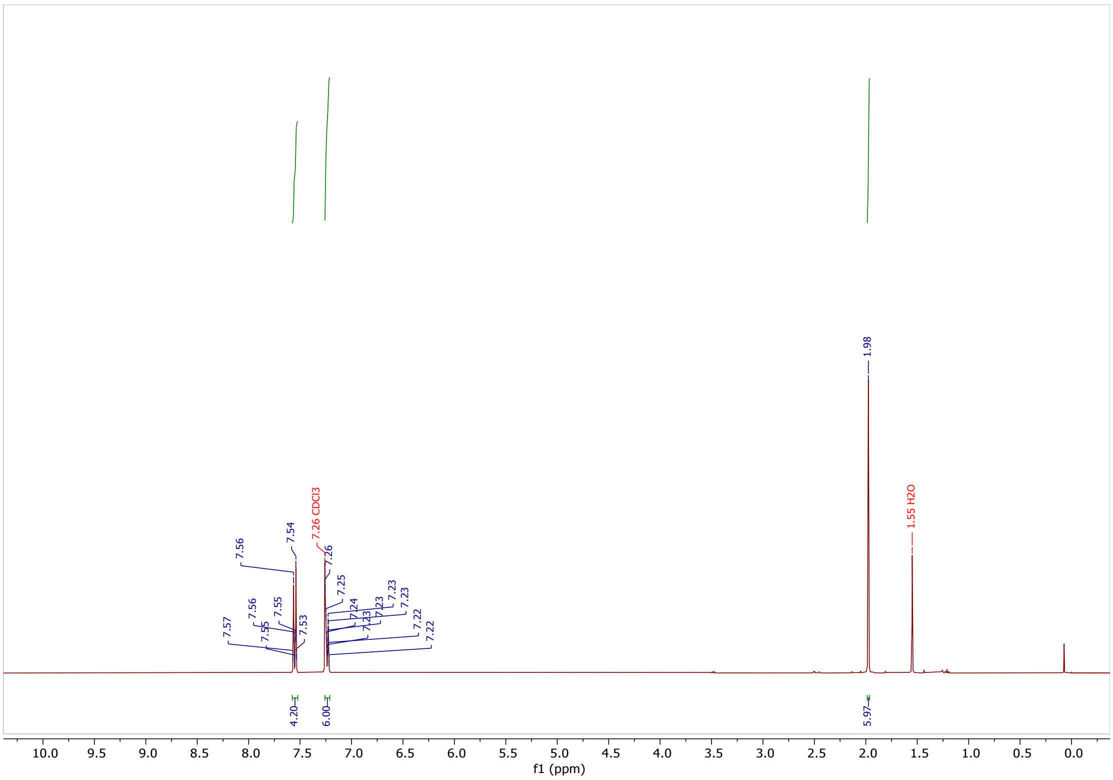

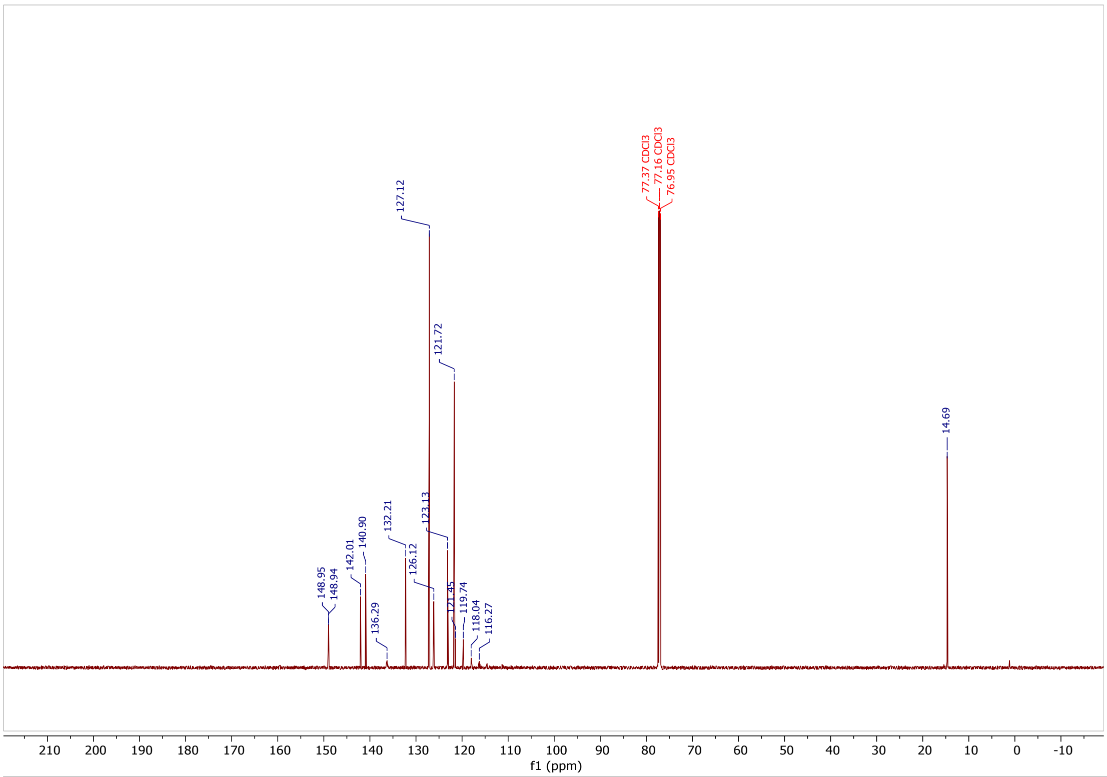
148.95
148.94
142.01
140.90
136.29
132.21
127.12
126.12
123.13
121.72
121.45
119.74
118.04
116.27
77.37 CDCl3
77.16 CDCl3
76.95 CDCl3
14.69
210 200 190 180 170 160 150 140 130 120 110 100 90 80 70 60 50 40 30 20 10 0 -10
f1 (ppm)

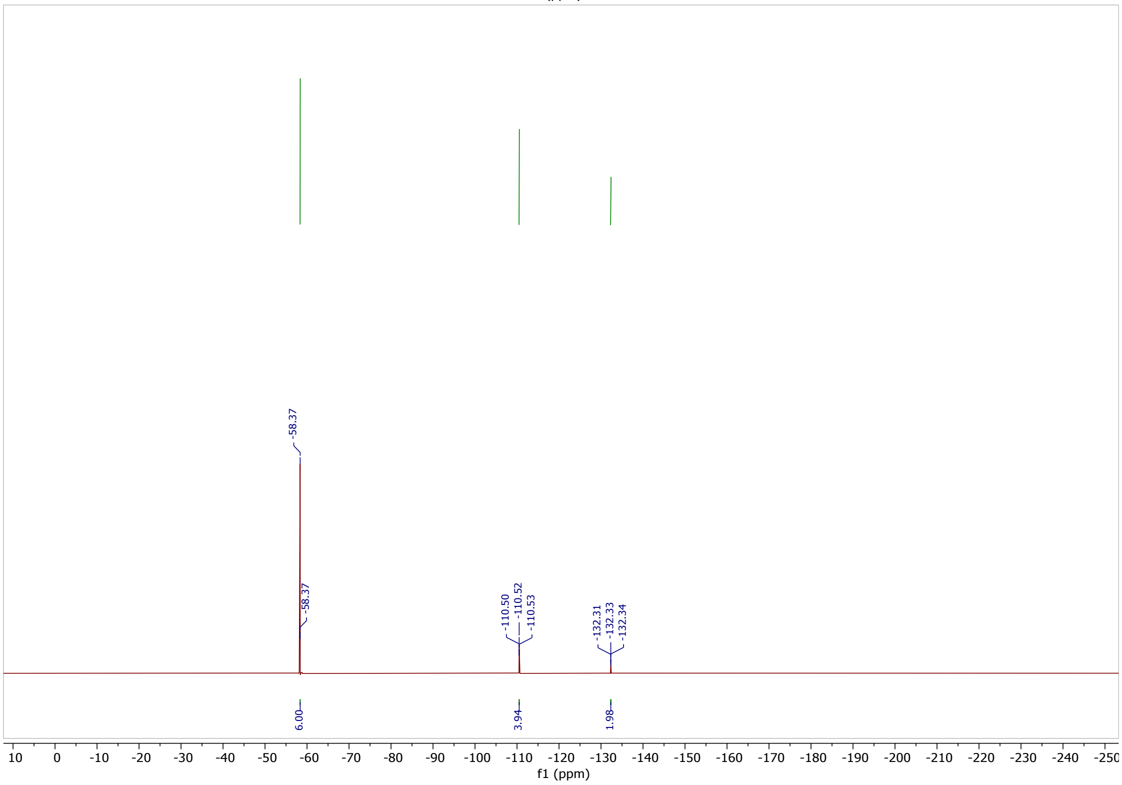
-58.37
-58.37
-110.50
-110.52
-110.53
-132.31
-132.33
-132.34
6.00
3.94
1.98
10 0 -10 -20 -30 -40 -50 -60 -70 -80 -90 -100 -110 -120 -130 -140 -150 -160 -170 -180 -190 -200 -210 -220 -230 -240 -250
f1 (ppm)

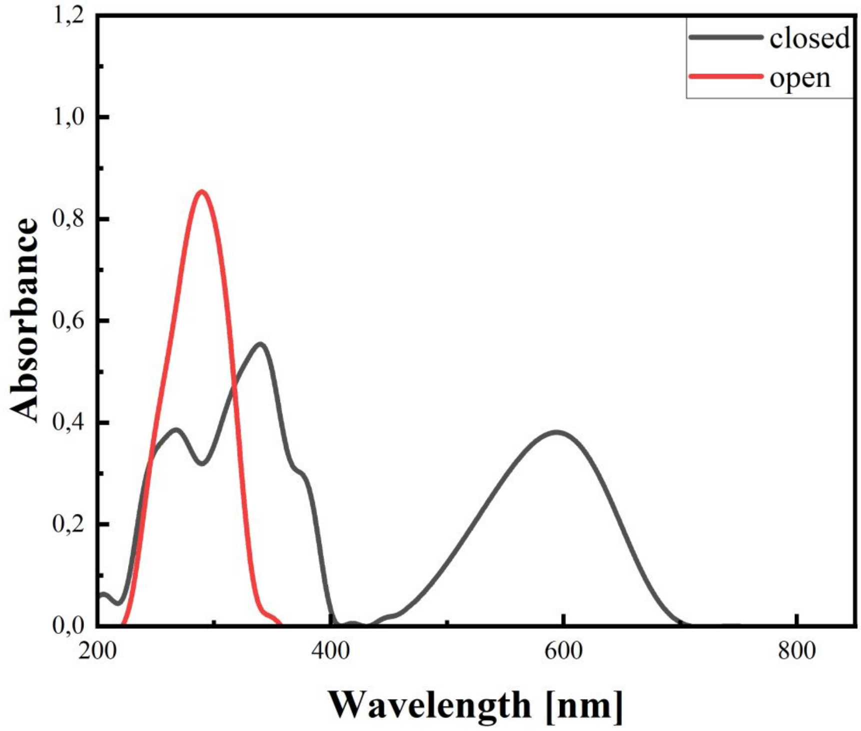

Synthesis of DAE-3

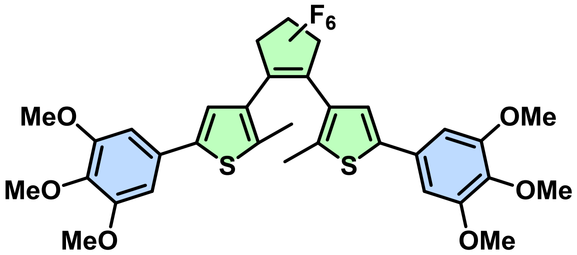

Following General Procedure A, 205.5 mg DAE-Br_2 (1.00 eq.), 268 mg potassium 3,4,5-trimethoxyphenyltrifluoroborate (2.50 eq.), 208 mg K_3PO_4 (2.50 eq.) and 15.2 mg G3 SPhos Pd (0.05 eq.) were used. The resulting crude product was purified *via* column chromatography using a gradient of petroleum ether/ ethyl acetate mixture (5:1 to 2:1 *v*:*v*) to give 126.5 mg of DAE-3 as a light blue solid (yield 47%). ^{1}H NMR (400 MHz, $CDCl_3$): δ = 7.15 (s, 2H), 6.71 (s, 4H), 3.90 (s, 12H), 3.86 (s, 6H), 2.00 (s, 6H) ppm. ^{13}C NMR (100 MHz, $CDCl_3$): δ=153.8 (o), 142.3 (o), 141.1 (o), 138.3 (o), 129.3 (o), 127.3 (o), 125.1 (o), 123.8 (o), 123.2 (o), 122.4 (o), 103.3 (+), 61.1 (+), 56.4 (+), 14.5 (+) ppm. IR (ATR): $\tilde{\nu}$ = 2999, 2942, 2827, 1578, 1509, 1454, 1408, 1334, 1315, 1268, 1235, 1188, 1113, 1004, 981, 888, 812, 764, 527 cm^{-1}. HRMS (APCI): calc.: 701.1461 [M+H$^+$], found: 701.1455 [M+H$^+$].

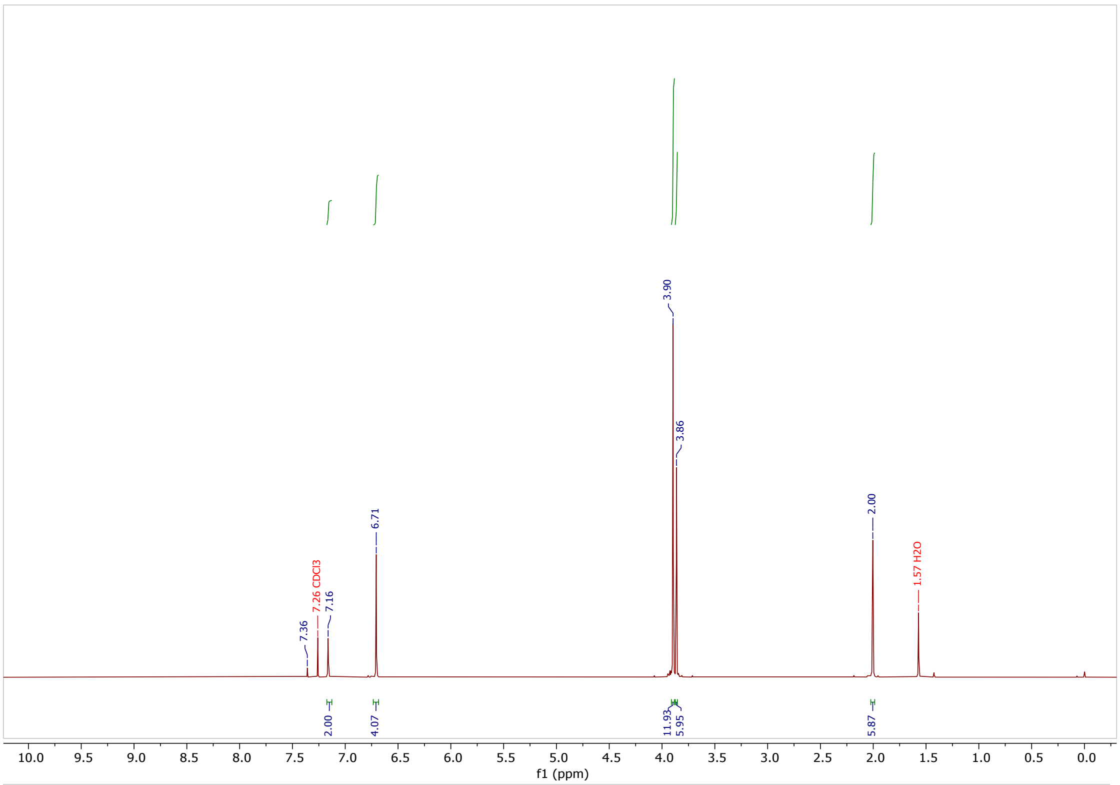
7.36
7.26 CDCl3
7.16
6.71
3.90
3.86
2.00
1.57 H2O
2.00
4.07
11.93
5.95
5.87
f1 (ppm)

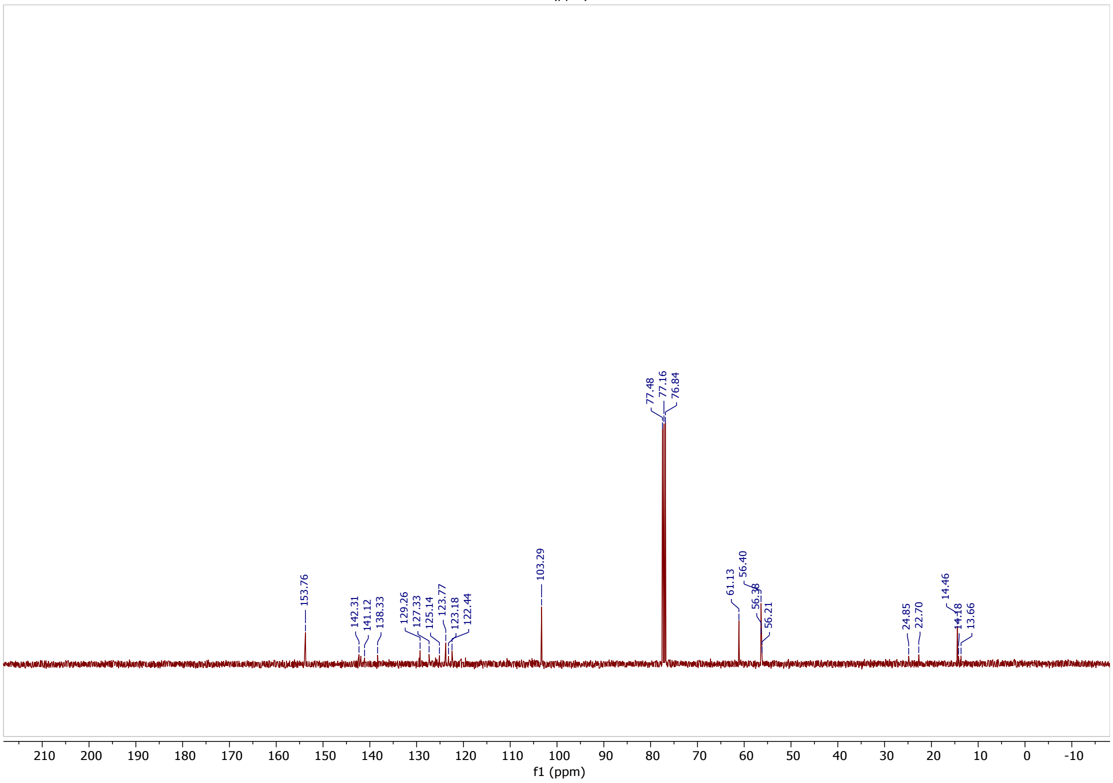
153.76
142.31
141.12
138.33
129.26
127.33
125.14
123.77
123.18
122.44
103.29
77.48
77.16
76.84
61.13
56.40
56.36
56.21
24.85
22.70
14.46
14.18
13.66
f1 (ppm)

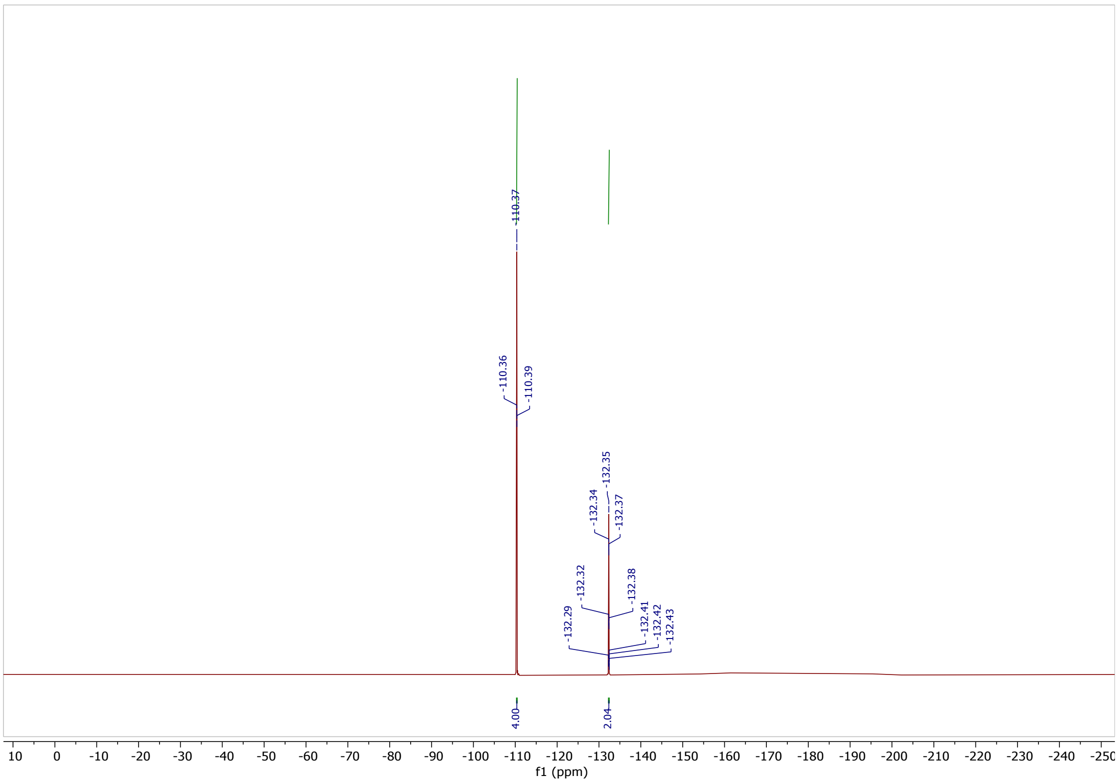

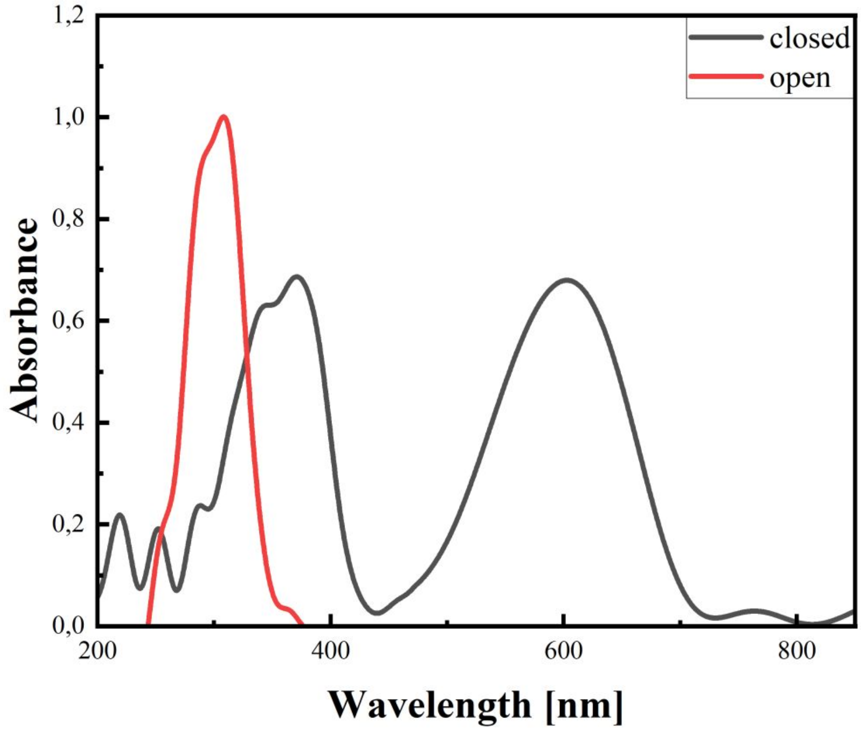

Synthesis of DAE-4

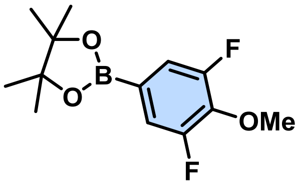

In an oven dried Schlenk flask 0.80 g (1.00 eq.) 5-bromo-1,3-difluoro-2-methoxybenzene, 1.06 g (1.20 eq.) bis(pinacolato)diboron, 0.94 g (2.75 eq.) KOAc and 0.18 g [1,1′-bis(diphenylphosphino)ferrocen]dichlorpalladium(II) were evacuated and flashed with nitrogen for 3 times. Then, the solids were suspended with the addition of 40 mL of dry dioxane. The mixture was refluxed under vigorous stirring for 12 h. To quench the reaction, 40 mL of water were added. The resulting biphasic mixture was separated and the water phase extracted 3 times with diethyl ether. The combined organic phases were washed with saturated NaCl solution 3 times and dried over $MgSO_4$. The solids were removed by filtration and the remaining solution concentrated *in vacuo*. The remaining crude product was mixed with silica and purified *via* column chromatography using a mixture of petroleum ether and ethylacetate (100:1 *v*:*v*) as the eluent to give 0.60 g of 2-(3,5-difluoro-4-methoxyphenyl)-4,4,5,5-tetramethyl-1,3,2-dioxaborolane as a blue liquid (yield 64%). ^{1}H NMR (400 MHz, $CDCl_3$): δ=7.30 (d, J = 9.0 Hz, 2H), 4.02 (s, 3H), 1.32 (s, 12H) ppm. HRMS (ESI): calc.: 293.1131 [$M+Na^+$], found: 293.1137 [$M+H^+$].

Spectroscopic data are in agreement with those reported in the literature.[4]

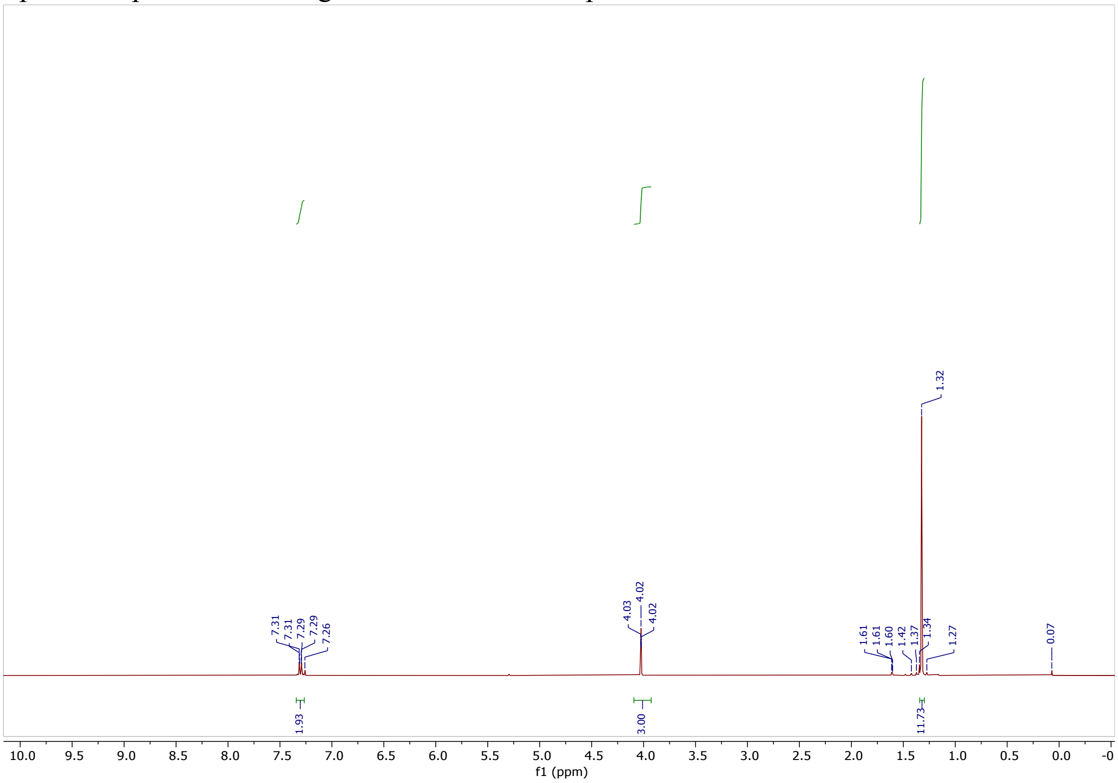

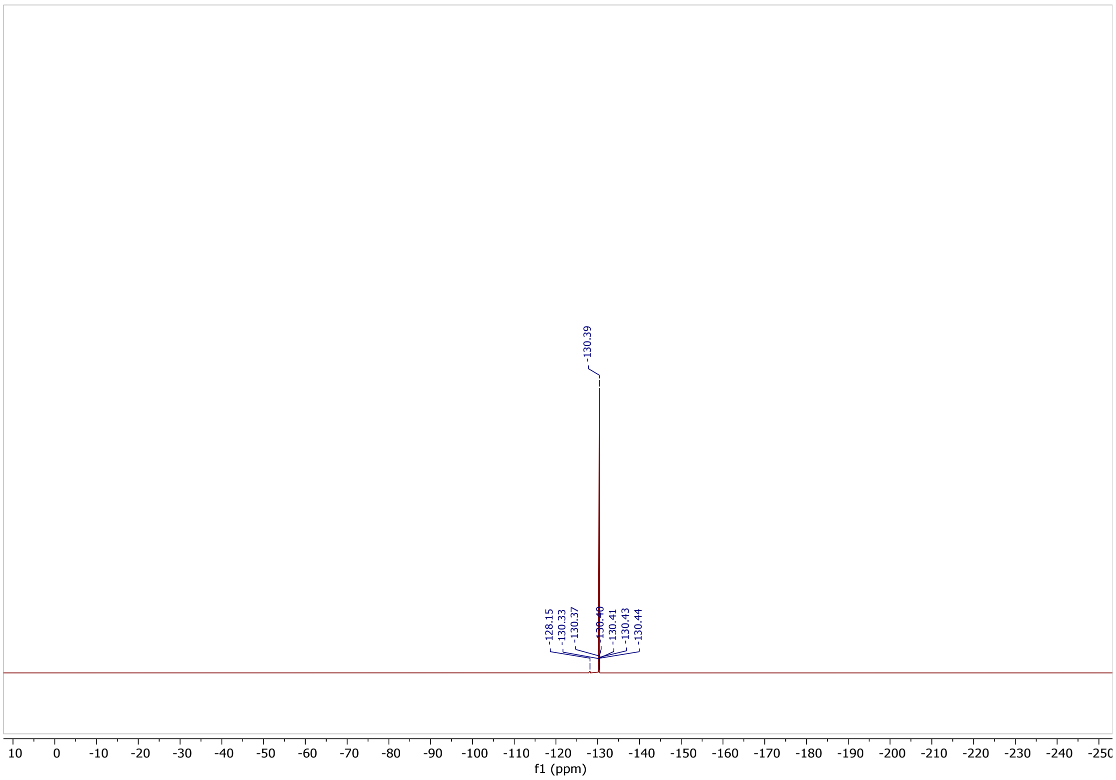

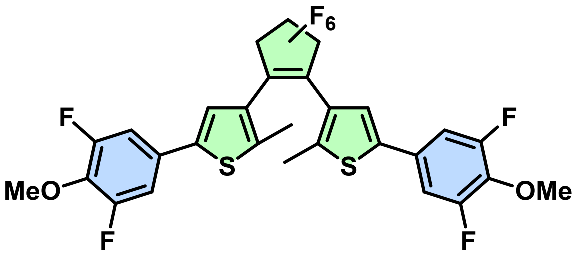

Following General Procedure A, 235 mg DAE-Br_2 (1.0 eq.), 302 mg 2-(3,5-difluoro-4-methoxyphenyl)-4,4,5,5-tetramethyl-1,3,2-dioxaborolane (2.5 eq.), 237 mg K_3PO_4 (2.5 eq.) and 17.4 mg G3 SPhos Pd (0.05 eq.) were used. The resulting crude product was purified *via* column chromatography using a gradient from petroleum ether to petroleum ether/ ethyl acetate mixture (200:1 *v*:*v*) to give 165.2 mg of DAE-3 as a light blue solid (yield 57%). ^{1}H NMR (400 MHz, $CDCl_3$): δ=7.17 (s, 2H), 7.02 (s, 2H), 7.05 (s, 2H), 4.02 (s, 6H), 1.96 (s, 6H) ppm. ^{13}C NMR (100 MHz, $CDCl_3$): δ=156.8 (o), 155.2 (o), 142.1 (o), 139.9 (o), 136.3 (o), 128.5 (o), 126.1 (o), 123.2 (+), 109.7 (+), 62.1 (+), 14.7 (+) ppm. IR (ATR): $\tilde{\nu}$ = 2957, 2843, 1574, 1515, 1476, 1432, 1338, 1267, 1246, 1190, 1108, 1027, 980, 892, 832, 753, 659, 564, 531 cm^{-1}. HRMS (APCI): calc.: 653.0661 [M+H$^+$], found: 653.0653 [M+H$^+$].

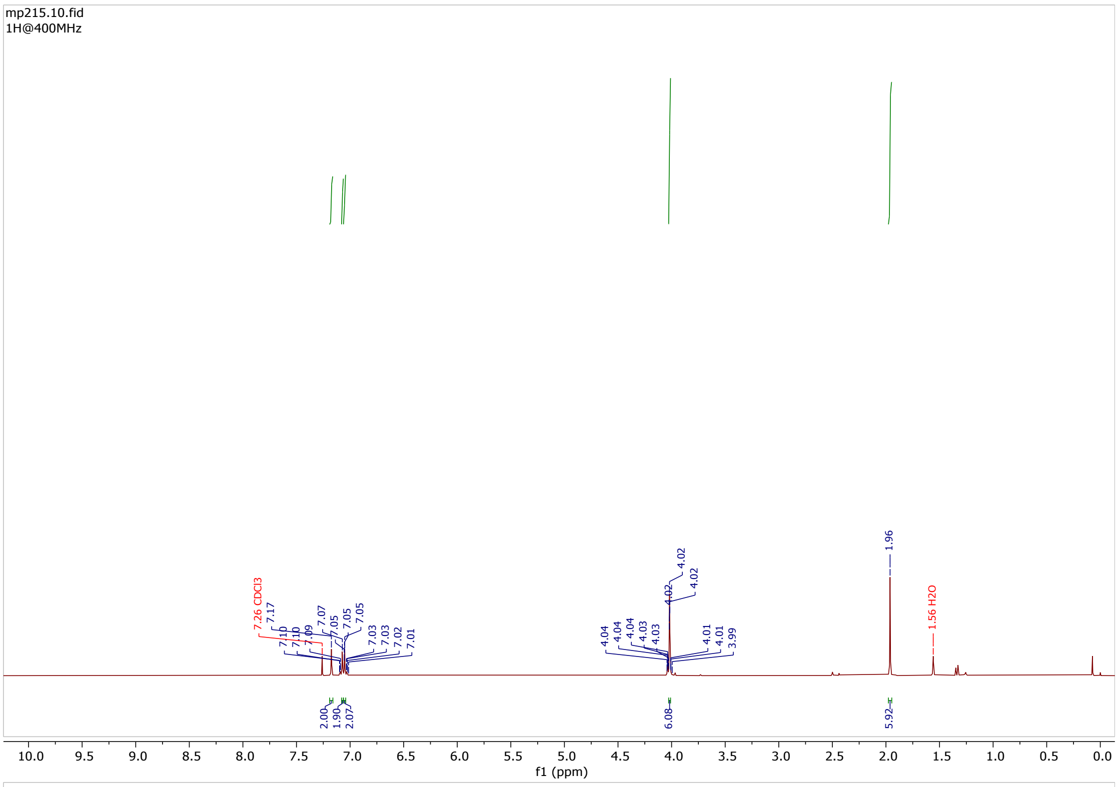

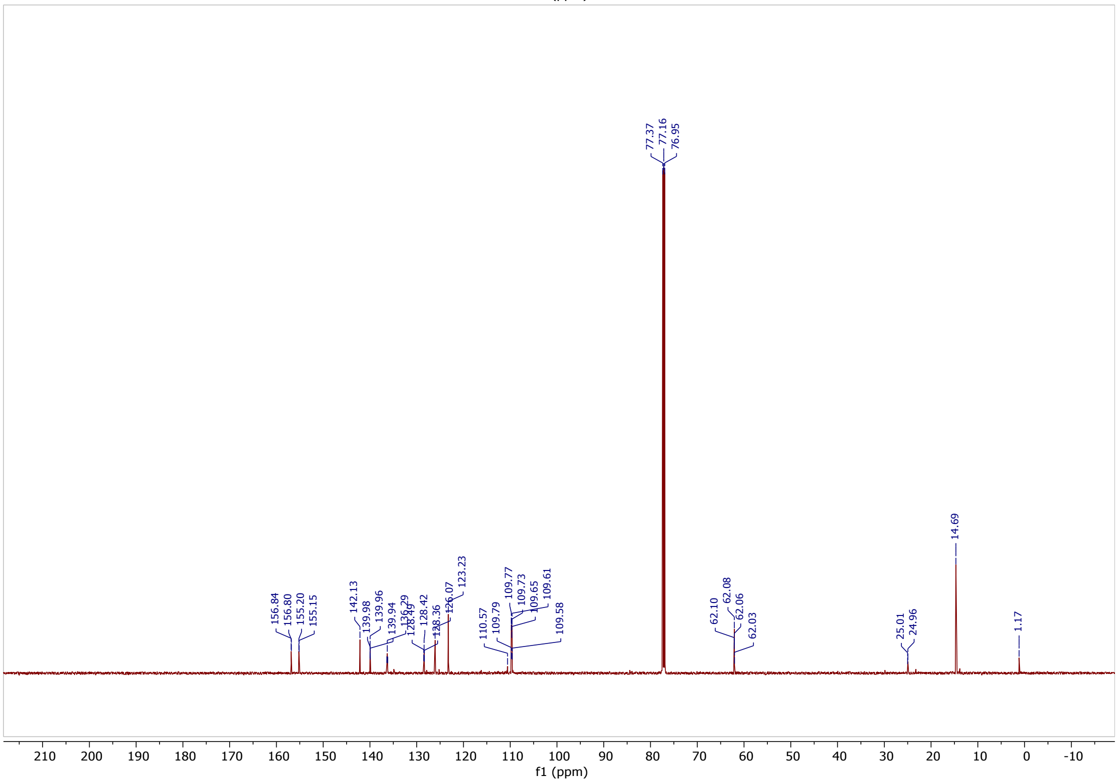

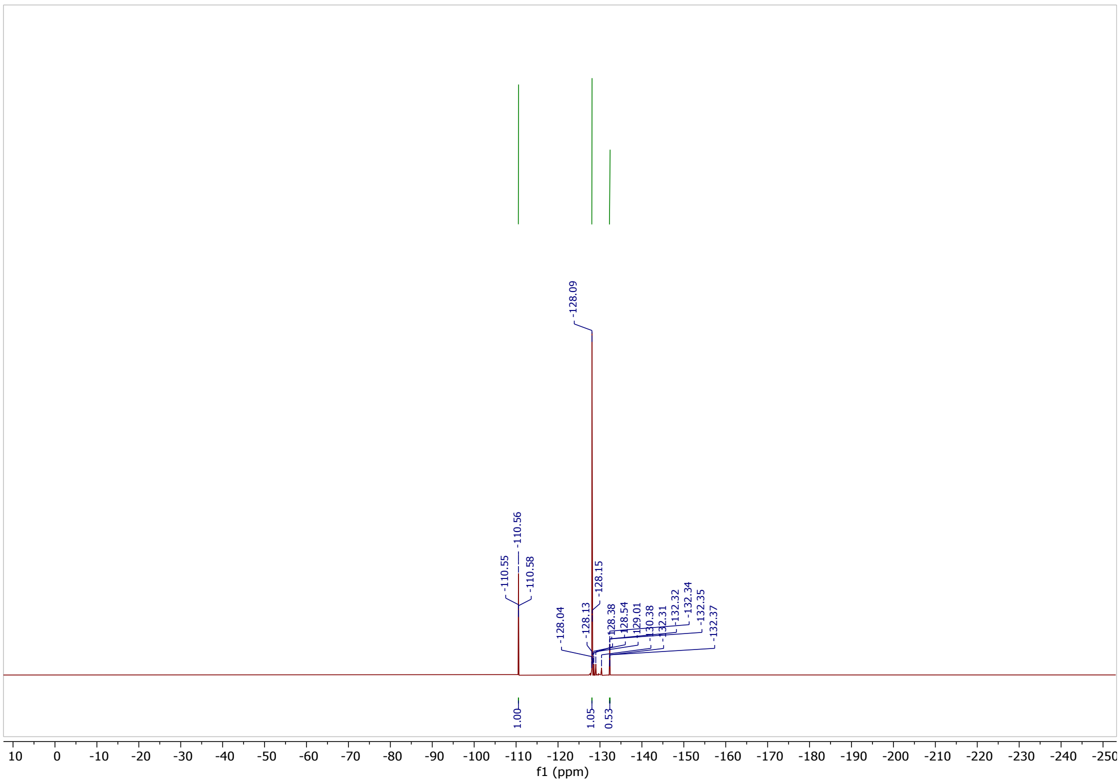
-128.09
-110.56
-110.55
-110.58
-128.15
-128.04
-128.13
-128.38
-128.54
-129.01
-130.38
-132.31
-132.32
-132.34
-132.35
-132.37
1.00
1.05
0.53
f1 (ppm)

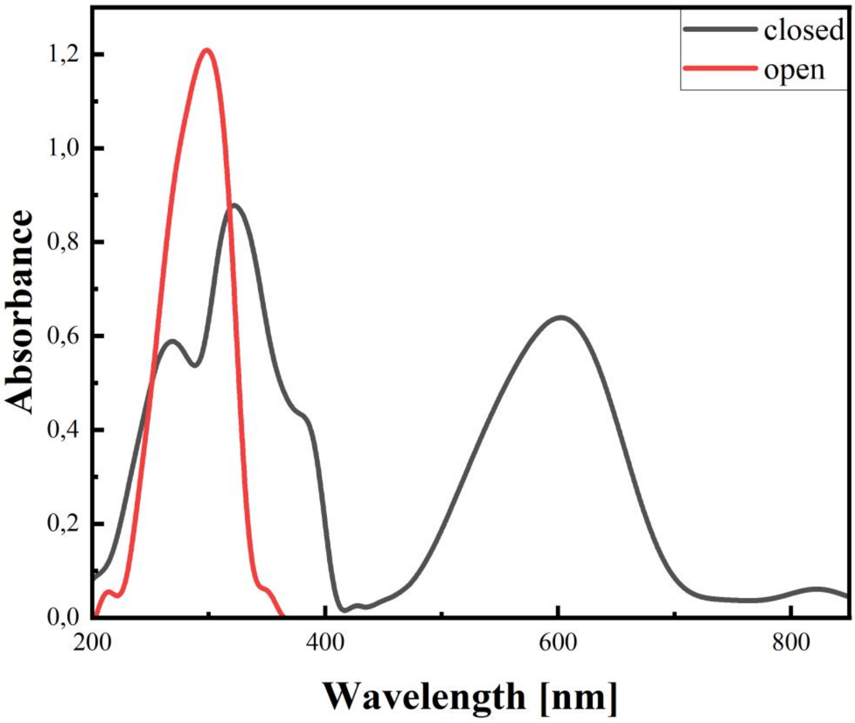
closed
open
Absorbance
Wavelength [nm]

Synthesis of DAE-5

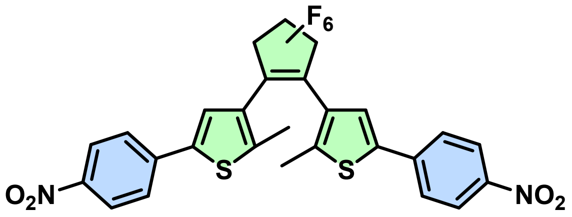

Following General Procedure A, 218.5 mg DAE-Br_2 (1.0 eq.), 173 mg (4-nitrophenyl)boronic acid (2.5 eq.), 220 mg K_3PO_4 (2.5 eq.) and 16.2 mg G3 SPhos Pd (0.05 eq.) were used. The resulting crude product was purified *via* column chromatography using petroleum ether/ ethyl acetate mixture (10:1 *v*:*v*) to give 144.3 mg of DAE-4 as a blue solid (yield 57%). ^{1}H NMR (400 MHz, $CDCl_3$): δ=8.26 (d, J = 8.8 Hz, 4H), 7.69 (d, J = 9.0 Hz, 4H), 7.44 (s, 2H), 2.03 (s, 6H) ppm. ^{13}C NMR (100 MHz, $CDCl_3$): δ=147.2 (o), 144.2 (o), 139.9 (o), 139.3 (o), 128.5 (+), 126.6 (o), 126.0 (+), 125.1 (+), 124.7 (+), 110.7 (+), 14.9 (+) ppm. IR (ATR): $\tilde{\nu}$ = 2920, 2850, 1592, 1510, 1338, 1263, 1181, 1105, 1051, 986, 887, 847, 750, 720, 688, 536, 476 cm^{-1}. HRMS (APCI): calc.: 610.0456 [M$^+$], found: 610.0444 [M$^+$].
Spectroscopic data are in agreement with those reported in the literature.[5]

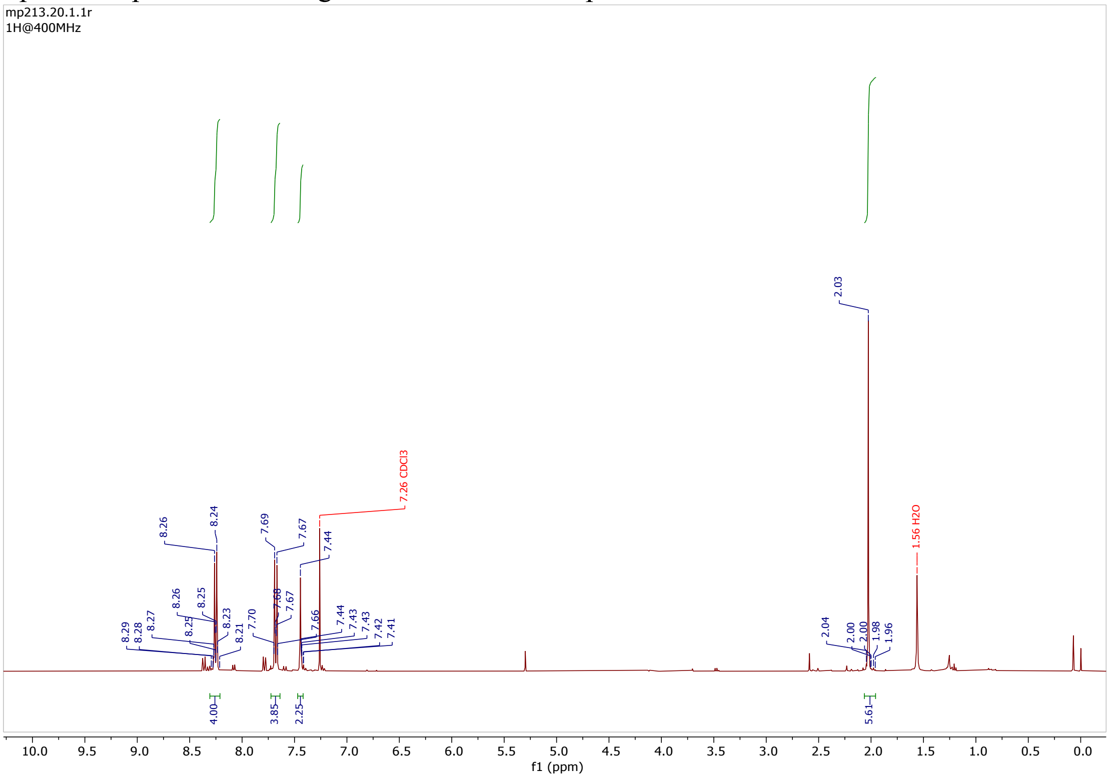

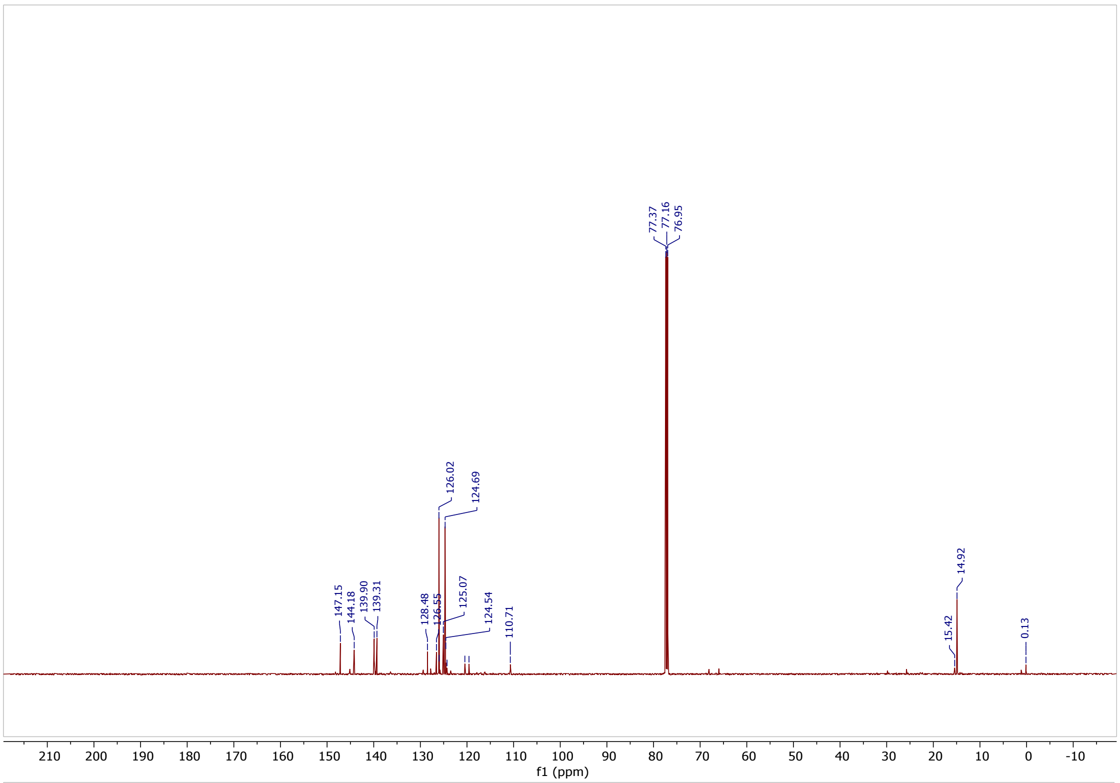
77.37
77.16
76.95
126.02
124.69
147.15
144.18
139.90
139.31
128.48
126.55
125.07
124.54
110.71
14.92
15.42
0.13
210 200 190 180 170 160 150 140 130 120 110 100 90 80 70 60 50 40 30 20 10 0 -10
f1 (ppm)

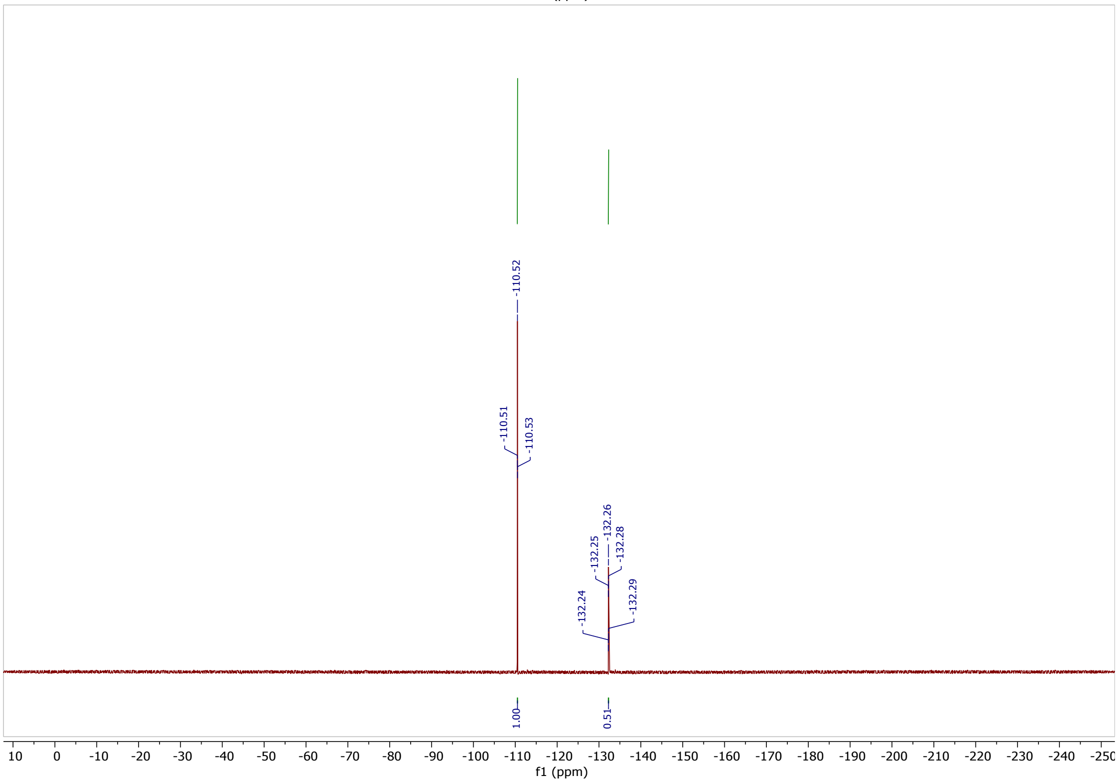
-110.52
-110.51
-110.53
-132.26
-132.25
-132.28
-132.24
-132.29
1.00
0.51
10 0 -10 -20 -30 -40 -50 -60 -70 -80 -90 -100 -110 -120 -130 -140 -150 -160 -170 -180 -190 -200 -210 -220 -230 -240 -250
f1 (ppm)

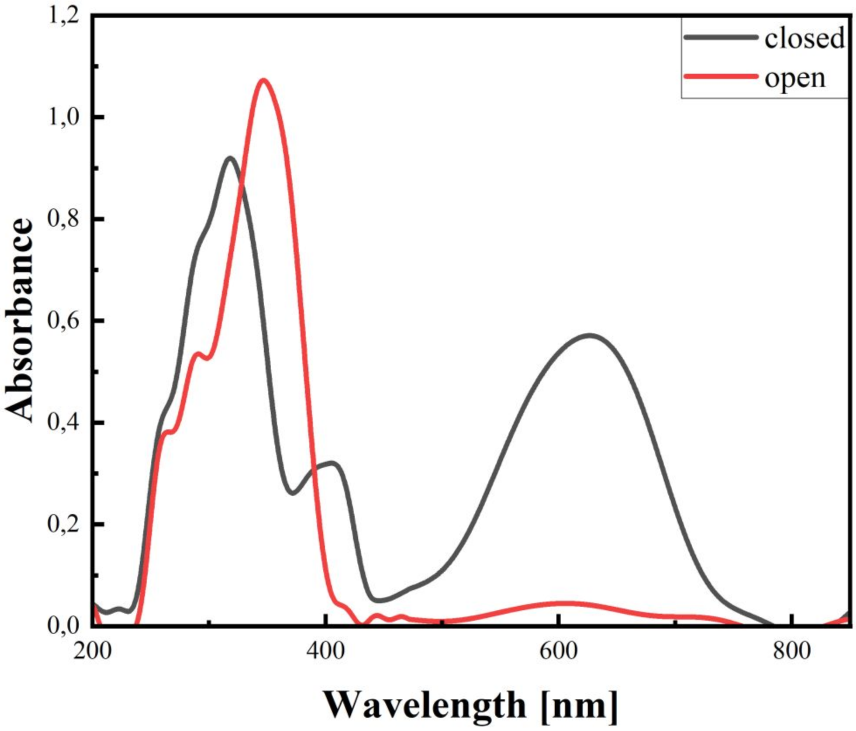

Synthesis of DAE-6

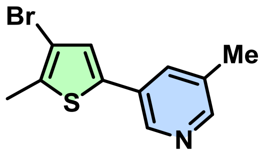

Following General Procedure D, 2 g (4-bromo-5-methylthiophen-2-yl)boronic acid (1 eq.), 1.496 g 3-bromo-5-methylpyridine (1 eq.), 2.822 g Na_2CO_3 (3 eq.) and 718 mg $Pd(PPh_3)_4$ (0.07 eq.) were used. The resulting crude product was purified *via* column chromatography using a petroleum ether/ ethyl acetate mixture (4:1 *v*:*v*) to give 1.705 g of DAE-6 precursor as a white solid (yield 64%). ^{1}H NMR (400 MHz, $CDCl_3$): δ=8.58 (s, 1H), 8.35 (s, 1H), 7.58 (s, 1H), 7.14 (s, 1H), 2.43 (s, 3H), 2.36 (s, 3H) ppm. HRMS (ESI): calc.: 289.9610 [M+Na$^+$], found: 289.9607 [M+Na$^+$].

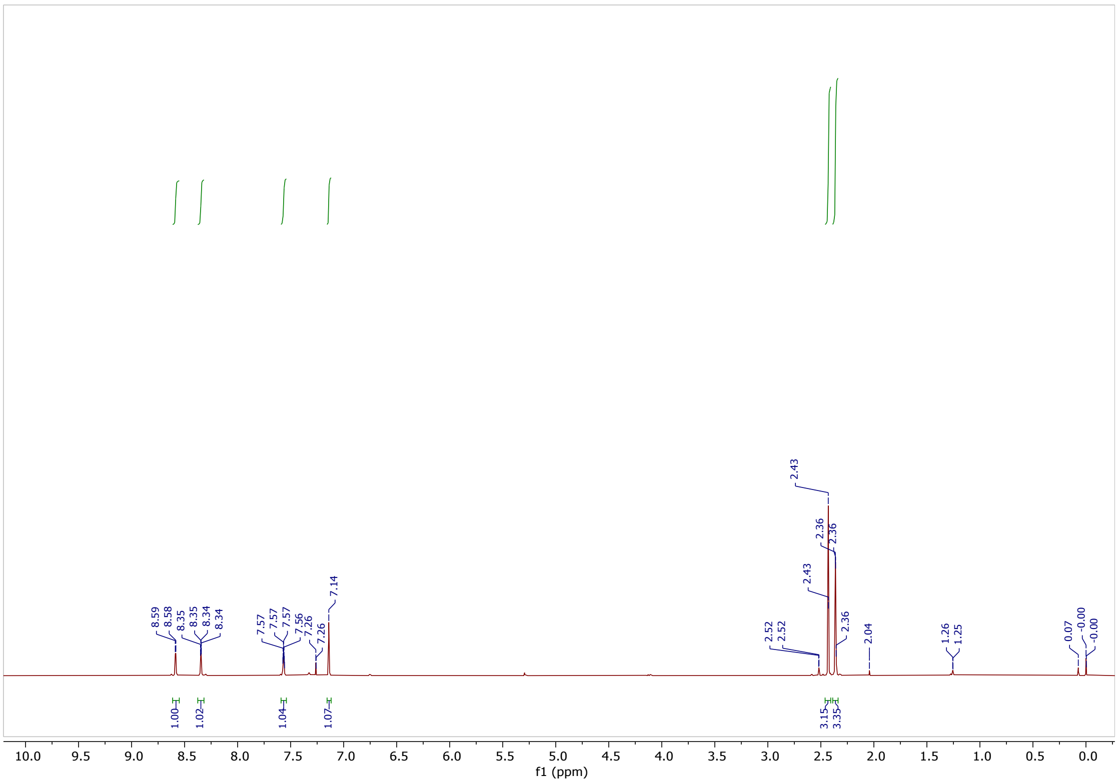

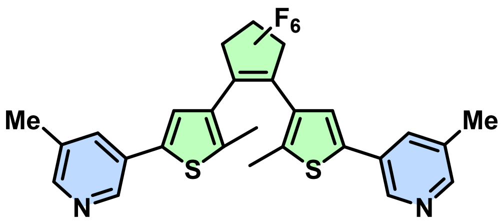

Following General Procedure B, 1.664 g 3-(4-bromo-5-methylthiophen-2-yl)-5-methylpyridine (1.0 eq.), 2.77 mL *n*-BuLi (2.8 M in hexane, 1.25 eq.) and 416 µl octafluorocyclopentene (0.5 eq.) reacted in 60 mL of dry THF. The crude product was purified *via* column chromatography using a petroleum ether/ ethyl acetate (1:2 *v*:*v*) mixture to give 651 mg of DAE-6 as a off-white solid (yield 39 %). ^{1}H NMR (400 MHz, $CDCl_3$): δ=8.6 (d, J = 2.19 Hz, 2H), 8.37 (d, J = 1.98 Hz, 2H), 7.63-7.62 (m, 2H), 7.30 (s, 2H), 2.38 (s, 6H), 2.00 (s, 6H) ppm. ^{13}C NMR (100 MHz, $CDCl_3$): δ=205.3 (o), 149.5 (+), 143.6 (+), 142.5 (o), 138.8 (o), 133.6 (o), 132.9 (+), 128.6 (o), 125.7 (o), 123.9 (+), 17.3 (+), 13.7 (+) ppm. IR (ATR): $\tilde{\nu}$ = 3032, 1595, 1550, 1490, 1431, 1329, 1267, 1186, 1094, 1055, 979, 871, 838, 818, 739, 704, 532 cm^{-1}. HRMS (APCI): calc.: 551.1045 [$M+H^+$], found: 551.1062 [$M+H^+$].

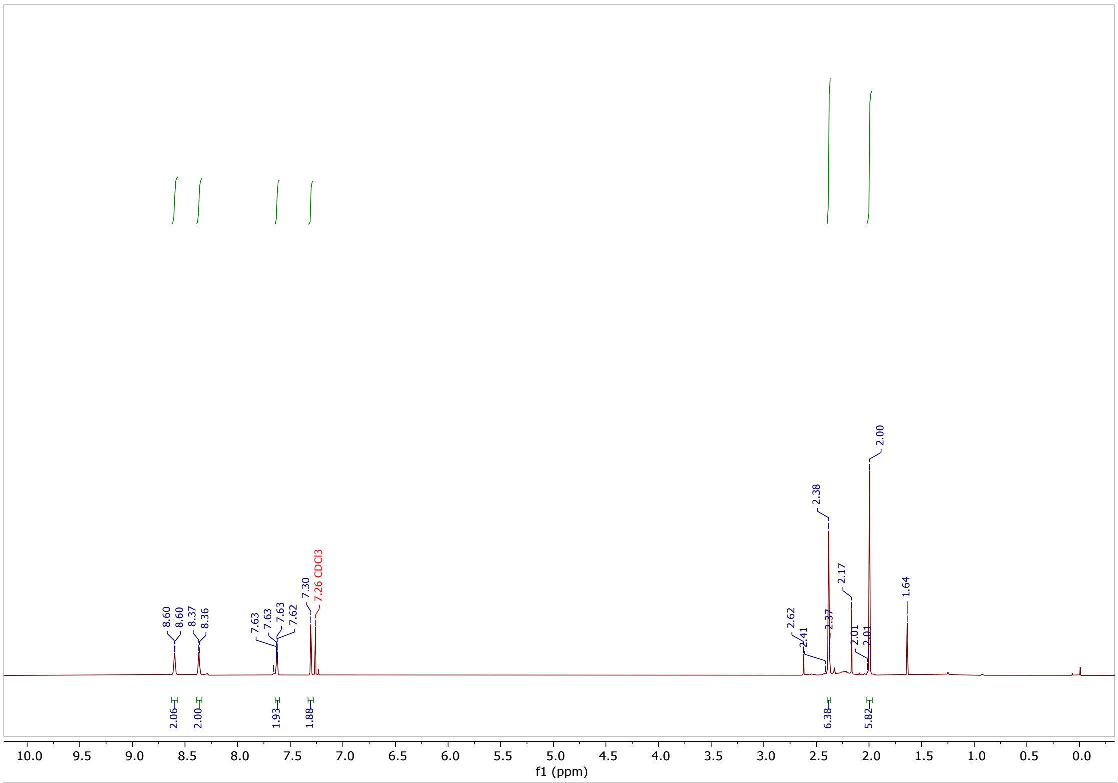
8.60
8.60
8.37
8.36
7.63
7.63
7.63
7.62
7.30
7.26 CDCl3
2.62
2.41
2.38
2.37
2.17
2.01
2.01
2.00
1.64
2.06
2.00
1.93
1.88
6.38
5.82
f1 (ppm)

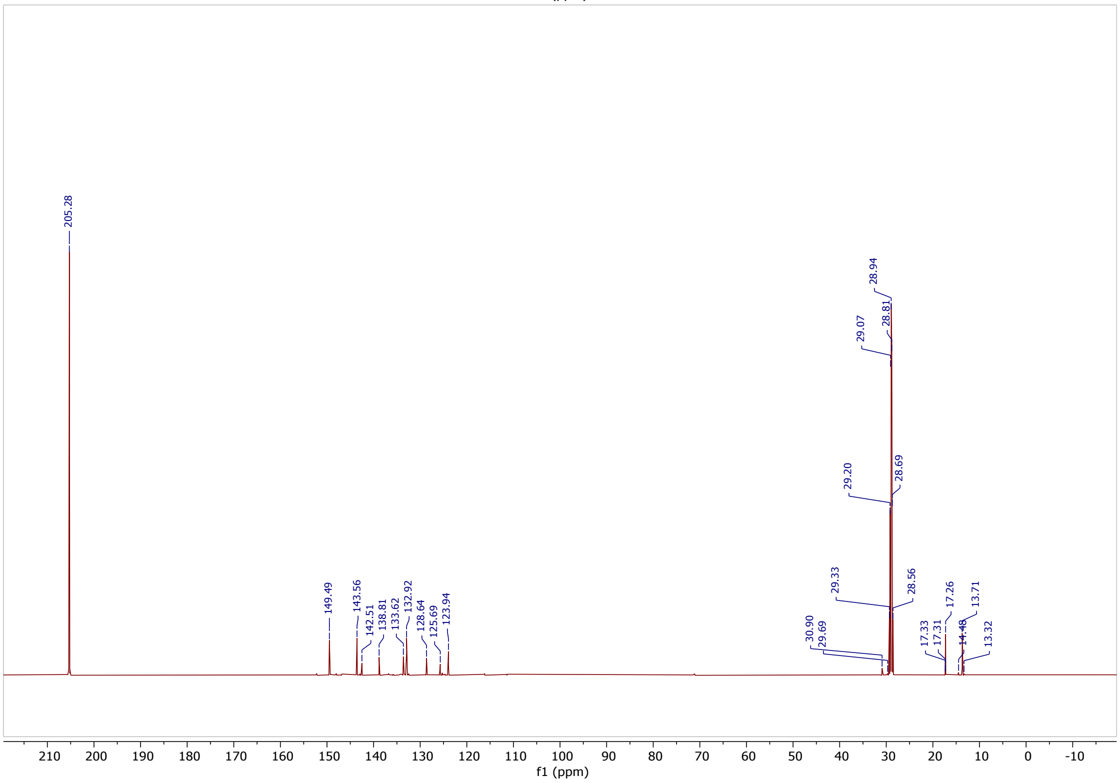
205.28
149.49
143.56
142.51
138.81
133.62
132.92
128.64
125.69
123.94
30.90
29.69
29.33
29.20
29.07
28.94
28.81
28.69
28.56
17.33
17.31
17.26
14.48
13.71
13.32
f1 (ppm)

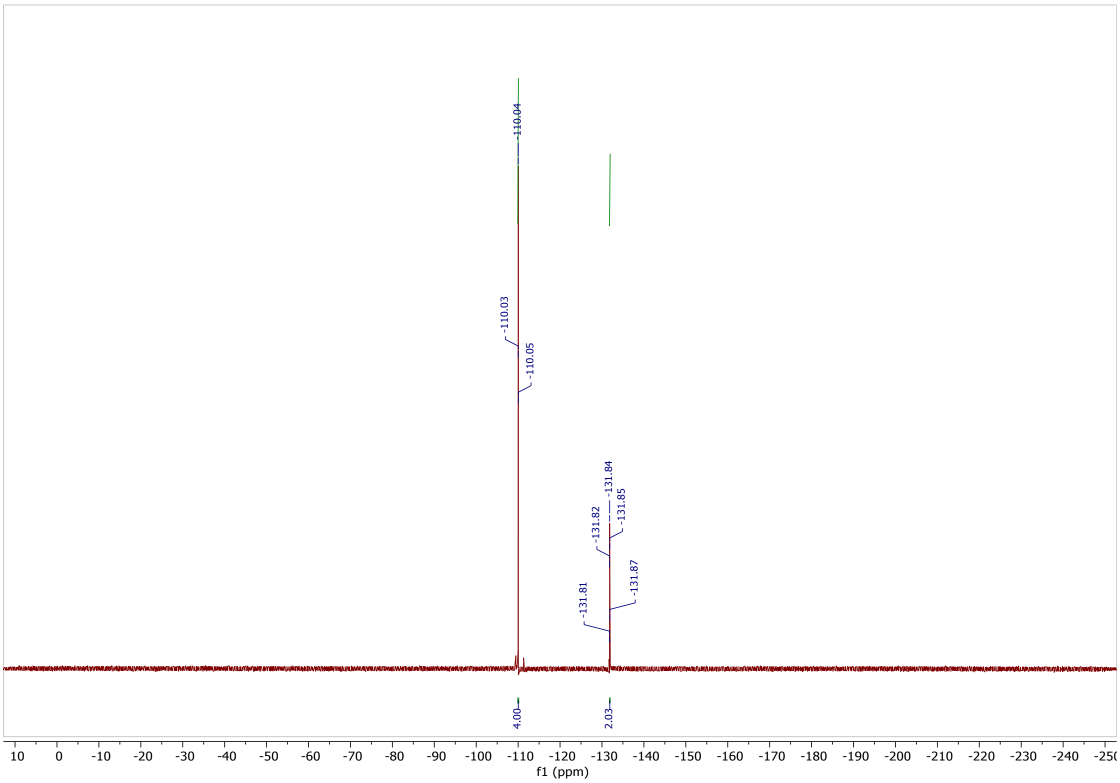

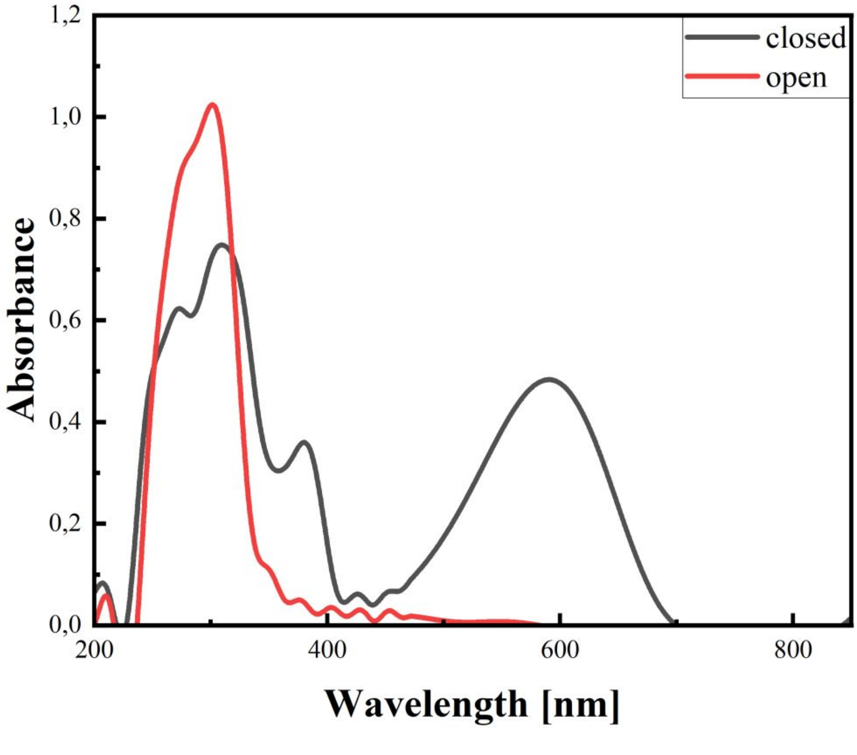

Synthesis of DAE-7

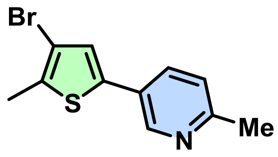

Following General Procedure D, 3 g (4-bromo-5-methylthiophen-2-yl)boronic acid (1 eq.), 2.468 g 5-bromo-2-methylpyridine (1.1 eq.), 4.232 g Na_2CO_3 (3 eq.) and 769 mg $Pd(PPh_3)_4$ (0.05 eq.) were used. The resulting crude product was purified *via* column chromatography using a gradient of petroleum ether/ ethyl acetate (5:1 to 2:1 *v*:*v*) mixture to give 3 g of DAE-7 precursor as a off-white solid (yield 85%). ^{1}H NMR (400 MHz, $CDCl_3$): δ=8.65 (d, J = 2.40, 1H), 7.65 (dd, J = 8.09, 2.47 Hz, 1H), 7.15 (d, J = 8.06 Hz, 1H), 7.10 (s, 1H), 2.56 (s, 3H), 2.42 (s, 3H) ppm. HRMS (ESI): calc.: 267.9790 [M+H$^+$], found: 267.9791 [M+H$^+$].
Spectroscopic data are in agreement with those reported in the literature.[6]

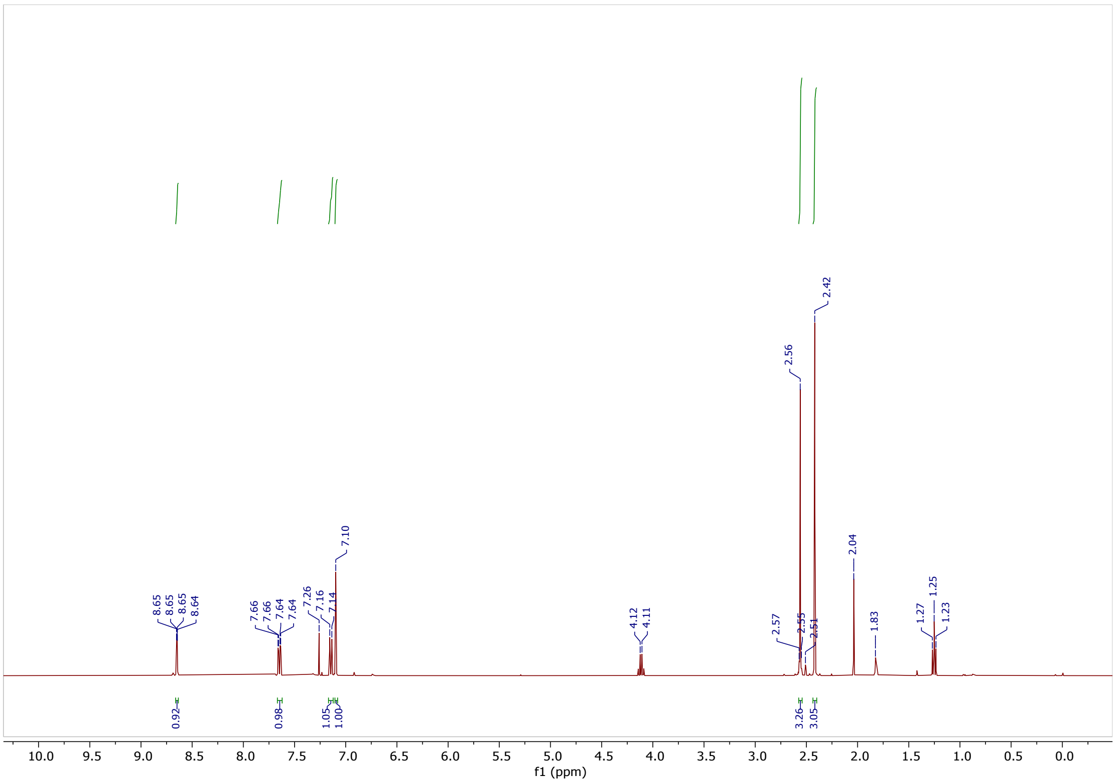

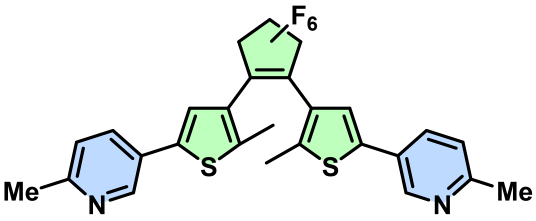

Following General Procedure B, 2.61 g 5-(4-bromo-5-methylthiophen-2-yl)-2-methylpyridine (1.0 eq.), 4.34 mL *n*-BuLi (2.8 M in hexane, 1.25 eq.) and 653 µl octafluorocyclopentene (0.5 eq.) reacted in 60 mL of dry THF. The crude product was purified *via* column chromatography using a gradient of petroleum ether/ ethyl acetate (2:1 to 1:2 *v*:*v*) mixture to give 620 mg of DAE-7 as a beige solid (yield 24 %). ^{1}H NMR (400 MHz, $CDCl_3$): δ=8.67 (d, J = 2.42 Hz, 2H), 7.71 (dd, J = 8.10, 2.44 Hz, 2H), 7.26 (s, 2H), 7.18 (d, J = 8.13 Hz, 2H), 2.58 (s, 6H), 1.99 (s, 6H) ppm. ^{13}C NMR (100 MHz, $CDCl_3$): δ=157.8 (o), 145.7 (+), 142.0 (o), 138.6 (o), 133.4 (+), 126.7 (o), 126.0 (o), 123.5 (+), 123.1 (+), 24.0 (+), 14.6 (+) ppm. IR (ATR): $\tilde{\nu}$ = 1566, 1466, 1333, 1295, 1261, 1184, 1093, 1053, 984, 886, 818, 727, 671, 531, 482 cm^{-1}. HRMS (APCI): calc.: 551.1045 [M+H$^+$], found: 551.1056 [M+H$^+$].

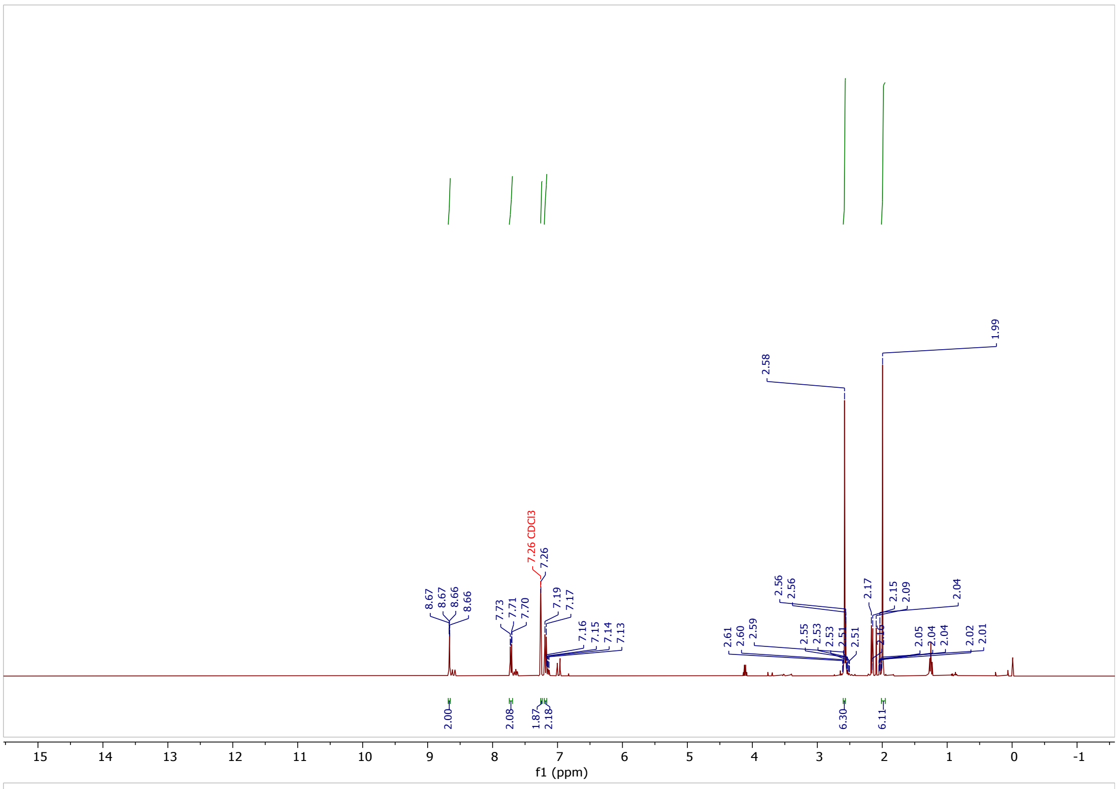

7.26 CDCl3
f1 (ppm)

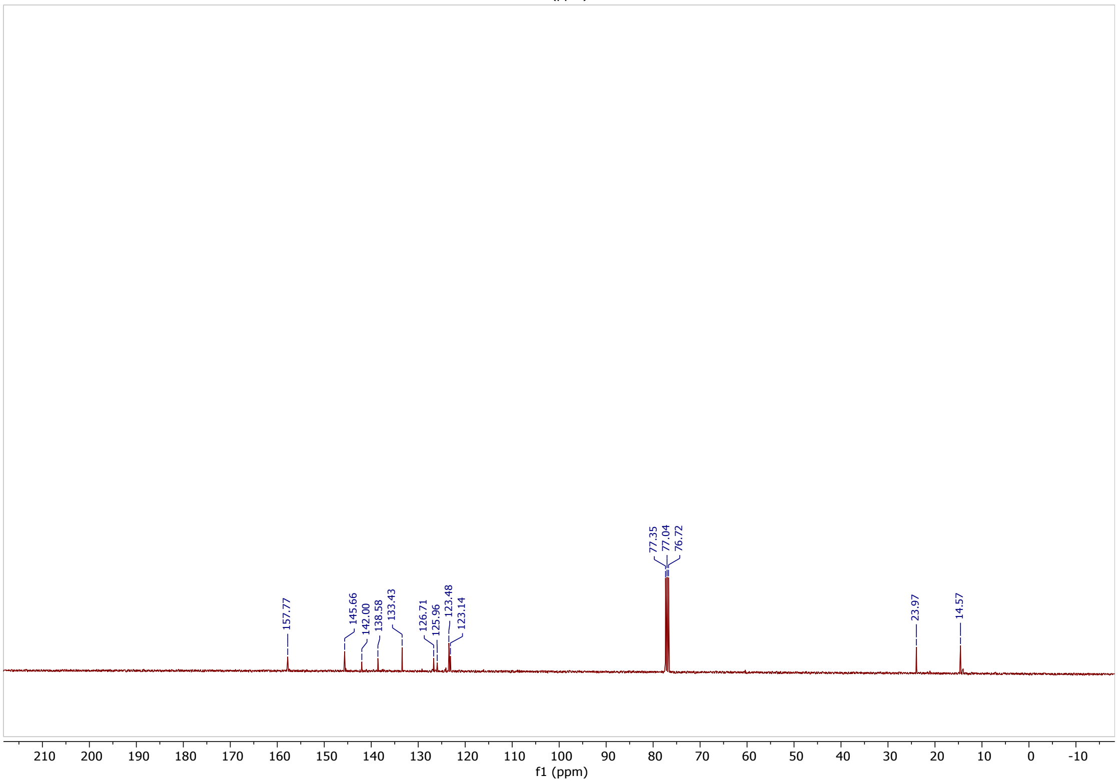

157.77
145.66
142.00
138.58
133.43
126.71
125.96
123.48
123.14
77.35
77.04
76.72
23.97
14.57
f1 (ppm)

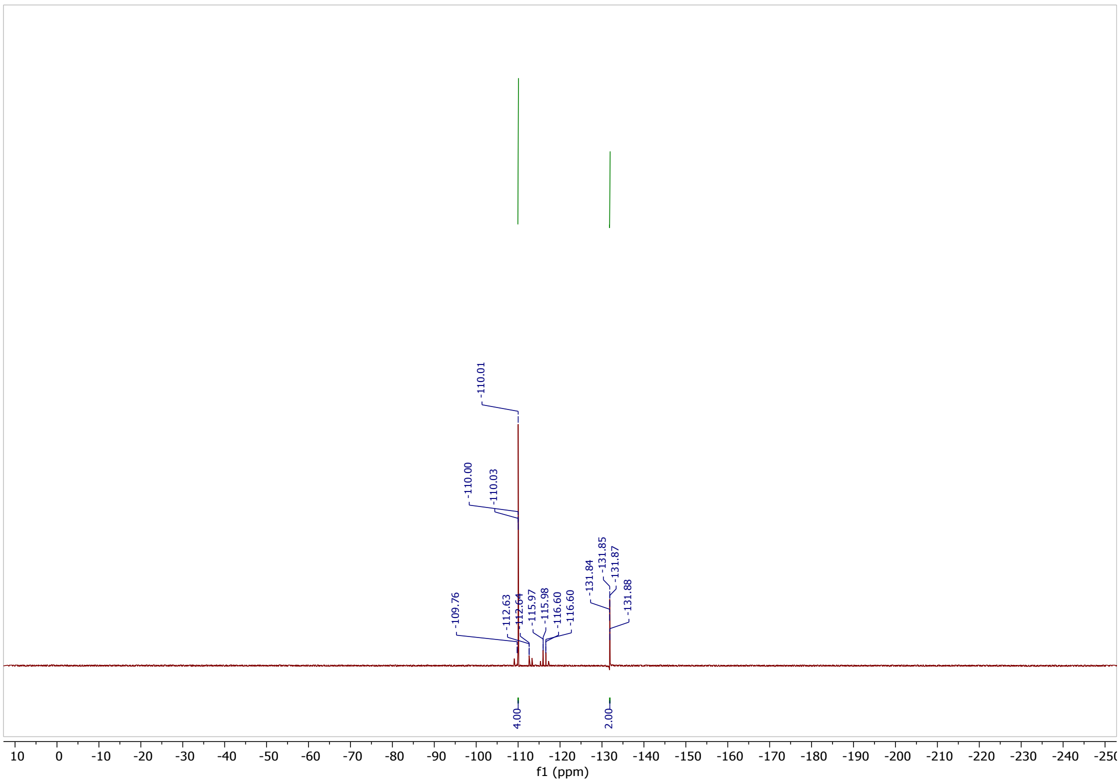

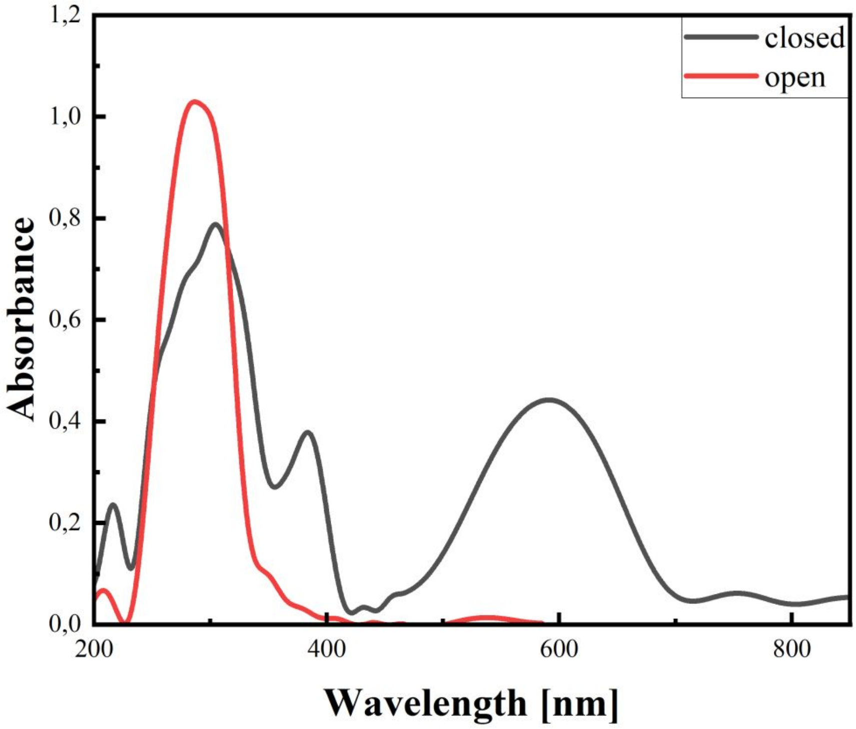

Synthesis of DAE-8

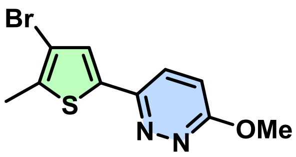

Following General Procedure D, 2 g (4-bromo-5-methylthiophen-2-yl)boronic acid (1 eq.), 1.789 g 3-bromo-6-methoxypyridazine (1.1 eq.), 3.566 g K_2CO_3 (3 eq.) and 994 mg $Pd(PPh_3)_4$ (0.1 eq.) were used. The resulting crude product was purified *via* column chromatography using a gradient of petroleum ether/ ethyl acetate (20:1 to 5:1 *v*:*v*) mixture to give 1.743 g of DAE-8 precursor as an off-white solid (yield 72%). ^{1}H NMR (400 MHz, $CDCl_3$): δ=7.59-7.55 (m, 1H), 7.30-7.23 (m, 1H), 6.97-6.92 (m, 1H), 4.12 (s, 3H), 2.42 (s, 3H) ppm. HRMS (ESI): calc.: 306.9511 [M+Na$^+$], found: 306.9515 [M+Na$^+$].

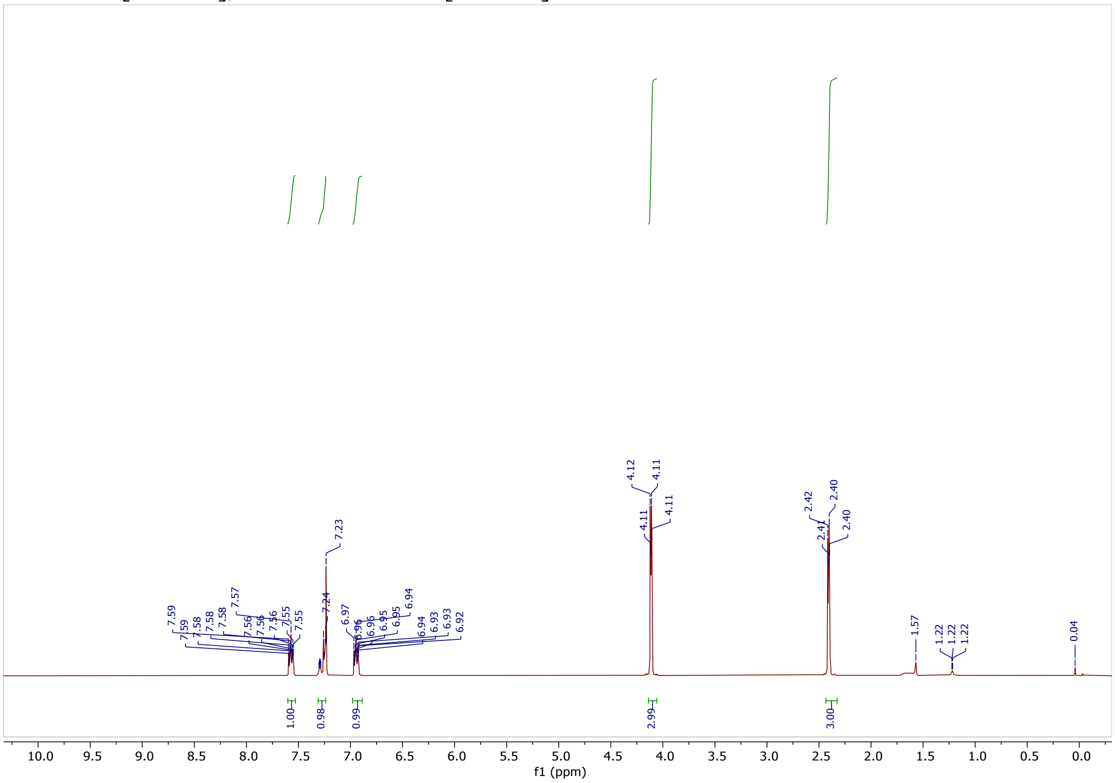

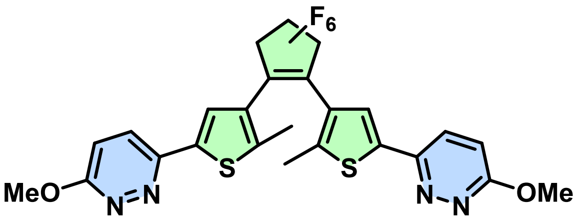

Following General Procedure B, 312 mg 3-(4-bromo-5-methylthiophen-2-yl)-6-methoxypyridazine (1.0 eq.), 488 µl *n*-BuLi (2.8 M in hexane, 1.25 eq.) and 74 µl octafluorocyclopentene (0.5 eq.) reacted in 40 mL of dry THF. The crude product was purified *via* column chromatography using a petroleum ether/ ethyl acetate (3:1 *v*:*v*) mixture to give 34 mg of DAE-7 as a light blue solid (yield 11 %). ^{1}H NMR (400 MHz, $CDCl_3$): δ=7.68 (d, J = 9.25 Hz, 2H), 7.46 (s, 2H), 7.00 (d, J = 9.22 Hz, 2H), 4.15 (s, 6H), 2.03 (s, 6H) ppm. ^{13}C NMR (100 MHz, $CDCl_3$): δ=171.2 (o), 164.2 (o), 150.2 (o), 144.9 (o), 139.1 (o), 125.4 (+), 124.2 (+), 118.9 (+), 14.9 (+), 14.1 (+) ppm. IR (ATR): $\tilde{\nu}$ = 2951, 1594, 1557, 1459, 1408, 1343, 1271, 1189, 1108, 1054, 1010, 986, 888, 837, 539 cm^{-1}. HRMS (APCI): calc.: 585.0848 [M+H$^+$], found: 585.0861 [M+H$^+$].

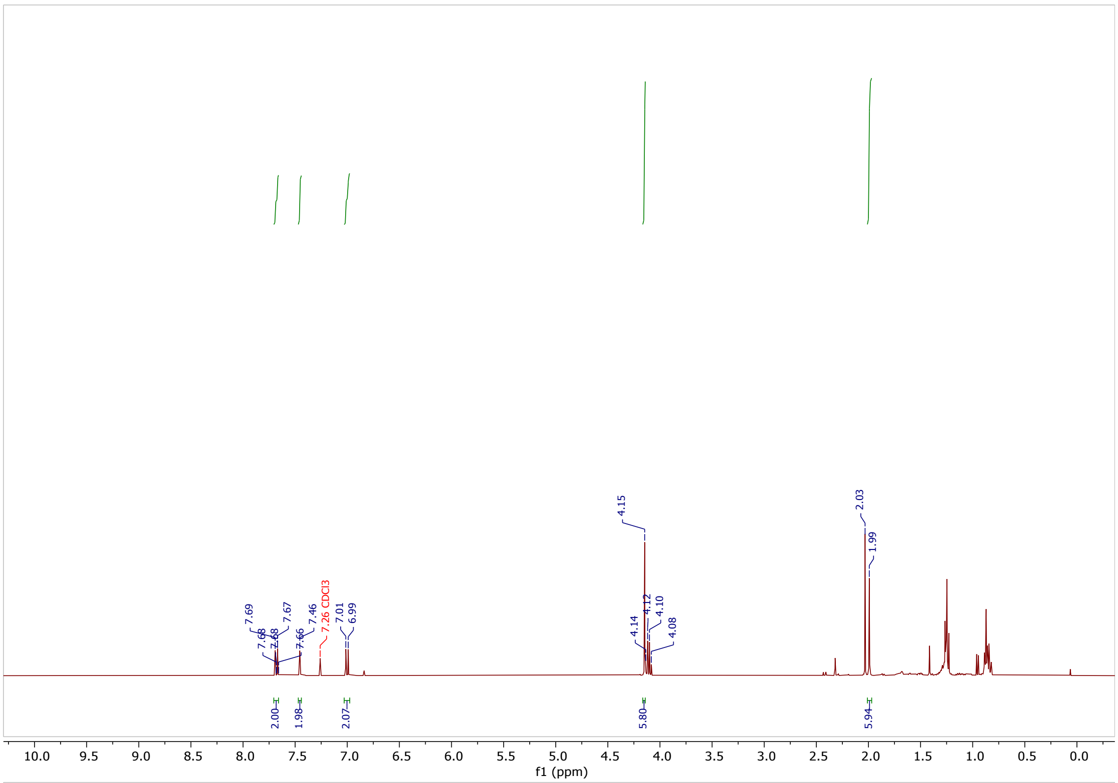
7.69
7.68
7.68
7.67
7.66
7.46
7.26 CDCl3
7.01
6.99
4.15
4.14
4.12
4.10
4.08
2.03
1.99
2.00
1.98
2.07
5.80
5.94
10.0 9.5 9.0 8.5 8.0 7.5 7.0 6.5 6.0 5.5 5.0 4.5 4.0 3.5 3.0 2.5 2.0 1.5 1.0 0.5 0.0
f1 (ppm)

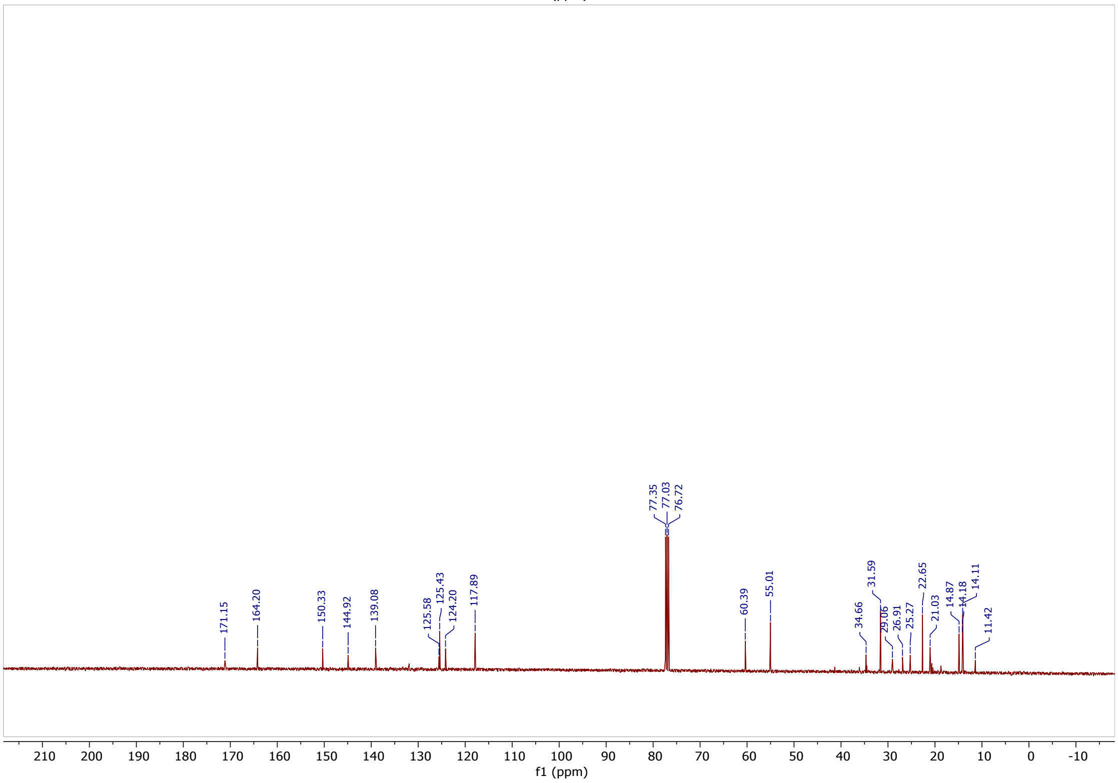
171.15
164.20
150.33
144.92
139.08
125.58
125.43
124.20
117.89
77.35
77.03
76.72
60.39
55.01
34.66
31.59
29.06
26.91
25.27
22.65
21.03
14.87
14.18
14.11
11.42
210 200 190 180 170 160 150 140 130 120 110 100 90 80 70 60 50 40 30 20 10 0 -10
f1 (ppm)

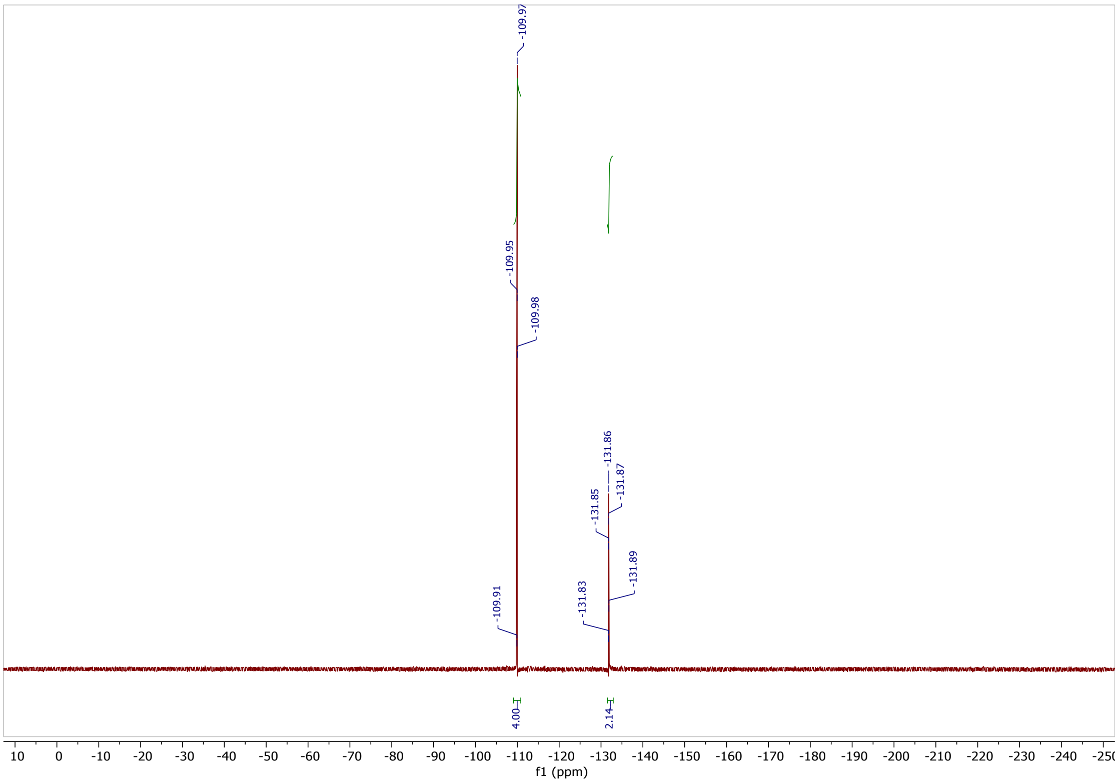
-109.97
-109.95
-109.98
-109.91
-131.86
-131.87
-131.85
-131.89
-131.83
4.00
2.14
10 0 -10 -20 -30 -40 -50 -60 -70 -80 -90 -100 -110 -120 -130 -140 -150 -160 -170 -180 -190 -200 -210 -220 -230 -240 -250
f1 (ppm)

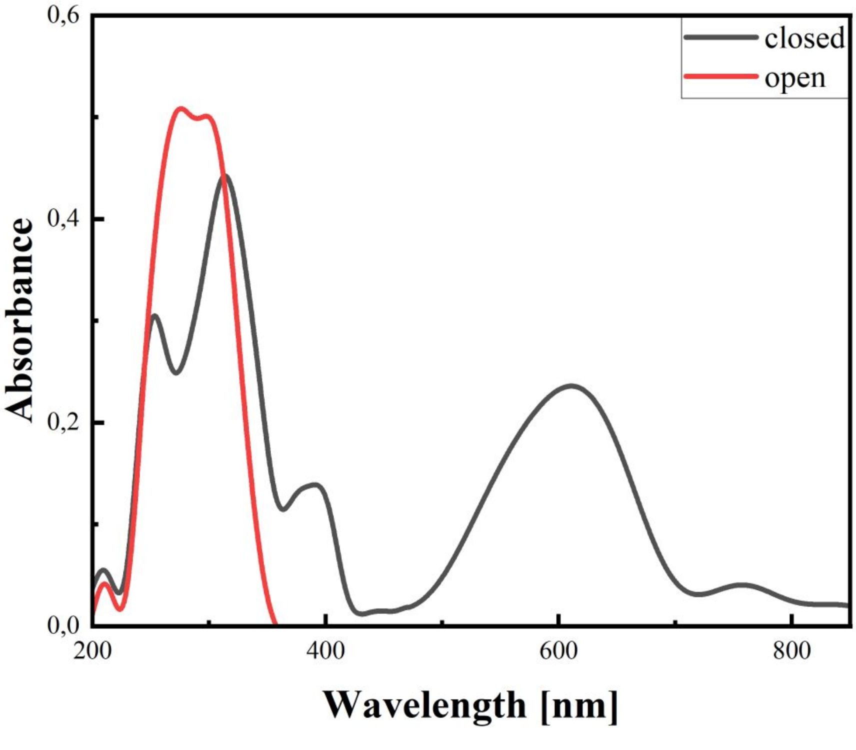
closed
open
Absorbance
0,0
0,2
0,4
0,6
200
400
600
800
Wavelength [nm]

Synthesis of DAE-9

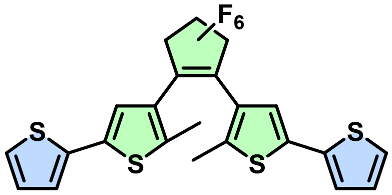

Following General Procedure A, 186.8 mg DAE-Br_2 (1.0 eq.), 169 mg potassium (thiophen-2-yl)trifluoroborate (2.5 eq.), 188 mg K_3PO_4 (2.5 eq.) and 13.9 mg G3 SPhos Pd (0.05 eq.) were used. The resulting crude product was purified *via* column chromatography using petroleum ether to give 85.6 mg of DAE-12 as an off white solid (yield 46%). ^{1}H NMR (400 MHz, $CDCl_3$): δ=7.24 (dd, J = 5.11, 1.19 Hz, 2H), 7.14 (m, 4H), 7.03 (m, 2H), 1.96 (s, 6H) ppm. ^{13}C NMR (100 MHz, $CDCl_3$): δ=140.9 (o), 136.3 (o), 135.7 (o), 128.0 (+), 125.6 (o), 125.0 (+), 124.2 (+), 122.9 (+), 14.6 (+) ppm. IR (ATR): $\tilde{\nu}$ = 2926, 1668, 1437, 1335, 1264, 1184, 1103, 1051, 983, 876, 809, 738, 689, 529, 469 cm^{-1}. HRMS (APCI): calc.: 532.9955 [M+H$^+$], found: 532.9944 [M+H$^+$].

Spectroscopic data are in agreement with those reported in the literature.[7]

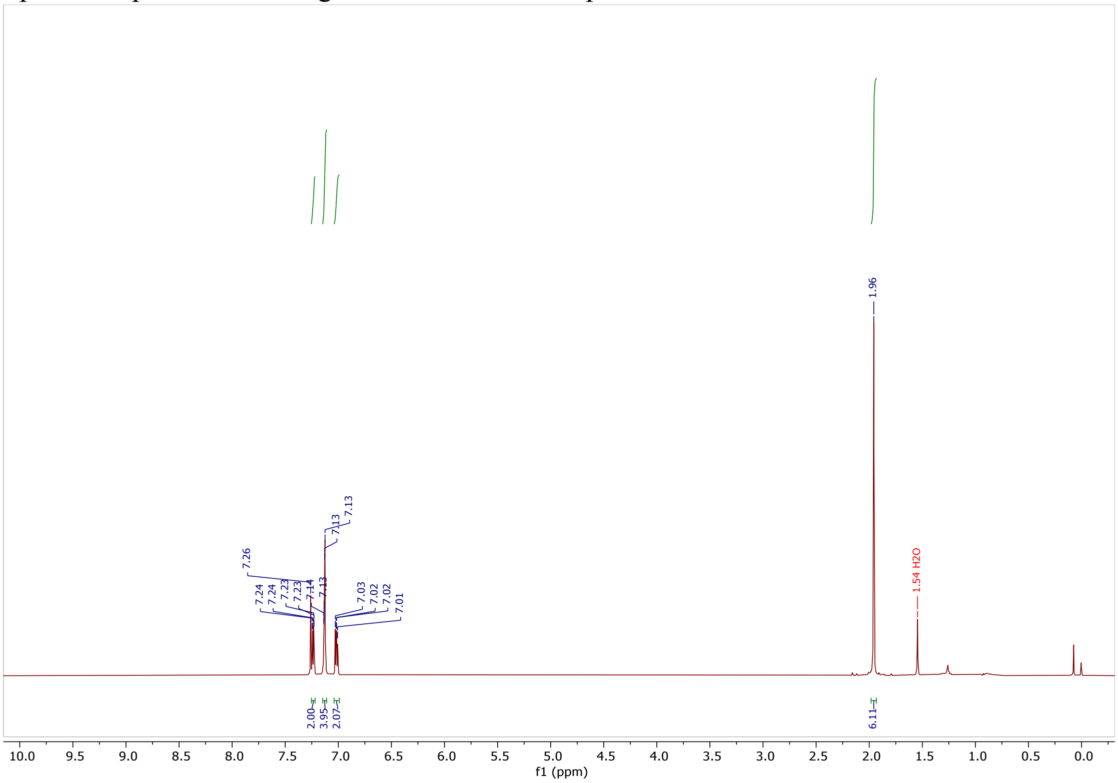

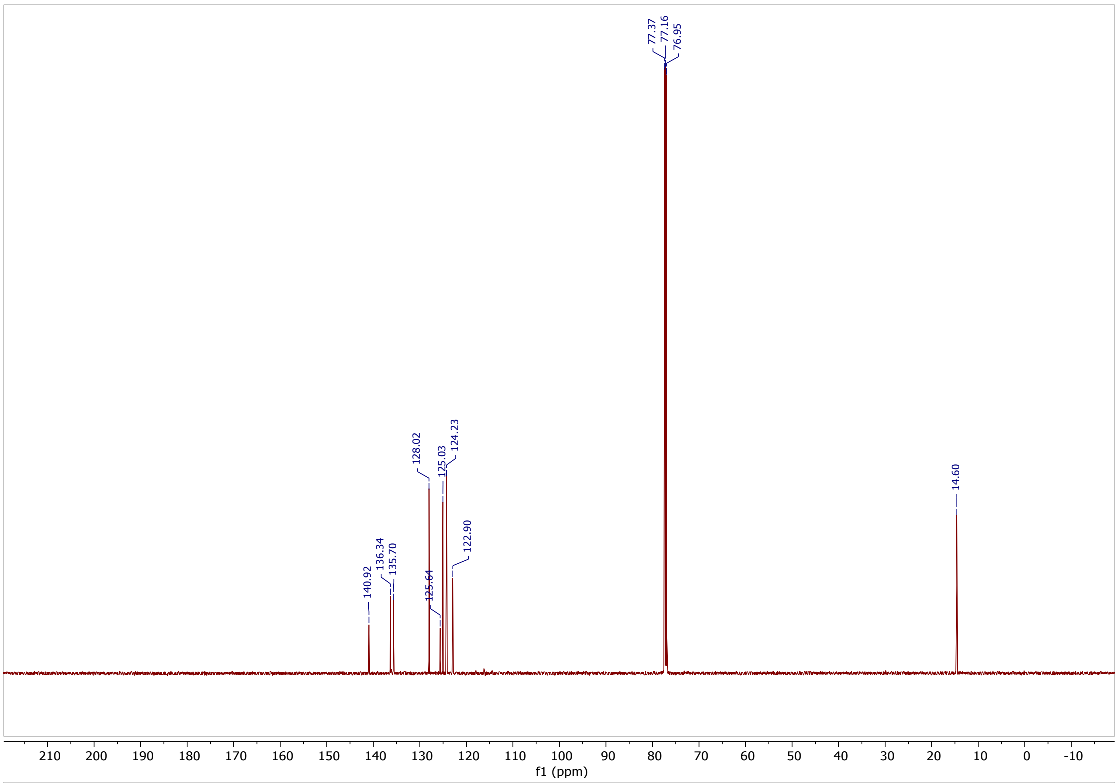
140.92
136.34
135.70
128.02
125.64
125.03
124.23
122.90
77.37
77.16
76.95
14.60
210 200 190 180 170 160 150 140 130 120 110 100 90 80 70 60 50 40 30 20 10 0 -10
f1 (ppm)

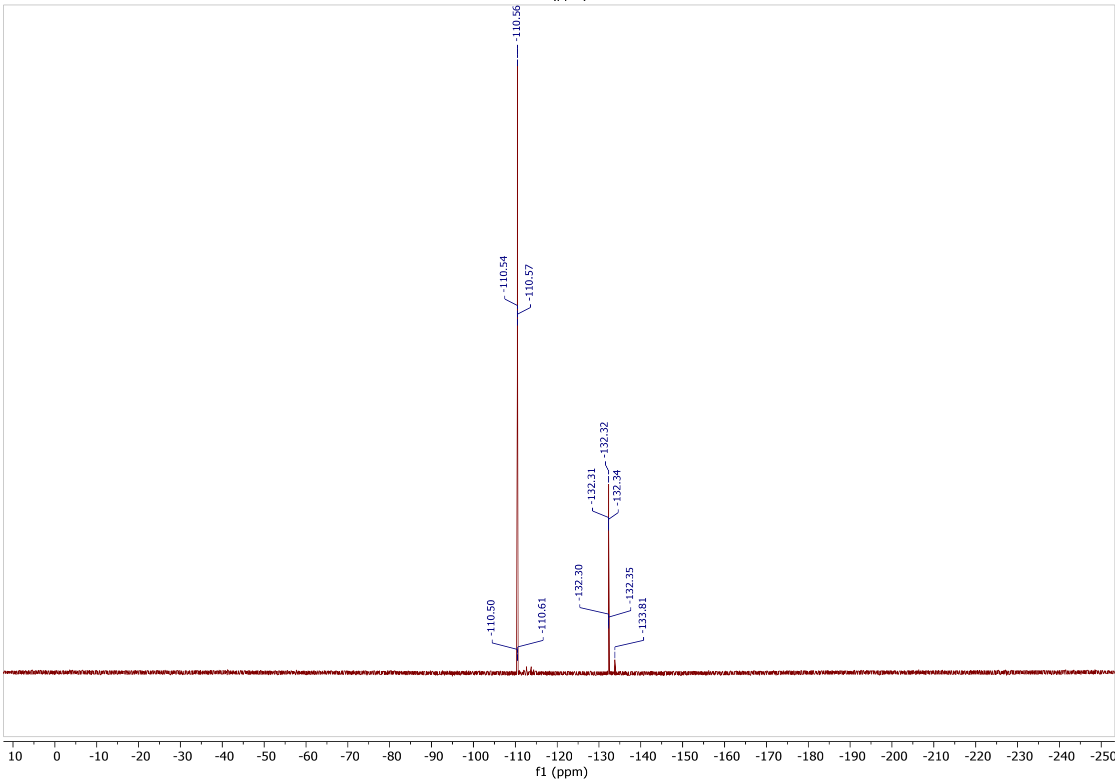
-110.56
-110.54
-110.57
-110.50
-110.61
-132.32
-132.31
-132.34
-132.30
-132.35
-133.81
10 0 -10 -20 -30 -40 -50 -60 -70 -80 -90 -100 -110 -120 -130 -140 -150 -160 -170 -180 -190 -200 -210 -220 -230 -240 -250
f1 (ppm)

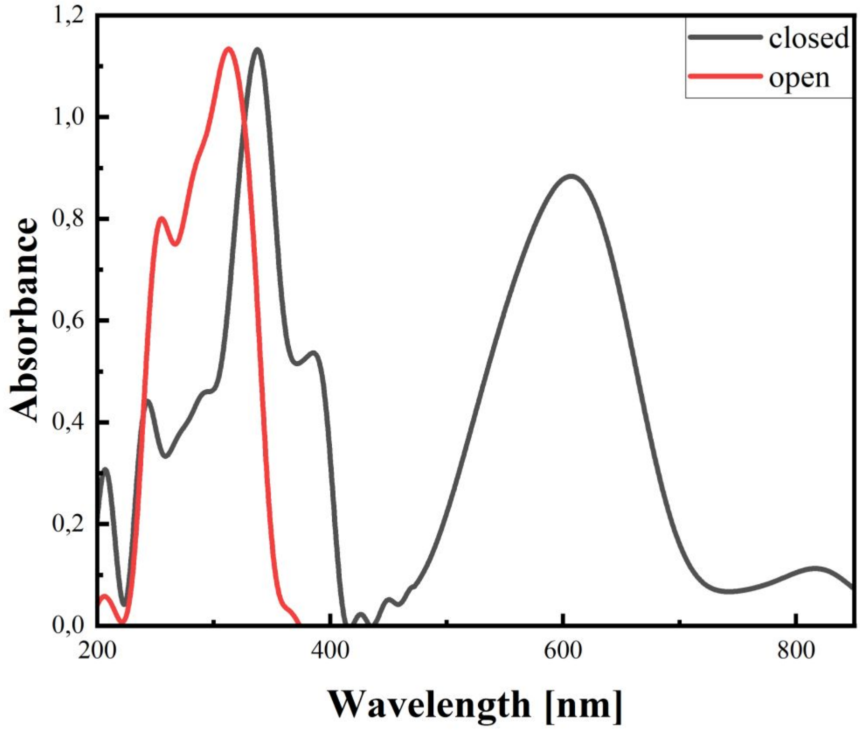

Synthesis of DAE-10

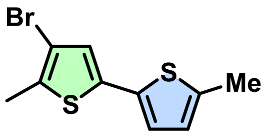

Following General Procedure C, 4.99 g bromothiophene (1 eq.), 2.39 g (5-methylthiophen-2-yl)boronic acid (1.1 eq.), 4.96 g Na_2CO_3 (3 eq.) and 901 mg $Pd(PPh_3)_4$ (0.05 eq.) were used. The resulting crude product was purified *via* column chromatography using a gradient of petroleum ether to petroleum ether/ ethyl acetate mixture (40:1 *v*:*v*) to give 2.73 g of DAE-10 precursor as a light brown solid (yield 65%). ^1H NMR (400 MHz, $CDCl_3$): δ=6.89-6.87 (m, 2H), 6.66-6.63 (m, 1H), 2.47 (s, 3H), 2.37 (s, 3H) ppm. HRMS (APCI): calc.: 271.9329 [M], found: 271.9319 [M].
Spectroscopic data are in agreement with those reported in the literature.[8]

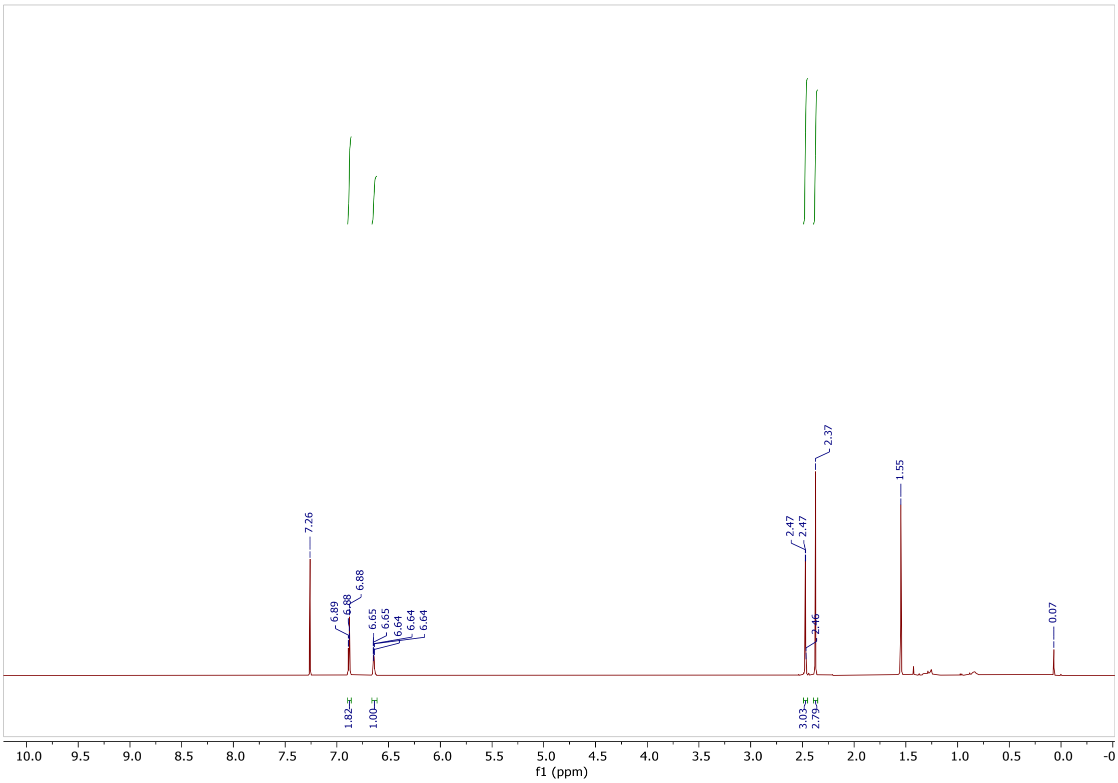

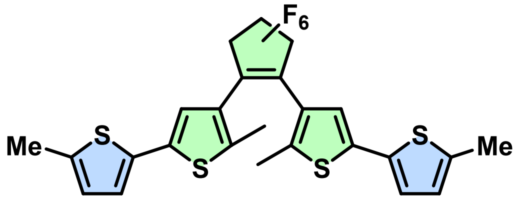

Following General Procedure B, 750 mg 4-bromo-5,5'-dimethyl-2,2'-bithiophene (1.0 eq.), 1,23 mL *n*-BuLi (2.8 M in hexane, 1.25 eq.) and 180 µl octafluorocyclopentene (0.5 eq.) reacted in 40 mL of dry THF. The crude product was purified *via* column chromatography using a gradient of petroleum ether to petroleum ether/ ethyl acetate (50:1 *v*:*v*) mixture to give 162 mg of DAE-10 as a beige solid (yield 22 %). ^{1}H NMR (400 MHz, $CDCl_3$): δ=6.96 (s, 2H), 6.85 (d, J = 3.48 Hz, 2H), 6.59 (d, J = 3.56 Hz, 2H), 2.41 (s, 6H), 1.85 (s, 6H) ppm. ^{13}C NMR (100 MHz, $CDCl_3$): δ=140.3 (o), 139.9 (o), 136.1 (o), 134.0 (o), 126.1 (+), 125.6 (o), 124.1 (+), 122.1 (+), 15.5 (+), 14.6 (+) ppm. IR (ATR): $\tilde{\nu}$ = 3060, 2943, 2920, 2855, 1619, 1500, 1439, 1337, 1261, 1182, 1103, 1052, 985, 900, 865, 792, 737, 530, 475 cm^{-1}. HRMS (APCI): calc.: 561.0268 [$M+H^{+}$], found: 561.0264 [$M+H^{+}$].
Spectroscopic data are in agreement with those reported in the literature.[2]

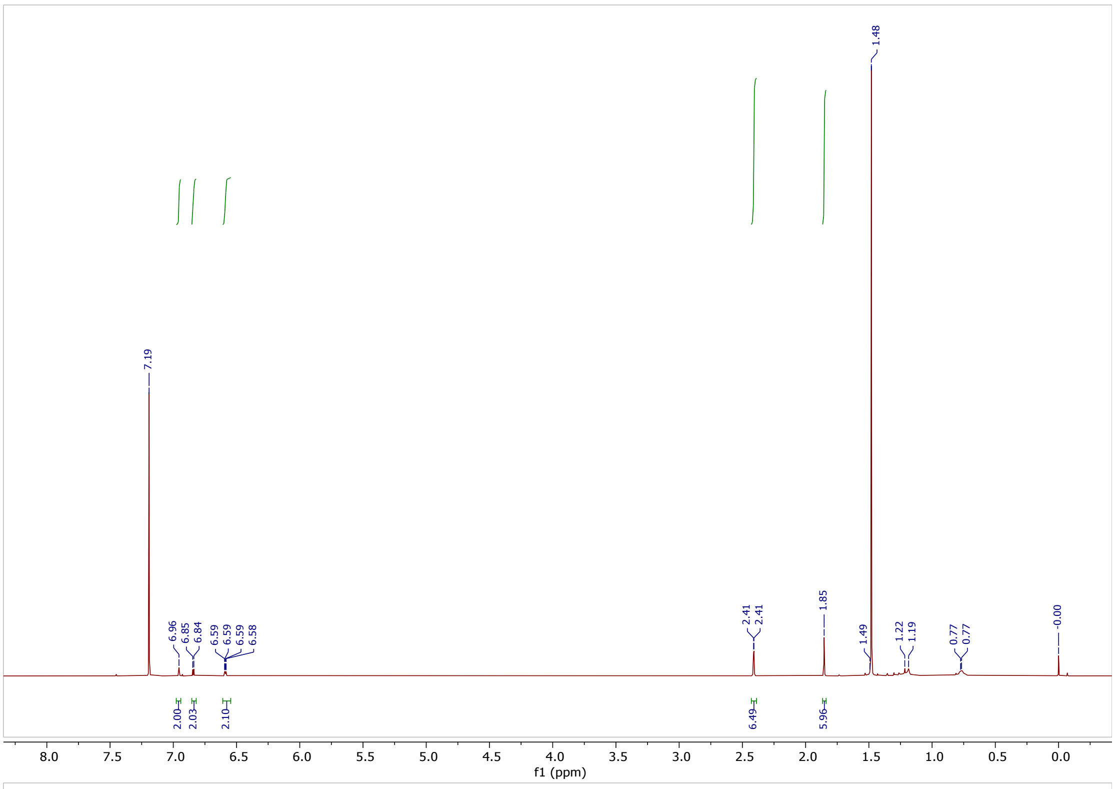
7.19
6.96
6.85
6.84
6.59
6.59
6.59
6.58
2.41
2.41
1.85
1.49
1.48
1.22
1.19
0.77
0.77
-0.00
2.00
2.03
2.10
6.49
5.96
8.0 7.5 7.0 6.5 6.0 5.5 5.0 4.5 4.0 3.5 3.0 2.5 2.0 1.5 1.0 0.5 0.0
f1 (ppm)

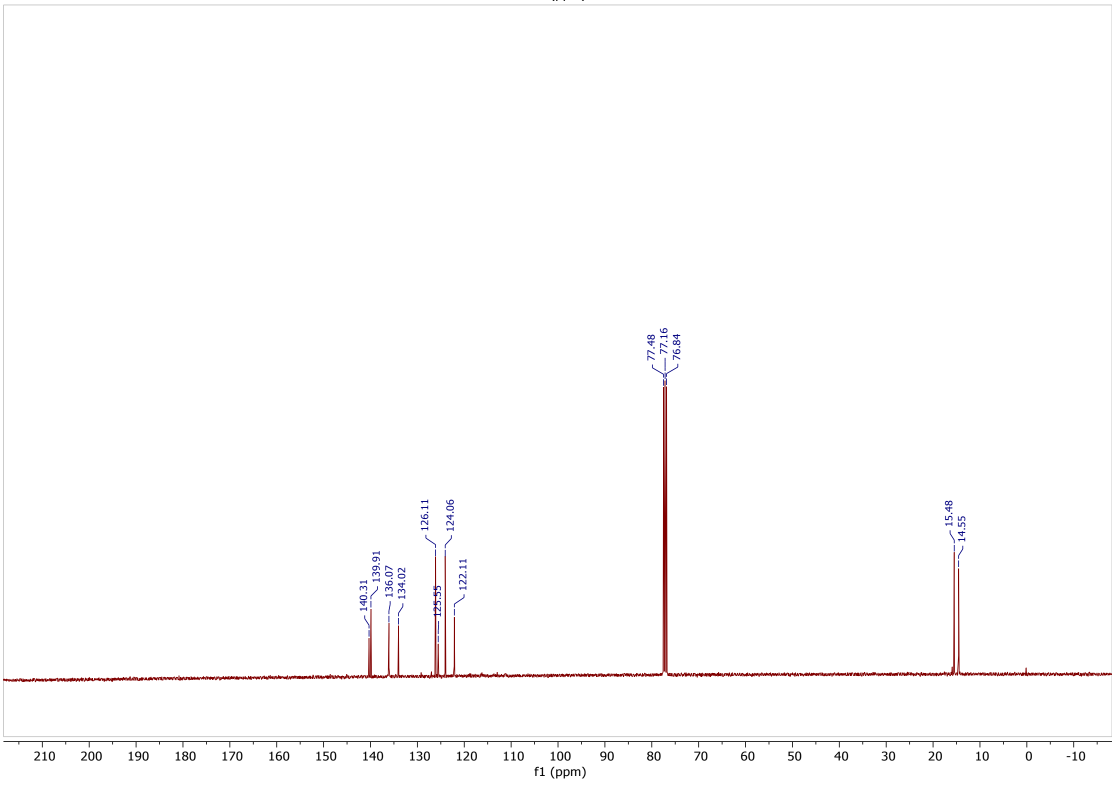
140.31
139.91
136.07
134.02
126.11
125.55
124.06
122.11
77.48
77.16
76.84
15.48
14.55
210 200 190 180 170 160 150 140 130 120 110 100 90 80 70 60 50 40 30 20 10 0 -10
f1 (ppm)

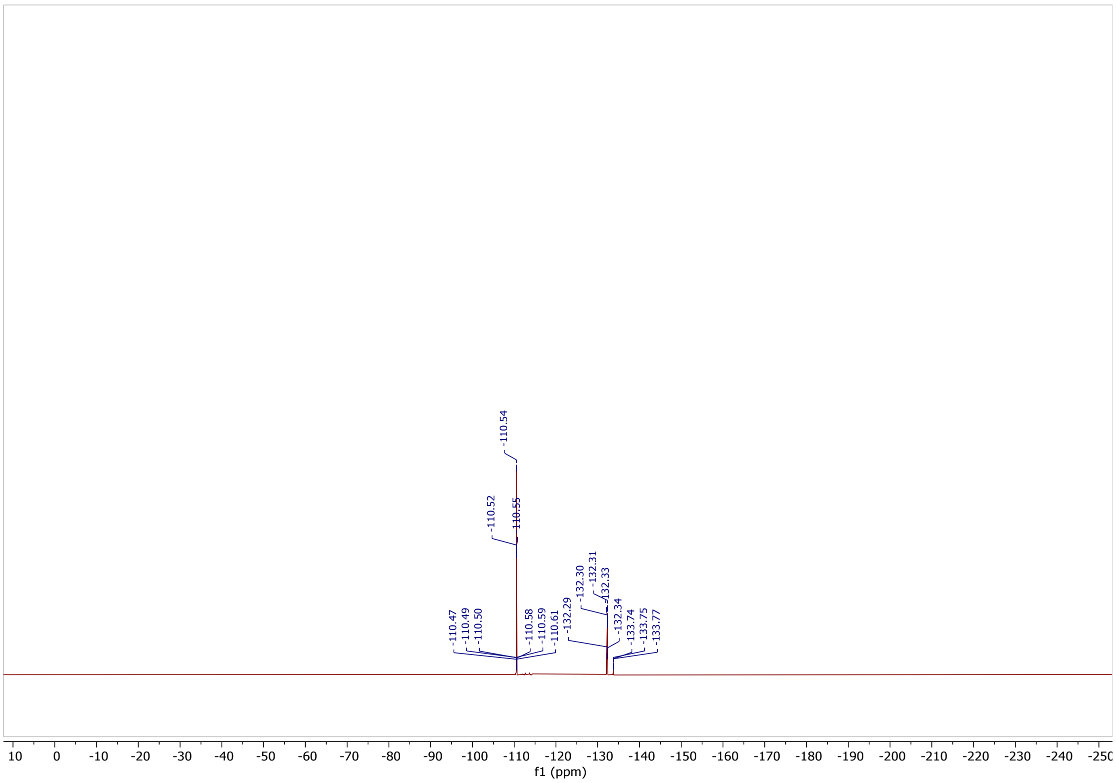
-110.54
-110.52
-110.55
-110.47
-110.49
-110.50
-110.58
-110.59
-110.61
-132.29
-132.30
-132.31
-132.33
-132.34
-133.74
-133.75
-133.77
f1 (ppm)

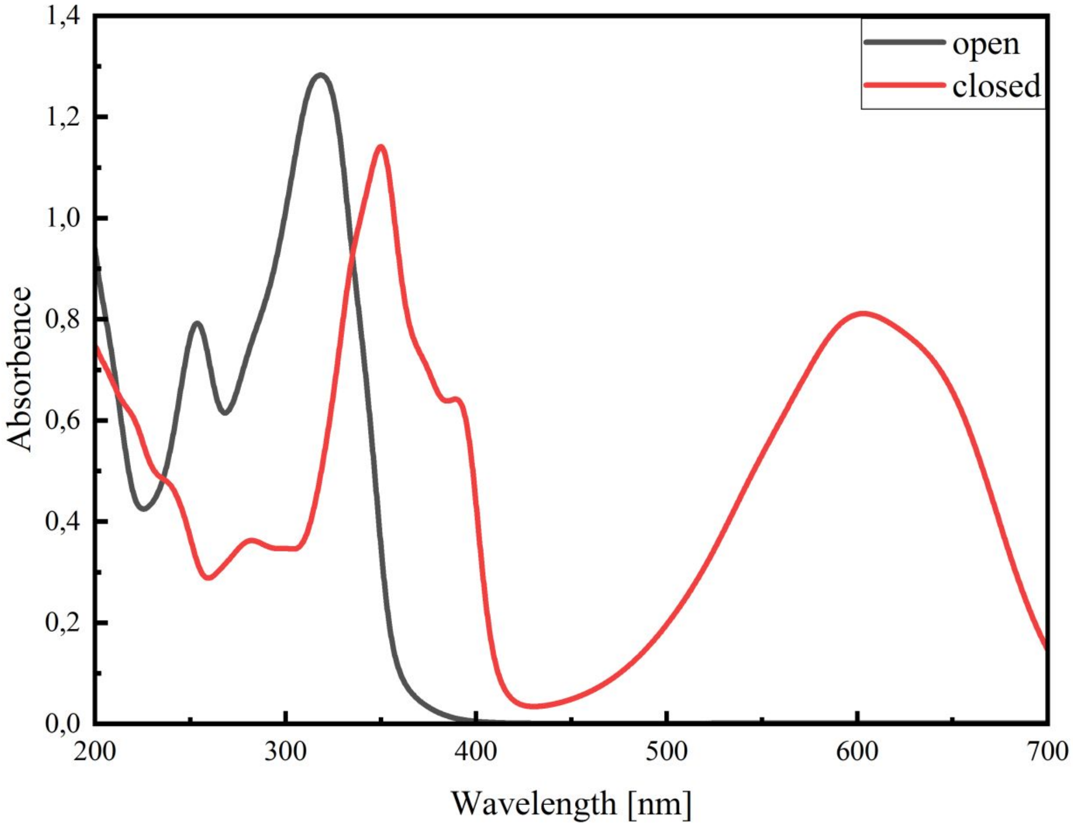
open
closed
Absorbence
Wavelength [nm]

Synthesis of DAE-11

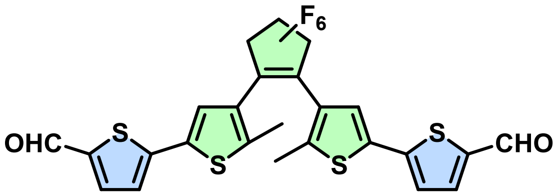

Following General Procedure A, 222 mg DAE-Br_2 (1.0 eq.), 165 mg (5-formylthiophen-2-yl)boronic acid (2.5 eq.), 224 mg K_3PO_4 (2.5 eq.) and 16.5 mg G3 SPhos Pd (0.05 eq.) were used. The resulting crude product was purified *via* column chromatography using a petroleum ether/ ethyl acetate mixture (3:1 *v*:*v*) to give 173 mg of DAE-14 as a blue solid (yield 70%). ^{1}H NMR (400 MHz, $CDCl_3$): δ=9.87 (s, 2H), 7.68 (d, J = 3.97 Hz, 2H), 7.29 (s, 2H), 7.22 (d, J = 3.97 Hz, 2H), 2.01 (s, 6H) ppm. ^{13}C NMR (100 MHz, $CDCl_3$): δ=182.6 (+), 145.5 (o), 143.6 (o), 142.5 (o), 137.2 (+), 134.6 (o), 128.6 (+), 126.7 (+), 125.3 (+), 124.8 (+), 99.6 (+), 52.7 (+), 14.8 (+) ppm. IR (ATR): $\tilde{\nu}$ = 3089, 2958, 2841, 1653, 1556, 1516, 1478, 1432, 1337, 1265, 1189, 1109, 1037, 983, 795, 754, 661, 565, 532, 473 cm^{-1}. HRMS (APCI): calc.: 588.9854 [M+H$^+$], found: 588.9852 [M+H$^+$].
Spectroscopic data are in agreement with those reported in the literature.[7]

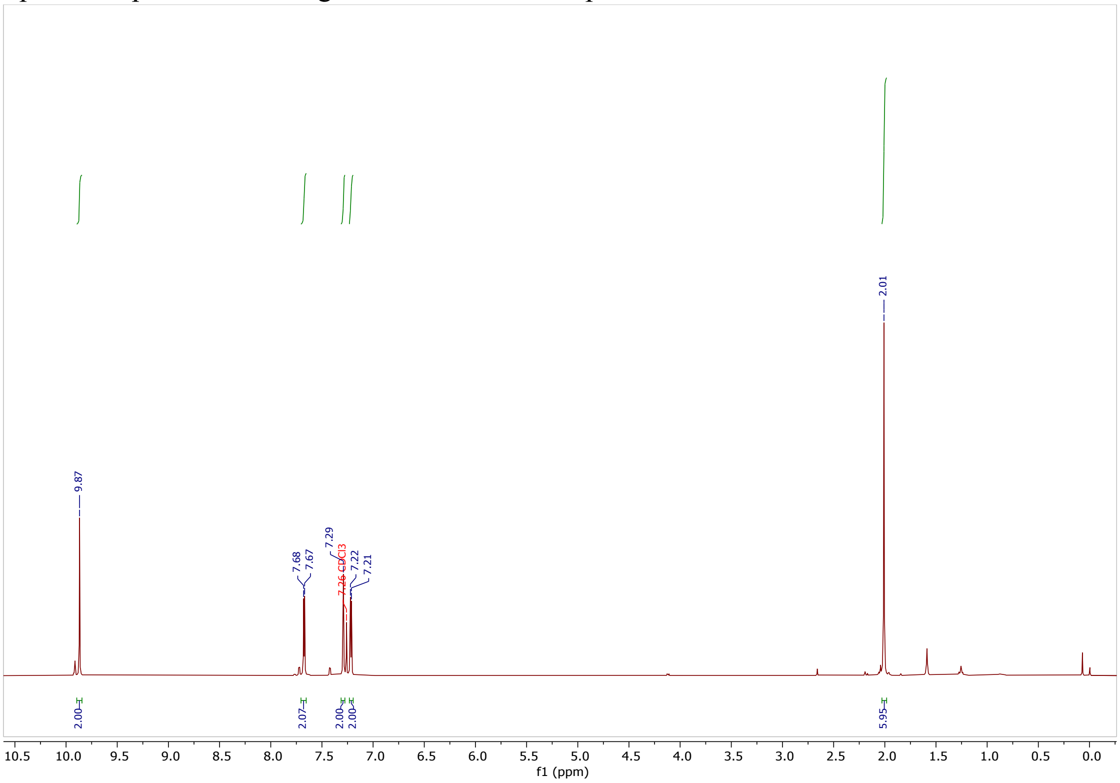

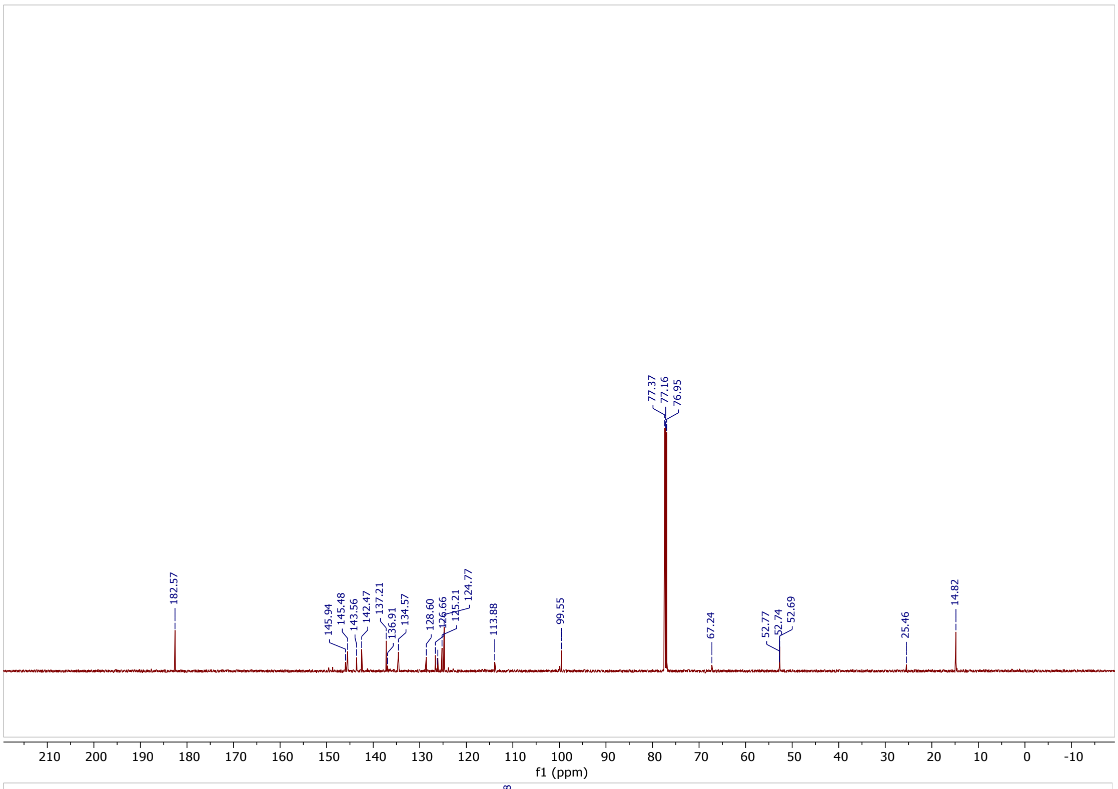
182.57
145.94
145.48
143.56
142.47
137.21
136.91
134.57
128.60
126.66
125.21
124.77
113.88
99.55
77.37
77.16
76.95
67.24
52.77
52.74
52.69
25.46
14.82
f1 (ppm)

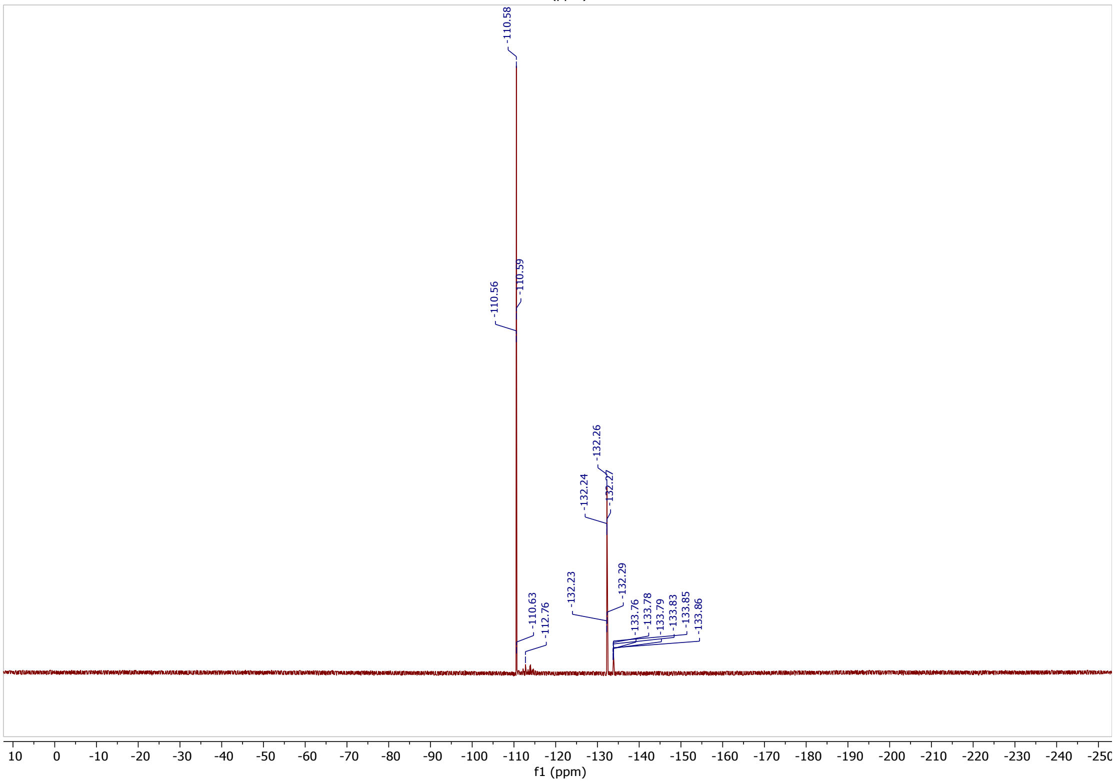
-110.58
-110.56
-110.59
-110.63
-112.76
-132.26
-132.24
-132.27
-132.23
-132.29
-133.76
-133.78
-133.79
-133.83
-133.85
-133.86
f1 (ppm)

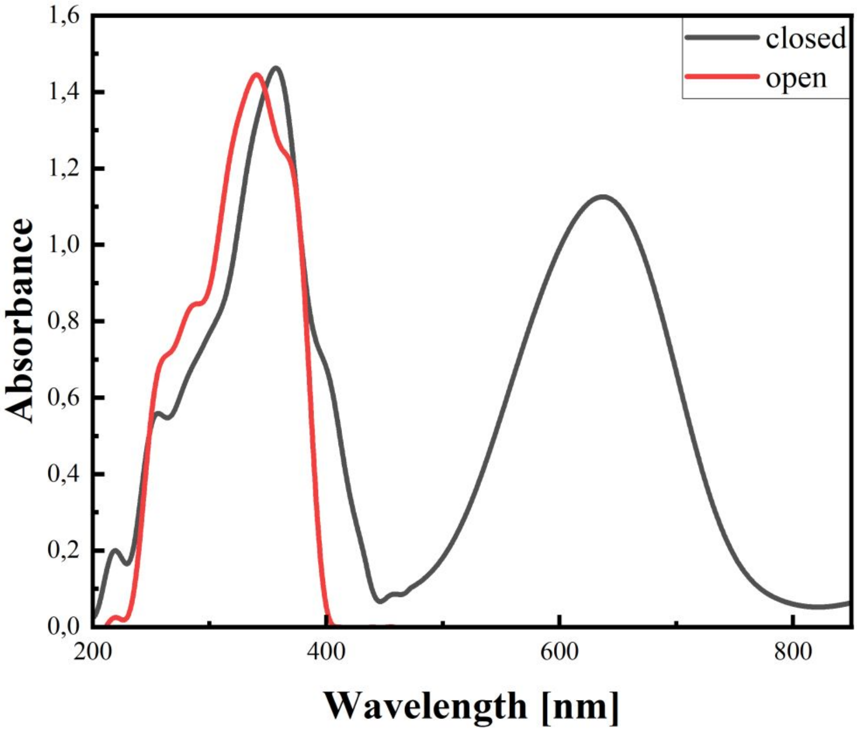

Synthesis of DAE-12

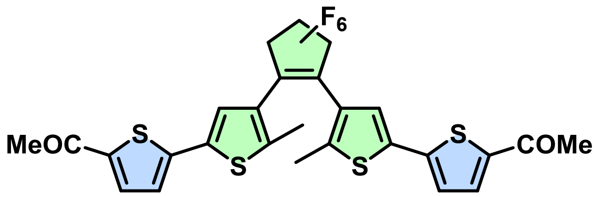

Following General Procedure A, 208 mg DAE-Br_2 (1.0 eq.), 168 mg (5-acetylthiophen-2-yl)boronic acid (2.5 eq.), 210 mg K_3PO_4 (2.5 eq.) and 15.4 mg G3 SPhos Pd (0.05 eq.) were used. The resulting crude product was purified *via* column chromatography using a gradient of petroleum ether/ ethyl acetate mixture (7:1 to 3:1 *v*:*v*) to give 157 mg of DAE-15 as a dark blue solid (yield 65%). ^{1}H NMR (400 MHz, $CDCl_3$): δ=7.59 (d, J = 3.94 Hz, 2H), 7.25 (s, 2H), 7.14 (d, J = 3.94 Hz, 2H), 2.55 (s, 6H), 1.99 (s, 6H) ppm. ^{13}C NMR (100 MHz, $CDCl_3$): δ=190.4 (o), 144.2 (o), 143.2 (o), 143.0 (o), 134.8 (o), 133.3 (+), 126.0 (o), 124.7 (+), 26.7 (+), 14.8 (+) ppm. IR (ATR): $\tilde{\nu}$ = 3064, 2926, 1656, 1641, 1474, 1435, 1337, 1265, 1188, 1110, 1049, 986, 860, 798, 745, 590, 461 cm^{-1}. HRMS (APCI): calc.: 617.0167 [M+H$^+$], found: 617.0162 [M+H$^+$].

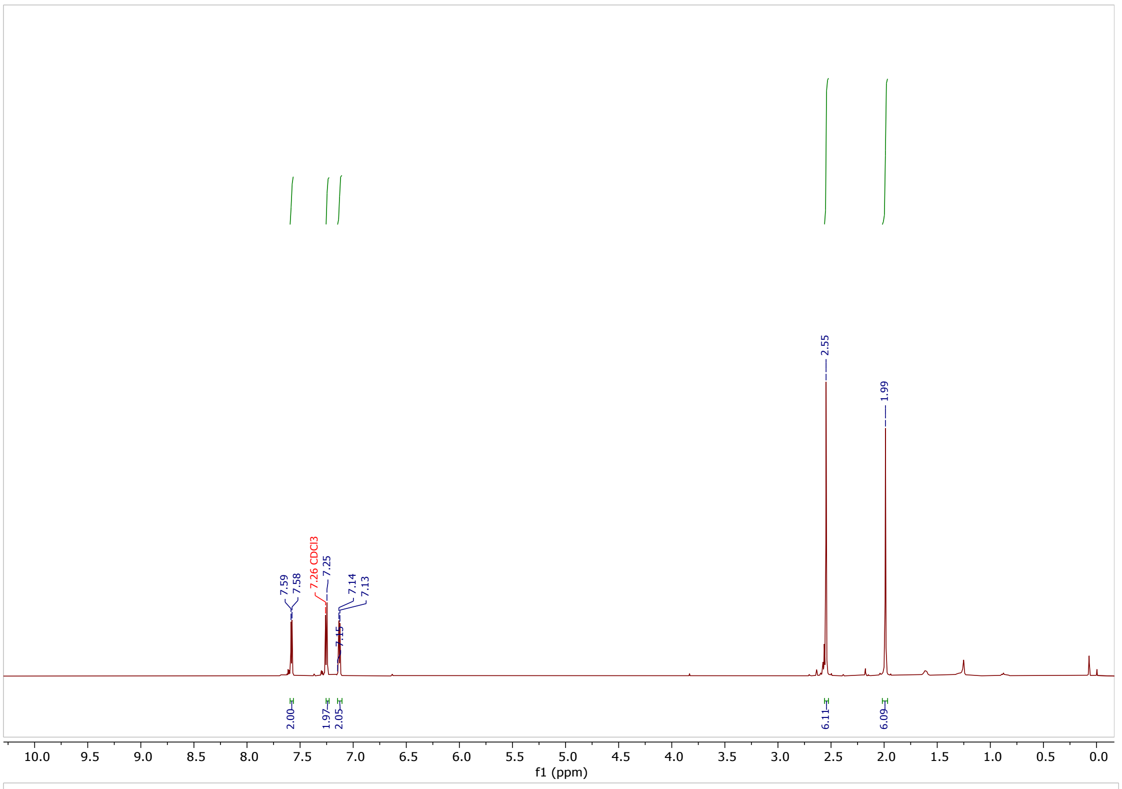
7.59
7.58
7.26 CDCl3
7.25
7.15
7.14
7.13
2.55
1.99
2.00
1.97
2.05
6.11
6.09
f1 (ppm)

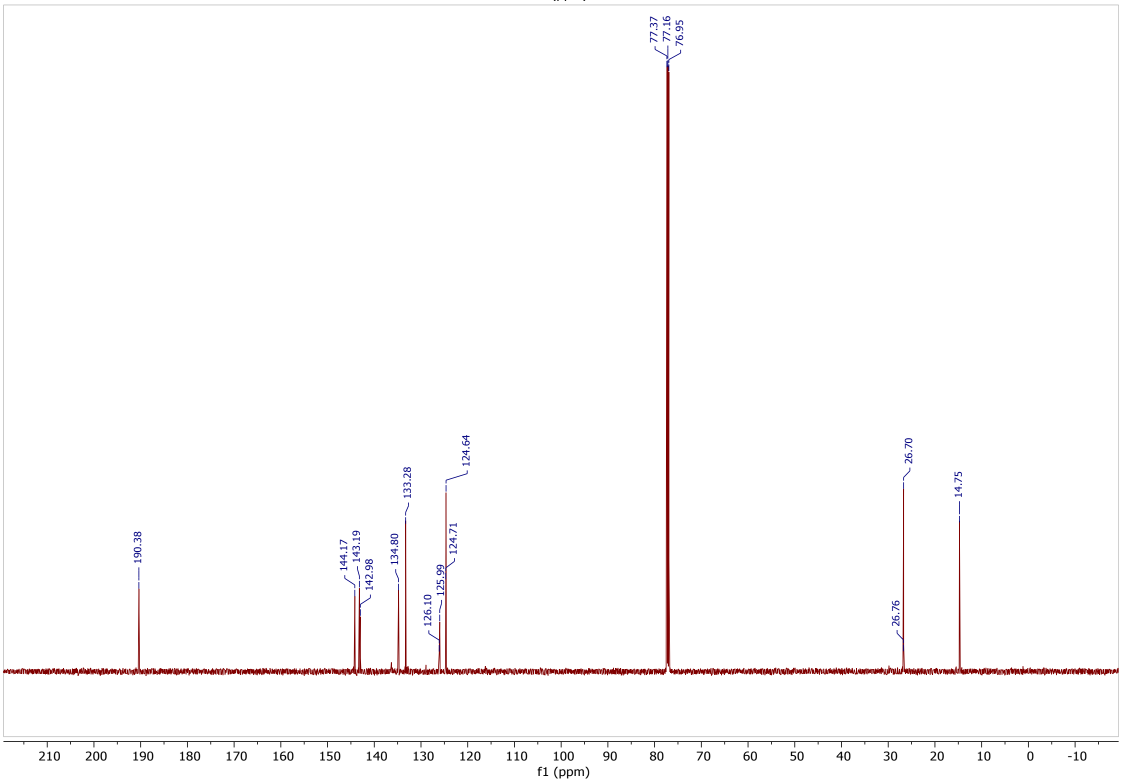
77.37
77.16
76.95
190.38
144.17
143.19
142.98
134.80
133.28
126.10
125.99
124.71
124.64
26.76
26.70
14.75
f1 (ppm)

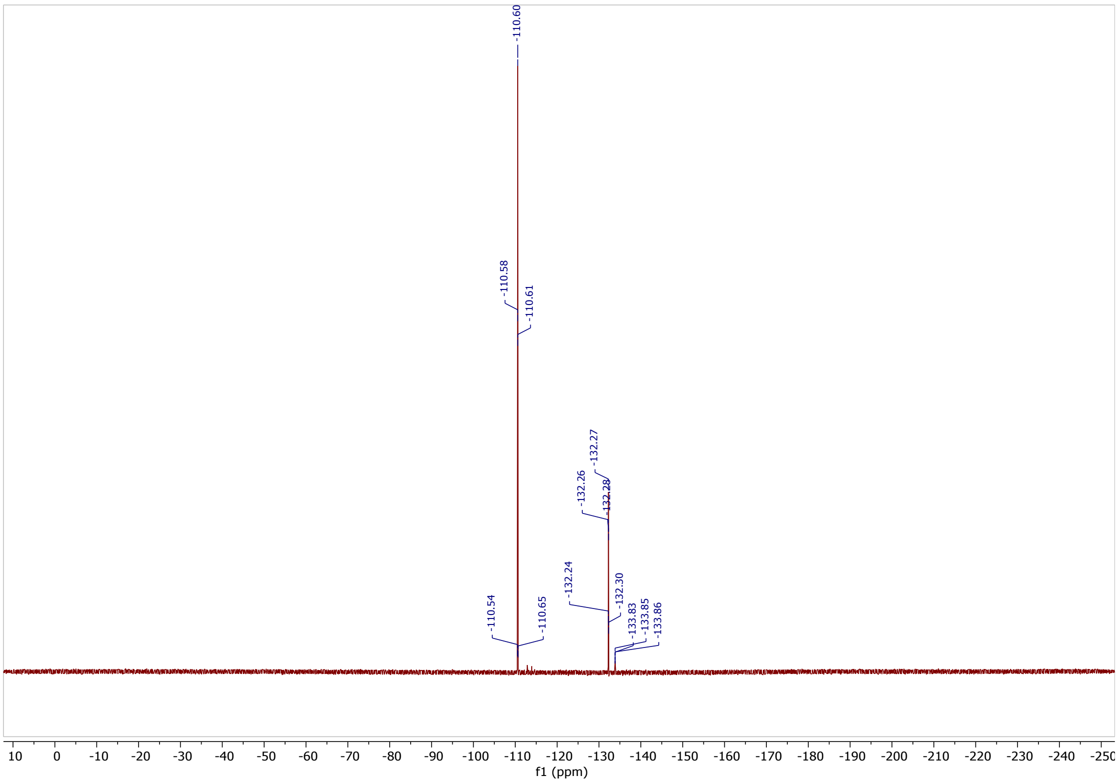
-110.60
-110.58
-110.61
-132.27
-132.26
-132.28
-132.24
-132.30
-110.54
-110.65
-133.83
-133.85
-133.86
10 0 -10 -20 -30 -40 -50 -60 -70 -80 -90 -100 -110 -120 -130 -140 -150 -160 -170 -180 -190 -200 -210 -220 -230 -240 -250
f1 (ppm)

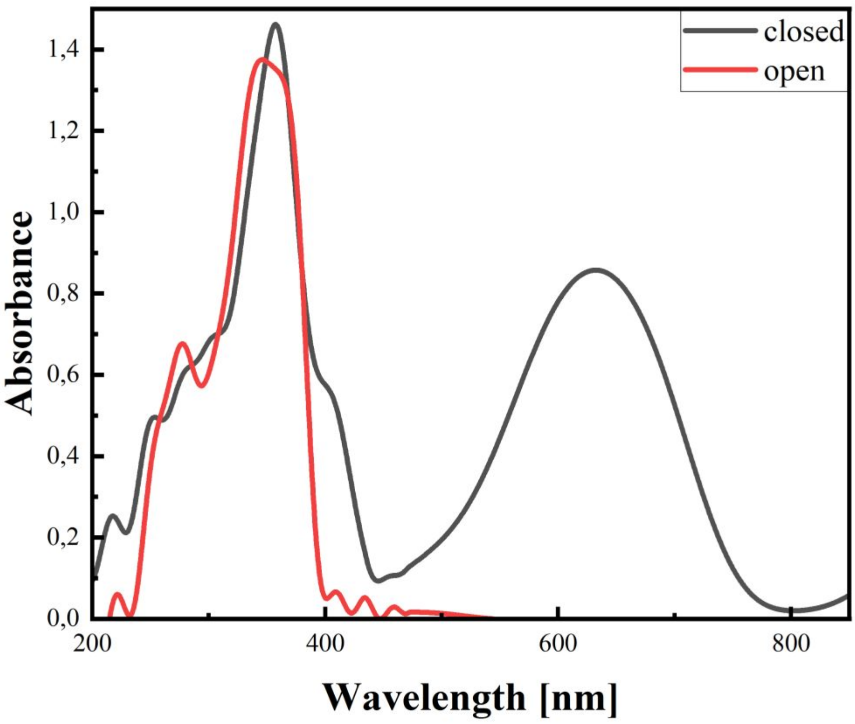
closed
open
Absorbance
0,0
0,2
0,4
0,6
0,8
1,0
1,2
1,4
200
400
600
800
Wavelength [nm]

Synthesis of DAE-13 *via* Wittig olefination

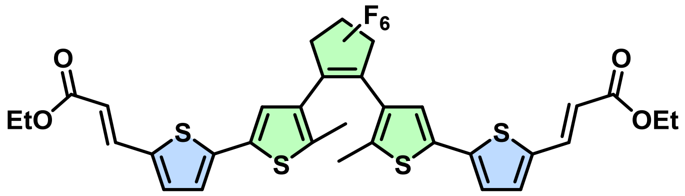

120 mg of DAE-14 were dissolved in 20 mL dry THF. To the vigorously stirred mixture 199 mg ethyl (triphenylphosphoranylidene)acetate (2.8 eq.) were added in one portion and the resulting solution was refluxed for 12 h. The reaction was quenched with the addition of 20 mL of a sat. NaCl solution. The resulting biphasic mixture was separated and the water phase was extracted three times with 10 mL diethyl ether. The combined organic phases were washed two times with sat. NaCl solution and one additional time with deionized water. The remaining solution was dried over $MgSO_4$. The solids were removed by filtration and the solvents were removed *in vacuo*. The crude product was further purified *via* column chromatography using a petroleum ether/ ethyl acetate mixture (9:1 *v*:*v*) to give 130.5 mg of DAE-16 as a dark blue solid (yield 88%). ^{1}H NMR (400 MHz, $CDCl_3$): δ=7.71 (d, *J* = 15.65 Hz, 2H), 7.16 (s, 2H), 7.15 (d, *J* = 3.82 Hz, 2H), 7.06 (d, *J* = 3.79 Hz, 2H), 6.20 (d, *J* = 15.67 Hz, 2H), 4.28 (q, *J* = 7.14 Hz, 4H), 1.98 (s, 6H), 1.33 (t, *J* = 7.12 Hz, 6H) ppm. ^{13}C NMR (100 MHz, $CDCl_3$): δ=165.7 (o), 140.9 (o), 137.7 (o), 135.5 (+), 133.9 (o), 130.9 (+), 128.3 (+), 123.7 (+), 122.7 (+), 116.2 (+), 59.6 (-), 28.7 (-), 13.6 (+), 13.3 (+) ppm. IR (ATR): $\tilde{\nu}$ = 2980, 2925, 1703, 1618, 1439, 1367, 1339, 1264, 1156, 1041, 985, 964, 851, 796, 737, 534, 485 cm^{-1}. HRMS (APCI): calc.: 729.0691 [M+H$^+$], found: 729.0679 [M+H$^+$].

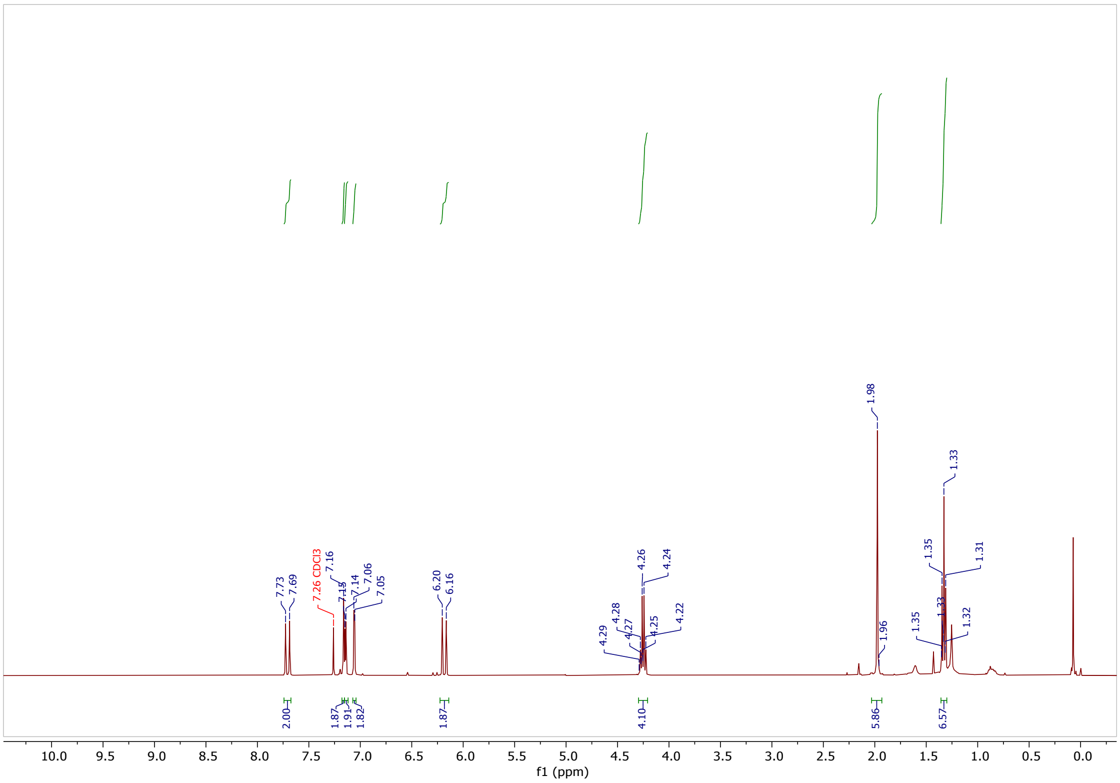

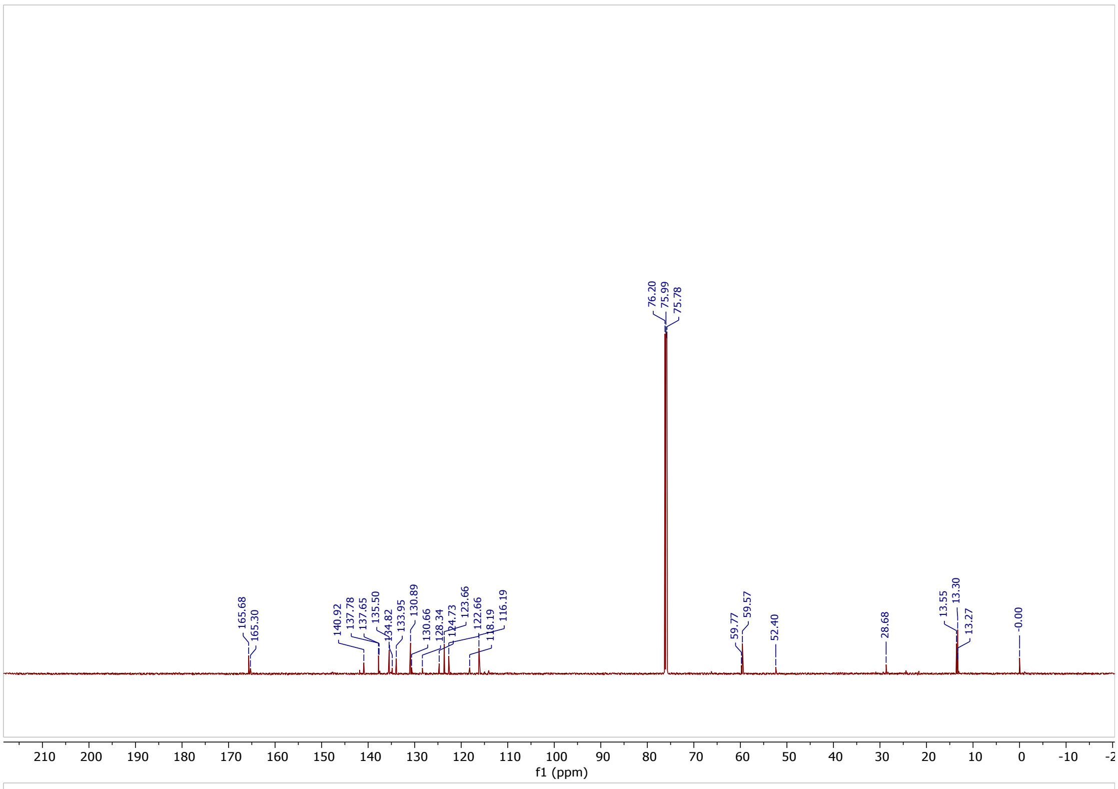
165.68
165.30
140.92
137.78
137.65
135.50
134.82
133.95
130.89
130.66
128.34
124.73
123.66
122.66
118.19
116.19
76.20
75.99
75.78
59.77
59.57
52.40
28.68
13.55
13.30
13.27
-0.00
f1 (ppm)

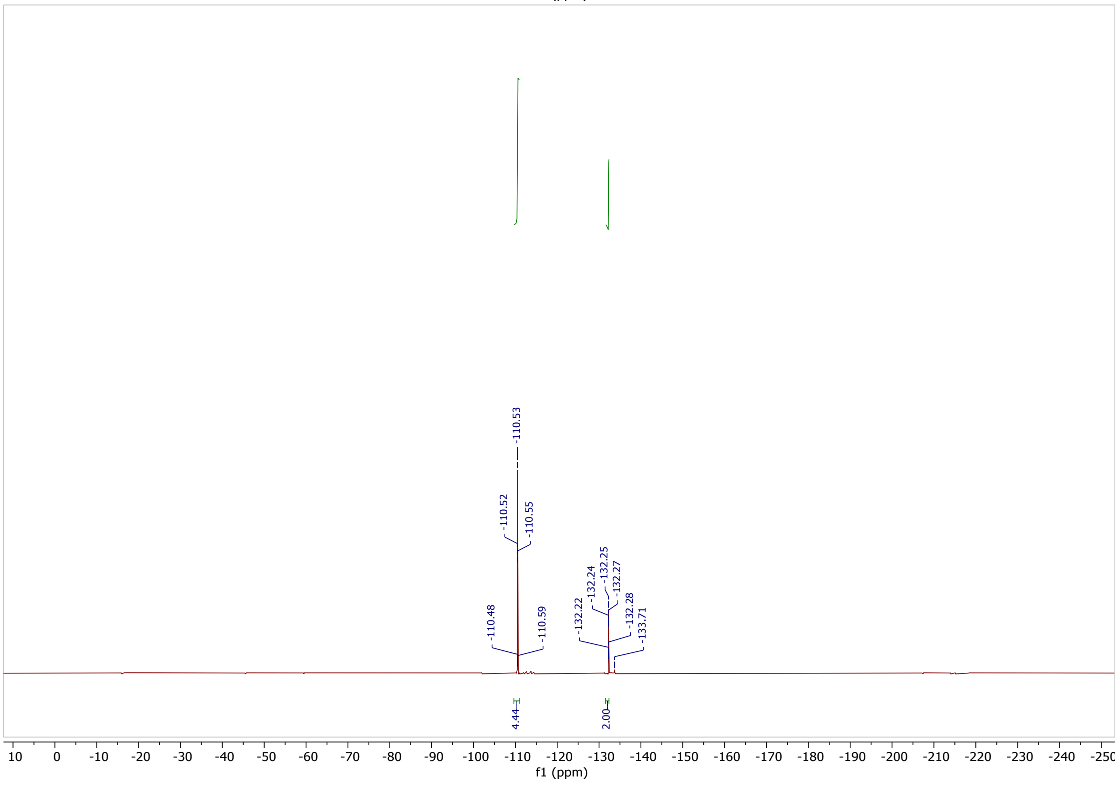
-110.53
-110.52
-110.55
-110.48
-110.59
-132.22
-132.24
-132.25
-132.27
-132.28
-133.71
4.44
2.00
f1 (ppm)

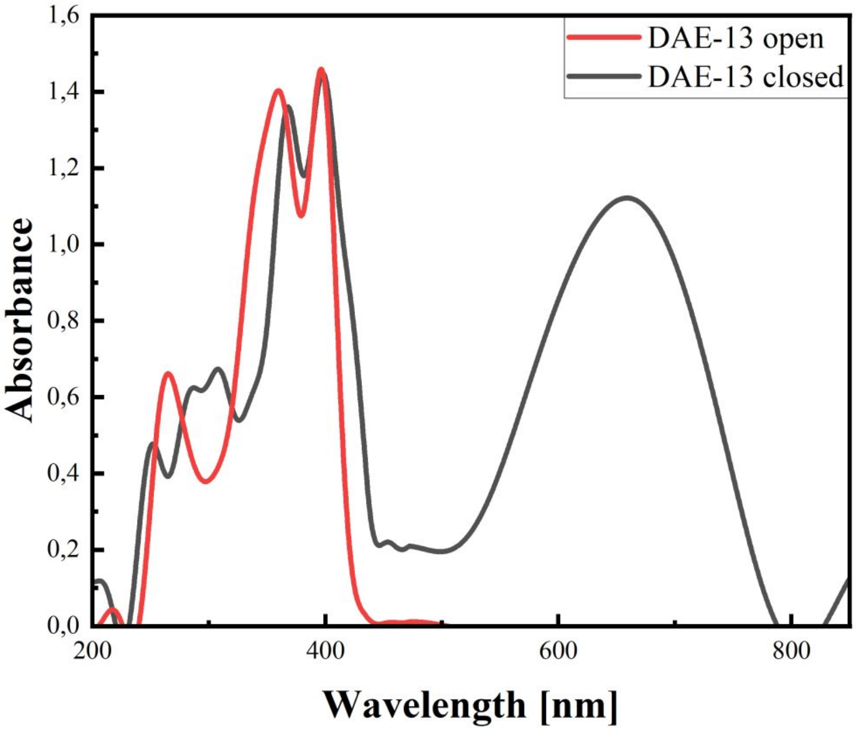

DFT calculations

Geometry optimization, ΔG and HOMO/LUMO calculations

Geometry optimizations were carried out starting from MMFF-optimized structures using density functional theory (DFT) at the B3LYP/Def2TZVP-level, including Grimme's D3 dispersion correction [1] with Becke-Johnson as implemented in Gaussian 16 which was used for all DFT performed DFT calculations. The solvent effects of anisole as well as the PMMA matrix were considered using the SMD implicit solvation model [2]. Frequency calculations were performed at the same level to verify that all stationary points correspond to minima. Of the respective open and closed forms the difference of the sum of electronic and thermal free energies (in Hartree, 1 H = 2625.499 kJ/mol) were calculated. All calculations were conducted on the scientific computing cluster of the *Gesellschaft für wissenschaftliche Datenverarbeitung mbH Göttingen* (GWDG) with 96 CPU cores (2 x Sapphire Rapids 8468) and 514 GB of RAM using the shared-memory multiprocessor parallel execution. Visualization have been carried out using the Avogadro software. For the quantitative molecular surface analysis (volume, surface and elongation in x-axis after the switching process) the software Multiwfn developed by Tian Lu was used [3a-c].

[1] S. Grimme, S. Ehrlich and L. Goerigk, "Effect of the damping function in dispersion corrected density functional theory," *J. Comp. Chem.* **32** (2011) 1456-65.

[2] A. V. Marenich, C. J. Cramer, and D. G. Truhlar, "Universal solvation model based on solute electron density and a continuum model of the solvent defined by the bulk dielectric constant and atomic surface tensions," *J. Phys. Chem. B*, **113** (2009) 6378-96.

[3] a) Tian Lu, Feiwu Chen, Multiwfn: A Multifunctional Wavefunction Analyzer, J. Comput. Chem. 33, 580-592 (2012) DOI: 10.1002/jcc.22885, b) Tian Lu, A comprehensive electron wavefunction

analysis toolbox for chemists, Multiwfn, J. Chem. Phys., 161, 082503 (2024) DOI: 10.1063/5.0216272, c) Tian Lu, Feiwu Chen, Quantitative analysis of molecular surface based on improved Marching Tetrahedra algorithm, J. Mol. Graph. Model., 38, 314-323 (2012) DOI: 10.1016/j.jmgm.2012.07.004.

Complete list of authors of Gaussian 16, Revision C.02:

M. J. Frisch, G. W. Trucks, H. B. Schlegel, G. E. Scuseria, M. A. Robb, J. R. Cheeseman, G. Scalmani, V. Barone, G. A. Petersson, H. Nakatsuji, X. Li, M. Caricato, A. V. Marenich, J. Bloino, B. G. Janesko, R. Gomperts, B. Mennucci, H. P. Hratchian, J. V. Ortiz, A. F. Izmaylov, J. L. Sonnenberg, D. Williams-Young, F. Ding, F. Lipparini, F. Egidi, J. Goings, B. Peng, A. Petrone, T. Henderson, D. Ranasinghe, V. G. Zakrzewski, J. Gao, N. Rega, G. Zheng, W. Liang, M. Hada, M. Ehara, K. Toyota, R. Fukuda, J. Hasegawa, M. Ishida, T. Nakajima, Y. Honda, O. Kitao, H. Nakai, T. Vreven, K. Throssell, J. A. Montgomery, Jr., J. E. Peralta, F. Ogliaro, M. J. Bearpark, J. J. Heyd, E. N. Brothers, K. N. Kudin, V. N. Staroverov, T. A. Keith, R. Kobayashi, J. Normand, K. Raghavachari, A. P. Rendell, J. C. Burant, S. S. Iyengar, J. Tomasi, M. Cossi, J. M. Millam, M. Klene, C. Adamo, R. Cammi, J. W. Ochterski, R. L. Martin, K. Morokuma, O. Farkas, J. B. Foresman, and D. J. Fox, Gaussian, Inc., Wallingford CT, 2019.

DAE1_open

Zero-point correction= 0.445099 (Hartree/Particle)
Thermal correction to Energy= 0.481031
Thermal correction to Enthalpy= 0.481975
Thermal correction to Gibbs Free Energy= 0.373680
Sum of electronic and zero-point Energies= -2664.708025
Sum of electronic and thermal Energies= -2664.672093
Sum of electronic and thermal Enthalpies= -2664.671149
Sum of electronic and thermal Free Energies= -2664.779444
0 imaginary frequencies

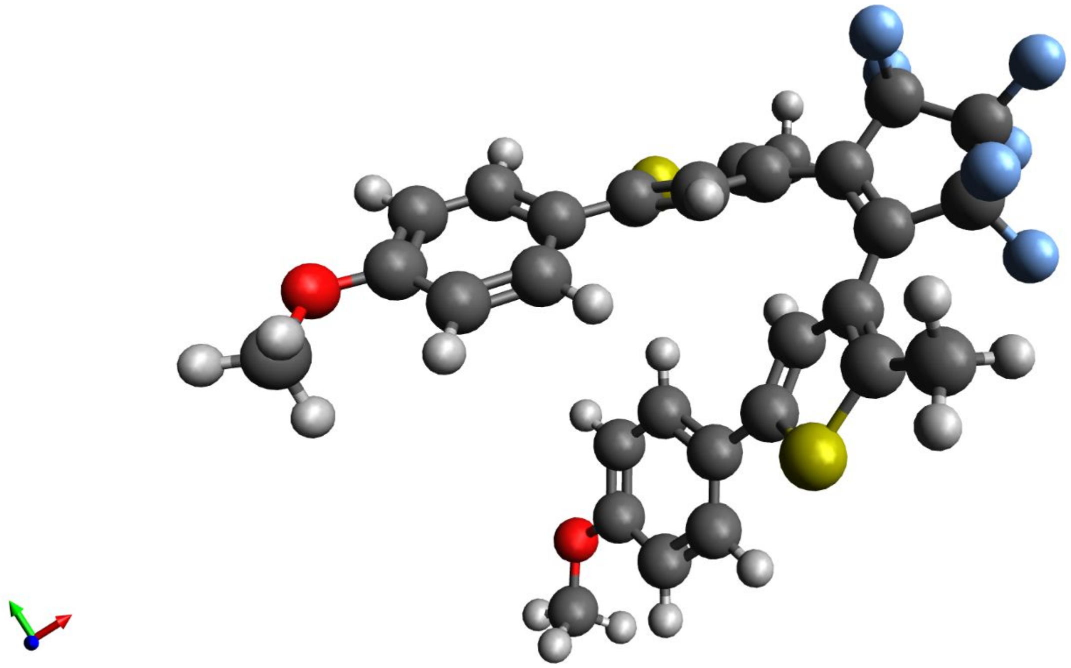

Coordinates (Angstroms)

C	1.2438880000	1.5991430000	-2.1117210000
C	1.5606500000	1.3048930000	-0.8050800000
C	0.5147310000	1.6426790000	0.1103820000
C	-0.5895890000	2.1823570000	-0.4825740000
S	-0.3419680000	2.2836570000	-2.2027440000
C	2.7673780000	0.6061650000	-0.3485890000
C	2.7999470000	-0.5368810000	0.3801710000
C	4.2164340000	-0.8540430000	0.7878780000
C	5.0920530000	0.0227900000	-0.1438530000
C	4.1437870000	1.1858420000	-0.5152830000
C	1.6533050000	-1.3770830000	0.7150310000
F	4.4829680000	-0.4990120000	2.0831570000
F	4.5351520000	-2.1673160000	0.6719440000
F	5.4106260000	-0.6775630000	-1.2579620000
F	6.2366820000	0.4281930000	0.4337620000
F	4.3484170000	2.2358150000	0.3399040000
F	4.4125320000	1.6615730000	-1.7570960000
C	1.4816480000	-2.1249290000	1.8667200000
S	-0.0771240000	-2.8644490000	1.8784320000
C	-0.5141220000	-2.2049290000	0.3290190000
C	0.5142260000	-1.4517890000	-0.1523360000
C	2.0056030000	1.3400680000	-3.3703840000
C	2.3702460000	-2.3352040000	3.0498770000
C	-1.8565800000	2.5679770000	0.1347070000
C	-1.8310830000	-2.4135210000	-0.2678740000
C	-2.3916620000	-1.4188090000	-1.0848280000
C	-3.6358000000	-1.5830520000	-1.6579900000
C	-4.3719420000	-2.7489660000	-1.4252000000

C	-3.8360220000	-3.7445230000	-0.6090990000
C	-2.5784130000	-3.5695680000	-0.0435680000
O	-5.5866090000	-2.8188190000	-2.0249960000
C	-6.3818310000	-3.9776760000	-1.8066540000
C	-2.6388960000	3.6183220000	-0.3650900000
C	-3.8427910000	3.9549610000	0.2221300000
C	-4.3052540000	3.2501180000	1.3367440000
C	-3.5417860000	2.2000570000	1.8471840000
C	-2.3370040000	1.8667880000	1.2426410000
O	-5.4972120000	3.6504880000	1.8456440000
C	-6.0120410000	2.9614590000	2.9786400000
H	0.5946620000	1.4845420000	1.1756510000
H	0.4750270000	-0.9711790000	-1.1158700000
H	2.6025030000	2.2056430000	-3.6657930000
H	2.6831760000	0.4965450000	-3.2449400000
H	1.3293590000	1.1078150000	-4.1943400000
H	2.6677150000	-1.3859910000	3.4958820000
H	1.8581170000	-2.9165760000	3.8162860000
H	3.2787040000	-2.8747410000	2.7786360000
H	-1.8507490000	-0.4966980000	-1.2494050000
H	-4.0657370000	-0.8079780000	-2.2787370000
H	-4.3796610000	-4.6567030000	-0.4136290000
H	-2.1708850000	-4.3627570000	0.5710680000
H	-7.3004070000	-3.8201970000	-2.3677250000
H	-5.8837170000	-4.8784110000	-2.1757350000
H	-6.6227640000	-4.1016870000	-0.7471830000
H	-2.2906140000	4.1899440000	-1.2163380000
H	-4.4402790000	4.7698530000	-0.1649600000
H	-3.8807670000	1.6264900000	2.6969000000
H	-1.7733770000	1.0266050000	1.6264540000
H	-6.9580580000	3.4427220000	3.2169740000
H	-6.1899710000	1.9058750000	2.7555370000
H	-5.3381210000	3.0451090000	3.8356930000

LUMO (-1.975 eV)

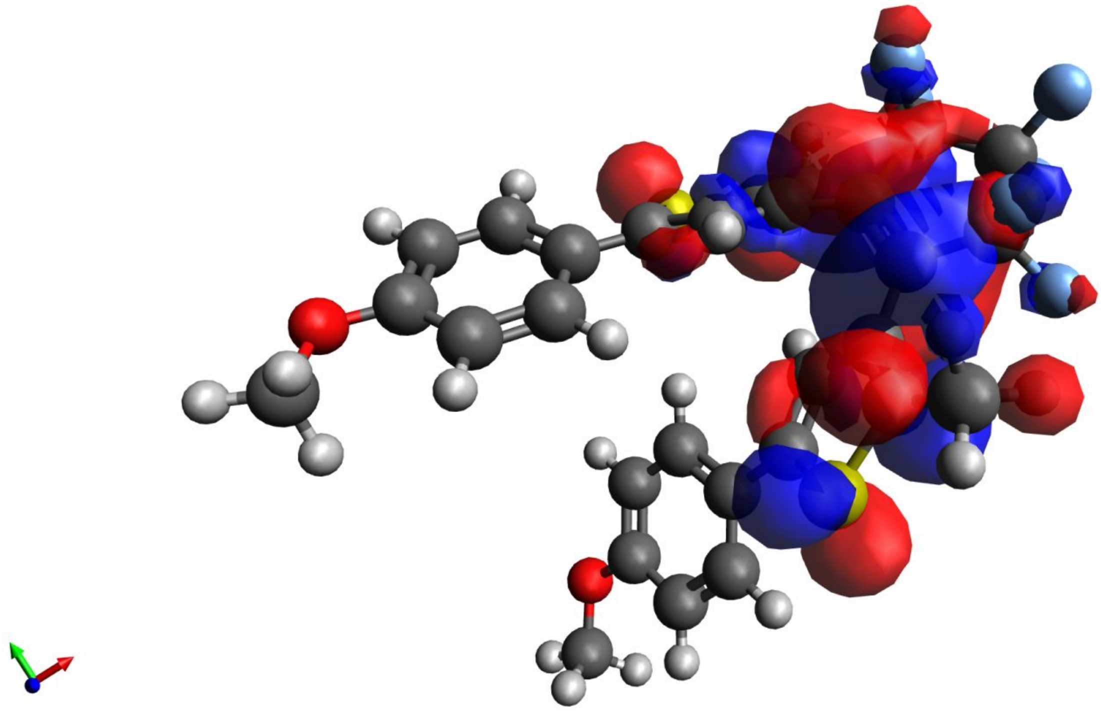

HOMO (-5.603 eV)

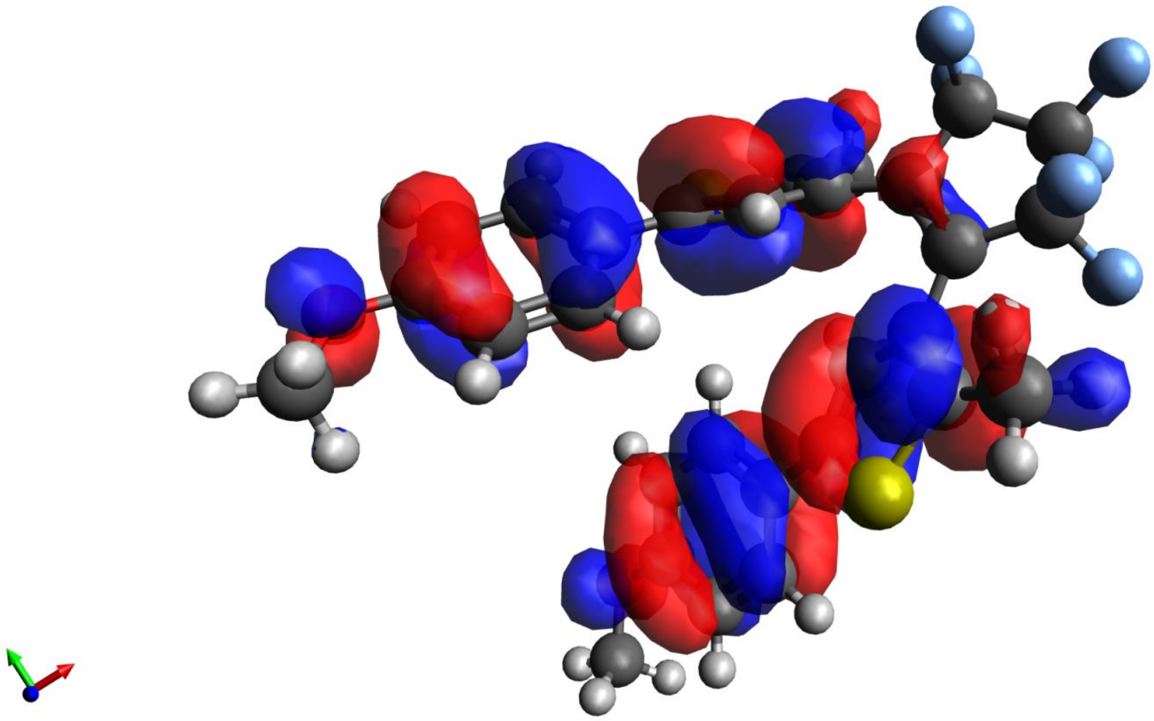

DAE1_closed

Zero-point correction= 0.446642 (Hartree/Particle)
Thermal correction to Energy= 0.481365
Thermal correction to Enthalpy= 0.482309
Thermal correction to Gibbs Free Energy= 0.379188
Sum of electronic and zero-point Energies= -2664.694555
Sum of electronic and thermal Energies= -2664.659833
Sum of electronic and thermal Enthalpies= -2664.658888
Sum of electronic and thermal Free Energies= -2664.762009
0 imaginary frequencies

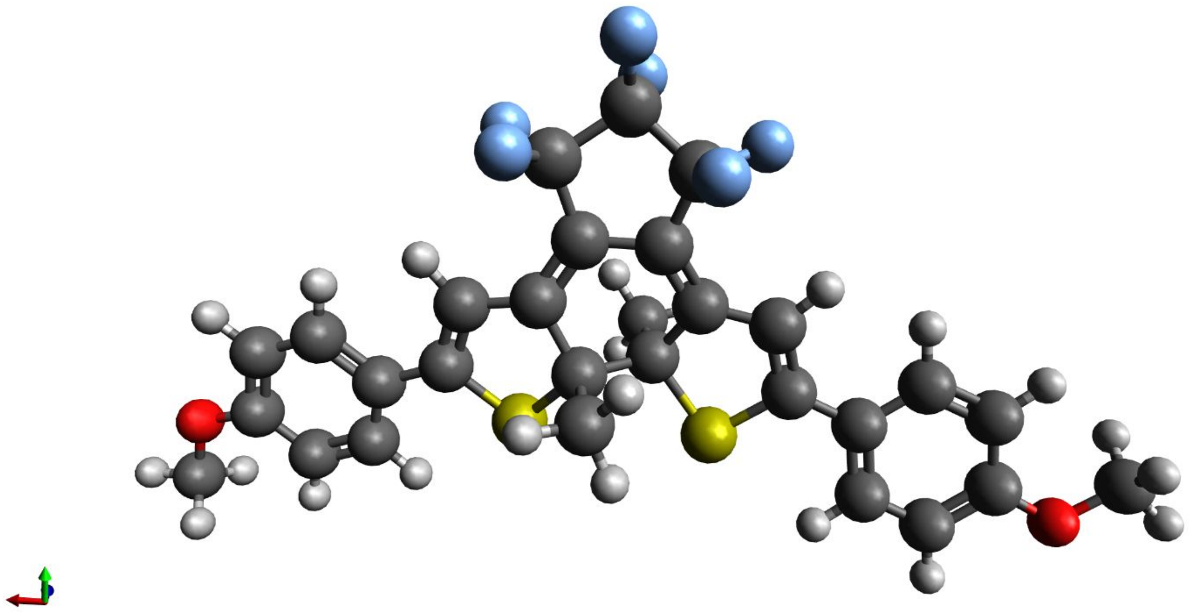

Coordinates (Angstroms)

C	-0.5898210000	-0.4824460000	0.4298050000
C	-1.4386880000	0.7554390000	0.1213860000
C	-2.8236100000	0.4679510000	0.0873140000
C	-3.1387730000	-0.8609430000	0.0778260000
S	-1.7201940000	-1.9122290000	0.0990490000
C	-0.7687750000	1.9269940000	-0.0425130000
C	0.6727710000	1.9781470000	-0.0976000000
C	1.1449120000	3.3904730000	-0.0659240000
C	-0.1392700000	4.2231430000	0.2478370000
C	-1.3233240000	3.3010500000	-0.1765620000
C	1.4239660000	0.8502080000	-0.2098490000
F	2.1180450000	3.6418180000	0.8576690000
F	1.6632040000	3.8054860000	-1.2669080000
F	-0.1536900000	5.4186880000	-0.3679490000
F	-0.2139730000	4.4396470000	1.5833770000
F	-2.4280160000	3.5507050000	0.5762840000
F	-1.6745570000	3.6103100000	-1.4706570000
C	0.6672710000	-0.4579330000	-0.4567860000
S	1.8975300000	-1.7830650000	-0.0592430000
C	3.2385640000	-0.6339790000	-0.1063940000
C	2.8259600000	0.6667460000	-0.1756310000

C	-0.2736370000	-0.4770980000	1.9375080000
C	0.3530560000	-0.5449940000	-1.9624240000
C	-4.4677030000	-1.4459430000	0.0334290000
C	4.6061540000	-1.1191120000	-0.0463260000
C	5.6946840000	-0.2256780000	-0.0290460000
C	6.9916300000	-0.6808640000	0.0288870000
C	7.2592050000	-2.0557560000	0.0726560000
C	6.1973300000	-2.9599630000	0.0586880000
C	4.8947810000	-2.4869980000	0.0001680000
O	8.5624110000	-2.4059630000	0.1263390000
C	8.8928700000	-3.7913940000	0.1693010000
C	-4.6575610000	-2.8155110000	-0.2167020000
C	-5.9190990000	-3.3694620000	-0.2642610000
C	-7.0472270000	-2.5697270000	-0.0576580000
C	-6.8826800000	-1.2049830000	0.1968180000
C	-5.6101470000	-0.6617790000	0.2406170000
O	-8.2424520000	-3.1964390000	-0.1173920000
C	-9.4256960000	-2.4300650000	0.0911450000
H	-3.5716540000	1.2423740000	0.0125750000
H	3.5149750000	1.4976500000	-0.1671920000
H	-1.2036070000	-0.4111310000	2.4999740000
H	0.2502600000	-1.3832690000	2.2330130000
H	0.3468690000	0.3815670000	2.1939820000
H	1.2767950000	-0.4405300000	-2.5293190000
H	-0.1057820000	-1.4985270000	-2.2136880000
H	-0.3253790000	0.2554070000	-2.2579070000
H	5.5198870000	0.8405320000	-0.0593170000
H	7.8227290000	0.0118190000	0.0415070000
H	6.3715780000	-4.0247320000	0.0918640000
H	4.0886600000	-3.2088900000	-0.0165850000
H	9.9787340000	-3.8371550000	0.2062940000
H	8.4790800000	-4.2693630000	1.0608350000
H	8.5384500000	-4.3105060000	-0.7247770000
H	-3.8026730000	-3.4569790000	-0.3872450000
H	-6.0553760000	-4.4244650000	-0.4609650000
H	-7.7351760000	-0.5650100000	0.3675690000
H	-5.5078500000	0.3928810000	0.4562150000
H	-10.2529170000	-3.1293330000	-0.0051570000
H	-9.4400590000	-1.9876520000	1.0904470000
H	-9.5265830000	-1.6438060000	-0.6612160000

LUMO (-2.683 eV)

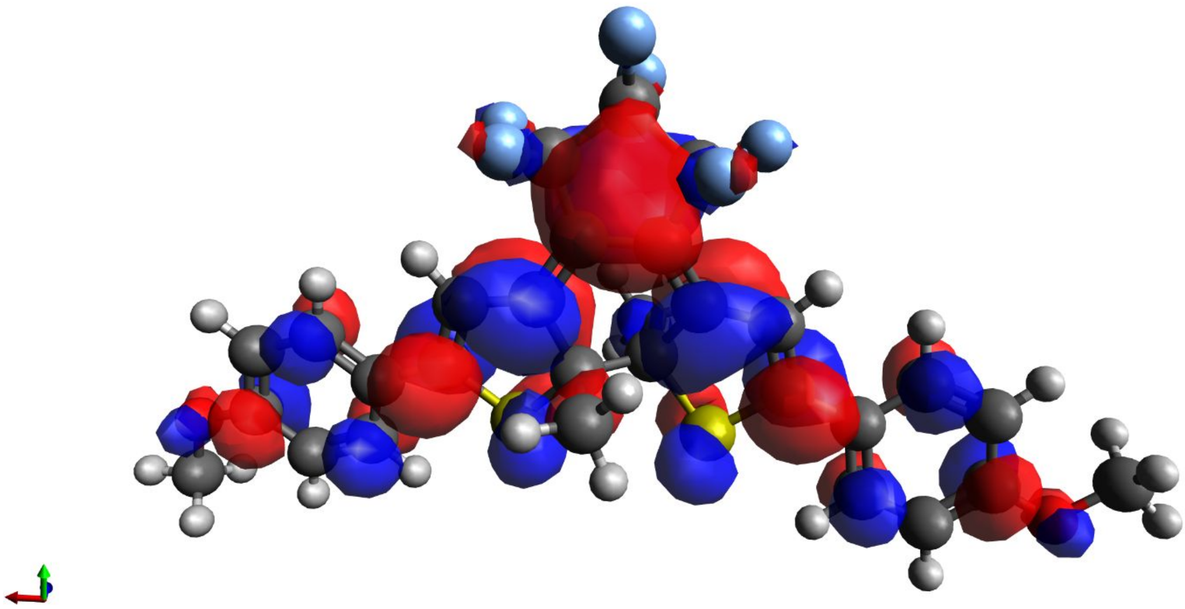

HOMO (-4.946 eV)

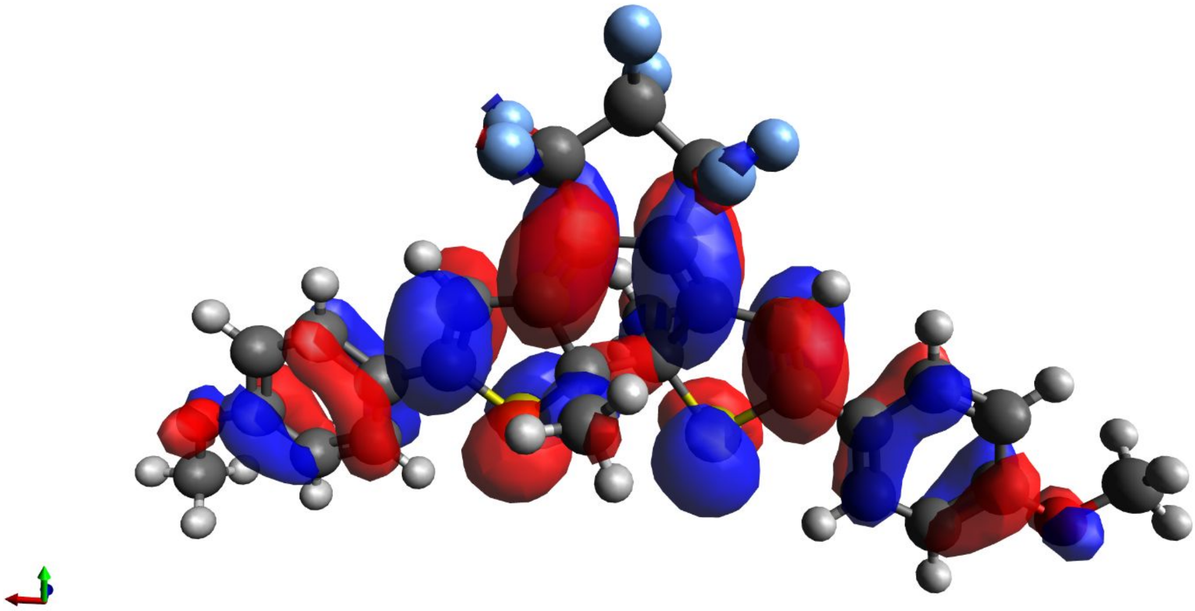

DAE8_open

Zero-point correction= 0.396603 (Hartree/Particle)
Thermal correction to Energy= 0.432146
Thermal correction to Enthalpy= 0.433090
Thermal correction to Gibbs Free Energy= 0.324968
Sum of electronic and zero-point Energies= -2728.868807
Sum of electronic and thermal Energies= -2728.833264
Sum of electronic and thermal Enthalpies= -2728.832320

Sum of electronic and thermal Free Energies= -2728.940442
0 imaginary frequencies

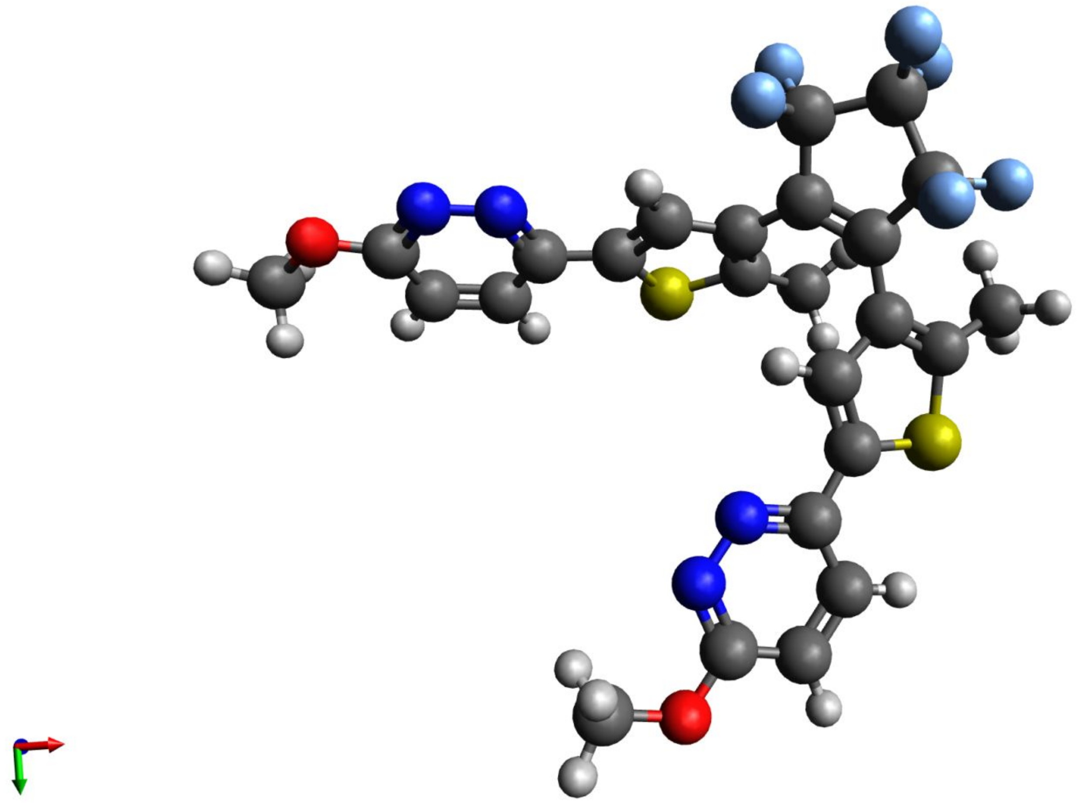

Coordinates (Angstroms)

C	3.6035860000	0.8231290000	0.9811520000
C	2.6228120000	0.2642190000	0.1905270000
C	1.6605370000	1.2175600000	-0.2577040000
C	1.9106070000	2.4907390000	0.1686990000
S	3.3429210000	2.5207480000	1.1542840000
C	2.4945900000	-1.1529460000	-0.1649870000
C	1.3820940000	-1.9058090000	-0.0377520000
C	1.5364750000	-3.2318120000	-0.7351150000
C	3.0586590000	-3.3484090000	-1.0168540000
C	3.5539090000	-1.8794520000	-0.9505430000
C	0.1256600000	-1.5576570000	0.6166540000
F	1.0894410000	-4.2785850000	0.0018540000
F	0.8520180000	-3.2684470000	-1.9169430000
F	3.3329330000	-3.9415310000	-2.1912310000
F	3.6467310000	-4.0686450000	-0.0335670000
F	4.7994390000	-1.8131730000	-0.4127770000
F	3.6628340000	-1.3696070000	-2.2126590000

C	0.0326400000	-0.9791420000	1.8671050000
S	-1.6157840000	-0.6986510000	2.2796670000
C	-2.1962090000	-1.3595700000	0.7804270000
C	-1.1487650000	-1.7880780000	0.0157250000
C	4.7312500000	0.1767460000	1.7152580000
C	1.1082670000	-0.6347870000	2.8388610000
C	1.1361650000	3.6956800000	-0.1193410000
C	-3.6185820000	-1.4166570000	0.4553610000
N	-3.9447970000	-2.1130110000	-0.6387120000
N	-5.2002020000	-2.2202260000	-1.0238150000
C	-6.1705670000	-1.6408570000	-0.3229020000
C	-5.9148250000	-0.8950740000	0.8351350000
C	-4.6045340000	-0.7830910000	1.2238690000
O	-7.3916810000	-1.8462960000	-0.8412340000
C	-8.5158540000	-1.2766510000	-0.1720650000
C	1.5938490000	4.9896630000	0.2079110000
C	0.8026690000	6.0559180000	-0.1047300000
C	-0.4177190000	5.7750830000	-0.7412640000
N	-0.8090560000	4.5466130000	-1.0302440000
N	-0.0372500000	3.5100850000	-0.7166550000
O	-1.2027440000	6.8120860000	-1.0603670000
C	-2.4434510000	6.5288220000	-1.7254760000
H	0.8190760000	0.9682470000	-0.8851770000
H	-1.2889410000	-2.2306850000	-0.9579010000
H	5.6616870000	0.2247080000	1.1454660000
H	4.9042760000	0.6672650000	2.6741690000
H	4.5152790000	-0.8726770000	1.9075950000
H	1.9904320000	-1.2494800000	2.6655910000
H	1.4066200000	0.4118570000	2.7479550000
H	0.7776200000	-0.7991390000	3.8652490000
H	-6.7040870000	-0.4211590000	1.3983450000
H	-4.3452430000	-0.2072180000	2.1023620000
H	-9.3845780000	-1.5656280000	-0.7580330000
H	-8.6152360000	-1.6722690000	0.8416700000
H	-8.4473590000	-0.1865620000	-0.1401490000

H	2.5526010000	5.1375520000	0.6871630000
H	1.0854820000	7.0769150000	0.1107880000
H	-2.9053990000	7.4990680000	-1.8907770000
H	-2.2678430000	6.0249880000	-2.6760140000
H	-3.0841780000	5.9046130000	-1.1025110000

LUMO (-1.943 eV)

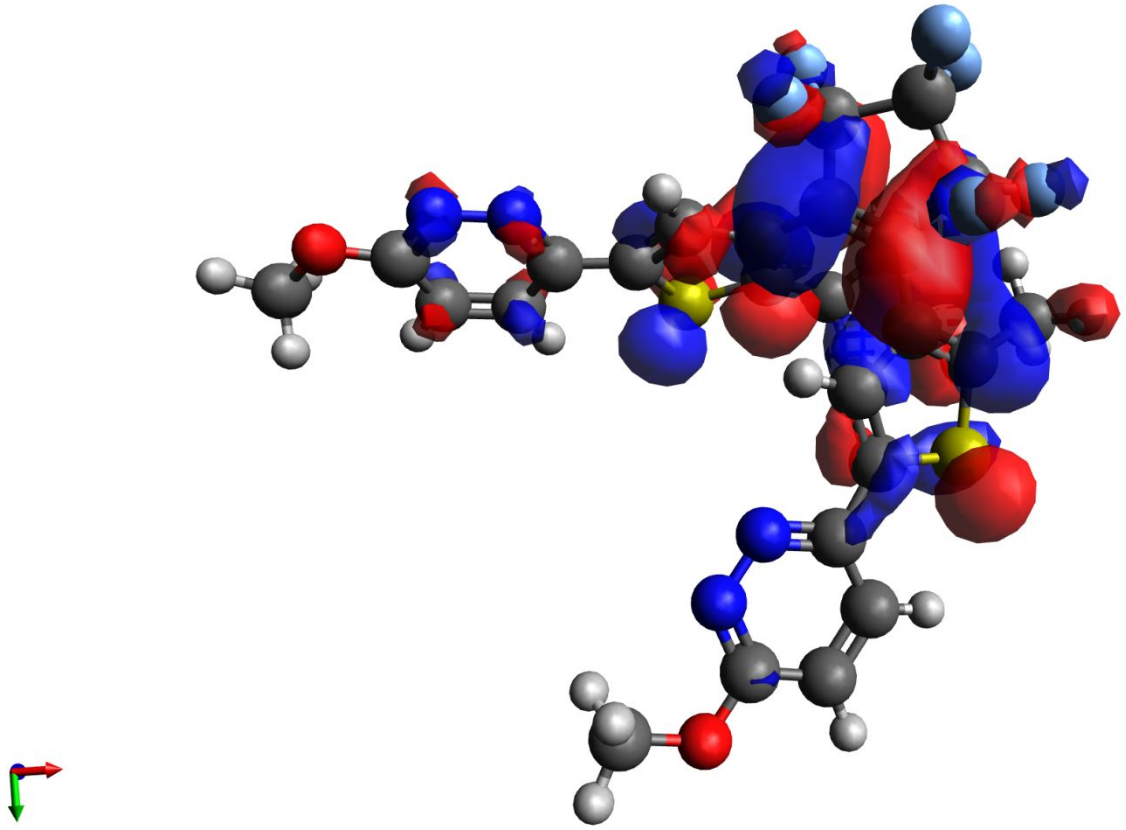

HOMO (-6.032 eV)

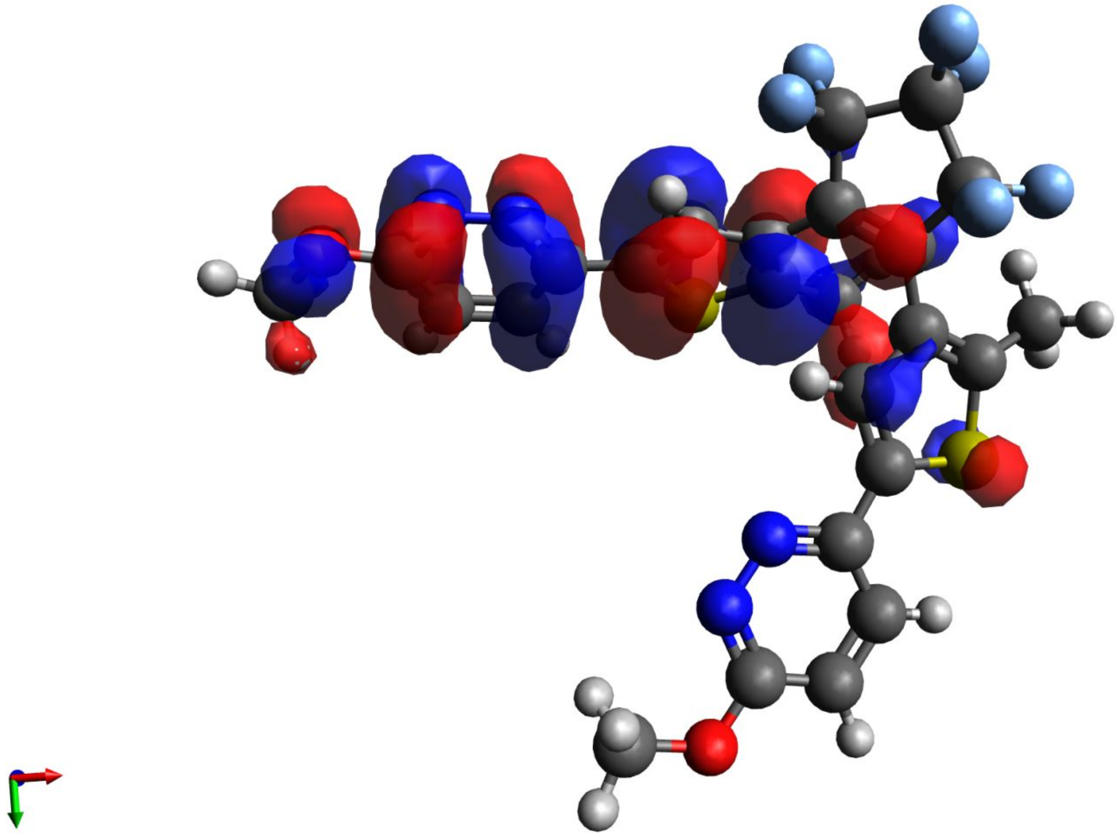

DAE8_closed

Zero-point correction= 0.398059 (Hartree/Particle)
Thermal correction to Energy= 0.432340
Thermal correction to Enthalpy= 0.433284
Thermal correction to Gibbs Free Energy= 0.330940
Sum of electronic and zero-point Energies= -2728.852228
Sum of electronic and thermal Energies= -2728.817947
Sum of electronic and thermal Enthalpies= -2728.817003
Sum of electronic and thermal Free Energies= -2728.919347
0 imaginary frequencies

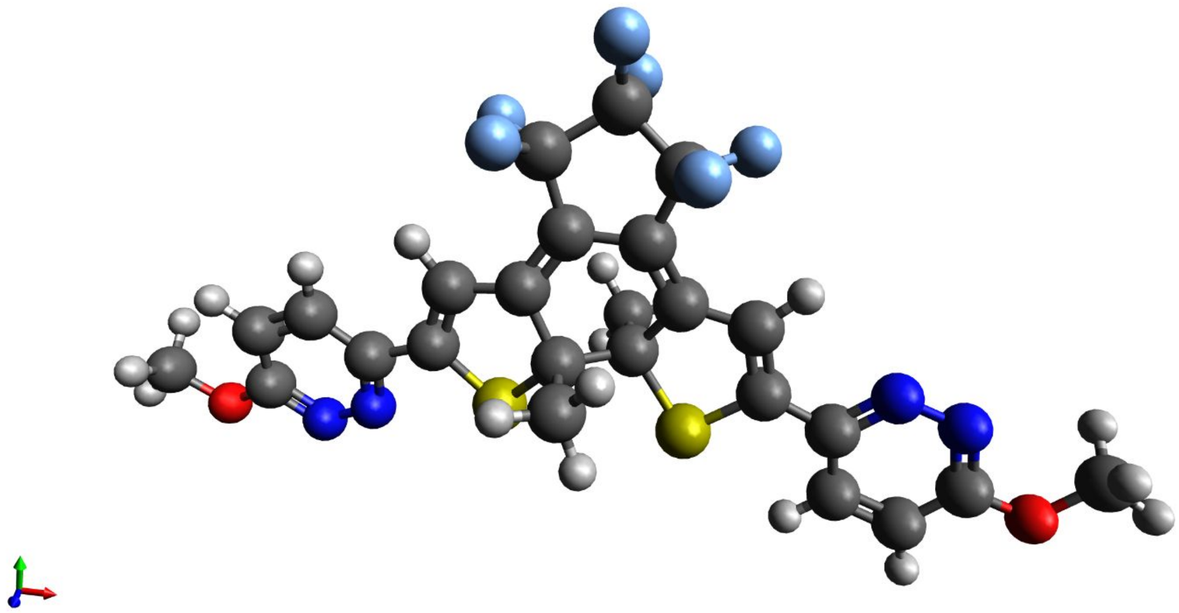

Coordinates (Angstroms)

C	0.5738360000	-0.5656520000	-0.3928360000
C	1.4399940000	0.6687240000	-0.1152050000
C	2.8247280000	0.3695980000	-0.1008990000
C	3.1103920000	-0.9616950000	-0.0729380000
S	1.6930780000	-2.0042540000	-0.0480720000
C	0.7870240000	1.8500300000	0.0366590000
C	-0.6522790000	1.9169290000	0.1086810000
C	-1.1112180000	3.3355460000	0.0524800000
C	0.1764690000	4.1463100000	-0.3017520000
C	1.3577120000	3.2242700000	0.1335400000
C	-1.4114160000	0.7994560000	0.2496340000
F	-2.0961770000	3.5723740000	-0.8605760000
F	-1.6035650000	3.7787830000	1.2521820000
F	0.2096240000	5.3573160000	0.2799180000
F	0.2340970000	4.3214290000	-1.6435200000
F	2.4541020000	3.4437440000	-0.6344720000
F	1.7172110000	3.5549670000	1.4172280000
C	-0.6741900000	-0.5184420000	0.5031810000
S	-1.9213890000	-1.8441450000	0.1362340000
C	-3.2241940000	-0.6675030000	0.1655250000
C	-2.8186180000	0.6321130000	0.2253130000
C	0.2477580000	-0.5844690000	-1.8987390000
C	-0.3490750000	-0.5900600000	2.0085540000
C	4.4481270000	-1.5460680000	-0.0418350000
C	-4.5894170000	-1.1589630000	0.0876690000
N	-4.7149350000	-2.4791380000	-0.0902200000
N	-5.8978950000	-3.0420070000	-0.1888070000
C	-6.9996570000	-2.2963670000	-0.1107920000
C	-6.9557670000	-0.9065170000	0.0817340000
C	-5.7166520000	-0.3310580000	0.1826170000

O	-8.1242740000	-3.0094820000	-0.2319700000
C	-9.3782940000	-2.3245230000	-0.1850670000
C	4.6670190000	-2.9378890000	0.0050890000
C	5.9549460000	-3.3901970000	0.0305300000
C	6.9674280000	-2.4190890000	0.0084490000
N	6.7153710000	-1.1167520000	-0.0339800000
N	5.4655050000	-0.6853240000	-0.0593790000
O	8.2335930000	-2.8403280000	0.0314970000
C	9.2741630000	-1.8479920000	0.0071450000
H	3.6012120000	1.1183720000	-0.0747510000
H	-3.5048000000	1.4658340000	0.1995770000
H	1.1739090000	-0.5384520000	-2.4693660000
H	-0.2892380000	-1.4892000000	-2.1738000000
H	-0.3637950000	0.2773880000	-2.1658570000
H	-1.2668310000	-0.4644030000	2.5809320000
H	0.0943540000	-1.5480720000	2.2698910000
H	0.3441900000	0.2025750000	2.2903760000
H	-7.8524520000	-0.3101370000	0.1507650000
H	-5.6163210000	0.7337510000	0.3369020000
H	-10.1360100000	-3.0928030000	-0.3128500000
H	-9.5214110000	-1.8302160000	0.7782530000
H	-9.4584260000	-1.5961050000	-0.9950390000
H	3.8389720000	-3.6334900000	0.0173540000
H	6.2051190000	-4.4413130000	0.0653830000
H	10.2028190000	-2.4123250000	0.0313240000
H	9.2162640000	-1.2494600000	-0.9016700000
H	9.2040080000	-1.1929880000	0.8752110000

LUMO (-3.017 eV)

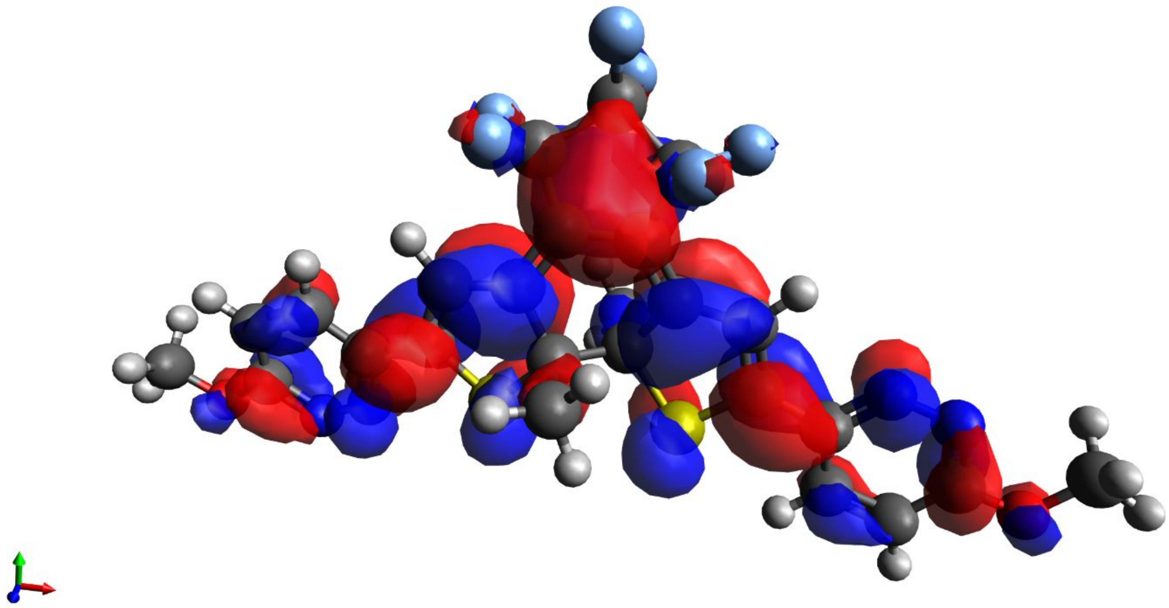

HOMO (-5.248 eV)

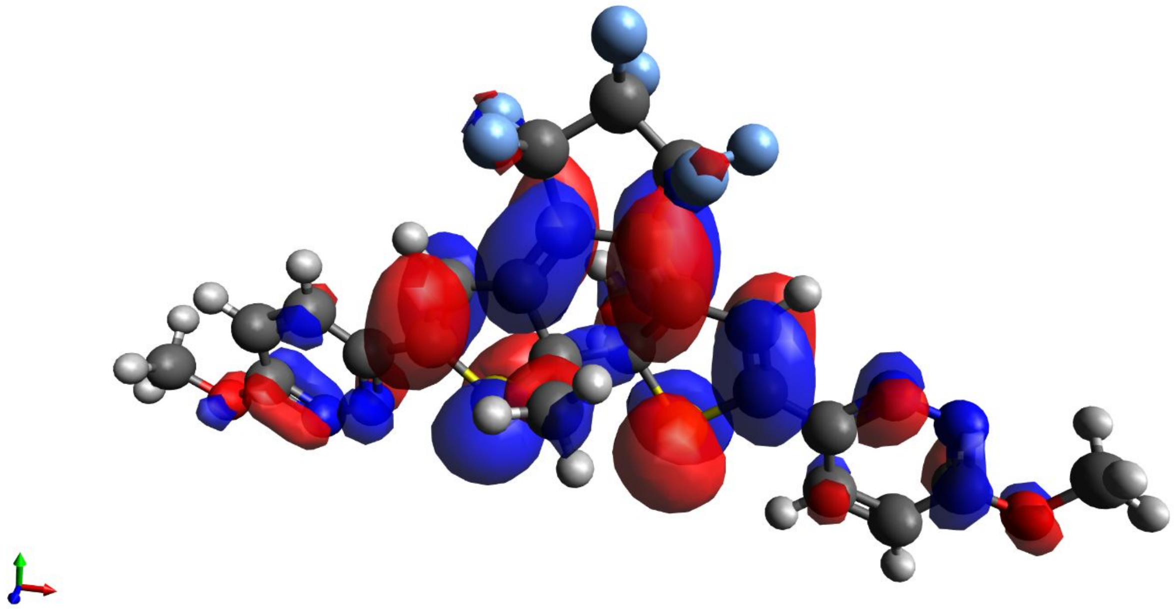

DAE13_open

Zero-point correction= 0.521797 (Hartree/Particle)
Thermal correction to Energy= 0.568369
Thermal correction to Enthalpy= 0.569313
Thermal correction to Gibbs Free Energy= 0.432692
Sum of electronic and zero-point Energies= -3766.529648
Sum of electronic and thermal Energies= -3766.483076
Sum of electronic and thermal Enthalpies= -3766.482132
Sum of electronic and thermal Free Energies= -3766.618753
0 imaginary frequencies

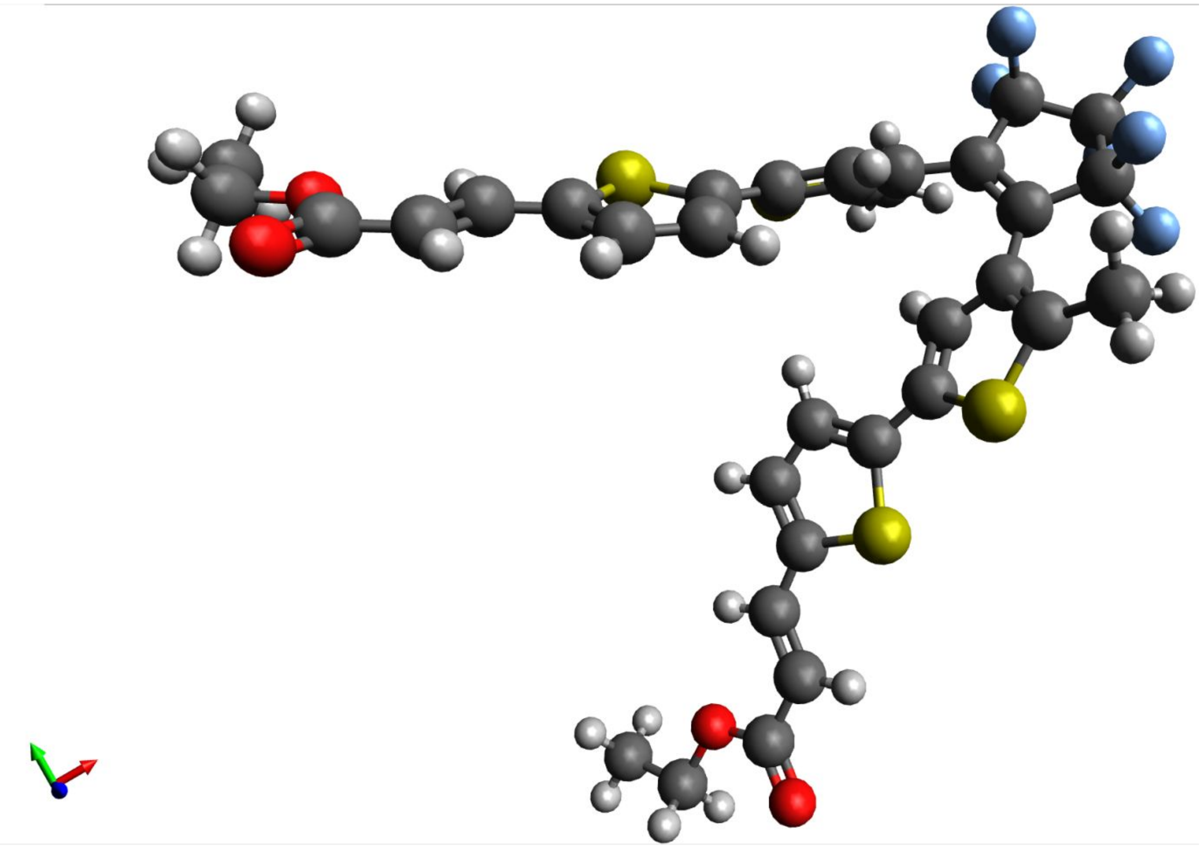

Coordinates (Angstroms)

C	2.4868630000	1.4991570000	-2.2544600000
C	2.9022110000	1.4125350000	-0.9438890000
C	1.9084580000	1.8395580000	-0.0138510000
C	0.7424020000	2.2455100000	-0.6037370000
S	0.8678430000	2.0994540000	-2.3336960000
C	4.1800860000	0.8540610000	-0.4867300000
C	4.3385030000	-0.1872840000	0.3632010000
C	5.7976440000	-0.3657290000	0.7043310000
C	6.5475530000	0.4433970000	-0.3861770000
C	5.4993550000	1.4960440000	-0.8163360000
C	3.2797730000	-1.0642680000	0.8578890000
F	6.1185220000	0.1597530000	1.9256890000
F	6.1949600000	-1.6612840000	0.7201410000
F	6.8324790000	-0.3656620000	-1.4333850000
F	7.6979870000	0.9816230000	0.0519890000
F	5.6835800000	2.6474070000	-0.1005200000
F	5.6491050000	1.8374230000	-2.1197360000
C	3.2254950000	-1.6800860000	2.0975230000
S	1.7597390000	-2.5696630000	2.2704310000
C	1.2096750000	-2.1536100000	0.6745550000
C	2.1310170000	-1.3558150000	0.0577730000
C	3.1833910000	1.0904710000	-3.5101430000
C	4.1746470000	-1.6536940000	3.2505930000
C	-0.4666860000	2.6884940000	0.0466160000

C	-0.0615350000	-2.5867310000	0.1476200000
C	-0.8329290000	-1.9432550000	-0.7957030000
C	-2.0196710000	-2.6349870000	-1.1009130000
C	-2.1783320000	-3.8078360000	-0.3923710000
S	-0.8153090000	-4.0633600000	0.6592750000
S	-1.6224390000	3.7067290000	-0.7387530000
C	-2.6711600000	3.7162500000	0.6477070000
C	-2.1177230000	2.9651490000	1.6653730000
C	-0.8840120000	2.3855050000	1.3283780000
C	-3.2902180000	-4.7080840000	-0.4870810000
C	-3.9085040000	4.4465610000	0.5963890000
C	-3.4690590000	-5.8453580000	0.2056460000
C	-4.7895710000	4.5576490000	1.6049290000
C	-4.6314650000	-6.7262410000	0.0607510000
O	-4.7763840000	-7.7478190000	0.7011100000
O	-5.5239880000	-6.2947340000	-0.8508060000
C	-6.0468230000	5.3064860000	1.5233670000
O	-6.8297190000	5.3935460000	2.4478760000
O	-6.2524090000	5.8883010000	0.3265360000
C	-7.4746790000	6.6437580000	0.1752720000
C	-7.5003890000	7.1973600000	-1.2278580000
C	-6.6980790000	-7.1138420000	-1.0456340000
C	-7.5540810000	-6.4463950000	-2.0934540000
H	2.0671060000	1.8516550000	1.0540310000
H	2.0101520000	-1.0104890000	-0.9559300000
H	3.6896350000	1.9360200000	-3.9806560000
H	3.9323690000	0.3273450000	-3.3036210000
H	2.4772280000	0.6826170000	-4.2347790000
H	4.4112340000	-0.6308740000	3.5438650000
H	3.7457040000	-2.1590040000	4.1155540000
H	5.1117760000	-2.1560720000	3.0063220000
H	-0.5567070000	-0.9913150000	-1.2245220000
H	-2.7576160000	-2.2863760000	-1.8100760000
H	-2.6015690000	2.8260120000	2.6214580000
H	-0.3204930000	1.7393670000	1.9856830000
H	-4.0489450000	-4.4123930000	-1.2028740000
H	-4.1377630000	4.9452220000	-0.3382680000
H	-2.7469780000	-6.1915780000	0.9345560000
H	-4.6188100000	4.0879460000	2.5644310000
H	-8.3213770000	5.9825890000	0.3657800000
H	-7.4920110000	7.4368090000	0.9244290000
H	-8.4156040000	7.7743020000	-1.3746930000
H	-6.6487750000	7.8562060000	-1.4061350000
H	-7.4802800000	6.3947980000	-1.9672400000
H	-6.3826840000	-8.1112850000	-1.3556100000
H	-7.2224670000	-7.2076160000	-0.0935510000
H	-8.4517520000	-7.0438290000	-2.2645810000
H	-7.8631390000	-5.4500620000	-1.7724360000
H	-7.0182000000	-6.3566230000	-3.0400260000

LUMO (-2.412 eV)

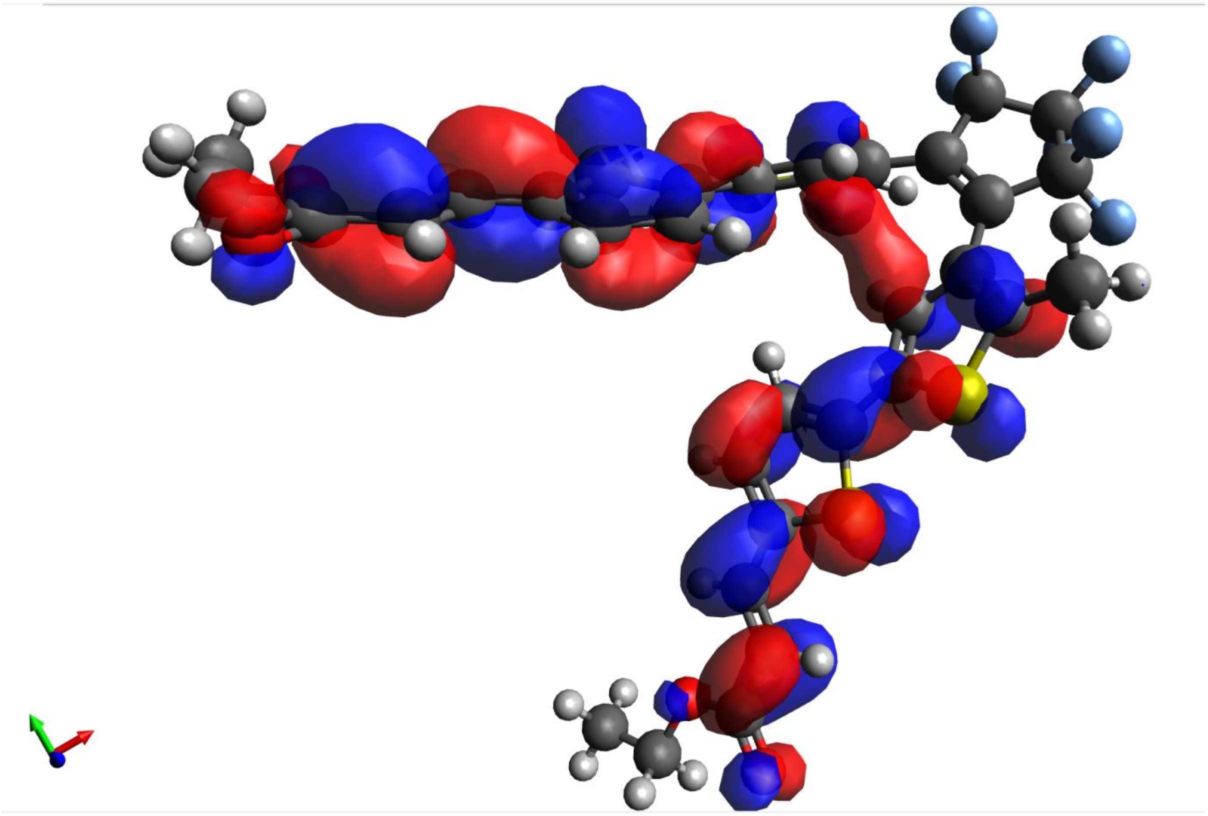

HOMO (-5.786 eV)

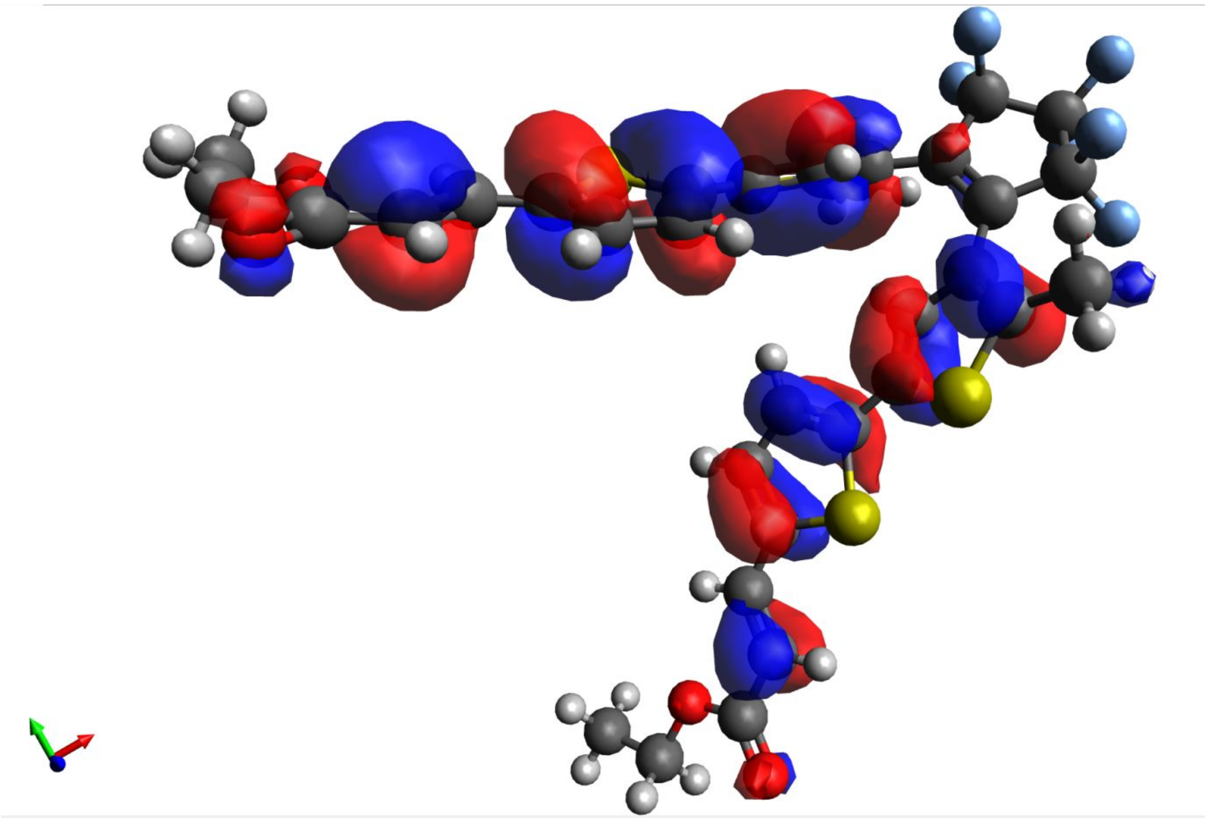

DAE13_closed

Zero-point correction= 0.523130 (Hartree/Particle)
Thermal correction to Energy= 0.568484
Thermal correction to Enthalpy= 0.569429
Thermal correction to Gibbs Free Energy= 0.437444
Sum of electronic and zero-point Energies= -3766.515982
Sum of electronic and thermal Energies= -3766.470628
Sum of electronic and thermal Enthalpies= -3766.469684
Sum of electronic and thermal Free Energies= -3766.601668
0 imaginary frequencies

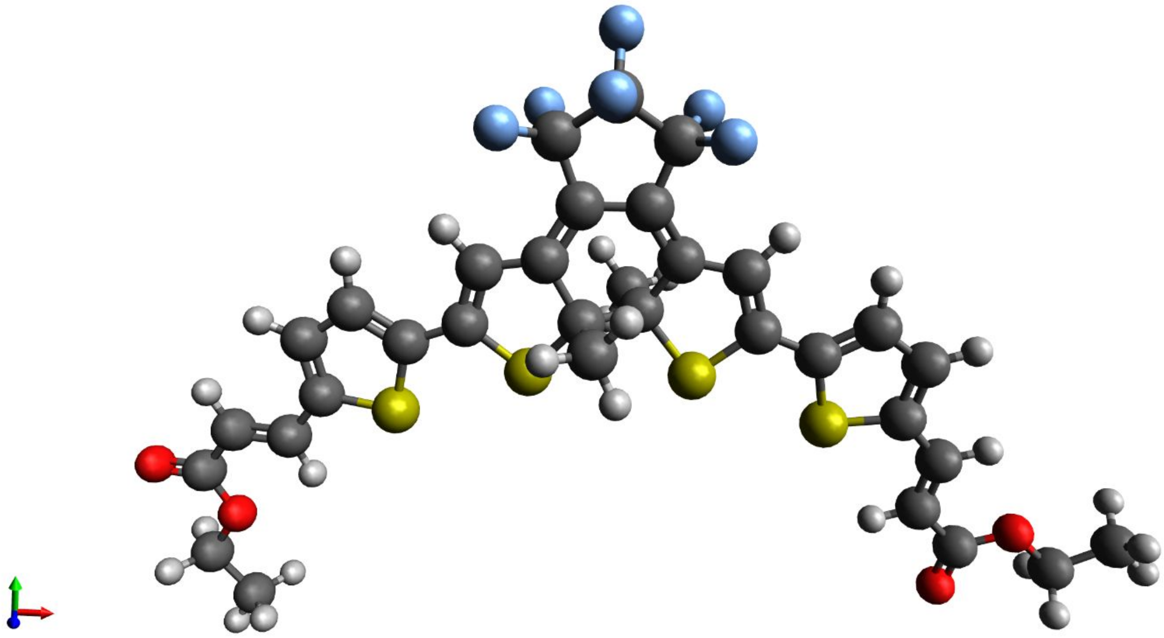

Coordinates (Angstroms)

C	-0.6286150000	0.9607330000	0.4456970000
C	-1.4336790000	2.2287520000	0.1354980000
C	-2.8241600000	1.9966470000	0.1023580000
C	-3.1816220000	0.6741270000	0.0936120000
S	-1.8073130000	-0.4289350000	0.1005520000
C	-0.7221930000	3.3807570000	-0.0199680000
C	0.7131950000	3.3822050000	-0.0788190000
C	1.2380550000	4.7782220000	-0.0531370000
C	-0.0152810000	5.6598260000	0.2551490000
C	-1.2348590000	4.7738820000	-0.1494350000
C	1.4279320000	2.2270450000	-0.1896490000
F	2.2160850000	4.9924020000	0.8726580000
F	1.7738970000	5.1629960000	-1.2539980000
F	0.0090510000	6.8432450000	-0.3810390000

F	-0.0737810000	5.8988610000	1.5866090000
F	-2.3179810000	5.0566440000	0.6207360000
F	-1.5961170000	5.0851570000	-1.4378420000
C	0.6290450000	0.9431760000	-0.4393330000
S	1.8165730000	-0.4235070000	-0.0349900000
C	3.1848670000	0.6858640000	-0.0980710000
C	2.8197180000	2.0047220000	-0.1599410000
C	-0.3185800000	0.9507680000	1.9540620000
C	0.3182620000	0.8642090000	-1.9458640000
C	-4.5179490000	0.1617480000	0.0540350000
C	4.5260930000	0.1846810000	-0.0604550000
C	5.7039880000	0.9114840000	-0.0098930000
C	6.8466560000	0.1036030000	0.0218820000
C	6.5711550000	-1.2525470000	-0.0026120000
S	4.8590530000	-1.5203540000	-0.0701550000
S	-4.8471980000	-1.5374680000	0.0974070000
C	-6.5577190000	-1.2852760000	0.0022270000
C	-6.8421730000	0.0696270000	-0.0566050000
C	-5.7040720000	0.8791580000	-0.0281320000
C	7.5436270000	-2.3065680000	0.0146540000
C	-7.4425590000	-2.4180900000	-0.0015480000
C	7.2967320000	-3.6268500000	-0.0083550000
C	-8.7829590000	-2.3542460000	-0.0671300000
C	8.3351610000	-4.6631320000	0.0082610000
O	8.0865020000	-5.8511640000	-0.0099330000
O	9.5862270000	-4.1696950000	0.0446760000
C	-9.6642480000	-3.5278060000	-0.0724990000
O	-10.8744420000	-3.4467950000	-0.1261670000
O	-9.0027650000	-4.6972790000	-0.0131630000
C	-9.8064580000	-5.8990680000	-0.0178700000
C	-8.8679990000	-7.0778210000	0.0547550000
C	10.6630580000	-5.1340950000	0.0592810000
C	11.9626220000	-4.3689080000	0.0932870000
H	-3.5508080000	2.7936150000	0.0510340000
H	3.5428320000	2.8069550000	-0.1550680000
H	-1.2473930000	1.0504210000	2.5132940000
H	0.1696590000	0.0252270000	2.2501070000
H	0.3324540000	1.7851190000	2.2150260000
H	1.2465300000	0.9405570000	-2.5096160000
H	-0.1675520000	-0.0752720000	-2.1988240000
H	-0.3343660000	1.6846290000	-2.2444740000
H	5.7279200000	1.9909760000	0.0060830000
H	7.8575440000	0.4845300000	0.0613650000
H	-7.8495310000	0.4547590000	-0.1183980000
H	-5.7335870000	1.9580190000	-0.0685840000
H	8.5733570000	-1.9695480000	0.0491990000
H	-6.9784800000	-3.3958010000	0.0521970000
H	6.2885350000	-4.0204450000	-0.0418230000
H	-9.3079770000	-1.4099460000	-0.1212620000
H	-10.4865500000	-5.8685080000	0.8345850000
H	-10.4074630000	-5.9141580000	-0.9282510000

H	-9.4467000000	-8.0036070000	0.0525340000
H	-8.1920130000	-7.0955350000	-0.8018820000
H	-8.2717780000	-7.0499640000	0.9684310000
H	10.5466420000	-5.7755180000	0.9340710000
H	10.5840720000	-5.7602680000	-0.8305990000
H	12.7981260000	-5.0715710000	0.1050690000
H	12.0656600000	-3.7304220000	-0.7858340000
H	12.0282130000	-3.7452710000	0.9865390000

LUMO (-3.307 eV)

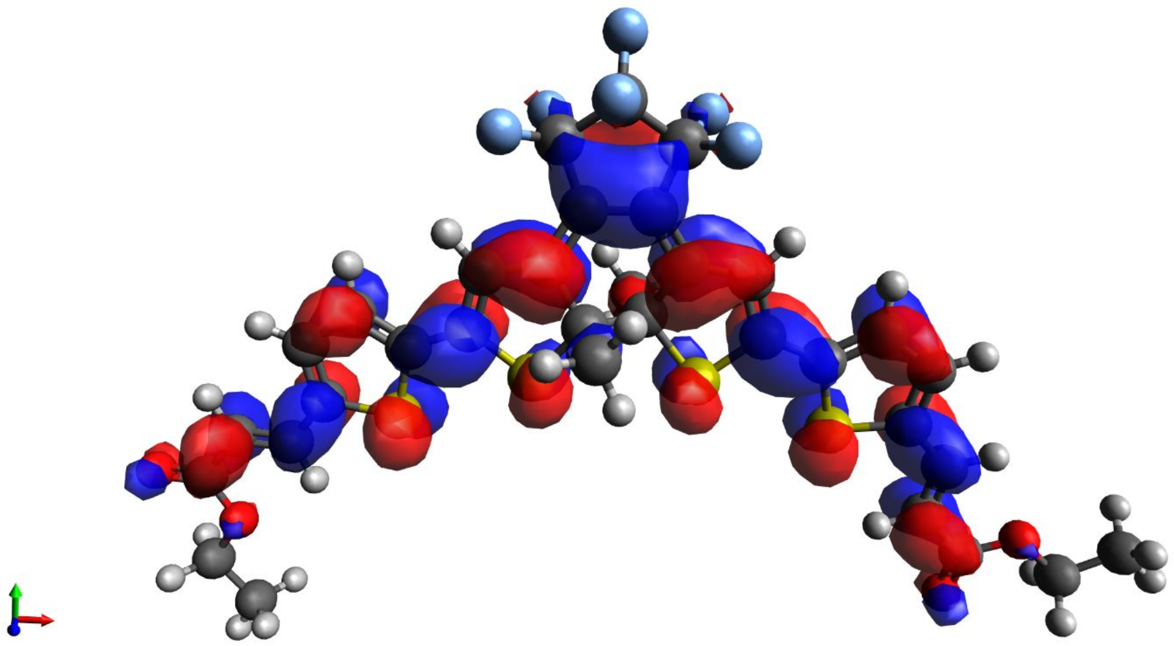

HOMO (-5.158 eV)

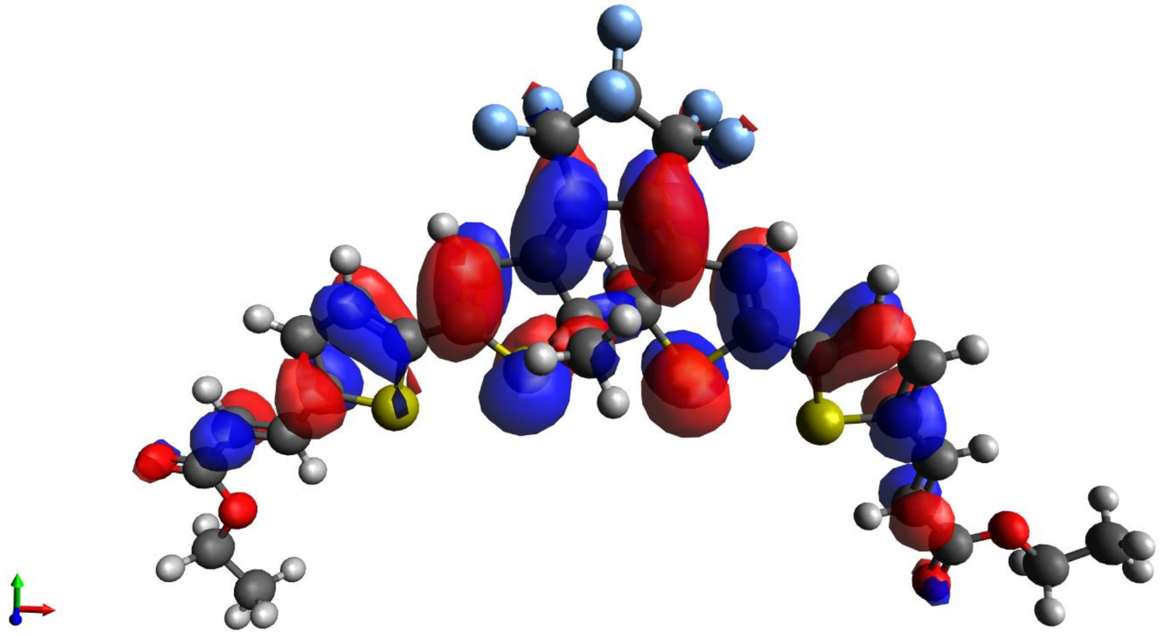

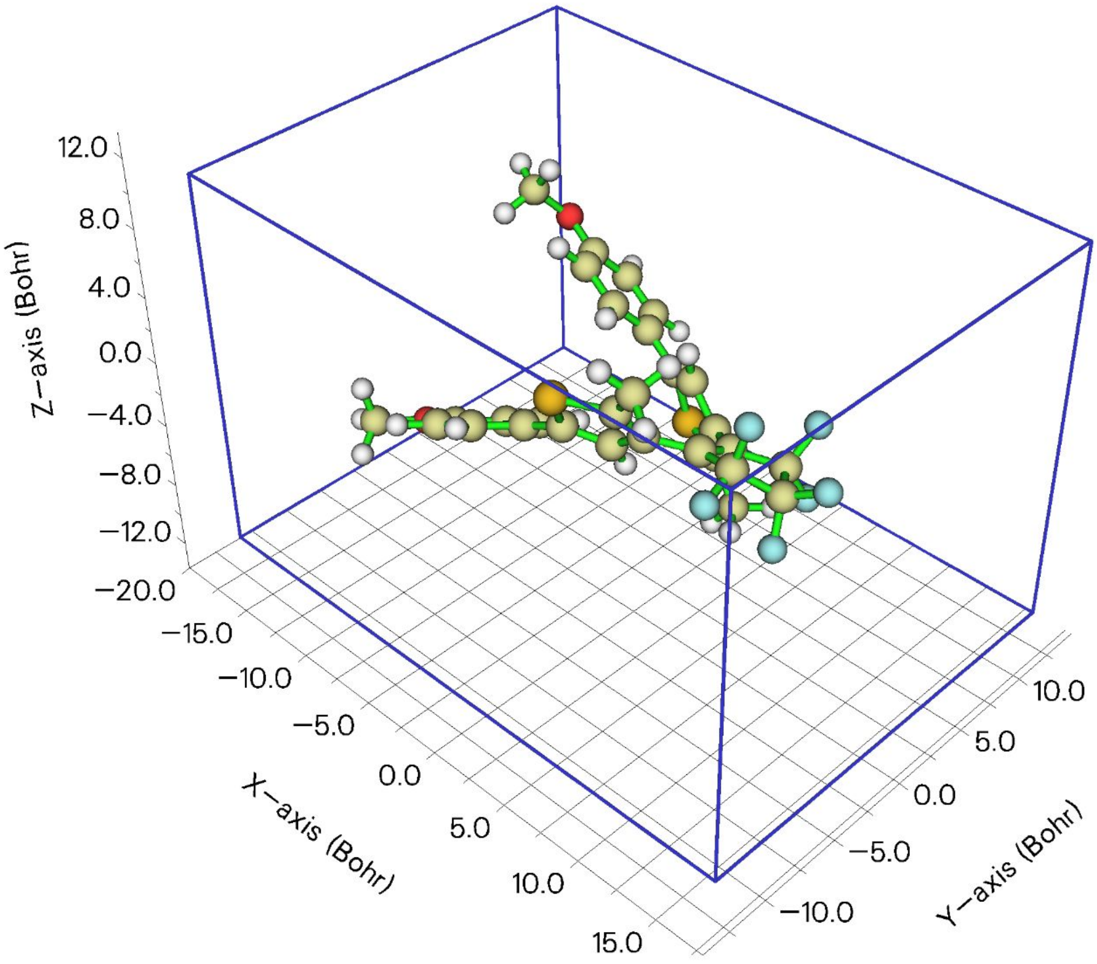

12.0
8.0
4.0
0.0
−4.0
−8.0
−12.0
−20.0
Z−axis (Bohr)
−15.0
−10.0
−5.0
0.0
5.0
10.0
15.0
X−axis (Bohr)
10.0
5.0
0.0
−5.0
−10.0
Y−axis (Bohr)

Figure S1: Surface visualization of DAE1-open.

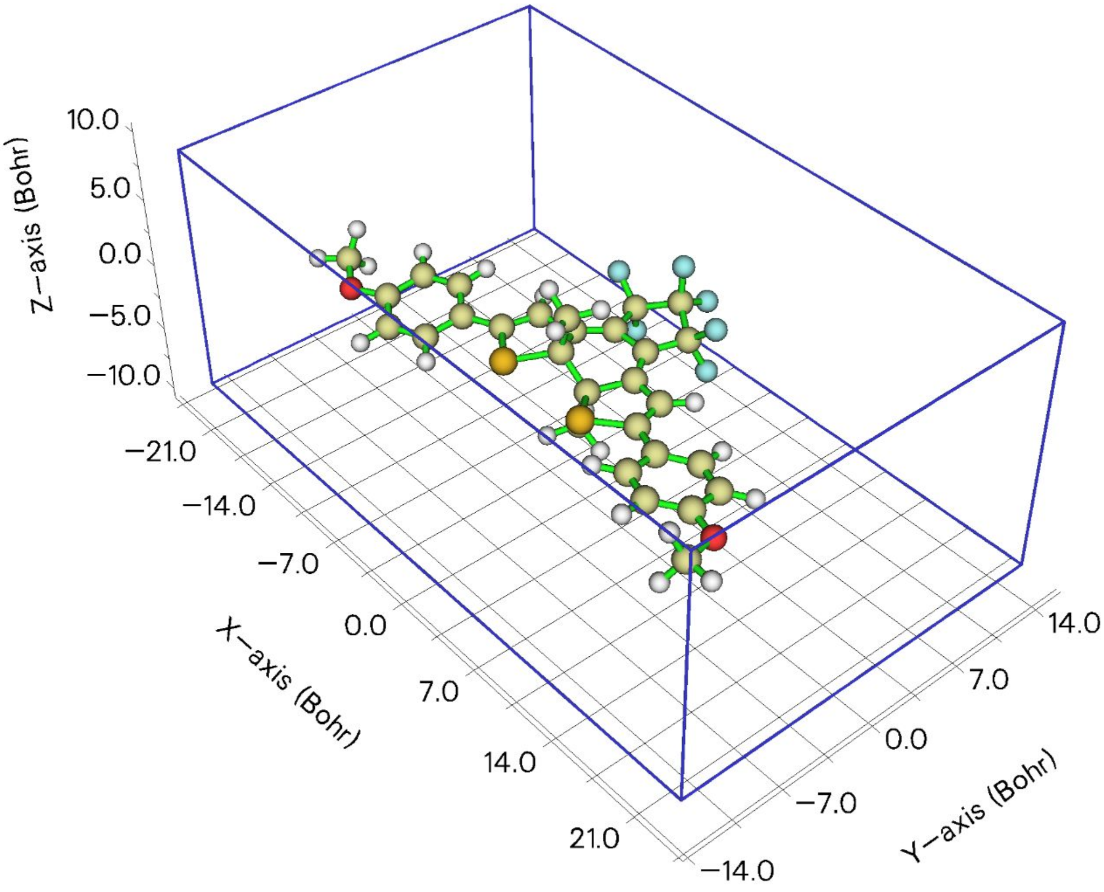

Figure S2: Surface visualization of DAE1-closed.

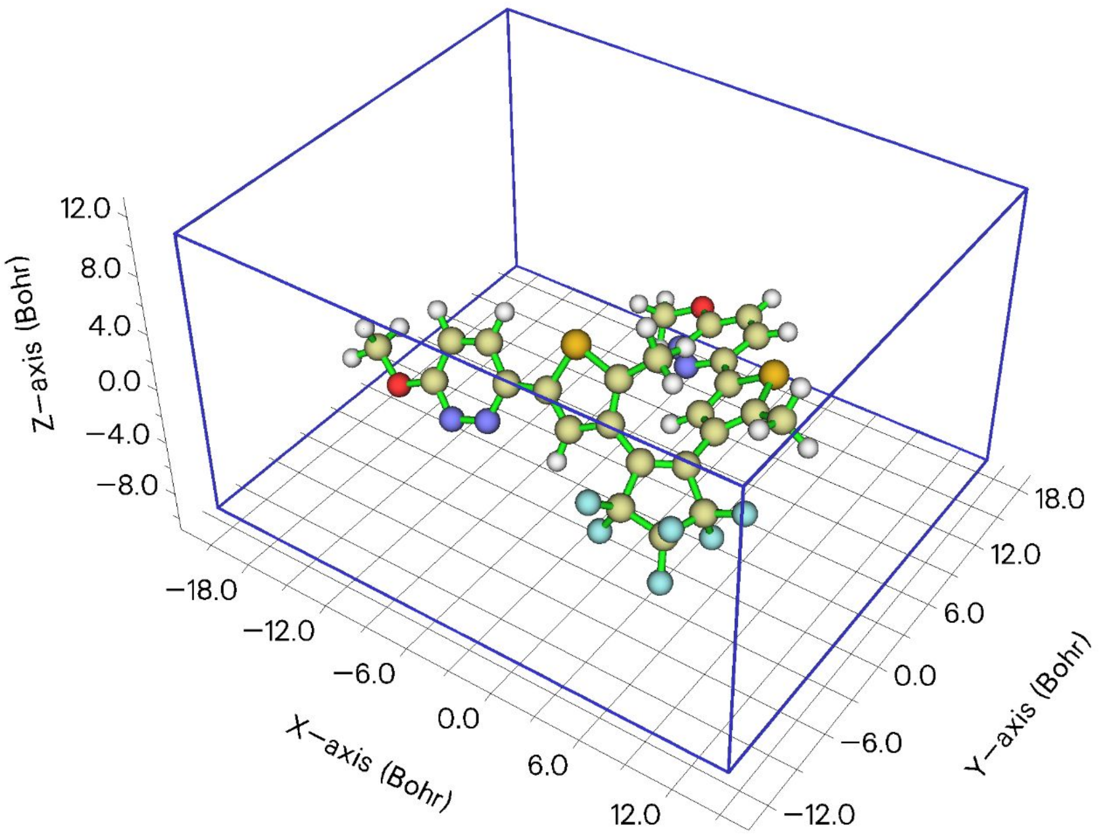

Figure S3: Surface visualization of DAE8-open.

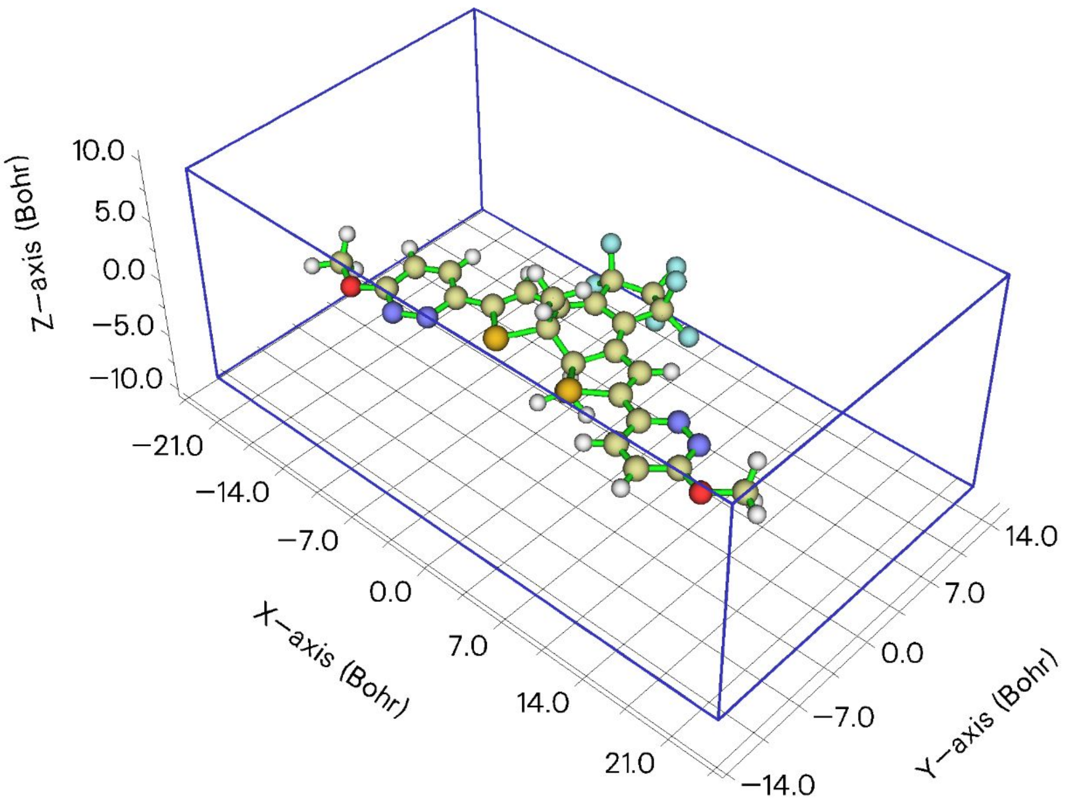

Figure S4: Surface visualization of DAE8-closed.

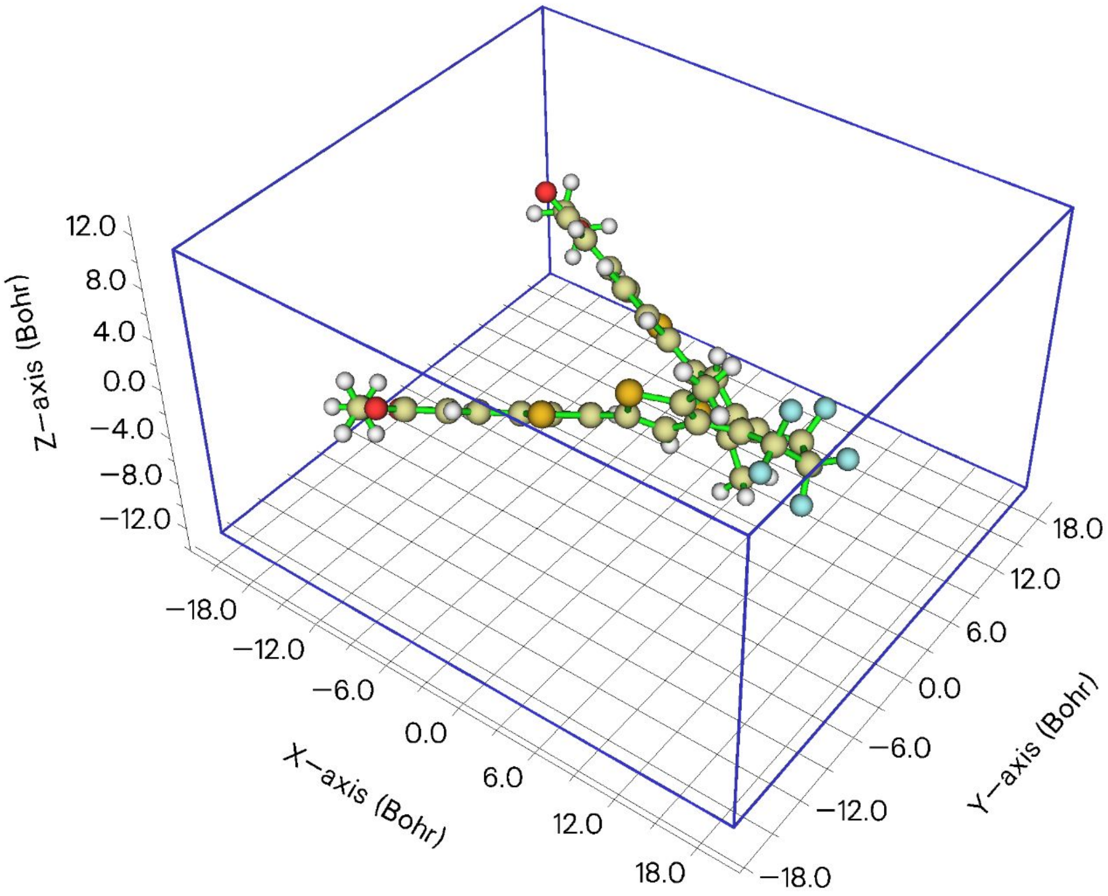

Figure S5: Surface visualization of DAE13-open.

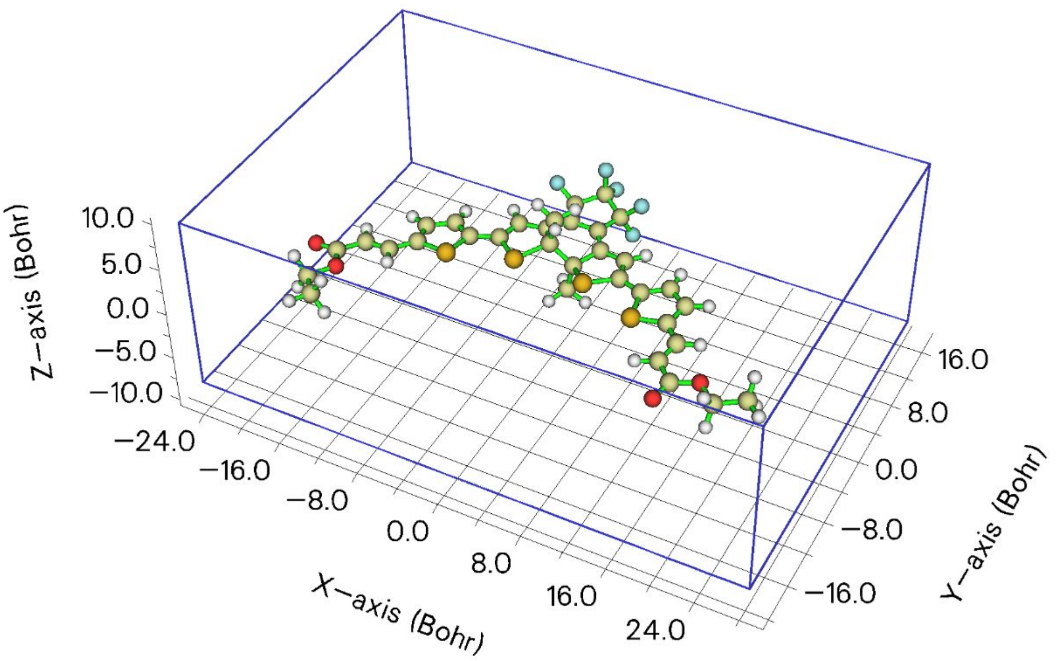

Figure S6: Surface visualization of DAE13-closed.

Table S1: ΔG-calculations using B3LYP/Def2TZVP GD3BJ

DAE	Vacuum	Anisole	PMMA
**1o [Eh]**	-2664.757568	-2664.782176	-2664.779444
**1c [Eh]**	-2664.741437	-2664.765761	-2664.762009
**ΔG(1) [kJ·mol^{-1}]**	42.35192	43.09757	45.77558
**8o [Eh]**	-2728.918757	-2728.944000	-2728.940442
**8c [Eh]**	-2728.897774	-2728.923576	-2728.919347
**ΔG(8) [kJ·mol^{-1}]**	55.09085	53.62319	55.3849
**13o [Eh]**	-3766.587607	-3766.622563	-3766.618753
**13c [Eh]**	-3766.571036	-3766.605538	-3766.601668
**ΔG(13) [kJ·mol^{-1}]**	43.50714	44.69912	44.85665

Table S2: ΔG-calculations using B3LYP/36G d,p

DAE	Vacuum	Anisole	PMMA
**1o [Eh]**	-2663.973916	-2663.996697	-2663.993162
**1c [Eh]**	-2663.96081	-2663.984607	-2663.98155
**ΔG(1) [kJ·mol^{-1}]**	34.40979	31.74228	30.48729
**8o [Eh]**	-2728.101995	-2728.125633	-2728.121724
**8c [Eh]**	-2728.08757	-2728.111331	-2728.107446
**ΔG(8) [kJ·mol^{-1}]**	37.87282	37.54989	37.48687
**13o [Eh]**	-3765.61556	-3765.648255	-3765.644559
**13c [Eh]**	-3765.603091	-3765.635609	-3765.632101
**ΔG(13) [kJ·mol^{-1}]**	32.73735	33.20206	32.70847

Table S3: Change of the distance of C2-C2' positions in PMMA (B3LYP/Def2TZVP)

DAE	Open form [Å]	closed form [Å]	Δx [Å]
**1**	5.455	1.538	3.917
**8**	4.097	1.537	2.560
**13**	5.440	1.538	3.902

Table S4: HOMO/LUMO Gap, in PMMA B3LYP/Def2TZVP

DAE	HOMO [eV]	LUMO [eV]	Gap [eV]
**1o**	-5.603	-1.975	3.628
**1c**	-4.946	-2.683	2.263
**8o**	-6.032	-1.943	4.089
**8c**	-5.248	-3.017	2.231
**13o**	-5.786	-2.412	3.374
**13c**	-5.158	-3.307	1.851

Table S5: Change in volume and surface area of the selected DAEs

<table>
<tr><th>DAE</th><th>Volume [Bohr³]</th><th>ΔV [Bohr³]</th><th>Surface [Bohr²]</th><th>ΔA [Bohr²]</th></tr>
<tr><td>1o</td><td>4615.901</td><td rowspan="2">72.689</td><td>1891.438</td><td rowspan="2">10.115</td></tr>
<tr><td>1c</td><td>4543.211</td><td>1901.553</td></tr>
<tr><td>8o</td><td>4410.404</td><td rowspan="2">81.104</td><td>1890.759</td><td rowspan="2">52.220</td></tr>
<tr><td>8c</td><td>4329.299</td><td>1838.539</td></tr>
<tr><td>13o</td><td>5783.070</td><td rowspan="2">76.224</td><td>2440.968</td><td rowspan="2">7.148</td></tr>
<tr><td>13c</td><td>5706.846</td><td>2448.116</td></tr>
</table>

Table S6: Diameter change of the system in Angström

DAE	Diameter [Å]	Δd[Å]
**1o**	17.137	
		5.495
**1c**	22.632	
**8o**	17.662	
		5.074
**8c**	22.736	
**13o**	20.932	
		5.424
**13c**	26.356	

Table S7: Hammett parameters and calculated excitation energy differences used for the correlation plot (Figure 4). Hammett parameters have been calculated using the webtool published by Ertl.[9]

DAE	σ_p	σ_m	$\frac{E_{T,R}-E_{T,H}}{2{,}303\cdot R\cdot T}$ (open)	$\frac{E_{T,R}-E_{T,H}}{2{,}303\cdot R\cdot T}$ (closed)
**1**	-0.037	0.029	-1.76652	-0.54029
**2**	0.15	0.161	-1.74109	-0.5585
**3**	0.025	0.094	-2.26277	-0.61205
**4**	0.188	0.199	-1.96381	-0.60618
**5**	0.372	0.303	-3.00893	-0.72552
**6**	0.089	0.129	-2.08183	-0.55245
**7**	0.068	0.115	-1.76652	-0.55245
**8**	0.18	0.141	-1.98773	-0.68664
**9**	0.09	0.147	-2.34979	-0.63534
**10**	0.04	0.116	-2.41362	-0.61205
**11**	0.385	0.344	-2.86566	-0.81638
**12**	0.298	0.286	-2.99138	-0.78487

Table S8: Location of uv and visible light maxima as well as the isosbestic point of the DAEs used in this work.

DAE	λ_{isos} [nm]	$\lambda_{UV,max,open}$ [nm]	$\lambda_{Vis,max,closed}$ [nm]
**1**	318	290	591
**2**	319	289	594
**3**	328	308	603
**4**	318	298	602
**5**	335	347	627
**6**	319	302	591
**7**	314	290	591
**8**	330	297	611
**9**	313	313	607
**10**	335	319	603
**11**	345	341	637
**12**	382	346	633
**13**	403	396	659

Table S9: Specification and seller details for the polymers used for film preparation. $\mathbf{T_g}$ values marked with * have been obtained by DSC measurement.

Polymer	Seller	M_w (g/mol)	T_g (°C)
**PMMA**	Sigma Aldrich	120000	110
**ZEONEX480R**	Nippon Zeon Co. Ltd	480000	138
**PBMA**	Sigma Aldrich	200000	34*
**PS**	Pressure Chemical Co.	390000	100
**PODMA**	Röhm GmbH	900000	33
**P2VP**	Polymer Source	8600	68
**P2VP-*b*-PMMA**	Polymer Source	160000/170000	100*
**PMMA2000**	Fluka	2000	40*
**OH-PMMA**	Polymer Source	2000	38*
**NH_2-PMMA**	Polymer Source	6700	34*
**SBDS**	Polymer Source	14000/73000/15000	-65

Synthesis of MMA/BA co-polymers

The ratios of methyl-methacrylate (MMA, 99% with 30 ppm MEHQ as inhibitor, Aldrich) and butylacrylate (BA, stabilized, Sigma-Aldrich) were calculated using the Fox-equation (Eq. S1) and can be found in table S10.[10]

$$\frac{1}{T_g} = \frac{\omega_1}{T_{g,1}} + \frac{\omega_2}{T_{g,2}} \qquad \text{Eq. S1}$$

T_g is the glass transition temperature of the copolymer, ω_1 and ω_2 are the mass equivalents of the monomers and $T_{g,1}$ and $T_{g,2}$ are the glass transition temperatures of the coresponding homo-polymers.

MMA and BA were purified using an inhibitor remover column (Al_2O_3, Aldrich). After dissolving the monomers in 9.2 mL xylene (Merck), they were flushed with argon for 15 minutes to purge oxygen from the solution and 20 mg 2,2′-azobis(2-methylpropionitrile) (AIBN, purum, Sigma-Aldrich) was added. The solution was put in a heating block for 24 h at 70 °C. Subsequently the polymer was precipitated in 100 mL methanol and washed a few times. Finally, the polymers were dried in a vacuum oven at 50 °C and 100 mbar for 24 h. Glass transition temperatures were obtained via DSC measurement (DSC 1, Mettler Toledo).

Table S10: Results of synthesized polymers with targeted and resulted glass transition temperature.

Target T_g in °C	Measured T_g in °C	MMA in mL	BA in mL
-20	-9	0.66	1.54
0	17	0.97	1.21
20	29	1.23	0.93
40	61	1.47	0.69
60	75	1.67	0.48
80	88	1.85	0.29
100	85	2.02	0.12
115	109	2.13	0

Modified Protocol for spincoating AMLs

1. Sapphire substrate (10.0 mm ∅) was attached onto the spincoater
2. 30 µl of a solution of ZEONEX480R (12 wt%) in 1,2-Dichlorobenzene was pipetted to the Sapphire substrate
3. Sapphire substrate was spincoated for 1 min at 2500 rpm
4. The substrate was dried at RT for 5 min
5. Onto the precoated substrate 30 µl of a solution of DAE (25 wt%) and ZEONEX480R (75 wt%) in 1,2-Dichlorobenzene (12 wt% of the solids in solvent) was pipetted to the substrate
6. Substrate was again spincoated for 1 min at 2500 rpm
7. Substrate was further dried at RT over night in the dark before further use

Setup for stability measurements of photochromes

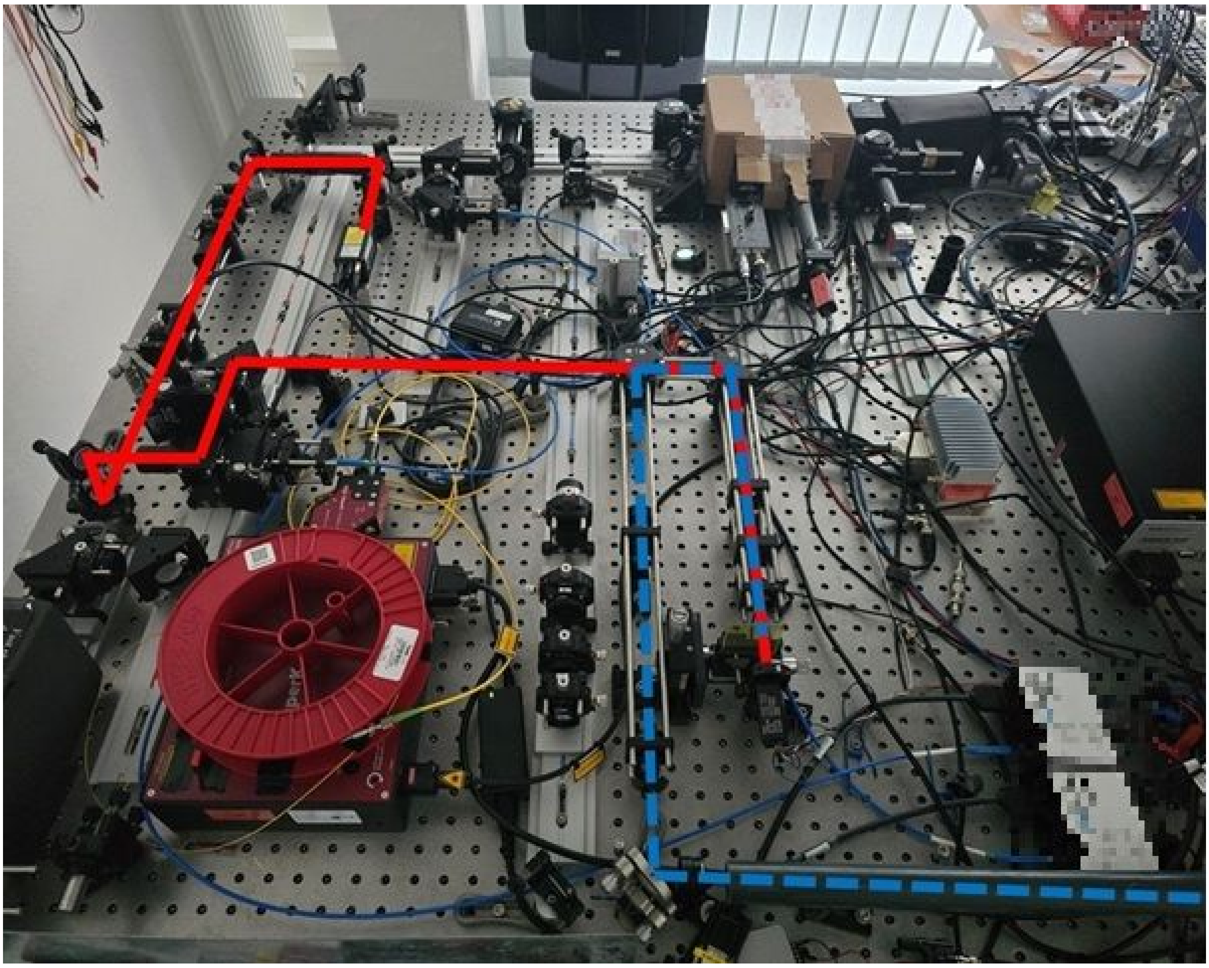

Figure S7: Setup for stability measurements of photochromes (*switching mode*).

References

(1) Nagorny, S.; Weingartz, T.; Namyslo, J. C.; Adams, J.; Schmidt, A. Correlation between Absorption and Substitution of Photochromic 1,2-Bis(Thienyl)Ethenes (BTEs) Using Modified Spectroscopic Hammett Equations. *Eur. J. Org. Chem.* **2023**, *26* (1), e202200996. https://doi.org/10.1002/ejoc.202200996.

(2) Nagorny, S.; Lederle, F.; Udachin, V.; Weingartz, T.; Hübner, E. G.; Dahle, S.; Maus-Friedrichs, W.; Adams, J.; Schmidt, A. Switchable Mesomeric Betaines Derived from Pyridinium-Phenolates and Bis(Thienyl)Ethane. *Eur. J. Org. Chem.* **2021**, *2021* (22), 3178–3189. https://doi.org/10.1002/ejoc.202100279.

(3) Nagorny, S.; Schewe, M.; Weingartz, T.; Eitzeroth, A.; Adams, J.; Rembe, C.; Schmidt, A. Stabilities of Bis(Thienyl)Ethenes in Polymethyl Methacrylate (PMMA) Coatings as Absorbance Modulation Layers for Nanoscale Imaging. *Mater. Adv.* **2024**, *5* (1), 159–170. https://doi.org/10.1039/D3MA00791J.

(4) Ding, M.; Reuven, J. A.; Hones, A. C.; Fox, M. A.; Steel, P. G. Iridium-Catalysed C−H Borylation of Fluoroarenes: Insights into the Balance between Steric and Electronic Control of Regioselectivity. *Eur. J. Org. Chem.* **2022**, *2022* (47), e202201005. https://doi.org/10.1002/ejoc.202201005.

(5) Steppert, A.-K.; Mikosch, A.; Haraszti, T.; Göstl, R.; Kuehne, A. J. C. Reversible Laser Threshold Modulation in Dithienylethene Conjugated Polymer Blends: A Concept for q-Switching in Organic DFB Lasers. *ACS Photonics* **2019**, *6* (2), 558–564. https://doi.org/10.1021/acsphotonics.8b01641.

(6) Sun, F.; Cui, S.; Liu, G.; Zheng, C.; Pu, S. Photochromism of Isomeric Diarylethenes with a Methylpyridine Substituent. *J. Mol. Struct.* **2015**, *1086*, 131–137. https://doi.org/10.1016/j.molstruc.2015.01.015.
(7) Oggioni, L.; Toccafondi, C.; Pariani, G.; Colella, L.; Canepa, M.; Bertarelli, C.; Bianco, A. Photochromic Polyurethanes Showing a Strong Change of Transparency and Refractive Index. *Polymers* **2017**, *9* (9). https://doi.org/10.3390/polym9090462.
(8) Branda, N. R.; Peters, A.; Wigglesworth, A. J. Photochromic and Electrochromic Compounds and Methods of Synthesizing and Using Same. WO2004015024A1, February 19, 2004.
(9) Ertl, P. A Web Tool for Calculating Substituent Descriptors Compatible with Hammett Sigma Constants. *Chemistry–Methods* **2022**, *2* (12), e202200041. https://doi.org/10.1002/cmtd.202200041.
(10) G, F. T. Influence of Diluent and of Copolymer Composition on the Glass Temperature of a Polymer System. *Bull Am Phs Soc* **1952**, *1*, 123.
